# Discovery of a Hydroxylamine-Based Brain-Penetrant EGFR Inhibitor for Metastatic Non-Small-Cell Lung Cancer

**DOI:** 10.1021/acs.jmedchem.3c01669

**Published:** 2023-11-07

**Authors:** Jarvis Hill, Robert M. Jones, David Crich

**Affiliations:** † Department of Pharmaceutical and Biomedical Sciences, University of Georgia, 250 West Green Street, Athens, Georgia 30602, United States; ‡ Department of Chemistry, University of Georgia, 302 East Campus Road, Athens, Georgia 30602, United States; § P.O. Box 568, Oakley, Utah 84055-0568, United States; ∥ Complex Carbohydrate Research Center, University of Georgia, 315 Riverbend Road, Athens, Georgia 30602, United States

## Abstract

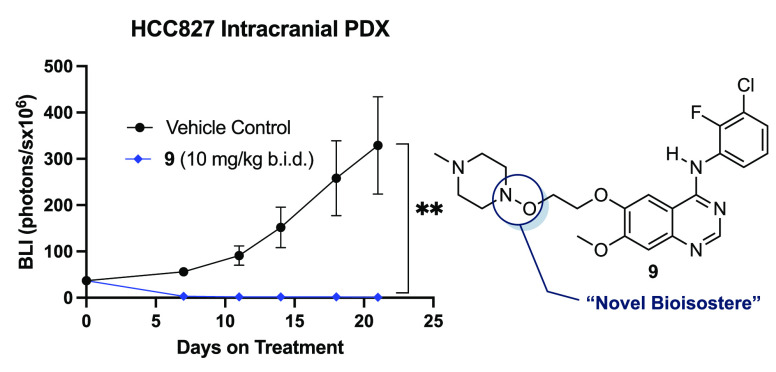

Metastases to the brain remain a significant problem in lung cancer, as treatment by most small-molecule targeted therapies is severely limited by efflux transporters at the blood–brain barrier (BBB). Here, we report the discovery of a selective, orally bioavailable, epidermal growth factor receptor (EGFR) inhibitor, **9**, that exhibits high brain penetration and potent activity in osimertinib-resistant cell lines bearing L858R/C797S and exon19del/C797S EGFR resistance mutations. *In vivo*, **9** induced tumor regression in an intracranial patient-derived xenograft (PDX) murine model suggesting it as a potential lead for the treatment of localized and metastatic non-small-cell lung cancer (NSCLC) driven by activating mutant bearing EGFR. Overall, we demonstrate that an underrepresented functional group in medicinal chemistry, the trisubstituted hydroxylamine moiety, can be incorporated into a drug scaffold without the toxicity commonly surmised to accompany these units, all while maintaining potent biological activity and without the molecular weight creep common to drug optimization campaigns.

## Introduction

**Figure 1:**
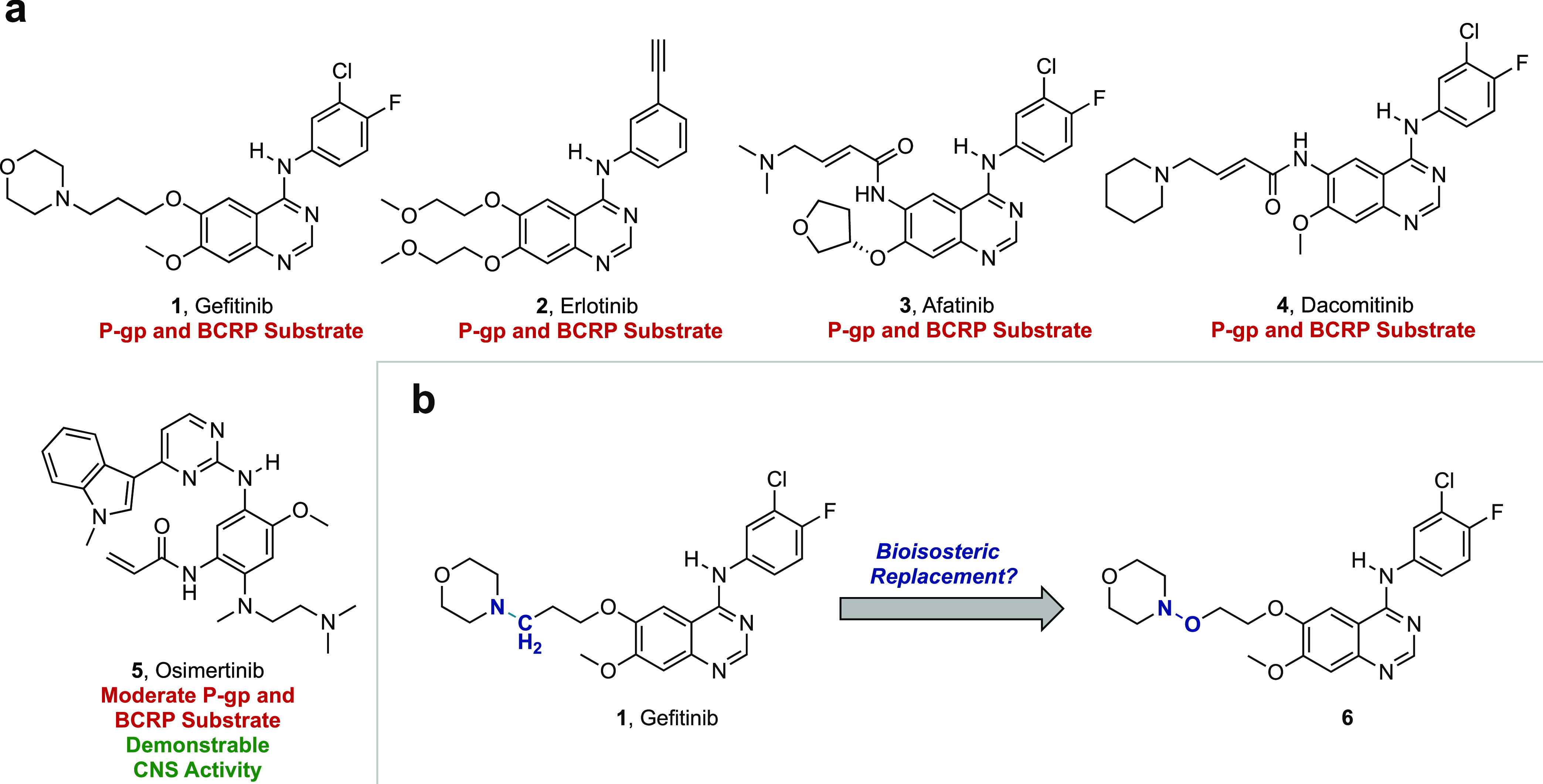
Overview of selected EGFR inhibitors and bioisosteric strategy used in this study. (A) Chemical structures of select approved EGFR inhibitors and their efflux status against common efflux transporters. (B) Proposed bioisosteric replacement.

Non-small-cell lung cancer (NSCLC) is a disease that comprises approximately 85% of all newly diagnosed lung cancers (Figure 1a).^1−3^ Activating mutations in epidermal growth factor receptor (EGFR, ERBB1, HER1), which are detected in 10 to 30% of patients with NSCLC, such as in-frame exon 19 deletions (del E746_A750) and a point mutation on exon 21 (L858R), confer sensitivity to reversible first-generation EGFR-targeted tyrosine kinase inhibitors (TKIs) such as gefitinib (**1**).^2−5^ Resistance to first-generation TKIs is mainly due to the acquisition of a secondary T790M “gatekeeper” mutation on exon 20, detected in 60% of drug-resistant NSCLC, which enhances the binding of adenosine triphosphate (ATP) to the EGFR kinase.^6−8^ Subsequent second- and third-generation irreversible covalent inhibitors overcome T790M resistance by targeting a conserved cysteine (C797).^9−11^ They are inactivated, however, through a C797S point mutation that converts the nucleophilic cysteine to a serine and blocks covalent binding.^12,13^ Additionally, patients with activating EGFR mutations who are treated with frontline osimertinib can develop unique secondary mutations such as L858R/C797S or del E746_A750/C797S, which confer resistance.^14,15^ Unfortunately in NSCLC, up to 40% of patients will also develop brain metastases (BM), and this number is likely to increase as treatment options continue to improve life expectancy for patients with advanced disease.^16^ As such, BM are a significant risk and result in a poor prognosis for NSCLC patients being treated with poorly central nervous system (CNS) penetrant TKIs.^16−20^ Multiple studies have revealed the low CNS penetrability of a majority of marketed EGFR TKIs due to active efflux by transporters such as P-glycoprotein (P-gp) and breast cancer resistance protein (BCRP), which are highly enriched at the blood–brain barrier (BBB), and in combination with tight junctions, ultimately exclude up to 98% of all drugs from the CNS.^17−20^ Thus, future treatment options for NSCLC would benefit from improved CNS disposition, thereby enabling treatment of the local disease and of ensuing BMs. Numerous strategies and guidelines have been proposed to reduce drug efflux and increase CNS permeability, such as the cLogBB optimization approach, that incorporates lipophilicity and total polar surface area (TPSA), or the multiparameter optimization (MPO) method, but frequently a resource-intensive iterative optimization strategy is employed.^20−23^ Instead, we were drawn to the differences in p*K*_a_ values between tertiary amines and even weakly basic ones such as *N*-methylmorpholine (p*K*_a_ = 7.4)^24^ and hydroxylamine itself (p*K*_a_ = 5.9).^25^ We hypothesized that the bioisosteric replacement of the *N*-alkylmorpholine unit in **1** by an *N*-noralkoxy morpholine moiety, effectively converting the morpholine group into a hydroxylamine, would attenuate the basicity and modify the lipophilicity of the compound leading in turn to a reduction in efflux propensity and increased CNS disposition (Figure 1b).

The use of nonclassical bioisosteres in modern-day drug design to modify potency and key pharmacokinetic parameters has had a profound impact on how small-molecule lead optimization campaigns are approached.^26−28^ Underrepresented functional groups are a rich reservoir of nonclassical bioisosteres that enhance small-molecule compound collections by providing access to novel chemical space and conferring unique biological properties.^29−31^ Despite this, the uptake of an underrepresented functional group in medicinal chemistry is often thwarted by synthetic tractability or by its designation as a “structural alert”.^32^ Such alerts are a feature of all high-throughput screening campaigns and refer to functional groups that are excluded in the earliest stages of the development process to minimize false positives and inherent toxicities.^32,33^ While it is true that exclusion of promiscuous functional groups and adherence to established drug-like properties in drug discovery programs has reduced clinical attrition rates, uncritical application of structural alert “rules” across broad functional group classes can preclude the development of structurally innovative lead candidates residing in novel chemical space.^34^

The presence of a heteroatom-heteroatom bond is one such structural alert, of which the hydroxylamine N–O bond, with its bond dissociation energy (BDE) of 55–65 kcal·mol^–1^ and reputation for inherent mutagenicity and genotoxicity, is a pertinent example.^35−37^ This broad moratorium typically excludes trisubstituted hydroxylamines from small-molecule optimization schemes even though they should not harbor the same mutagenic potential as lesser substituted hydroxylamines.^38,39^ This is because the mutagenicity of less substituted hydroxylamines arises from sulfation or acetylation by cytosolic acetyltransferases and sulfotransferases and subsequent elimination to reactive nitroso compounds.^38,39^ The related hydroxamates undergo Lossen rearrangement to form reactive isocyanates that can subsequently act as electrophiles for DNA.^40,41^ In contrast, trisubstituted hydroxylamines are not susceptible to these modes of activation and their consequent toxicity; nevertheless, they are routinely excluded from compound optimization schemes. To our knowledge, there is only one actual small-molecule pharmaceutical containing the trisubstituted hydroxylamine moiety: the tetracycline, Sarecycline.^42^ To access novel chemical space accessible to trisubstituted hydroxylamines, we have developed synthetic strategies for their assembly by direct N–O bond formation.^43−47^ Using this method, we conducted a matched molecular pair (MMP) analysis^48^ to assess the early absorption, distribution, metabolism, and excretion (ADME) parameters of trisubstituted hydroxylamines and found them to exhibit unique properties that include similarities in lipophilicity to morpholine units.^47^ This suggested that trisubstituted hydroxylamines could serve as a novel bioisosteric modification of nitrogen heterocycles, which have received little attention in bioisosteric design compared to carbocycles,^49−52^ but which are present in roughly 60% of unique small-molecule drugs.^53^ Herein, we report on the success of this novel approach to bioisosterism and describe our efforts that culminated in the discovery of compound **9**: a promising lead for the treatment of CNS metastases in EGFR+ NSCLC that bears an atypical amine bioisostere, the trisubstituted hydroxylamine, and that lacks the mutagenicity and genotoxicity commonly surmised to accompany these units.

## Results and Discussion

**Figure 2:**
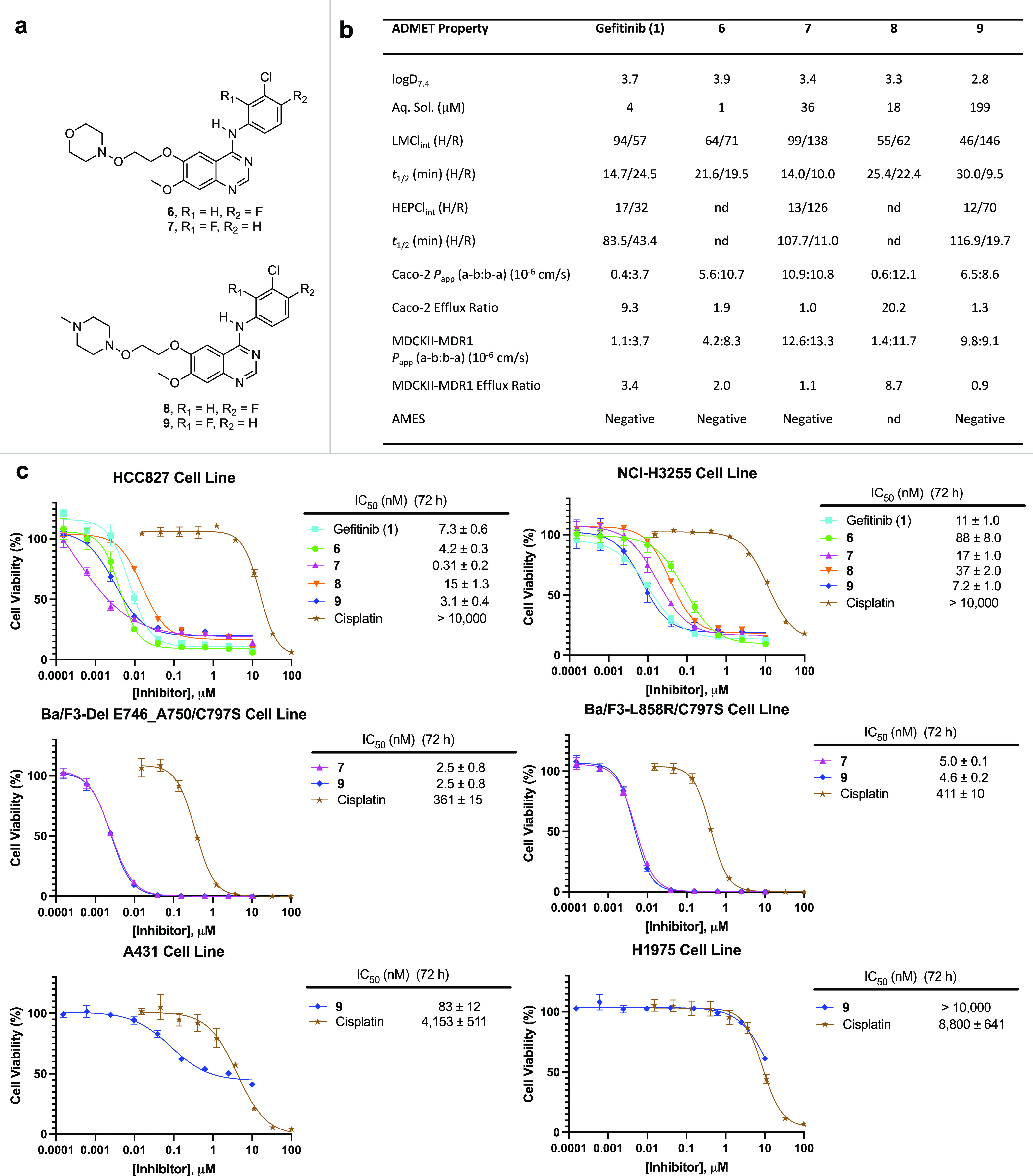
In vitro antiproliferative activity and early absorption, distribution, metabolism, excretion, and toxicity (ADMET) profile of the inhibitors. (A) Chemical structures of the hydroxylamine-bearing EGFR inhibitors used in this study. (B) *In vitro* ADMET properties of gefitinib and the hydroxylamine-based EGFR inhibitors. Values represent the mean of *n* = 2 independent replicates unless otherwise stated. Negative AMES results on **9** valid up to 50 μM, after which bacterial cytotoxicity was observed. Aq. Sol., aqueous solubility; LMCl_int_, intrinsic clearance in liver microsomes; HEPCl_int_, intrinsic clearance in hepatocytes; *t*_1/2_, half-life; *P*_app_, apparent permeability; MDCK, Madine-Darby-canine kidney; MDR1, multidrug resistance 1 (or P-glycoprotein). Abbreviations: H, human; R, rat; and nd, not determined. (C) **9** displays potent antiproliferative activity in patient-derived non-small-cell lung cancer cell lines HCC827 (*EGFR* mutation = exon19del), NCI-H3255 (*EGFR* mutation = L858R), and osimertinib-resistant engineered cell lines (Ba/F3-L858R/C797S; Ba/F3 del E746_A750/C797S) while displaying no activity in NCI-H1975 (*EGFR* mutation = L858R/T790M) and minimal activity in A431 cell line (overexpressed EGFR^wt^) (72 h dosing period). For all antiproliferative assays, points indicate mean and error bars indicate standard deviation (SD); *n* = 3 independent replicates; IC_50_ values (nM) are reported beside the dose–response curves and represent mean ± standard error of the mean (SEM). IC_50_ values (nM) are unadjusted for fetal bovine serum (FBS).

We began by evaluating inhibitor-binding constants and biochemical inhibition of the relevant EGFR kinase forms of **1** and a direct hydroxylamine analogue **6**; both **1** and **6** exhibited single-digit nanomolar activity against activating mutant bearing EGFR, indicating that an *N*-(noralkoxy)morpholine unit is indeed an effective bioisostere of the *N*-alkylmorpholine unit (Tables S1 and S3). We then assessed *in vitro* ADME properties, beginning with a colon carcinoma (Caco-2) cell permeability assay, which expresses both P-gp and BCRP; **6** displayed a 14-fold enhancement in permeability and greatly decreased efflux compared with **1** (Figure 2b). Substantiating this effect, **6** also showed a roughly 4-fold increase in permeability and decreased efflux compared to **1** in a Madin-Darby canine kidney (MDCK) MDCKII-MDR1 cell permeability assay that is commonly used to mimic the BBB through overexpression of the active efflux transporter P-gp, or MDR1. These results indicated that replacement of the *N*-alkylmorpholine unit by an *N*-(noralkoxy)morpholine unit might overcome the known^54^ poor BBB permeability and active efflux observed with **1**. Of note, currently osimertinib (**5**), a third-generation EGFR TKI, is the only approved EGFR TKI that shows promise in treating BM in EGFR+ NSCLC despite it being a substrate for both P-gp and BCRP.^18^ We then performed an AMES fluctuation assay both with and without metabolic activation by rat liver S9 (± S9) across 4 *Salmonella* strains (TA98, TA100, TA1537, and TA1535) and found that neither **1** or **6** were mutagenic.^55^ Taken in combination with a similar stability profile across multiple species in liver microsomes and plasma as compared to **1**, these findings dispelled any early concerns regarding perceived mutagenicity and instability toward oxidative metabolism of the trisubstituted hydroxylamine unit in **6** (see the Supporting Information for details).

**Figure 3:**
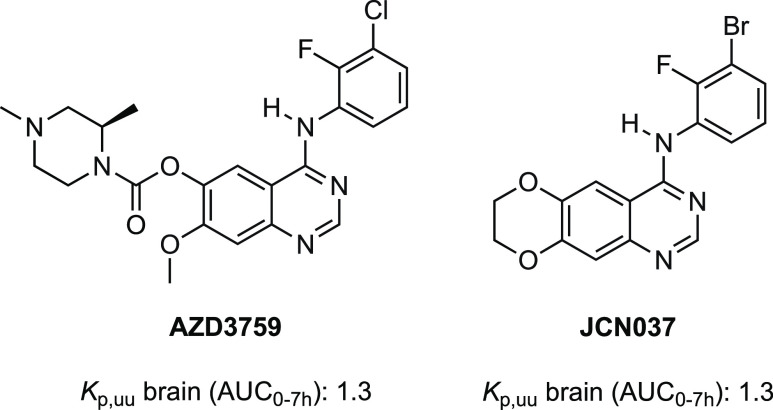
Chemical structures of investigational EGFR inhibitors AZD3759 and JCN037. *K*_p,uu_ brain (AUC_0–7h_) values refer to the unbound brain-to-unbound plasma partitioning coefficient.

**Figure 4:**
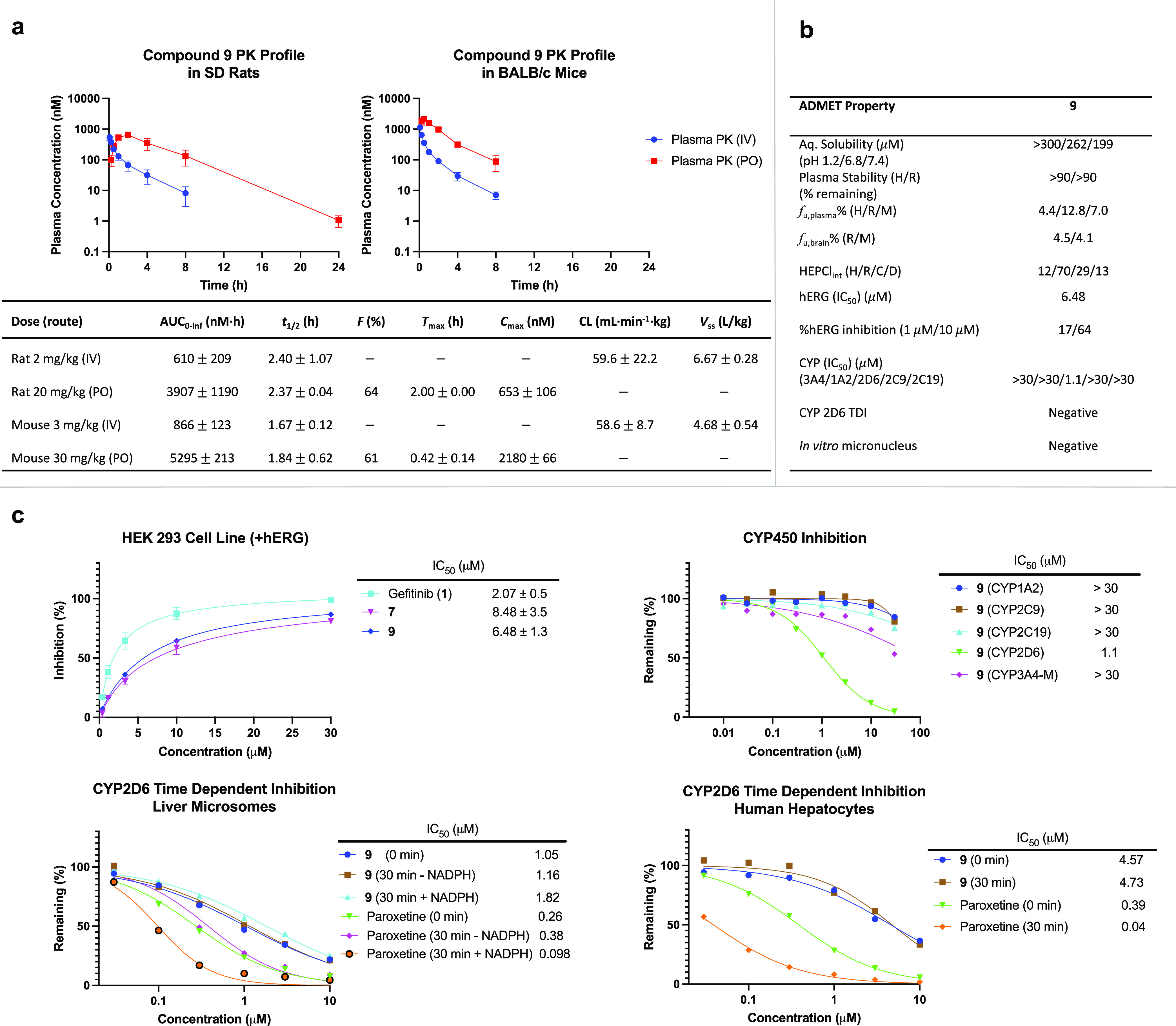
Pharmacokinetic profile of **9** and additional *in vitro* ADMET parameters. (A) Total plasma vs time profile (0–24 h) of **9** after administration into Sprague-Dawley rats at a single dose of 2 mg/kg IV and 20 mg/kg PO and BALB/c nude mice at 3 mg/kg IV and 30 mg/kg PO. AUC_0-inf_ (nM·h), area under concentration–time curve from 0 to ∞; *t*_1/2_ (h), mean elimination half-life obtained from either intravenous infusion (IV) or oral gavage (PO); *F*(%), bioavailability (%); *T*_max_ (h), time to reach peak plasma concentration; *C*_max_ (nM), peak plasma concentration; CL (mL·min^–1^·kg), clearance obtained from intravenous infusion. For pharmacokinetic profiles, points indicate mean and error bars indicate SD; *n* = 3 animals per route (*n* = 6 total). Values represent mean ± SD. (B) Additional *in vitro* ADMET profile of **9**. Values represent the mean of *n* = ≥2 independent replicates. *In vitro* micronucleus negative test results valid to 31 μM + rat liver S9 and 8 μM − rat liver S9, after which cytotoxicity was observed. Aq. Sol., aqueous solubility; *f*_u,plasma_%, percent fraction unbound in plasma; *f*_u,brain_%, percent fraction unbound in brain; HEPCl_int_, intrinsic clearance in hepatocytes. Abbreviations: H, human; R, rat; C, cynomolgus monkey; and D, dog. (C) **9** has low hERG inhibitory potential; points indicate mean, and error bars indicate the SD; *n* = 3 independent replicates; IC_50_ values (μM) are reported beside the dose–response curves and represent mean ± SEM. Low DDI is predicted with only minimal inhibition of CYP2D6 observed. Points indicate mean; *n* = 2 independent replicates. IC_50_ values (μM) are reported beside the dose–response curves and represent the mean.

We sought to further minimize efflux and improve drug-like properties, particularly the aqueous solubility of **6**, by synthesizing additional trisubstituted hydroxylamine-bearing inhibitors (**7**–**9**) (*vide infra*). In view of the importance of hydrogen bond donors (HBD) on efflux transporter substrate recognition, and noting the improved CNS penetrability of EGFR inhibitors AZD3759 and JCN037, **7** was designed with a *para-* to *ortho*-fluorine switch to minimize the HBD capability of the adjacent aniline (Figures 2a and 3).^56−59^ Additionally, **8** and **9** were prepared by exchange of the morpholine unit with an *N*-methylpiperazine group, all while maintaining the key trisubstituted hydroxylamine moiety, with the intent of improving aqueous solubility and potential *in vivo* exposure as borne out by subsequent pharmacokinetic (PK) measurements (*vide infra*) (Figure 2a). All newly prepared analogues maintained single-digit to subnanomolar binding and biochemical inhibition of activating mutant bearing EGFR (Tables S1 and S3). In the Caco-2 cellular assay, **7** and **9**, which bear the *ortho*-fluorine, exhibited remarkably enhanced permeability and reduced efflux ratios compared to **1** and even improved to that seen with **6** (Figure 2b). Notably, **8**, which contains a more basic nitrogen heterocycle than **1**, **6**, and **7** and a *para*-fluoro substitution pattern on the aniline ring, showed high efflux and low permeability, highlighting the synergistic improvement of modified HBD and reduced p*K*_a_ on efflux transporter substrate recognition. In the MDCKII-MDR1 cellular assays, **7** and **9** showed excellent permeability and low efflux as compared to **1**, **8**, and even **6**. Importantly, **9** also displayed good stability in human and rat microsomes and hepatocytes while exhibiting markedly improved aqueous solubility compared to both **1** and **6** (Figure 4b).

We profiled the anticancer activity of the hydroxylamine-bearing inhibitors against four patient-derived cell lines harboring differing *EGFR* status with cisplatin as the positive control throughout (Figure 2c). Notably, **9** displayed excellent activity against the NCI-H3255 NSCLC cell line bearing the common EGFR^L858R^ mutation, with an IC_50_ of 7.2 nM representing a roughly 12-fold improvement over **6**. Compound **9** also had potent activity against HCC827 NSCLC cells harboring EGFR^del E746_A750^ with an IC_50_ of 3.1 nM. In osimertinib-resistant engineered Ba/F3 cell lines bearing EGFR^L858R/C797S^ and EGFR^del E746_A750/C797S^, which block covalent inhibitor binding by a C797S point mutation, like **1**,^60^**9** exhibited strong activity, with IC_50_ values of 4.6 and 2.5 nM, respectively.^12,13^ In the skin-derived A431 cell line bearing overexpressed EGFR^wt^**9** exhibited a comparatively high IC_50_ of 83 nM, affording 12- to 33-fold selectivity for activating mutant EGFR over wild-type EGFR. Overall, **9** is a potent inhibitor in osimertinib-resistant engineered cell lines and patient-derived NSCLC cell lines NCI-H3255 and HCC827, which harbor mutations in *EGFR* that encompass approximately 85% of all newly diagnosed mutant EGFR+ NSCLC cases.

Moving forward with **9**, we determined the unbound fractions in plasma and brain tissue (Figure 4b).^20^ With regard to plasma protein binding, **9** exhibited good unbound fractions in plasma and excellent unbound rodent brain tissue fractions (*f*_u,brain_% = 4.5 and 4.1) (Table S8). With respect to potential toxicity, only moderate human Ether-à-go-go-Related Gene (hERG) potassium ion channel inhibition by **9** was observed with an IC_50_ of 6.48 μM and a maximal inhibition of approximately 60% at 10 μM (Figure 4c). We also assayed for CYP inhibition across all major isoforms (3A4, 1A2, 2C9, 2D6, 2C19) and saw none (IC_50_ = >30 μM) except for moderate inhibition of CYP2D6 (IC_50_ = 1.1 μM) (Figure 4c). In a follow-up CYP2D6 time-dependent inhibition (TDI) IC_50_-shift experiment in human liver microsomes and primary human hepatocytes, this latter activity was shown not to be time-dependent, thus dispelling concerns of potential drug–drug interactions (DDI) (Figure 4c).^61^ In the AMES fluctuation assay across 4 *Salmonella* strains (TA98, TA100, TA1537, and TA1535), and an *in vitro* micronucleus test in Chinese hamster ovary (CHO-K1) cells both with and without metabolic activation by rat liver S9 (± S9), **9** exhibited neither mutagenic nor genotoxic potential, contrary to the popular belief that hydroxylamines are inherently mutagenic and genotoxic (Tables S33 and S35).^34,35,55,62^ Finally, to dispel concerns regarding the perceived metabolic instability of the N–O bond, we conducted an *in vitro* metabolite identification (MetID) study in both human and rat hepatocytes after 4 h incubation. Gratifyingly, the metabolism of **9** closely followed that of **1**,^63^ with N–O bond cleavage only contributing to minor metabolites identified (<5%) (Figures S1–S2). Overall, these experiments indicate that **9** is a potent inhibitor of activating mutant bearing EGFR but exhibits much reduced efflux compared to those of most currently approved targeted EGFR therapies.

**Figure 5:**
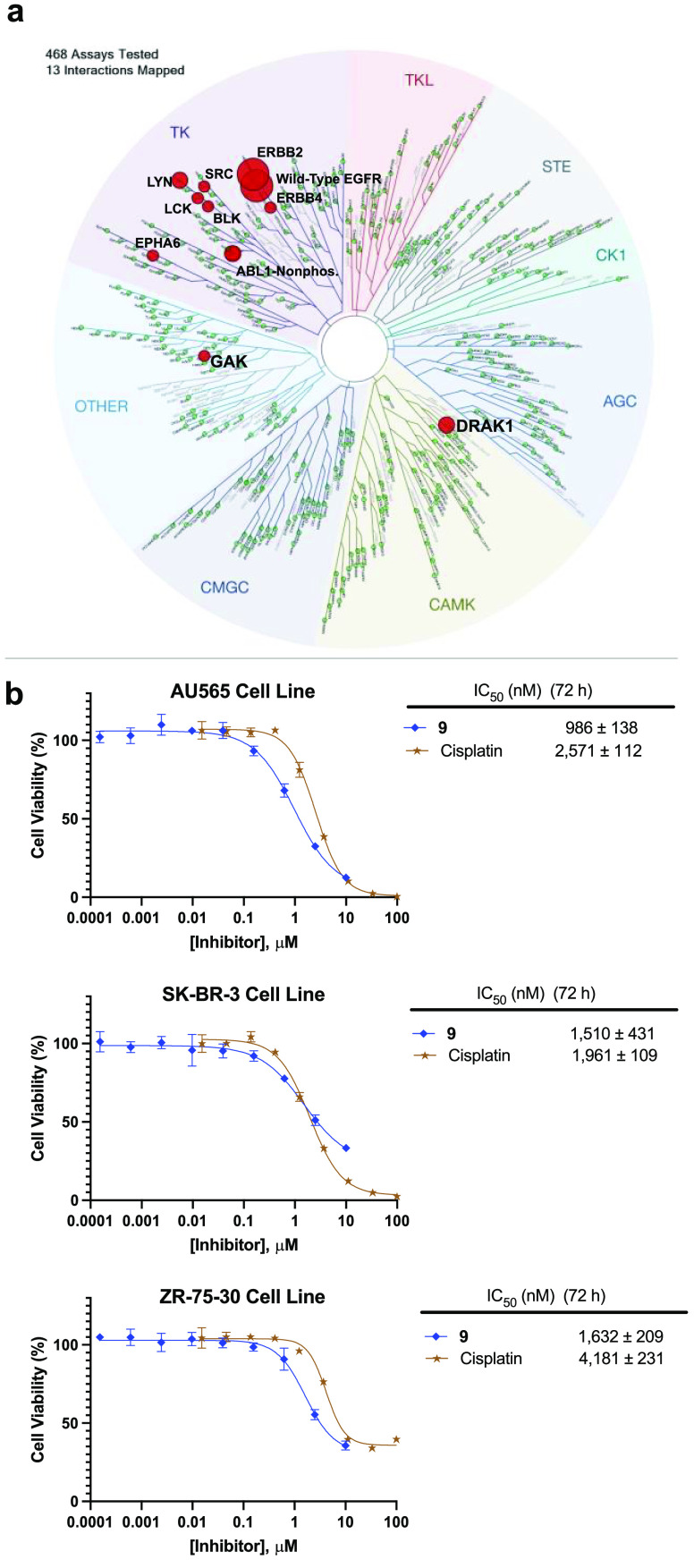
Kinase selectivity profile for **9** and extended *in vitro* assays. (A) KINOMEScan nonmutant or lipid kinase screening results of **9** at a screening concentration of 1 μM. The size of circles mapped onto the kinase phylogenetic tree using DiscoverX TREEspot corresponds to strength of binding affinity. Full KINOMEScan results are listed in the Supporting Information. (B) **9** displays moderate antiproliferative activity in patient-derived *HER2*-positive breast cancer cell lines: AU565, SK-BR-3, and ZR-75–30. For all antiproliferative assays, points indicate mean and error bars indicate SD; *n* = 3 independent replicates; IC_50_ values (nM) are reported beside the dose–response curve and represent mean ± SEM. IC_50_ values (nM) are unadjusted for FBS.

Using KINOMEScan technology, we determined the selectivity of **9** against a panel of >400 human kinases at a concentration of 1 μM; exquisite kinase selectivity was found with an S(10) score of 0.015 (6/403 nonmutant kinases showing ≤10% activity at 1 μM) (Figure 5a; Table S38).^64^ Apart from EGFR^wt^ (0.30%), **9** only showed high affinity for ERBB2 (HER2) (0.45%). Moderate affinity for ABL1 (7.7%), DRAK1 (8.3%), LYN (7.4%), and PIKFYVE (1.6%) was also observed but confirmed in a follow-up binding experiment to be of minor significance considering the potent binding to EGFR (*K*_d_ = <0.2 nM) (Tables S1 and S3). As a result of the moderate affinity for HER2 that was confirmed in a follow-up binding experiment (*K*_d_ = 21 nM) and considering the lack of efficient treatments for metastatic HER2+ breast cancer,^65^ we further profiled **9** against HER2 using additional biochemical and cellular assays (Tables S2 and S4; Figure 5b). We found that **9** exhibited low antiproliferative activity in HER2+ breast cancer cell lines in comparison to the activating mutant bearing EGFR+ cells, with IC_50_’s ranging from 0.99 to 1.6 μM.

**Figure 6:**
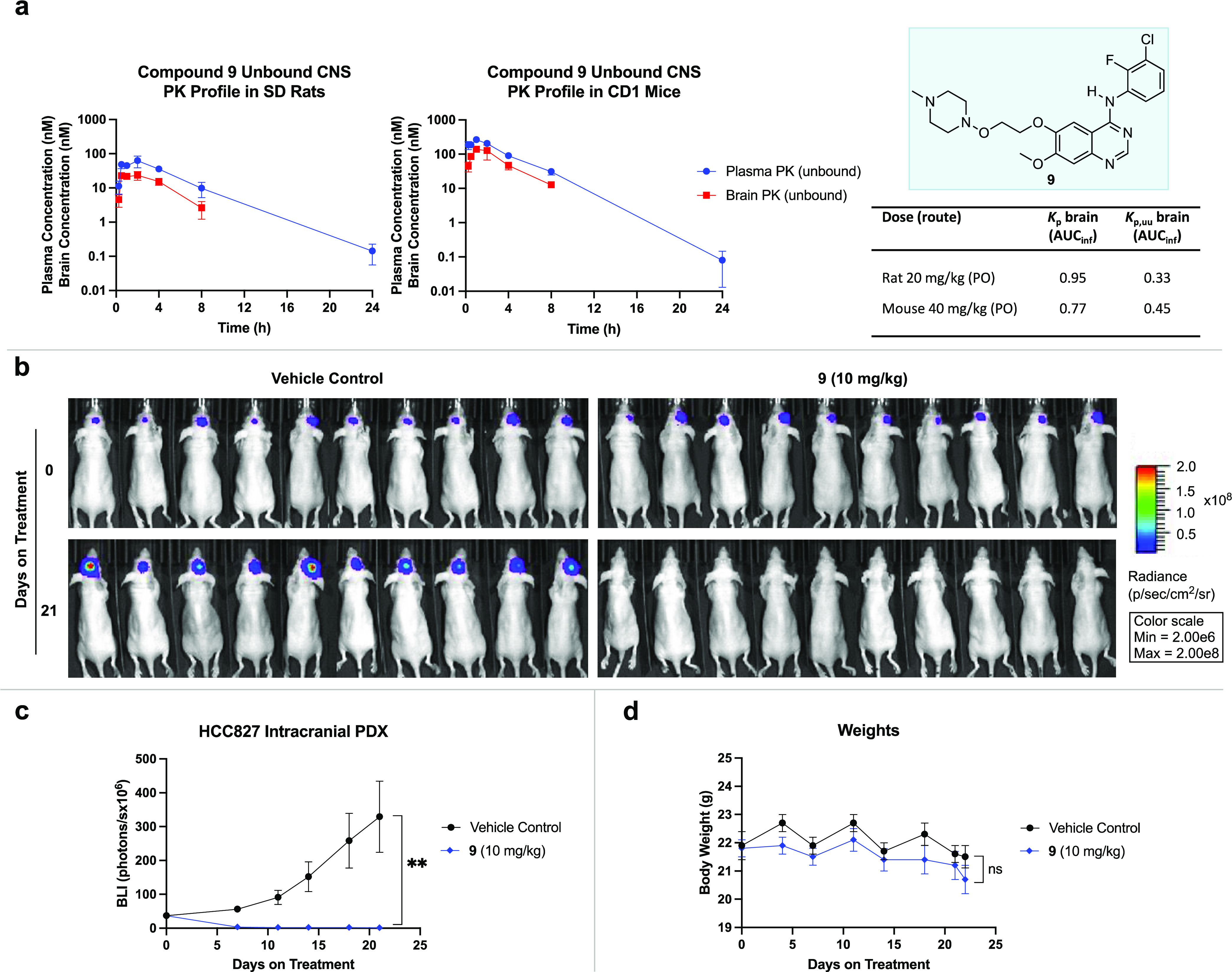
Central nervous system pharmacokinetic profile and intracranial efficacy assessment of **9**. (A) Unbound plasma and brain concentration vs time profile (0–24 h) of **9** after administration into Sprague-Dawley rats at a single dose of 20 mg/kg and CD1 mice at 40 mg/kg by oral gavage (PO) shows excellent unbound brain penetration. Unbound concentrations were calculated by multiplying total concentrations at a given time point by the unbound fraction values in plasma or brain. *K*_p_ brain (AUC_inf_) refers to the brain-to-plasma partitioning coefficient. *K*_p,uu_ brain (AUC_inf_) refers to the unbound brain-to-unbound plasma partitioning coefficient. For pharmacokinetic profiles, points indicate mean and error bars indicate SD; *n* = 3 animals at each time point (*n* = 21 total). (B) Bioluminescence images of vehicle control and **9** (10 mg/kg PO b.i.d.) dosing groups indicating a change in tumor volume over 21 days of treatment. (C) Intracranial bioluminescence over time. Points indicate mean, and error bars indicate the SEM; *P* = 0.0059; *n* = 10 mice per group. PDX, patient-derived xenograft. *p* value was obtained from an unpaired two-tailed *t*-test comparing the means of vehicle control and **9** (10 mg/kg PO b.i.d.) study arms after 21 days of treatment. *P < 0.05; **P < 0.01. (D) Mean body weight of mice during the efficacy study. Points indicate mean, and error bars indicate the SEM; *P* = 0.2275; *n* = 10 mice per group. *P* value was obtained from an unpaired two-tailed *t*-test comparing the means of vehicle control and **9** (10 mg/kg PO b.i.d.) study arms after 21 days of treatment. ns, not significant.

To qualify **9** as a candidate for further studies, we performed a head-to-head pharmacokinetic study with **7** by administration into Sprague–Dawley rats (Figures S3–S4). No adverse events or signs of toxicity were observed for the compound at the tested doses, contrary to the common belief that hydroxylamines are inherently toxic.^34,35^ As we had hypothesized (*vide supra*), **9** exhibited excellent exposure (AUC_inf_ = 279 h·ng/mL) and half-life (*t*_1/2_ = 2.29 h) compared to **7** (AUC_inf_ = 89 h·ng/mL) (*t*_1/2_ = 0.56 h) at 1 mg/kg intravenous (IV) and 5 mg/kg oral (PO) dosing, that we attribute in part to the increased solubility wrought by the piperazine for morpholine exchange (Figure 2a; Figures S3–S4). To further demonstrate the excellent pharmacokinetic properties of **9** we increased the dose to 2 mg/kg IV and 20 mg/kg PO dosing in Sprague-Dawley rats and saw excellent oral bioavailability (*F* = 64%), exposure (AUC_inf_ = 3907 h·ng/mL) and an acceptable half-life (*t*_1/2_ = 2.37 h) with a high volume of distribution (*V*_*ss*_ = 6.67 L/kg) (Figure 4a). Furthermore, after administration of a single oral dose of 20 mg/kg in Sprague-Dawley rats, **9** exhibited a *K*_p,uu_ (unbound brain-to-unbound plasma partitioning coefficient) (AUC_inf_) of 0.33 and a *K*_p_ (brain-to-plasma partitioning coefficient) (AUC_inf_) of 0.95, indicating excellent brain penetration (Figure 6a).^20,66^ Substantiating this effect, after administration of a single oral dose of 40 mg/kg in CD1 mice, a *K*_p,uu_ (AUC_inf_) of 0.45 and a *K*_p_ (AUC_inf_) of 0.77 was obtained (Figure 6a). This stands in contrast, to **1** which is reported^18^ to have a *K*_p,uu_ (AUC_0−16h_) of 0.0092 after administration of a single oral dose of 10 mg/kg in Han Wistar rats, and underlines the potential of **9** in the treatment of CNS metastatic NSCLC.

Finally, to assess the viability of **9** as a potential treatment for BM in EGFR+ NSCLC, we performed an intracranial patient-derived xenograft (PDX) model with luciferase-tagged HCC827 cells implanted into the brains of BALB/c nude mice (Figure 6b). At 10 mg/kg PO b.i.d. (twice daily)^67^ dosing, profound tumor regression (*P* = 0.0059) relative to vehicle control (1% methylcellulose) was observed, confirming that **9** has intracranial antitumor activity (Figure 6c). Additionally, over the 21-day treatment window, mean body weight loss never exceeded 10% and no adverse clinical events were observed at the studied dose, thereby demonstrating the potential of **9** as a candidate for CNS metastatic EGFR+ NSCLC (Figure 6d).

## Chemistry

**Figure 7:**
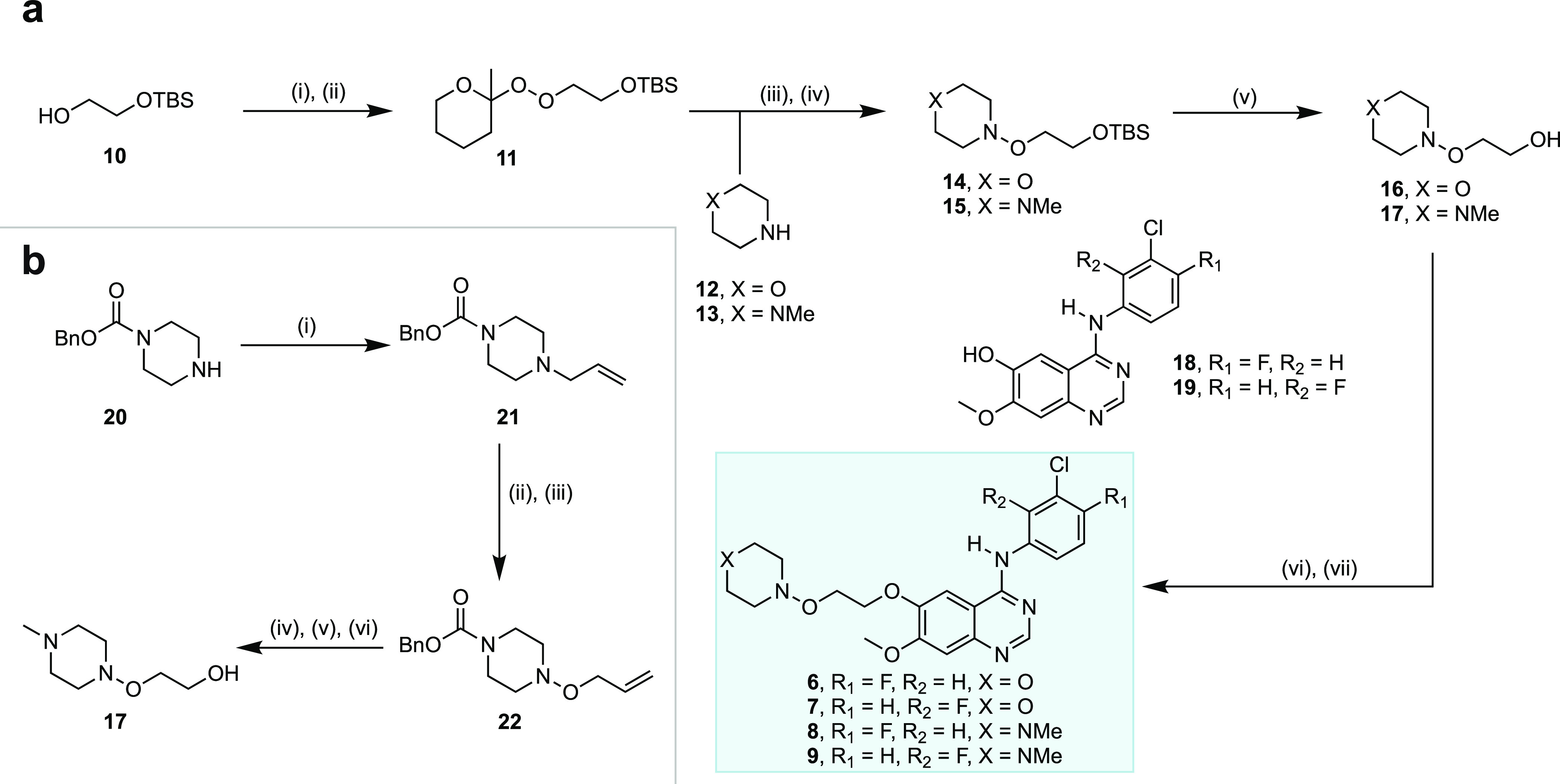
Chemical synthesis of hydroxylamine-bearing EGFR inhibitors and precursors. (A) Chemical synthesis of inhibitors **6**–**9**. Reagents and conditions: (i) Triflic anhydride (Tf_2_O), pyridine, dichloromethane (DCM), 0 °C, 45 min; (ii) 2-methyltetrahydropyranyl hydroperoxide (MTHPOOH), potassium *tert*-butoxide (KO^*t*^Bu), tetrahydrofuran (THF), 0 °C to r.t., 90 min; (iii) **12** (morpholine) or **13** (*N*-methylpiperazine), ^*i*^PrMgCl·LiCl (isopropylmagnesium chloride lithium chloride), THF, 0 °C to r.t., 45 min; (iv) **11**, THF, r.t., 3 h; (v) tetra-*N*-butylammonium fluoride (TBAF), THF, 0 °C to r.t., 1−1.5 h; (vi) thionyl chloride (SOCl_2_), toluene, r.t. to 60 °C, 3 h; (vii) **18** or **19**, sodium hydride (NaH, 60% dispersion in mineral oil), THF, 0–80 °C, 16 h. (B) Alternative scaled synthesis of the key hydroxylamine precursor (**17**). Reagents and conditions: (i) Allyl bromide, potassium carbonate (K_2_CO_3_), THF, r.t. to 65 °C, 16 h; (ii) *meta*-chloroperoxybenzoic acid (MCPBA), DCM, −30 °C, 1 h; (iii) toluene, 80 °C, 14 h; (iv) O_3_, DCM/MeOH, −78 °C, 30 min; (v) sodium borohyride (NaBH_4_), DCM/MeOH, −78 °C to r.t., 2 h; (vi) lithium aluminum hydride (LiAlH_4_), THF, 0 °C to r.t., 2 h.

The synthesis of all inhibitors commenced with a modified version of our N–O bond-forming reaction as the key step (Figure 7a).^43,68^ To this end, the 2-methyltetrahydropyranyl (MTHP) monoperoxyacetal^69−71^ (**11**) derived from commercially available 2-((*tert*-butyldimethylsilyl)oxy)ethanol (**10**) was exposed to a morpholine (**12**) or *N*-methylpiperazine (**13**) derived magnesium amide affording the hydroxylamines (**14**–**15**) in 65–67% yields, respectively, on multigram scales. Notably, we discovered that the use of Knochel’s^68^ turbo-Grignard (^*i*^PrMgCl·LiCl) as base proved optimal for N–O bond formation on a larger scale (Figure S6; see the Supporting Information for details). Deprotection of the *tert*-butyldimethylsilyl groups in **14** and **15** afforded alcohols (**16** and **17**) in 71–79% yields, that on conversion to the chloride by reaction with thionyl chloride and subsequent displacement with commercially available phenols (**18**–**19**) under basic conditions, afforded inhibitors **6**–**9** in 20–35% yields over 2 steps. A crystal structure was also obtained to support the structure of the direct hydroxylamine analogue derivative (**6**) (see the Supporting Information for details).

While we have encountered no issues with our direct N–O bond-forming reaction^43^ up to the decagram scale, an alternative scalable approach to the key *N*-methylpiperazine-derived hydroxylamine precursor (**17**) was also developed (Figure 7b). Thus, the allylation of commercially available **20** under basic conditions gave the *N*-allyl derivative (**21**) in 72% yield. Multigram scale quantities of **22** were then obtained in 47% yield by *N*-oxidation of **21** with *meta*-chloroperoxybenzoic acid (MCPBA) followed by [2,3]-Meisenheimer rearrangement.^72,73^ Compound **22** was then subjected to ozonolysis followed by reductive workup with sodium borohydride (NaBH_4_), after which, the carboxybenzyl group was reduced with lithium aluminum hydride^74^ (LiAlH_4_) to give the intended hydroxylamine precursor **17** in 50% yield over 3 steps. This alternative route to **17** proceeds in 17% overall yield and the longest linear sequence of only 3 steps from commercially available benzyl 1-piperazinecarboxylate (**20**).

## Conclusions

A highly selective, orally bioavailable, brain-penetrant EGFR inhibitor, **9**, bearing a novel amine bioisostere, a trisubstituted hydroxylamine, has been reported. In contrast to the common expectation for hydroxylamines in medicinal chemistry, **9** lacks mutagenic or genotoxic potential and exhibits good stability *in vitro* and *in vivo*. An intracranial PDX murine model revealed profound tumor regression on oral dosing with **9** suggesting this novel compound as a potential lead in the treatment of localized and CNS metastatic NSCLC driven by activating mutant bearing EGFR and for osimertinib-resistant EGFR+ NSCLC bearing the C797S resistance mutation profile (L858R/C797S and exon19del/C797S). All told, our results show that the trisubstituted hydroxylamine moiety, a perceived “structural alert” in medicinal chemistry, can be incorporated into drug scaffolds to improve drug properties while maintaining potent biological activity and avoiding molecular weight creep. These findings support the broader application of trisubstituted hydroxylamines as bioisosteres in drug discovery programs for lead optimization and in patent life-cycle management.

## Experimental Section

### Chemistry

#### General Experiment and Information

All reactions were conducted in single-neck oven-dried glassware fitted with a rubber septum under an argon atmosphere, unless otherwise stated. All organic solutions were concentrated under reduced pressure on a rotary evaporator and a water bath. Flash column chromatography was performed using silica gel (Fischer Silica Gel Sorbent (230–400 Mesh, grade 60)).^75^ Thin-layer chromatography (TLC) was carried out with 250 μM glass back silica (XHL) plates with a fluorescent indicator (254 nm). TLC plates were visualized by exposure to ultraviolet light (UV) and/or submersion in ceric ammonium molybdate (CAM) in ethanol, followed by heating on a hot plate (120 °C, 10–15 s). Solvents were purchased from Sigma-Aldrich and used without further purification. Starting material 2-hydroperoxy-2-methyltetrahydro-2*H*-pyran (MTHPOOH) was prepared according to literature procedures^43,69,70^ and spectral data were in accord with that previously reported. 2-(*tert*-Butyldimethylsilyloxy)-ethan-1-ol was purchased from AK Scientific. Morpholine was purchased from Sigma-Aldrich. *N*-Methylpiperazine was purchased from Sigma-Aldrich. Isopropylmagnesium chloride lithium chloride complex solution (1.3 M in THF) was purchased from Sigma-Aldrich. Ethylmagnesium bromide (3 M in THF) was purchased from Sigma-Aldrich. Tetrabutylammonium fluoride (1 M in THF) was purchased from TCI. Thionyl chloride was purchased from Alfa Aesar. Sodium Hydride (60% dispersion in mineral oil) was purchased from Sigma-Aldrich. Pyridine was purchased from OakWood Chemicals. 4-(3-Chloro-4-fluorophenylamino)-7-methoxyquinazolin-6-ol was purchased from AK Scientific. 4-(3-Chloro-2-fluorophenylamino)-7-methoxyquinazolin-6-ol was purchased from AmBeed. Gefitinib (**1**) (≥98% (HPLC), SML1657–50MG) was purchased from Sigma-Aldrich for *in vitro* assays. Nuclear magnetic resonance (NMR) spectra of all compounds were obtained in either CDCl_3_ (δ_H_ 7.26 and δ_C_ 77.16 ppm, respectively) or C_6_D_6_ (δ_H_ 7.16 and δ_C_ 128.06 ppm, respectively), Toluene-D_8_ (δ_H_ 7.09, 6.98, 7.00, 2.09 and δ_C_ 137.86, 129.24, 128.33, 125.49, 20.40 ppm, respectively), or DMSO–D_6_ (δ_H_ 2.50 and δ_C_ 39.52 ppm, respectively) using a 500 MHz, EZC500 JEOL instrument at 298 K unless otherwise specified. The chemical shifts (δ) are calculated with respect to residual solvent peak and are given in ppm. Multiplicities are abbreviated as follows: s (singlet), m (multiplet), b (broad), d (doublet), t (triplet), q (quartet), and hept (heptet). High-resolution mass spectra were obtained on a ThermoFisher Orbitrap Q-Exactive instrument using electrospray ionization (ESI). Melting point of solids **6**, **7**, **8**, and **9** were determined on a Barstead Electrothermal 9100. Ultra high-performance liquid chromatography (UHPLC) traces of compounds **6**, **7**, **8**, and **9** were obtained using a ThermoFisher Vanguish UHPLC with PDA detector and an Acclaim 120 ^18^C 4.6 mm × 50 mm column, and the %purity was determined using the Avalon peak area algorithm. Purities of all final compounds were confirmed to be >95% by UHPLC. UHPLC conditions for **6**, **7**, **8**, and **9** are given in the catalogue of spectra section on pages S109–S113 in the Supporting Information. The purity of commercial gefitinib (**1**) was confirmed by NMR and high-resolution mass spectrometry (HRMS) prior to *in vitro* study initiation.

##### *tert*-Butyldimethyl(2-((2-methyltetrahydro-2*H*-pyran-2-yl)peroxy)ethoxy)silane (**11**)

To a stirred solution of 2-(*tert*-butyldimethylsilyloxy)-ethan-1-ol (**10**) (13.33 g, 75.71 mmol, 1.0 equiv) in anhydrous DCM (250 mL) at 0 °C was added pyridine (9.66 mL, 113.57 mmol, 1.5 equiv) followed by dropwise addition of Tf_2_O (15.26 mL, 90.85 mmol, 1.2 equiv), and the solution was stirred for 45 min at 0 °C. After such time, the mixture was diluted with DCM (100 mL) and washed successively with 1 N HCl (1×, 150 mL), aq. NaHCO_3_ (1×, 150 mL), and brine (1×, 150 mL). The combined organic layers were dried over Na_2_SO_4_, filtered, and concentrated *in vacuo*. The crude triflate was used directly in the next step without further purification. To a stirred solution of MTHPOOH^43,69,70^ (12 g, 90.85 mmol, 1.2 equiv) in anhydrous THF (260 mL) at 0 °C was added KO*^t^*Bu (10.19 g, 90.85 mmol, 1.2 equiv), and the solution was stirred for 30 min, after which the crude triflate obtained in the previous step was added dropwise in anhydrous THF (40 mL). The solution was warmed to rt and stirred for 90 min. After such time, the mixture was diluted with EtOAc (100 mL) and quenched via the addition of aq. NaHCO_3_ (1×, 200 mL). The layers were separated, and the aqueous layer was extracted with EtOAc (2×, 100 mL). The combined organic layers were washed with brine (1×, 100 mL), dried over Na_2_SO_4_, filtered and concentrated *in vacuo*. The residue obtained was purified by flash column chromatography on silica (eluent: 10:90 EtOAc/Hexanes) to afford the title compound **11** (11.19 g, 38.56 mmol, 51%) as an off-yellow oil. Spectral data are in accord with that previously reported in the literature.^47^ TLC *R_f_* = 0.50 (20:80 EtOAc/Hexanes; CAM). ^1^H NMR (500 MHz, CDCl_3_) δ 4.13–4.07 (m, 2H), 3.92 (td, *J =* 11.4, 2.8 Hz, 1H), 3.85 (t, *J =* 5.4 Hz, 2H), 3.71–3.68 (m, 1H), 1.79–1.50 (m, 6H), 1.43 (s, 3H), 0.90 (s, 9H), 0.08 (s, 6H). ^13^C NMR (126 MHz, CDCl_3_) δ: 102.5, 76.7, 61.8, 60.8, 33.3, 26.0, 24.9, 24.6, 19.2, 18.5, −5.2. **CAUTION**: While we have not encountered any decomposition, standard caution should be exercised when working with peroxides (i.e., avoid exposure to light, reducing agents, excessive heat, and work behind a protective shield).

##### 4-(2-((*tert*-Butyldimethylsilyl)oxy)ethoxy)morpholine (**14**)

To a stirred solution of morpholine (**12**) (7.64 mL, 87.33 mmol, 3.0 equiv) in anhydrous THF (73 mL) at 0 °C was added *^i^*PrMgCl·LiCl (1.3 M in THF) (56 mL, 72.78 mmol, 2.5 equiv) and the solution was brought to r.t. and stirred for 45 min. After such time, **11** (8.45 g, 29.11 mmol, 1.0 equiv) was added dropwise in anhydrous THF (73 mL) and the solution was stirred for 3 h. After this time, the solution was quenched with aq. NaHCO_3_ (100 mL). The layers were separated and the aqueous layer was extracted with EtOAc (3×, 75 mL). The combined organic layers were dried over Na_2_SO_4_, filtered, and concentrated *in vacuo*. The residue obtained was purified by flash column chromatography on silica (eluent: 15:85 EtOAc/Hexanes) to afford the title compound **14** (4.95 g, 18.95 mmol, 65%) as a light yellow oil. TLC *R_f_* = 0.20 (10:90 EtOAc/Hexanes; CAM). ^1^H NMR (500 MHz, CDCl_3_) δ 3.88 (d, *J =* 11.8 Hz, 2H), 3.79–3.72 (m, 4H), 3.62–3.54 (m, 2H), 3.15 (d, *J =* 10.6 Hz, 2H), 2.66 (td, *J =* 10.9, 3.3 Hz, 2H), 0.89 (s, 9H), 0.06 (s, 6H). ^13^C NMR (126 MHz, CDCl_3_) δ: 73.1, 66.4, 61.6, 56.4, 26.0, 18.5, −5.1. HRMS-ESI (*m*/*z*): [M + H]^+^ calculated for [C_12_H_28_O_3_NSi]^+^: 262.1833, found: 262.1835. See Figure S6 for the reaction setup.

##### 1-(2-((*tert*-Butyldimethylsilyl)oxy)ethoxy)-4-methylpiperazine (**15**)

To a stirred solution of *N*-methylpiperazine (**13**) (12.62 mL, 113.73 mmol, 3.0 equiv) in anhydrous THF (95 mL) at 0 °C was added *^i^*PrMgCl·LiCl (1.3 M in THF) (72.9 mL, 94.78 mmol, 2.5 equiv) and the solution was brought to r.t. and stirred for 45 min. After such time, **11** (11 g, 37.91 mmol, 1.0 equiv) was added dropwise in anhydrous THF (95 mL) and the solution was stirred for 3 h. After this time, the solution was quenched with aq. NaHCO_3_ (75 mL). The layers were separated and the aqueous layer was extracted with EtOAc (3×, 50 mL). The combined organic layers were dried over Na_2_SO_4_, filtered, and concentrated *in vacuo*. The residue obtained was purified by flash column chromatography on silica (eluent: 40:55:5 Hexanes/EtOAc/Et_3_N) to afford the title compound **15** (6.99 g, 25.5 mmol, 67%) as a yellow oil. TLC *R_f_* = 0.30 (40:55:5 Hexanes/EtOAc/Et_3_N; CAM). ^1^H NMR (500 MHz, C_6_D_6_) δ 3.83 (t, *J =* 5.2 Hz, 2H), 3.75 (t, *J =* 5.5 Hz, 2H), 3.15–3.13 (m, 2H), 2.84 (brs, 2H), 2.49–2.46 (m, 2H), 2.10–2.06 (m, 2H), 2.02 (s, 3H), 0.99 (s, 9H), 0.09 (s, 6H). ^13^C NMR (126 MHz, C_6_D_6_) δ: 73.2, 62.0, 55.9, 54.4, 45.6, 26.2, 18.6, −5.1. HRMS-ESI (*m*/*z*): [M + H]^+^ calculated for [C_13_H_31_O_2_N_2_Si]^+^: 275.2149, found: 275.2146. See Figure S8 for the reaction setup.

##### 2-(Mopholinooxy)ethan-1-ol **(16**)

To a stirred solution of **14** (4.90 g, 18.80 mmol, 1.0 equiv) in anhydrous THF (150 mL) at 0 °C was added TBAF (1 M in THF) (37.60 mL, 37.60 mmol, 2.0 equiv) dropwise, and the solution was brought to r.t. and stirred for 1 h. After such a time, the mixture was concentrated *in vacuo* and the residue obtained was purified by flash column chromatography (eluent: 95:5 EtOAc/Et_3_N) to afford the title compound **16** (2.17 g, 14.75 mmol, 79%) as a yellow oil. TLC *R_f_* = 0.50 (95:5 EtOAc/Et_3_N; CAM). ^1^H NMR (500 MHz, CDCl_3_) δ 3.91 (d, *J =* 12.3 Hz, 2H), 3.86–3.79 (m, 4H), 3.56 (t, *J =* 12.6 Hz, 2H), 3.34 (br s, 1H), 3.21 (d, *J =* 11.9 Hz, 2H), 2.67 (td, *J =* 10.9, 3.3 Hz, 2H). ^13^ C NMR (126 MHz, CDCl_3_) δ: 71.6, 66.3, 63.6, 56.2. HRMS-ESI (*m*/*z*): [M + H]^+^ calculated for [C_6_H_14_O_3_N]^+^: 148.0968, found: 148.0964.

##### 2-((4-Methylpiperazin-1-yl)oxy)ethan-1-ol (**17**)

To a stirred solution of **15** (6.35 g, 23.16 mmol, 1.0 equiv) in anhydrous THF (300 mL) at 0 °C was added TBAF (1 M in THF) (46.32 mL, 46.32 mmol, 2.0 equiv) dropwise, and the solution was brought to rt and stirred for 90 min. After such time, the mixture was concentrated *in vacuo* and the residue obtained was purified by flash column chromatography (eluent: 90:10 EtOAc/Et_3_N) to afford the title compound **17** (2.18 g, 13.61 mmol, 71%) as a yellow oil. TLC *R_f_* = 0.10 (90:10 EtOAc/Et_3_N; CAM).^1^H NMR (500 MHz, Toluene-D_8_) δ 3.69–3.66 (m, 2H), 3.65–3.60 (m, 2H), 3.01 (br d, *J =* 10.3 Hz, 2H), 2.73–2.59 (m, 2H), 2.41 (br d, *J =* 11.3 Hz, 2H), 1.96 (s, 3H), 1.94–1.90 (m, 2H).^13^ C NMR (126 MHz, Toluene-D_8_) δ 72.2, 63.2, 55.4, 54.2, 45.3. HRMS-ESI (*m*/*z*): [M + H]^+^ calculated for [C_7_H_17_O_2_N_2_]^+^: 161.1285, found: 161.1282.

##### *N*-(3-Chloro-4-fluorophenyl)-7-methoxy-6-(2-(morpholinooxy)ethoxy)quinazolin-4-amine (**6**)

To a stirred solution of **16** (1 g, 6.80 mmol, 1.0 equiv) in anhydrous toluene (20 mL) at 0 °C was added SOCl_2_ (1.23 mL, 17.00 mmol, 2.5 equiv) dropwise. The reaction mixture was stirred until no longer exothermic and then brought to 60 °C and stirred for 3 h. After such time, the mixture was concentrated *in vacuo* and the resulting residue was used directly in the next step without further purification. To a stirred solution of **18** (3.26 g, 10.20 mmol, 1.5 equiv) in anhydrous *N*,*N*-dimethylformamide (DMF, 35 mL) at 0 °C was added NaH (60% dispersion in mineral oil) (406 mg, 10.20 mmol, 1.5 equiv) slowly, and the solution was warmed to r.t. and stirred for 45 min. Following this, the crude chloride obtained in the previous step was added dropwise in DMF (5 mL) and the solution was brought to 80 °C and stirred for 16 h. After such time the mixture was concentrated *in vacuo* and coconcentrated with toluene (3×, 50 mL). The resulting residue was then redissolved in EtOAc (200 mL) and washed successively with 1 M NaOH (2×, 100 mL), brine (2×, 100 mL), then the combined organic layers were dried over Na_2_SO_4_, filtered and concentrated *in vacuo*. The residue obtained was purified by flash column chromatography (eluent: 10:90 Et_3_N/EtOAc) to afford the title compound **6** (1.08 g, 2.41 mmol, 35% over 2 steps) as a light yellow powder. TLC *R_f_* = 0.50 (10:90 Et_3_N/EtOAc; UV, CAM). ^**1**^H NMR (500 MHz, DMSO–D_6_) δ 9.51 (s, 1H), 8.50 (s, 1H), 8.12 (dd, *J =* 6.8, 2.7 Hz, 1H), 7.83–7.78 (m, 2H), 7.44 (t, *J =* 9.1 Hz, 1H), 7.20 (s, 1H), 4.29 (t, *J =* 4.5 Hz, 2H), 4.08 (t, *J =* 4.5 Hz, 2H), 3.94 (s, 3H), 3.81 (d, *J =* 11.6 Hz, 2H), 3.44 (t, *J* = 11.4 Hz, 2H) 3.18 (d, *J =* 10.4 Hz, 2H), 2.54 (d, *J =* 11.8 Hz, 2H). ^13^C NMR (126 MHz, DMSO–D_6_) δ 156.0, 154.5, 153.1 (d, ^1^*J*_C–F_ = 243.2 Hz), 152.7, 148.2, 147.0, 136.8 (d, ^3^*J*_C–F_ = 3.8 Hz), 123.4, 122.2 (d, ^3^*J*_C–F_ = 6.3 Hz), 118.8 (d, ^2^*J*_C–F_ = 18.9 Hz), 116.5 (d, ^2^*J*_C–F_ = 21.4 Hz), 108.7, 107.4, 102.7, 69.0, 67.4, 65.5, 56.2, 55.8. ^13^C NMR {^19^F} (126 MHz, DMSO–D_6_) δ 156.0, 154.5, 153.1, 152.7, 148.2, 147.0, 136.8, 123.4, 122.2, 118.8, 116.5, 108.7, 107.4, 102.7, 69.0, 67.4, 65.5, 56.2, 55.8.^19^F {^1^H} (470 MHz, DMSO–D_6_) δ −123.2. HRMS-ESI (*m*/*z*) [M + H]^+^ calculated for [C_21_H_23_O_4_N_4_ClF]^+^: 449.1386, found: 449.1378. Mp 186.4–187.5 °C (mean of *n* = 3 determinations).

##### *N*-(3-Chloro-2-fluorophenyl)-7-methoxy-6-(2-(morpholinooxy)ethoxy)quinazolin-4-amine (**7**)

To a stirred solution of **16** (1.90 g, 12.9 mmol, 1.0 equiv) in anhydrous toluene (40 mL) at 0 °C was added SOCl_2_ (3.84 mL, 32.25 mmol, 2.5 equiv) dropwise. The reaction mixture was stirred until no longer exothermic and then brought to 60 °C and stirred for 3 h. After such time, the mixture was concentrated *in vacuo* and the resulting residue was used directly in the next step without further purification.

To a stirred solution of **19** (6.19 g, 19.35 mmol, 1.5 equiv) in anhydrous DMF (70 mL) at 0 °C was added NaH (60% dispersion in mineral oil) (770 mg, 19.35 mmol, 1.5 equiv) slowly, and the solution was warmed to rt and stirred for 45 min. Following this, the crude chloride obtained in the previous step was added dropwise in DMF (10 mL) and the solution was brought to 80 °C and stirred for 16 h. After such time the mixture was concentrated *in vacuo* and coconcentrated with toluene (3×, 75 mL). The resulting residue was then redissolved in EtOAc (150 mL) and washed successively with 1 M NaOH (2×, 75 mL), brine (2×, 75 mL), then the combined organic layers were dried over Na_2_SO_4_, filtered and concentrated *in vacuo*. The residue obtained was purified by flash column chromatography (eluent: 10:90 Et_3_N/EtOAc) to afford the title compound **7** (1.67 g, 3.73 mmol, 29% over 2 steps) as an off-white powder. TLC *R_f_* = 0.50 (10:90 Et_3_N/EtOAc; UV, CAM). ^1^H NMR (500 MHz, DMSO–D_6_) δ 9.61 (s, 1H), 8.38 (s, 1H), 7.82 (s, 1H), 7.54–7.52 (m, 1H), 7.49–7.46 (m, 1H), 7.28 (t, *J =* 8.3 Hz, 1H), 7.21 (s, 1H), 4.28 (t, *J =* 4.6 Hz, 2H), 4.07 (t, *J =* 4.6 Hz, 2H), 3.94 (s, 3H), 3.81 (d, *J =* 12.0 Hz, 2H), 3.44 (t, *J =* 11.3 Hz, 2H), 3.17 (d, *J =* 10.4 Hz, 2H), 2.55–2.52 (m, 2H). ^13^C NMR (126 MHz, DMSO–D_6_) δ 156.9, 154.6, 153.0, 152.4 (d, ^1^*J*_C–F_ = 249.5 Hz), 148.2, 147.0, 128.4 (d, ^2^*J*_C–F_ = 12.6 Hz), 127.1, 126.9, 124.9 (d, ^3^*J*_C–F_ = 5.0 Hz), 120.2 (d, ^2^*J*_C–F_ = 16.4 Hz), 108.6, 107.2, 102.8, 69.0, 67.3, 65.5, 56.2, 55.9. ^13^C NMR {^19^F} (126 MHz, DMSO–D_6_) δ: 156.9, 154.6, 153.0, 152.4, 148.2, 147.0, 128.4, 127.1, 126.9, 124.9, 120.2, 108.6, 107.2, 102.8, 69.0, 67.3, 65.5, 56.2, 55.9. ^19^F {^1^H} (470 MHz, DMSO–D_6_) δ −120.4. HRMS-ESI (*m*/*z*): [M + H]^+^ calculated for [C_21_H_23_O_4_N_4_ClF]^+^: 449.1386, found:449.1376. Mp 141.3–142.9 °C (mean of *n* = 3 determinations).

##### *N*-(3-Chloro-4-fluorophenyl)-7-methoxy-6-(2-((4-methylpiperazin-1-yl)oxy)ethoxy)quinazolin-4-amine (**8**)

To a stirred solution of **17** (300 mg, 1.87 mmol, 1.0 equiv) in anhydrous toluene (7 mL) at 0 °C was added SOCl_2_ (340 μL, 4.68 mmol, 2.5 equiv) dropwise. The reaction mixture was stirred until no longer exothermic and then brought to 60 °C and stirred for 3 h. After such time, the mixture was concentrated *in vacuo*, and the resulting residue was dissolved in EtOAc (20 mL) and washed with an aq. K_2_CO_3_ solution (2×, 15 mL). The combined organic layers were then dried over Na_2_SO_4_, filtered, and concentrated. The residue obtained was used without further purification.

To a stirred solution of **18** (898 mg, 2.81 mmol, 1.5 equiv) in anhydrous DMF (10 mL) at 0 °C was added NaH (60% dispersion in mineral oil) (112 mg, 2.81 mmol, 1.5 equiv) slowly, and the solution was warmed to r.t. and stirred for 45 min. Following this, the crude chloride obtained in the previous step was added dropwise in DMF (5 mL) and the solution was brought to 80 °C and stirred for 16 h. After such time, the mixture was concentrated *in vacuo* and coconcentrated with toluene (3×, 20 mL). The resulting residue was then redissolved in EtOAc (30 mL) and washed successively with 1 M NaOH (2×, 20 mL), brine (2×, 20 mL), then the combined organic layers were dried over Na_2_SO_4_, filtered, and concentrated *in vacuo*. The residue obtained was purified by flash column chromatography (eluent: 85:10:5 EtOAc/MeOH/Et_3_N) to afford the title compound **8** (286 mg, 0.687 mmol, 33% over 2 steps) as a light orange powder. TLC *R_f_* = 0.40 (85:10:5 EtOAc/MeOH/Et_3_N; UV, CAM). ^1^H NMR (500 MHz, DMSO–D_6_) δ 9.53 (s, 1H), 8.50 (s, 1H), 8.12 (dd, *J =* 6.8, 2.6 Hz, 1H), 7.84–7.78 (m, 2H), 7.44 (t, *J =* 9.1 Hz, 1H), 7.21 (s, 1H), 4.28 (t, *J =* 5.0 Hz, 2H), 4.04 (t, *J =* 5.0 Hz, 2H), 3.94 (s, 3H), 3.15–3.13 (m, 2H), 2.70–2.67 (m, 2H), 2.57 (br s, 2H), 2.13–2.09 (m, 5H). ^13^C NMR (126 MHz, DMSO–D_6_) δ 156.0, 154.5, 153.1 (d, ^1^*J*_C–F_ = 243.2 Hz), 152.7, 148.2, 147.0, 136.8, 126, 123.4, 122.2 (d, ^3^*J*_C–F_ = 6.3 Hz), 118.8 (d, ^2^*J*_C–F_ = 18.9 Hz), 116.5 (d, ^2^*J*_C–F_ = 21.4 Hz), 108.7, 107.4, 102.7, 69.1, 67.4, 55.9, 55.0, 53.5, 45.0. ^13^C NMR {^19^F} (126 MHz, DMSO–D_6_) δ: 156.0, 154.5, 153.1, 152.7, 148.2, 147.0, 136.8, 123.4, 122.2, 118.8, 116.5, 108.7, 107.4, 102.7, 69.0, 67.4, 55.9, 55.0, 53.5, 45.0. ^19^F {^1^H} (470 MHz, DMSO–D_6_): δ −123.2. HRMS-ESI (*m*/*z*): [M + H]^+^ calculated for [C_22_H_26_O_3_N_5_ClF]^+^: 462.1703, found: 462.1691. Mp 64.0–66.5 °C (mean of *n* = 3 determinations).

##### *N*-(3-Chloro-2-fluorophenyl)-7-methoxy-6-(2-((4-methylpiperazin-1-yl)oxy)ethoxy)quinazolin-4-amine (**9**)

To a stirred solution of **17** (2.10 g, 13.12 mmol, 1.0 equiv) in anhydrous toluene (50 mL) at 0 °C was added SOCl_2_ (2.38, mL, 32.80 mmol, 2.5 equiv) dropwise. The reaction mixture was stirred until no longer exothermic, and then brought to 60 °C and stirred for 3 h. After such time, the mixture was concentrated *in vacuo*, and the resulting residue was dissolved in EtOAc (100 mL) and washed with an aq. K_2_CO_3_ solution (2×, 75 mL). The combined organic layers were then dried over Na_2_SO_4_, filtered, and concentrated. The residue obtained was used without further purification.

To a stirred solution of **19** (6.29 g, 19.68 mmol, 1.5 equiv) in anhydrous DMF (90 mL) at 0 °C was added NaH (60% dispersion in mineral oil) (783 mg, 19.68 mmol, 1.5 equiv) slowly, and the solution was warmed to rt and stirred for 45 min. Following this, the crude chloride obtained in the previous step was added dropwise in DMF (10 mL) and the solution was brought to 80 °C and stirred for 16 h. After such time the mixture was concentrated *in vacuo* and coconcentrated with toluene (3×, 25 mL). The resulting residue was then redissolved in EtOAc (150 mL) and washed successively with 1 M NaOH (2×, 100 mL), brine (2×, 100 mL), then the combined organic layers were dried over Na_2_SO_4_, filtered, and concentrated *in vacuo*. The residue obtained was purified by flash column chromatography (eluent: 85:10:5 EtOAc/Et_3_N/MeOH) to afford the title compound **9** (1.19 g, 2.58 mmol, 20% over 2 steps) as a light yellow powder. TLC *R_f_* = 0.30 (85:10:5 EtOAc/MeOH/Et_3_N; UV, CAM). ^1^H NMR (500 MHz, DMSO–D_6_) δ 9.61 (s, 1H), 8.39 (s, 1H), 7.83 (s, 1H), 7.57–7.51 (m, 1H), 7.48–7.46 (m, 1H), 7.29–7.26 (m, 1H), 7.21 (s, 1H), 4.27 (t, *J =* 4.6 Hz, 2H), 4.03 (t, *J =* 4.6 Hz, 2H), 3.94 (s, 3H), 3.15–3.12 (m, 2H), 2.69–2.67 (m, 2H), 2.57 (br s, 2H), 2.13–2.06 (m, 5H). ^13^C NMR (126 MHz, DMSO–D_6_) δ 156.9, 154.6, 153.0, 152.4 (d, ^1^*J*_C–F_ = 249.5 Hz), 148.2, 147.0, 128.4 (d, ^2^*J*_C–F_ = 11.3 Hz), 127.1, 126.8, 124.9 (d, ^3^*J*_C–F_ = 5.0 Hz), 120.2 (d, ^2^*J*_C–F_ = 16.4 Hz), 108.6, 107.2, 102.8, 69.0, 67.2, 55.9, 55.0, 53.6, 45.0. ^13^C NMR {^19^F} (126 MHz, DMSO–D_6_) δ 156.9, 154.6, 153.0, 152.4, 148.2, 147.0, 128.4, 127.1, 126.8, 124.9, 120.1, 108.6, 107.2, 102.8, 69.0, 67.2, 55.9, 55.0, 53.6, 45.0.^19^F {^1^H} (470 MHz, DMSO–D_6_) δ −120.4. HRMS-ESI (*m*/*z*): [M + H]^+^ calculated for [C_22_H_26_O_3_N_5_ClF]^+^: 462.1703, found: 462.1695. Mp 143.5–144.9 °C (mean of *n* = 3 determinations).

##### Benzyl 4-Allylpiperazine-1-carboxylate (**21**)

To a stirred solution of benzyl piperazine-1-carboxylate (**20**) (15 g, 68.14 mmol, 1.0 equiv) in anhydrous THF (250 mL) at r.t. was added K_2_CO_3_ (18.8 g, 136.28 mmol, 2.0 equiv) followed by slow addition of allyl bromide (11.8 mL, 136.28 mmol, 2.0 equiv). The solution was brought to 65 °C and stirred for 16 h, after which time the mixture was cooled to rt and quenched via the addition of 1 M NaOH (200 mL). The layers were separated and the aqueous layer was extracted with EtOAc (3×, 100 mL). The combined organic layers were dried over Na_2_SO_4_, filtered, and concentrated *in vacuo*. The residue obtained was purified by flash column chromatography on silica (eluent: 50:45:5 Hexanes/EtOAc/Et_3_N) to afford the title compound **21** (12.81 g, 49.2 mmol, 72%) as a yellow oil. TLC *R_f_* = 0.60 (50:45:5 Hexanes/EtOAc/Et_3_N; CAM, UV). ^1^H NMR (500 MHz, CDCl_3_) δ 7.37–7.29 (m, 5H), 5.89–5.78 (m, 1H), 5.23–5.14 (m, 2H), 5.13 (br s, 2H), 3.56 (m, 4H), 3.00 (d, *J =* 6.6 Hz, 2H), 2.40 (br s, 4H). ^13^C NMR (126 MHz, CDCl_3_) δ: 155.4, 136.9, 134.7, 128.6, 128.1, 128.0, 118.5, 67.2, 61.8, 52.8, 43.9. HRMS-ESI (*m*/*z*) [M + H]^+^ calculated for [C_15_H_21_N_2_O_2_]^+^: 261.1598, found: 261.1594.

##### Benzyl 4-(Allyoxy)piperazine-1-carboxylate (**22**)

To a stirred solution of **21** (12.2 g, 46.9 mmol, 1.0 equiv) in anhydrous DCM at −30 °C was added MCPBA (70% mixture with 3-chlorobenzoic acid and water) (11.56 g, 46.9 mmol, 1.0 equiv) portion-wise for 5 min. After full addition, the reaction mixture was stirred at −30 °C for 1 h, then was quenched with aq. NaHCO_3_ (150 mL), and the layers were separated. The organic layer was washed with NaHCO_3_ (3×, 150 mL) after which the organic layer was dried over Na_2_SO_4_, filtered, and concentrated *in vacuo*. The residue obtained was dissolved in toluene and heated to 80 °C with stirring for 14 h. After such time, the mixture was cooled to r.t. and concentrated *in vacuo*. The residue obtained was purified by flash column chromatography on silica (eluent: 80:15:5 Hexanes/EtOAc/Et_3_N) to afford the title compound **22** (6.13 g, 22.2 mmol, 47%) as a clear oil. TLC *R_f_* = 0.50 (30:70 EtOAc/Hexanes; CAM, UV). ^1^H NMR (500 MHz, CDCl_3_) δ 7.39–7.30 (m, 5H) 5.99–5.88 (m, 1H), 5.27 (d, *J =* 17.3 Hz, 1H), 5.18 (d, *J =* 10.3 Hz, 1H), 5.13 (s, 2H), 4.21 (d, *J =* 6.1 Hz, 2H), 4.03 (s, 2H), 3.19–3.11 (m, 4H), 2.57 (s, 2H). ^13^C NMR (126 MHz, CDCl_3_): δ 155.3, 136.8, 134.6, 128.7, 128.2, 128.1, 118.0, 73.2, 67.4, 55.3, 42.7. HRMS-ESI (*m*/*z*): [M + H]^+^ calculated for [C_15_H_21_N_2_O_3_]^+^: 277.1547, found: 277.1540.

##### 2-((4-Methylpiperazin-1-yl)oxy)ethan-1-ol (**17**)

Compound **22** (5.6 g, 20.27 mmol, 1.0 equiv) was dissolved in a mixture of DCM (200 mL) and methanol (50 mL) and cooled to −78 °C. Ozone was bubbled through the reaction mixture for 30 min until full consumption of the starting material. On completion, as indicated by ESIMS, argon was bubbled through the reaction mixture to disperse residual ozone. NaBH_4_ (1.53 g, 40.54 mmol, 2.0 equiv) was then added portion-wise at −78 °C and the solution was gradually warmed to rt and stirred for 1 h. After such time, the mixture was quenched with aq. NaHCO_3_ (200 mL) and the layers separated. The aqueous layer was extracted with DCM (2×, 150 mL), and the combined organic layers were washed with brine (2×, 150 mL), dried over Na_2_SO_4_, filtered, and concentrated *in vacuo* to afford the alcohol as a clear oil (5.26 g, 18.8 mmol, 93% crude yield). TLC *R_f_* = 0.10 (70:30 EtOAc/Hexanes; CAM, UV). ^1^H NMR (500 MHz, CDCl_3_) δ: 7.38–7.30 (m, 5H), 5.13 (s, 2H), 4.07 (s, 2H), 3.84–3.81 (m, 4H), 3.24–3.10 (m, 5H), 2.60–2.56 (m, 2H). ^13^C NMR (126 MHz, CDCl_3_) δ: 155.2, 136.6, 128.7, 128.3, 128.1, 71.9, 67.5, 65.3, 55.0, 42.7. HRMS-ESI (*m*/*z*) [M + H]^+^ calculated for [C_14_H_21_O_4_N_2_]^+^: 281.1496, found: 281.1489.

The so-obtained crude alcohol (5.26 g, 18.8 mmol, 1.0 equiv) obtained in the previous step was dissolved in anhydrous THF (68 mL) and cooled to 0 °C. LiAlH_4_ (2.31 g, 60.81 mmol, 3.2 equiv) was then added portion-wise for 5 min. After full addition, the solution was warmed to r.t. and stirred for 2 h. After such time, the reaction mixture was cooled back to 0 °C and quenched by slow addition of H_2_O. After effervescence ceased, the solution was acidified with concentrated HCl until pH ≈ 2. The aqueous layer was then extracted with EtOAc (4×, 150 mL), after which, the aqueous layer was cooled to 0 °C and basified to pH ≈ 10 by portion-wise addition of solid NaOH. The basified aqueous layer was extracted with EtOAc (4×, 100 mL) and the resulting organic layer was dried over Na_2_SO_4_, filtered, and concentrated *in vacuo* to afford the title compound **17** (1.63 g, 10.2 mmol, 50% yield over 3 steps from **22**) as a yellow oil with spectral data identical to that of the above sample.

### Kinase Activity

*In vitro* kinase activity (inhibitor binding constants (*K*_d_) and biochemical inhibition (IC_50_’s)) were assessed by Eurofins DiscoverX (*K*_d_ determinations) and Eurofins Cerep (biochemical IC_50_ determinations). For *K*_d_ determinations, compounds were run in duplicate (*n* = 2) and assayed using an 11-point, 3-fold dilution series at a top compound testing concentration of 10 μM with Eurofin’s KINOMEScan KdELECT assay. For biochemical IC_50_ determinations, compounds were run in duplicate (*n* = 2) and tested in an enzymatic radiometric assay using a 9-point, half-log dilution series at a top compound testing concentration of 10 μM and an ATP concentration of 10 μM with Eurofin’s KinaseProfiler technology.

### Cell Lines

The A431, HCC827, SK-BR-3, ZR-75–30, AU565, and Caco-2 cell lines were obtained from ATCC. The NCI-H1975 cell line was obtained from SIBS. The NCI-H3255 cell line was obtained from CoBioer. Engineered Ba/F3 cell lines were obtained from Crown Bioscience. MCKII-MDR1 cells were obtained from The Netherlands Cancer Institute. HEK293 cells were obtained from Invitrogen. A431 cells were cultured in DMEM (Life Technologies) with 10% FBS. HCC827, NCI-H1975, and AU565 cells were cultured in RPMI1640 (Invitrogen) with 10% FBS. NCI-H3255 cells were cultured in BEGM (Lonza) with 10% FBS. Ba/F3 EGFR-del E746_A750/C797S and Ba/F3 EGFR-L858R/C797S cells were cultured in RPMI (Invitrogen) with 10% FBS. SK-BR-3 cells were cultured in McCoy’s 5a (Invitrogen) with 10% FBS. ZR-75-30 cells were cultured in RPMI1640 (Invitrogen) with 20% FBS. Caco-2 and MCKII-MDR1 culture information is given in the Permeability Studies section. HEK293 cells were cultured in DMEM (Gibco) with 10% FBS, 0.1 mM NEAA, 25 mM HEPES, 100 U/mL penicillin-streptomycin, μg/mL blasticidin, and 400 μg/mL Geneticin. All cells were cultured in a humidified incubator with 5% CO_2_ at 37 °C.

### Cell Viability Assay

Viability assays using A431, HCC827, NCI-H1975, NCI-H3255, Ba/F3 EGFR-del E746_A750/C797S, Ba/F3 EGFR-L858R/C797S, AU565, SK-BR-3, and ZR-75-30 cells were performed at Crown BioScience. Cells were plated into 96-well plates at 1500–7000 cells per well and dosed in triplicate (*n* = 3) in a nine-point, 4-fold dilution series with compounds (0.15 nM to 10 μM) in DMSO and incubated for 72 h. After 72 h, cell viability was assayed by CellTiter-Glo Luminescent Viability Assay (Promega). Dose–response curves were generated and used to calculate the IC_50_ values which were calculated on GraphPad Prism from the nonlinear regression equation fitted with a sigmoidal dose response and are presented as mean ± SEM.

### Lipophilicity Assay

Compound lipophilicity was determined by Pharmaron, Inc. using the shake-flask method with 1-octanol and PBS (pH 7.4). Each compound was assessed in duplicate (*n* = 2) at 1 μM in a 96-well plate shaken at 25 °C and 2000 rpm for 2 h. Samples were analyzed by liquid chromatography and tandem mass spectrometry (LC-MS/MS).

### Solubility Assay

Solubility was determined by Pharmaron, Inc. and each compound was assessed in duplicate (*n* = 2) by adding 15 μL of compound stock solution (10 mM in DMSO) into a 96-well plate along with 485 μL of buffer followed by shaking at 25 °C, 1100 rpm for 2 h. Wells were filtered, and samples (5 μL) were taken followed by dilution with an equal volume of DMSO (5 μL) and 490 μL of aqueous solution. Samples were analyzed by LC-MS/MS.

### Plasma Protein Binding and Brain Tissue Binding Assays

Plasma protein binding and brain tissue binding were determined by Pharmaron, Inc. using the equilibrium dialysis method. Each compound was assessed in duplicate (*n* = 2) at 5 μM with the final percent volume of the organic solvent at 0.5%. Compounds were incubated at 37 °C, 5% CO_2_ for 6 h at 100 rpm, and samples (50 μL) were taken at the beginning and end of the incubation. Following incubation, the samples were analyzed by LC-MS/MS and the concentrations of the compounds were determined in the buffer and plasma solution chambers.

### Metabolic Stability Assay

Assessment of the compound’s metabolic stability in incubations containing liver microsomes and hepatocytes of human and rat using the compound depletion approach was performed by Pharmaron, Inc. Compound **9** underwent additional stability evaluation in monkey and dog hepatocytes. Microsome stability was assessed in duplicate (*n =* 2) in the presence and absence of nicotinamide adenine dinucleotide phosphate (NADPH) with liver microsomes (0.5 mg/mL) from human (BD Gentest) and Sprague-Dawley rats (BD Gentest). Compounds were incubated in liver microsomes at 1 μM and samples (30 μL) were taken at 0.5, 15, 30, 45, and 60 min time points for analysis by LC-MS/MS. Hepatocyte stability was assessed in duplicate (*n =* 2) at a working cell density of 0.5 × 10^6^ cells/mL with hepatocytes from human (BiolVT), Sprague-Dawley rat (BiolVT), cynomolgus monkey (RILD) or beagle dog (BiolVT). Compounds were incubated in hepatocytes at 1 μM and samples (25 μL) were taken at 0.5, 15, 30, 60, 90, and 120 min time points for analysis by LC-MS/MS. The *in vitro* half-life and intrinsic clearances were determined as previously described.^76^

*In vitro* half-life (*in vitro t*_1/2_) was determined from the slope value in
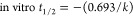
where *k* was determined by linear regression of the natural logarithm of the remaining percentage of the parent drug vs the incubation time curve.

### Plasma Stability Assay

Assessment of compounds to determine stability in incubations containing plasma of human (Pharmaron) and Sprague-Dawley rat (IPHASE) was performed by Pharmaron, Inc. using the compound deletion approach. Compounds were assessed in duplicate (*n =* 2) and incubated in plasma at 5 μM and samples (50 μL) were taken at 0, 15, 30, 60, and 120 min time points for analysis by LC-MS/MS. Peak area ratios were determined from the extracted ion chromatogram and the percent compound remaining at each time point was calculated by the following equation:
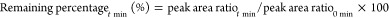
where peak area ratio_*t* min_ is the peak area ratio of control and test compound at *t* min and peak area ratio_0 min_ is the peak area ratio of control and test compound at zero time point.

### Permeability Studies

Compounds were evaluated at Pharmaron, Inc. for their ability to permeate Caco-2 cells and MDCKII-MDR1 cells. A 96-well HTS Transwell plate (Corning) was used for Caco-2 and MDCKII-MDR1 cell seeding. Caco-2 cells were seeded at a density of 6.86 × 10^5^ cells/mL and cultivated for 14–18 days prior to assays. MDCKII-MDR1 cells were seeded at a density of 1.56 × 10^6^ cells/mL and cultivated for 4–8 days prior to assays. To determine the rate of drug transport in both the absorptive (apical to basolateral (A–B)) and secretory (basolateral to apical (B–A)) directions, compounds (5 μM in DMSO for Caco-2 cells, and 1 μM in DMSO for MDCKII-MDR1 cells) were added to the donor wells. Plates were incubated at 37 °C for 2 h, and samples (50 μL) were taken at the beginning and end of the incubation in both the donor and acceptor wells with the assay run in duplicate (*n =* 2). Samples were analyzed by LC-MS/MS. The apparent permeability coefficient (*P*_app_) in units (cm/s × 10^–6^) was calculated using the following equation:
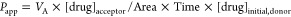
where *V*_A_ represents the volume (mL) in the acceptor well, Area is the surface of the membrane (0.143 cm^2^ for Transwell 96-well plate), and Time is the total transport time in seconds.

The efflux ratio was determined by using the following equation:
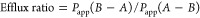
where *P*_app_ (B–A) indicates the apparent permeability in the basolateral to apical direction and *P*_app_ (A–B) indicates the apparent permeability in the apical to basolateral direction.

### hERG Channel Inhibition Assay

The potential inhibitory effect of compounds on the hERG channel was assessed by Pharmaron, Inc. using the manual patch clamp system as previously described.^77^ The HEK293 cell line (Invitrogen) stably transfected with the hERG gene was employed. Compounds were tested at 5 concentrations (0.37, 1.11, 3.33, 10, and 30 μM) and run in triplicate (*n =* 3). IC_50_ values were determined by plotting the % inhibition against the concentration of compounds using GraphPad Prism from the nonlinear regression equation fitted with a sigmoidal dose–response curve, and the IC_50_ values are presented as mean ± SEM.

### Determination of Human CYP450 Inhibition by **9**

Assessment of compound **9** was carried out at Pharmaron, Inc. for its potential to inhibit cytochrome P450 (CYP) isoforms using human liver microsomes. The activities tested were CYP1A2-mediated phenacetin *O*-demethylation, CYP2C19-mediated (*S*)-mephenyotin 4′-hydroxylation, CYP2C9-mediated diclofenac 4′-hydroxylation, CYP2D6-mediated dextromethorphan *O*-demethylation, and CYP3A4-mediated midazolam 1′-hydroxylation. Concentrations of substrates were phenacetin (40 μM), mephenytoin (50 μM), diclofenac (6 μM), dextromethorphan (2 μM), and midazolam (1 μM). Probe substrates phenacetin, mephenytoin, and dextromethorphan were incubated at 37 °C for 20 min. Probe substrates diclofenac and midazolam were incubated at 37 °C for 5 min. Compound (**9**) was tested in an eight-point, 3-fold dilution series (0.01–30 μM) in DMSO and incubated with pooled human liver microsomes (0.5 mg/mL) (BD Gentest) and a cocktail of the probe substrates for selective CYP isoform. The reactions were initiated by adding NADPH (1 mM final concentration) after a 5 min preincubation, and the assay was run in duplicate (*n =* 2). Samples were analyzed by ultra-performance liquid chromatography tandem mass spectrometry (UPLC-MS/MS). Inhibition of each P450 enzyme was measured as the percentage decrease in the activity of marker metabolite formation compared to noninhibited controls. IC_50_ values were determined on GraphPad Prism with remaining activity (%) and logarithm of inhibitor concentrations fitted with a nonlinear fit [inhibitor] vs normalized response with variable slope.

### Determination of CYP2D6 Time-Dependent Inhibition by **9**

Assessment of compound **9** was carried out at Pharmaron, Inc. for the potential time-dependent inhibition of CYP2D6 using human liver microsomes (BD Gentest) and primary human hepatocytes (BiolVT). Bufuralol (2 μM) was used as a substrate for liver microsomes, and dextromethorphan (40 μM) was used for hepatocytes. The activities tested were CYP2D6-mediated bufuralol 1′-hydroxylation and CYP2D6-mediated dextromethorphan *O*-demethylation. Compound **9** was assayed in duplicate (*n =* 2) and tested in a six-point, 3-fold dilution series (0.03–10 μM) in DMSO and incubated with either pooled human liver microsomes (0.5 mg/mL) or human hepatocytes (0.3 × 10^6^ cells/mL). Incubations in pooled human liver microsomes were carried out with NADPH (1 mM final concentration) with or without 30 min preincubation at 37 °C, 5% CO_2_. Incubations in human hepatocytes were carried out without preincubation or with 30 min preincubation at 37 °C, 5% CO_2_. The experiment was carried out at 37 °C in 5% CO_2_ for 5 min. Inhibition of CYP2D6 was measured as the percentage decrease in the activity of marker metabolite formation compared to noninhibited controls. IC_50_ values were determined on GraphPad Prism with remaining activity (%) and logarithm of inhibitor concentrations fitted with a nonlinear fit [inhibitor] vs normalized response with variable slope.

### AMES Fluctuation Assay

Compounds were assessed for mutagenicity at the Eurofins Panlabs in 384-well plates using four *Salmonella* strains: TA98, to probe for frameshift mutation (with quercetin as control), TA100 and TA1535, to probe for base pair insertions/deletions (with streptozotocin as control), and TA1537, to probe for frameshift mutations (with aminacridine as control) as previously described.^78^ Each compound was incubated at 37 °C for 96 h at four different concentrations (5, 10, 50, and 100 μM), with each concentration tested using 48 replicates, both with and without rat liver S9 metabolic activation. A concurrent bacterial cytotoxicity assay was run at 0.6, 1.2, 2.5, 5, 10, 25, 50, and 100 μM to rule out false negatives. Bacterial cytotoxicity was expressed as percent of control growth (OD_650_). Compounds with growth of less than 60% control were flagged and considered bacterial cytotoxic. Wells that displayed bacterial growth due to the reversion of the histidine mutation (as judged by the ratio of OD_430_/OD_570_ being greater than 1.0) were counted and recorded as positive counts. Significances of the positive counts between the treatment and control were determined using a one-tailed Fisher’s exact test.

### *In Vitro* Micronucleus Test

Compound **9** was assessed for genotoxicity by Eurofins Panlabs using the *in vitro* micronucleus test in Chinese hamster ovary (CHO-K1) cells as previously described.^79^ Compound **9** was incubated in a 96-well plate format at varying concentrations (8, 16, 31, 62, 125, 250, 500, and 1000 μM) for 4 h at 37 °C with metabolic activation by rat liver S9 and 24 h at 37 °C without metabolic activation by rat liver S9. High content analysis and fluorescence imaging were used to detect micronuclei and compared against positive controls (cyclophosphamide (ran at 7.2 μM) and mitomycin C (ran at 0.3 μM)). Significances of the positive counts between the treatment and control were determined by one-tailed *t* test with two-sample equal variance.

### *In Vitro* Metabolite Identification of **9**

Compound **9** underwent *in vitro* metabolite identification by Pharmaron, Inc. A 200 μL sample of compound **9** was tested at 10 μM in human and rat hepatocytes (1.0 × 10^6^ cells/mL) and incubated for 0, 120, and 240 min at 37 °C. After incubation, reactions were quenched with 2 volumes of acetonitrile followed by centrifugation for 15 min at 16,000*g*. Supernatants were then analyzed by UHPLC-MS/MS. A figure of identified metabolites is provided in the Supporting Information (Figures S1–S2).

### Kinase Selectivity Determination of **9**

Profiling of a 468-member human kinase panel was performed with Eurofins DiscoverX using the KINOMEScan platform. A panel of 468 kinases was assayed at a single concentration of 1 μM for **9**. Percent control was mapped onto the kinome tree using TREE*spot*. The S scores were calculated as previously described^64^ and are reflective of the number of kinases bound by **9** over the total number of wild-type kinases.

### Animal Studies

Animal experiments were performed at Pharmaron, Inc. and animal use was approved by Pharmaron’s Institutional Animal Care and Use Committee (IACUC) in Pharmaron (Pharmaron IACUC, Protocol #PK-M-07182022, #PK-R-06012022, and #ON-CELL-XEN-06012022) following the guidance of AAALAC. Six- to eight-week-old male Sprague-Dawley rats (approximately 200–300 g) obtained from Si Bei Fu Laboratory Animal Technology Co., six- to eight-week-old male CD1 mice (approximately 20–30 g) obtained from Si Bei Fu Laboratory Animal Technology Co., and six- to eight-week-old female BALB/c nude mice (approximately 20–30 g) obtained from Si Bei Fu Laboratory Animal Technology Co., were used in the pharmacokinetic studies. Six- to eight-week-old female BALB/c nude mice (approximately 18–22 g) obtained from Beijing Anikeeper Biotech Co., Ltd. were used in the intracranial PDX study. Animals were housed at 20–25 °C with humidity ranging from 40 to 70% relative humidity, were exposed to 12 h light and dark cycles, and were supplied with food and water ab libitum.

### Pharmacokinetic Studies of **9**

Standard pharmacokinetic assessment of **9** was done by IV (intravenous) tail vein injection (formulation: DMSO: 10% captisol in saline = 1:99) and PO (oral) oral gavage (formulation: 1% methylcellulose) followed by blood sampling at 8 time points for IV (0.0833, 0.25, 0.5, 1,2, 4, 7, 24 h post dose) and 7 time points for PO (0.25, 0.5, 1, 2, 4, 8, 24 h post dose) with *n* = 3 animals per dosing route (*n* = 6 total). Approximately 0.03 mL of blood was collected from the dorsal metatarsal vein (in the case of CD1 mice), 0.03 mL of blood from the orbit vein (in the case of BALB/c nude mice), or 0.2 mL of blood from the jugular vein (in the case of Sprague-Dawley rats) at each time point. Blood at each sampling point was transferred into a plastic micro centrifuge tube containing K_2_-EDTA, and collection tubes with blood samples and anticoagulant were inverted several times for proper mixing of the tube contents and placed on ice prior to centrifugation for plasma. Blood samples were centrifuged at 4 °C, 4000*g* for 5 min to obtain plasma. Samples were stored in a freezer at −75 °C prior to analysis. Concentrations of test articles in the plasma samples were determined using LC-MS/MS and WinNonlin 8.3 (Phoenix) was used for pharmacokinetic calculations. The values obtained were plotted on GraphPad Prism software and are presented as mean ± SD.

Oral CNS pharmacokinetic assessment of **9** was done by oral gavage (formulation: 1% methylcellulose) followed by blood and brain sampling at 7 time points (0.25, 0.5, 1, 2, 4, 8, 24 h post dose) with *n* = 3 animals per time point (*n* = 21 total). For plasma collection, approximately 0.03 mL of blood was collected from the dorsal metatarsal vein (in the case of CD1 mice) or 0.2 mL from the jugular vein (in the case of Sprague-Dawley rats) at each time point. Blood at each sampling point was transferred into a plastic micro centrifuge tube containing K_2_-EDTA, and collection tubes with blood samples and anticoagulant were inverted several times for proper mixing of the tube contents and placed on ice prior to centrifugation for plasma. Blood samples were then centrifuged at 4 °C, 4000*g* for 5 min to obtain plasma, and the samples were stored in a freezer at −75 °C prior to analysis. For brain sample collection, rodents were fully exsanguinated prior to collection, and the thoracic cavity was opened exposing the heart, which was catheterized from the left ventricular. A small incision was then made at the right atrial appendage and a gentle administration of saline via syringe was performed. Brain samples were collected at each time point and quickly frozen in an ice box. Samples were stored at −75 °C prior to analysis. All brain samples were prepared with water to achieve a brain weight (g)/water volume (mL) ratio of 1:4 prior to analysis. Concentrations of **9** in the plasma and brain samples were determined using LC-MS/MS and WinNonlin 8.3 (Phoenix) was used for pharmacokinetic calculations. The values obtained were plotted on GraphPad Prism software and are presented as mean ± SD.

### Intracranial PDX Model of **9**

HCC827 cells stably expressing luciferase (HCC827-luc) were injected intracranially. Briefly, 3 × 10^5^ HCC827-luc tumor cells suspended in 2 μL of RPMI1640 medium were injected into the right forebrain of anesthetized mice (anesthetic: intramuscular injection of ZoletlTM 50 (Virbac S.A)). Mice were imaged biweekly using IVIS Lumina III (PerkinElmer). Images were acquired 10 min post intraperitoneal (IP) injection with 15 mg/mL (at 5 μL/g body weight) of D-luciferin in anesthetized mice (anesthetic: 1–2% isoflurane inhalation). On day 20 postcellular inoculation, mice were randomized into 2 treatment groups (*n* = 10 mice per group): (1) 1% methylcellulose as vehicle control and (2) **9** (10 mg/kg) (formulation: 1% methylcellulose), PO, b.i.d. (twice daily) dosing (12 h intervals) × 7 days for 21 days. Body weights of all mice were measured biweekly throughout the study and BW change, expressed in % was calculated using the following formula:
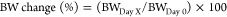
where BW_Day X_ is the BW on a given day and BW_Day 0_ is BW on day 0 (initiation of treatment).

### Statistical Analyses

Statistical analysis was performed by using GraphPad Prism 9.0. Data are presented as mean ± SD or SEM as indicated when *n* = ≥3, or as mean when *n* = 2. For *in vitro* ADME and kinase activity studies, data is presented as a mean of *n =* 2 independent replicates. For *in vivo* PK experiments, data is presented as mean ± SD, *n* ≥ 3 animals per study arm. For *in vitro* short-term growth delay experiments, IC_50_ values were determined from the nonlinear regression equation fitted with a sigmoidal dose response curve and are presented as mean ± SEM, *n* = 3 independent replicates. Intracranial PDX study data (bioluminescence and body weight means on day-21 post-treatment initiation, *n* = 10 animals per study arm) were compared using a two-tailed unpaired *t*-test. A *P* value of <0.05 was considered statistically significant.

## Supporting Information Available

Additional *in vitro* ADMET data and characterization data; NMR spectra; X-ray crystallographic data for **6** (Tables S1–S48) (Figures S1–S21); and references (PDF)
Molecular formula string spreadsheet of compounds **1**, **6**-**9** with cellular IC_50_ values and *in vitro* ADMET data (CSV)

## Abbreviations Used

ABL1: Abelson murine leukemia viral oncogene homologue 1
Aq. Sol.: aqueous solubility at pH 7.4
BCRP: breast cancer resistance protein
BDE: bond dissociation energy
BM: brain metastases
C: cynomolgus monkey
*C*_max_: maximal concentration
Caco-2: colon carcinoma cell line
DRAK1: death receptor-associated kinase 1
FBS: fetal bovine serum
*f*_u_: fraction unbound
H: human
HEPCl_int_: intrinsic clearance in hepatocytes
HER2: human epidermal growth factor receptor 2
^*i*^PrMgCl·LiCl: isopropylmagnesium chloride lithium chloride
*K*_d_: dissociation constant
*K*_p_: brain-to-plasma partitioning coefficient
*K*_p,uu_: unbound brain-to-unbound plasma partitioning
LiAlH_4_: lithium aluminum hydride
LMCl_int_: intrinsic clearance in liver microsomes
logD_7.4_: logarithm of distribution at pH 7.4
LYN: Lck/Yes novel kinase
M: mouse
MDCK: madine-darby canine kidney cell line
MetID: metabolite identification
MMP: matched molecular pair
MPO: multiparameter optimization
MTHP: 2-methyltetrahydropyranyl
nd: not determined
NaBH_4_: sodium borohydride
*P*_app_: apparent permeability
PDA: photo diode array
PDX: patient-derived xenograft
PIKFYVE: phosphoinositide kinase, FYVE-type zinc finger containing
R: rat
SD: standard deviation
SD Rat: Sprague–Dawley Rat
SEM: standard error of the mean
TDI: time-dependent inhibition
TKI: tyrosine kinase inhibitor
*T*_max_: time to reach maximal concentration
*V*_ss_: volume of distribution at steady state
