# Supplementary material for: Discovery of a Hydroxylamine-Based Brain-Penetrant EGFR Inhibitor for Metastatic Non-Small-Cell Lung Cancer
Source: J Med Chem. 2023 Nov 7;66(22):15477–92. doi: 10.1021/acs.jmedchem.3c01669 (PMC10683025; doi:10.1021/acs.jmedchem.3c01669)
Supplement: Supplementary file 1 — jm3c01669_si_001.pdf [file jm3c01669_si_001.pdf]

Supporting Information for

Discovery of a Hydroxylamine-Based Brain Penetrant EGFR Inhibitor for Metastatic Non-Small-Cell Lung Cancer

Jarvis Hill,[1,2] Robert M. Jones,[3] David Crich[1,2,4,*]

[1]Department of Pharmaceutical and Biomedical Sciences, University of Georgia, 250 West Green Street, Athens, GA 30602, USA.

[2]Department of Chemistry, University of Georgia, 302 East Campus Road, Athens, GA 30602, USA.

[3]P.O. Box 568, Oakley, UT 84055-0568, USA.

[4]Complex Carbohydrate Research Center, University of Georgia, 315 Riverbend Road, Athens, GA 30602, USA.

[*]To whom correspondence should be addressed: David.Crich@uga.edu (D.C.)

Table of Contents

1. Extended Figures S2
2. Biological Materials and Data S7
3. Supporting Chemistry Experimental Details S43
4. Catalog of Spectra S50
5. X-ray Crystal Structure of **6** S114
6. References S116

1. Extended Figures

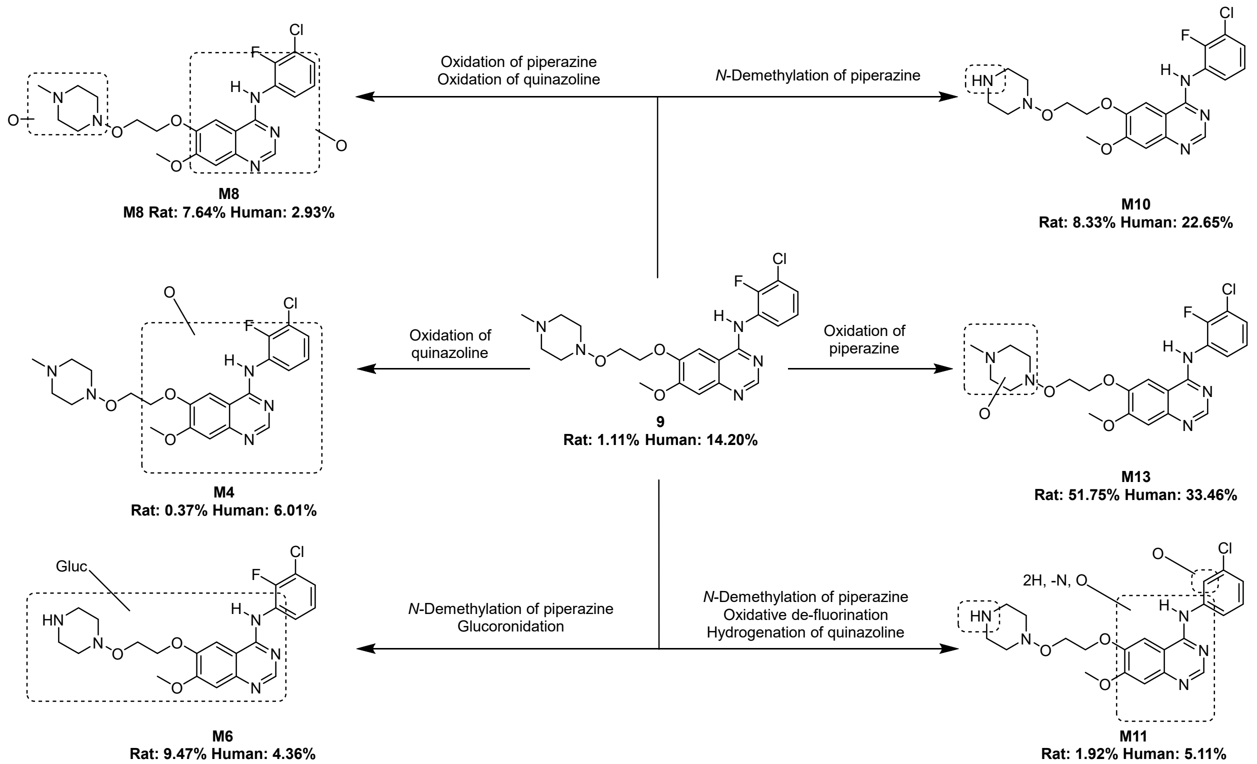

Figure S1. Major metabolites (> 2% in humans) identified after incubation of compound **9** in human and rat hepatocytes. Sites of metabolism have been highlighted by circling the scaffold of interest. O, oxidation; Gluc; glucuronide; 2H, hydrogenation.

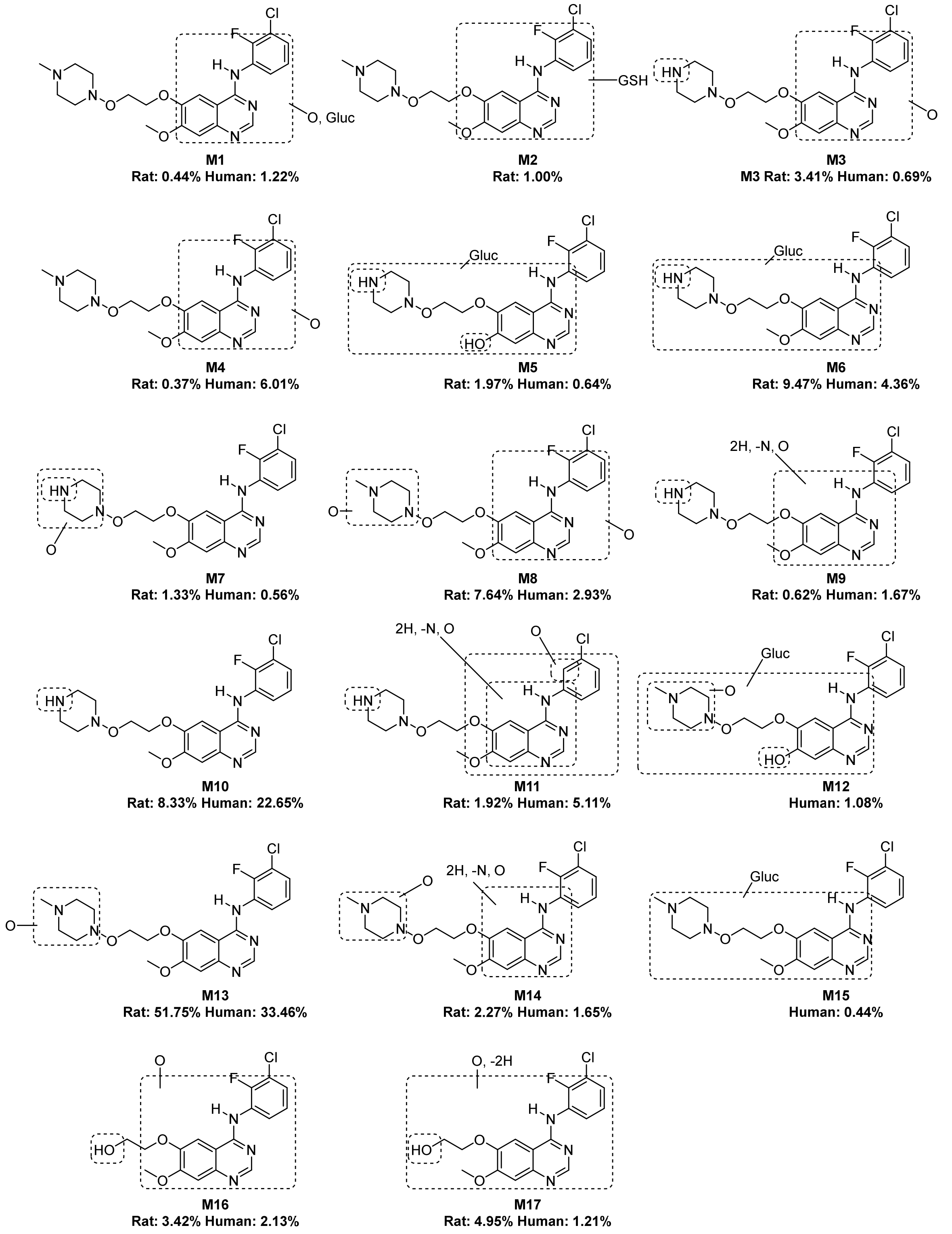

Figure S2. All identified metabolites formed after incubation of compound **9** in human and rat hepatocytes. Sites of metabolism have been highlighted by circling the scaffold of interest.O, oxidation; Gluc; glucuronide; 2H, hydrogenation; GSH, glutathione.

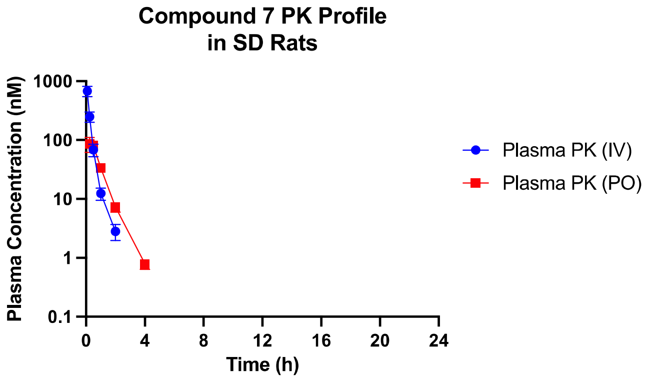

Dose (route)	$AUC_{0\text{-inf}}$ (nM·h)	$t_{1/2}$ (h)	*F* (%)	T_{max} (h)	C_{max} (nM)	CL ($mL{\cdot}min^{-1}{\cdot}kg$)	V_{ss} (L/kg)
Rat 1 mg/kg (IV)	222 ± 43	0.46 ± 0.22	- - -	- - -	- - -	76.9 ± 14.0	1.07 ± 0.29
Rat 5 mg/kg (PO)	89 ± 12	0.56 ± 0.02	8	0.33 ± 0.14	93 ± 13	- - -	- - -

Figure S3. Total plasma vs time profile (0 to 24 h) of **7** after administration into SD rats at a single dose of 1 mg/kg IV and 5 mg/kg PO. $AUC_{0\text{-inf}}$ (nM·h), area under concentration time curve from 0 to ∞; $t_{1/2}$ (h), mean elimination half-life obtained from either intravenous infusion (IV) or oral gaavge (PO); *F*(%), bioavailability (%); T_{max} (h), time to reach peak plasma concentration; C_{max} (nM), peak plasma concentration; CL ($mL{\cdot}min^{-1}{\cdot}kg$), clearance obtained from intravenous infusion. For pharmacokinetic profiles, points indicate the mean and error bars indicate SD; $n = 3$ animals per route ($n = 6$ total). Values represent the mean ± SD.

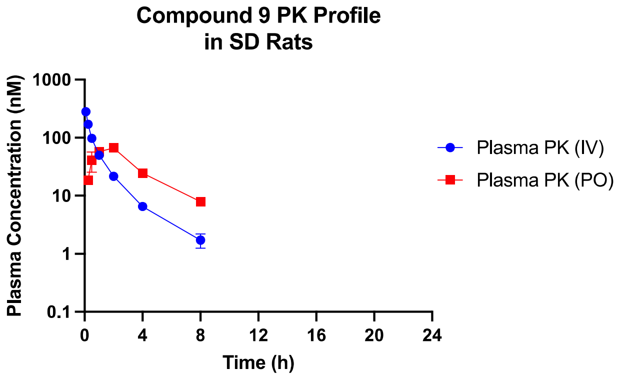

Dose (route)	$AUC_{0\text{-}inf}$ (nM·h)	$t_{1/2}$ (h)	*F* (%)	T_{max} (h)	C_{max} (nM)	CL (mL·min^{-1}·kg)	V_{ss} (L/kg)
Rat 1 mg/kg (IV)	219 ± 7	1.69 ± 0.19	- - -	- - -	- - -	76.1 ± 2.6	5.92 ± 0.93
Rat 5 mg/kg (PO)	279 ± 28	2.29 ± 0.15	25	1.67 ± 0.58	68 ± 9	- - -	- - -

Figure S4. Total plasma vs time profile (0 to 24 h) of **7** after administration into SD rats at a single dose of 1 mg/kg IV and 5 mg/kg PO. $AUC_{0\text{-}inf}$ (nM·h), area under concentration time curve from 0 to ∞; $t_{1/2}$ (h), mean elimination half-life obtained from either intravenous infusion (IV) or oral gaavge (PO); *F*(%), bioavailability (%); T_{max} (h), time to reach peak plasma concentration; C_{max} (nM), peak plasma concentration; CL (mL·min^{-1}·kg), clearance obtained from intravenous infusion. For pharmacokinetic profiles, points indicate the mean and error bars indicate SD; $n = 3$ animals per route ($n = 6$ total). Values represent the mean ± SD.

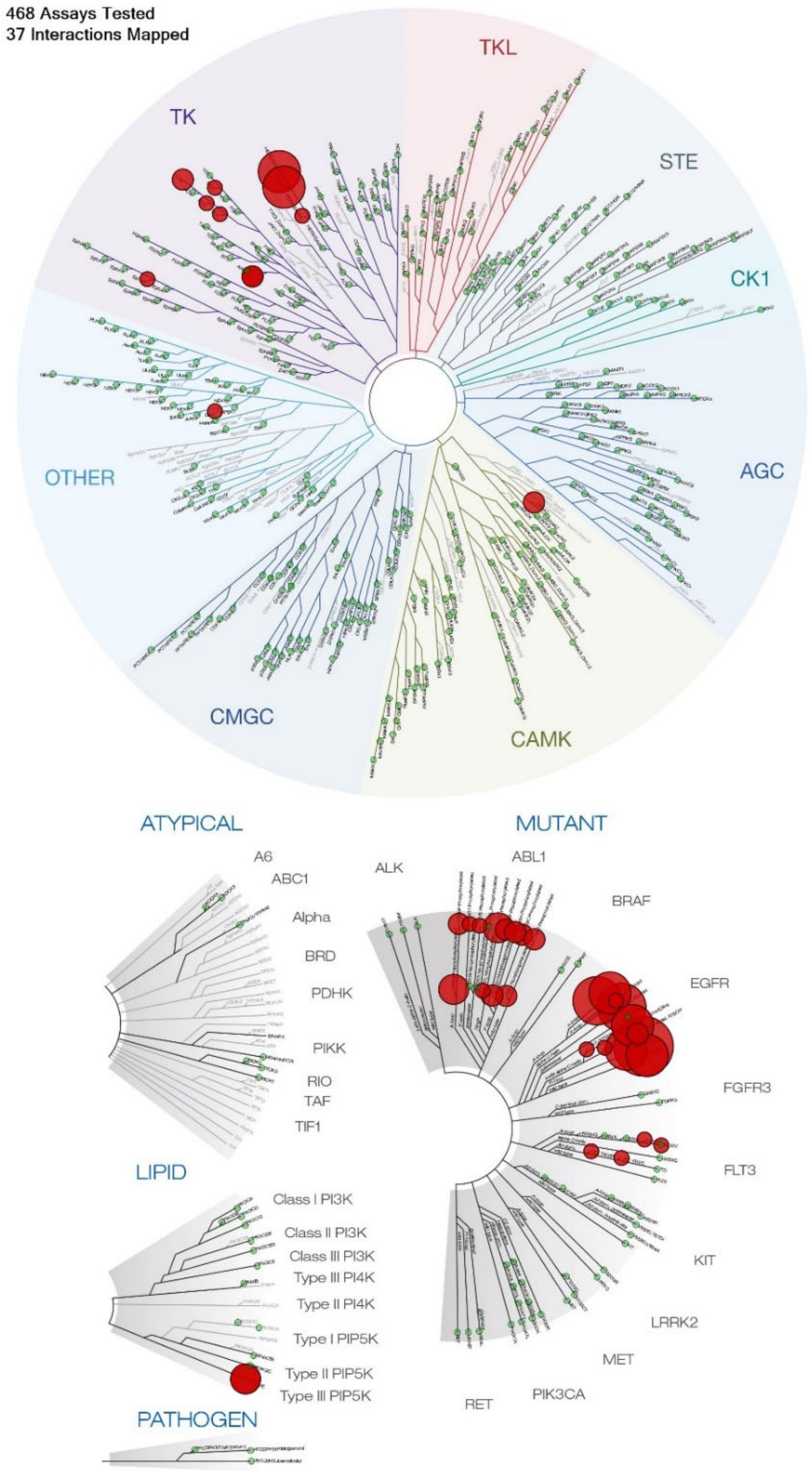

Figure S5. Full KINOMEScan[1] screening results of 9 at a screening concentration of 1 µM. The size of circles mapped onto the phylogenetic tree using DiscoverX TREEspot corresponds to strength of binding affinity.

2. Biological Materials and Data

KINOMEScan K_d determination

Table S1. K_d results for compounds against $EGFR^{wt}$ and activating mutant EGFR.

**Compound**	**Gefitinib (1)**	**6**	**7**	**8**	**9**
**EGFR K_d (nM)[a]**					
wt	0.9	0.6	< 0.2	0.8	< 0.2
L858R	1.2	0.8	< 0.2	0.6	< 0.2
del E746_A750	0.5	0.3	< 0.2	0.4	< 0.2

[a]Values < 0.2 nM were below the sensitivity of the test and as such are reported as < 0.2 nM.

Table S2. Extended inhibitor binding profile for **9** against kinases which showed ≤ 10% activity in KINOMEScan.

**Compound**	**Kinase K_d (nM)**	BCR-ABL^{wt} (Nonphos.)	BCR-ABL^{wt} (Phos.)	DRAK1	ERBB2	LYN
**9**		260	180	87	21	110

KinaseProfiler Biochemical human RTK kinase enzymatic radiometric assay

Table S3. Biochemical inhibition results for compounds against $EGFR^{wt}$ and $EGFR^{L858R}$.

**Compound**	**Gefitinib (1)**	**6**	**7**	**8**	**9**
**EGFR IC_{50} (nM)[a]**					
wt	4	5	3	3	3
L858R	< 1	< 1	1	2	2

[a]Values < 1 nM were below the sensitivity of the test and as such are reported as < 1 nM.

Table S4. Extended biochemical inhibition profile for **9** against kinases which showed ≤ 10% activity in KINOMEScan.

**Compound**	**Kinase IC_{50} (nM)**	BCR-ABL^{wt}	DRAK1	ERBB2	ERBB4	LYN
**9**		976	1033	12	330	58

Plasma protein binding by equilibrium dialysis

Table S5. Plasma details used in the plasma protein binding assay.

**Item**	**Supplier**
Human plasma (mixed gender)	BiolVT (Batch No. HMN 666664)
Mouse plasma (CD-1/mixed gender)	IPHASE or BiolVT (Batch No. MSE427337, M220076)
Rat plasma (SD/mixed gender)	BiolVT (Batch No. RAT501985)

Table S6. Results for plasma protein binding in human, rat and mouse plasma.

**Compound**	**Species**	**%Bound**	**%Unbound**	**%Recovery**	**%Remaining at 6 h**
Ketoconazole	Human	99.27	0.73	101.21	100.67
Gefitinib (**1**)	Human	92.34	7.66	105.65	98.81
**6**	Human	94.88	5.12	101.24	99.52
**7**	Human	95.88	4.12	98.88	99.36
**9**	Human	95.58	4.42	101.33	102.02
Ketoconazole	Rat	99.50	0.50	103.90	102.07
Gefitinib (**1**)	Rat	97.51	2.49	104.92	104.64
**7**	Rat	89.54	10.46	109.57	100.75
**9**	Rat	87.25	12.75	105.51	99.63
Ketoconazole	Mouse	99.50	0.50	90.16	92.31
Gefitinib (**1**)	Mouse	92.19	7.81	99.39	98.00
**7**	Mouse	93.35	6.65	98.36	102.01
**9**	Mouse	92.98	7.02	95.39	92.88

Brain tissue binding by equilibrium dialysis

Table S7. Brain homogenate details used in the brain tissue binding assay.

Item	Supplier
Rat Brain Homogenate SD Male	Pharmaron (Lot No. PH-Rat(SD)-07132022)
Mouse Brain Homogenate CD-1 Male	Pharmaron (Lot No. PH-Mouse (CD-1)-11282022)

Table S8. Results for brain tissue binding in rat and mouse brain homogenates.

Compound	Species	%Bound$_{brain}$	%Unbound$_{brain}$	%Recovery	%Remaining at 6 h
Telmisartan	Rat	98.54	1.46	107.20	100.46
Gefitinib (**1**)	Rat	99.40	0.60	99.73	104.52
**7**	Rat	95.22	4.78	94.71	98.32
**9**	Rat	95.53	4.47	102.08	94.70
Telmisartan	Mouse	98.57	1.43	108.95	107.50
**9**	Mouse	95.95	4.05	92.98	92.64

Metabolic stability in human and rat liver microsomes

Table S9. Microsome details used in the microsome materials assay.

Item	Supplier
Human Liver Microsomes, Pooled, Mixed Gender	BD Gentest (Cat No. 452117; Lot No. 38296, 38297)
Rat Liver Microsomes, Pooled, Male SD	BD Gentest (Cat No. 452501, Lot No. 1300002)

Table S10. Results for metabolic stability in human and rat liver microsomes expressed as % remaining.

Compound	Species	Assay Format	Remaining Percentage (%) 0.5 min	15 min	30 min	45 min	60 min
Verapamil	Human	+Cofactors	100.00	15.67	4.40	1.92	1.18
		-Cofactors	100.00	-	-	-	101.55
Gefitinib (**1**)	Human	+Cofactors	100.00	39.75	19.60	12.23	8.14
		-Cofactors	100.00	-	-	-	100.00
**6**	Human	+Cofactors	100.00	49.30	28.74	20.85	14.00
		-Cofactors	100.00	-	-	-	89.19
**7**	Human	+Cofactors	100.00	43.79	21.93	10.84	7.20
		-Cofactors	100.00	-	-	-	87.34
**8**	Human	+Cofactors	100.00	60.04	39.49	24.83	20.34
		-Cofactors	100.00	-	-	-	95.02
**9**	Human	+Cofactors	100.00	69.65	46.26	31.49	26.65
		-Cofactors	100.00	-	-	-	109.86
Verapamil	Rat	+Cofactors	100.00	5.55	BLOD	BLOD	BLOD
		-Cofactors	100.00	-	-	-	113.69
Gefitinib (**1**)	Rat	+Cofactors	100.00	63.13	37.48	25.25	19.26
		-Cofactors	100.00	-	-	-	115.38
**6**	Rat	+Cofactors	100.00	50.62	25.81	17.98	11.88
		-Cofactors	100.00	-	-	-	109.68
**7**	Rat	+Cofactors	100.00	28.79	12.97	8.49	5.84
		-Cofactors	100.00	-	-	-	118.60
**8**	Rat	+Cofactors	100.00	58.02	32.28	22.82	15.90
		-Cofactors	100.00	-	-	-	97.68
**9**	Rat	+Cofactors	100.00	34.07	11.66	6.01	2.50
		-Cofactors	100.00	-	-	-	113.38

Abbreviations: BLOD, below level of detection.

Metabolic stability in hepatocytes

Table S11. Hepatocyte details used in the hepatocyte stability assay.

Item	Supplier
Human Hepatocytes, Mixed-Gender	BiolVT (Cat No. X008001, Lot No. QZW)
SD Rat Hepatocytes, Male	BiolVT (Cat. No M00005, Lot No. DVB)
Cynomolgus Monkey Hepatocytes, Male	RILD (Cat. No. HP-SXH-02M, Lot No. CJNC)
Beagle Dog Hepatocytes, Male	BiolVT (Cat. No. M00205, Lot No. UHC)

Table S12. Results for hepatocyte stability data in human, rat, monkey and dog hepatocytes.

Compound	Species	Remaining Percentages (%)					
		0.5 min	15 min	30 min	60 min	90 min	120 min
Verapamil	Human	100.00	63.73	36.84	16.90	9.97	4.28
Gefitinib (**1**)	Human	100.00	97.55	94.76	73.38	50.47	39.53
**7**	Human	100.00	96.13	87.34	76.27	61.58	45.68
**9**	Human	100.00	98.94	89.67	75.36	58.28	52.37
Verapamil	Rat	100.00	14.03	5.49	BLOD	BLOD	BLOD
Gefitinib (**1**)	Rat	100.00	66.86	59.92	39.44	21.69	13.94
**7**	Rat	100.00	38.18	15.61	2.48	0.92	0.74
**9**	Rat	100.00	47.58	28.51	11.71	4.98	2.53
Verapamil	Monkey	100.00	13.77	BLOD	BLOD	BLOD	BLOD
**9**	Monkey	100.00	49.08	30.35	13.17	4.27	BLOD
Verapamil	Dog	100.00	89.23	72.58	51.08	29.98	18.67
**9**	Dog	100.00	88.01	79.61	68.74	60.01	41.31

Abbreviations: BLOD, below level of detection.

Plasma stability

Table S13. Plasma details used in the plasma stability assay.

Item	Supplier
Human Plasma with EDTA-K2, Mixed Gender	Pharmaron (Lot No. H-BLO-20220620-01-C, H-BLO-20220718-01-C)
Rat Plasma, Mixed Gender	IPHASE or BIOLVT (Lot No. R220116, RAT510985)

Table S14. Results for plasma stability in human and rat plasma assay.

Compound	Species	Remaining Percentages (%)					$t_{1/2}$ (min)
		0 min	15 min	30 min	60 min	120 min	
Propantheline	Human	100.00	69.10	48.79	22.07	4.85	27.55
Gefitinib (**1**)	Human	100.00	103.40	103.92	101.33	104.45	∞
**6**	Human	100.00	98.26	102.87	101.74	97.90	∞
**7**	Human	100.00	100.68	93.61	100.91	97.91	∞
**9**	Human	100.00	93.43	92.26	97.47	92.38	∞
Mevinolin	Rat	100.00	1.54	0.42	0.30	0.27	2.49
Gefitinib (**1**)	Rat	100.00	100.11	98.28	95.04	93.66	∞
**6**	Rat	100.00	100.23	99.35	99.28	104.21	∞
**7**	Rat	100.00	91.34	93.84	93.29	95.67	∞
**9**	Rat	100.00	96.53	95.56	99.37	96.60	∞

Abbreviations: $t_{1/2}$, half-life; ∞, compound did not show any degradation.

Caco-2 permeability

Table S15. Caco-2 cellular details used in the Caco-2 cellular permeability assay.

Item	Supplier
Caco-2 cells	ATCC (ATCC No. HTB-37)
HTS Transwell 96 Well	Corning (Cat No. 3391)

Table S16. Results for Caco-2 cellular permeability.

Compound	$P_{app(a-b)}$ (10^{-6}, cm/s)	$P_{app(b-a)}$ (10^{-6}, cm/s)	Efflux Ratio	Recovery% (AP-BL)	Recovery% (BL-AP)
Metoprolol	24.90	22.21	0.89	97.43	98.37
Digoxin	0.27	13.43	50.12	108.16	96.15
Gefitinib (**1**)	0.39	3.70	9.49	30.20	61.50
**6**	5.59	10.69	1.91	53.78	60.32
**7**	10.88	10.84	1.00	72.91	72.22
**8**	0.63	12.10	19.34	63.34	72.31
**9**	6.52	8.61	1.32	53.85	73.49

Abbreviations: P_{app}, apparent permeability; AP, apical; BL, basolateral.

MDCKII-MDR1 Permeability

Table S17. MDCKII-MDR1 cellular details used in the MDCKII-MDR1 permeability assay.

**Item**	**Supplier**
MDCKII-MDR1 cells	Netherlands Cancer Institute (Amsterdam)
HTS Transwell 96 Well	Corning (Cat No. 3391)

Table S18. Results for MDCKII-MDR1 cellular permeability.

**Compound**	**$P_{app(a-b)}$ (10^{-6}, cm/s)**	**$P_{app(b-a)}$ (10^{-6}, cm/s)**	**Efflux Ratio**	**Recovery% (AP-BL)**	**Recovery% (BL-AP)**
Metoprolol	25.87	20.90	0.81	105.41	98.39
Digoxin	0.25	10.22	40.61	98.78	100.10
Gefitinib (**1**)	10.8	3.71	3.45	33.84	65.10
**6**	4.19	8.26	1.99	51.59	66.44
**7**	12.56	13.29	1.06	73.50	76.43
**8**	1.35	11.69	8.65	58.59	76.14
**9**	9.82	9.06	0.92	68.56	89.96

Abbreviations: P_{app}, apparent permeability; AP, apical; BL, basolateral.

hERG Safety evaluation by manual patch-clamp system

Table S19. HEK293 cellular details used in hERG safety evaluation.

Item	Supplier
HEK 293 Cell Line	Invitrogen (Cat No. K1236)
TrypLE^{TM} Express	Gibco (Cat No. 12604)
Dofetilide	TRC (Cat No. D525700)

Table S20. hERG safety evaluation results.

Compound	hERG IC_{50} (μM)[a]
Dofetilide[b]	0.015 ± 0.0008
Gefitinib (**1**)	2.07 ± 0.5
**7**	8.48 ± 3.5
**9**	6.48 ± 1.3

[a]IC_{50} values are presented as the mean ± SEM. [b]Dofetilide tested at 5 concentrations (0.00185, 0.00556, 0.01667, 0.05000, 0.15000 μM) and run in triplicate.

CYP Inhibition of CYP1A2, CYP2C9, CYP2C19, CYP2D6 and CYP3A4 in human liver microsomes

Table S21. CYP450 control compound details used in the CYP inhibition assay.

CYP Isoform	Positive Inhibitors	Final Concentration (µM)
CYP1A2	Furafylline	0, 0.0075, 0.025, 0.075, 0.25, 0.75, 2.5, 7.5, 25
CYP2C9	Sulfaphenazole	0, 0.0015, 0.005, 0.015, 0.05, 0.15, 0.5, 1.5, 5
CYP2C19	Tranylcypromine	0, 0.0075, 0.025, 0.075, 0.25, 0.75, 2.5, 7.5, 25
CYP2D6	Quinidine	0, 0.0015, 0.005, 0.015, 0.05, 0.15, 0.5, 1.5, 5
CYP3A4	Ketoconazole	0, 0.0015, 0.005, 0.015, 0.05, 0.15, 0.5, 1.5, 5

Table S22. CYP450 substrate concentration details.

CYP Isoform	Substrate	Working Concentration (µM)	Final Concentration (µM)	Incubation Time
CYP1A2	Phenacetin	800	40	20 min
CYP2C9	Diclofenac	120	6	5 min
CYP2C19	Mephenytoin	1000	50	20 min
CYP2D6	Dextromethorphan	40	2	20 min
CYP3A4	Midazolam	20	1	5 min

Table S23. Results for CYP450 inhibition assay.

Compound	IC$_{50}$ (µM)[a]				
	CYP1A2	CYP2C9	CYP2C19	CYP2D6	CYP3A4-M
Furafylline	2.27	-	-	-	-
Sulfaphenazole	-	0.23	-	-	-
Tranylcypromine	-	-	7.96	-	-
Quinidine	-	-	-	0.030	-
Ketoconazole	-	-	-	-	0.021
**9**	>30	>30	>30	1.11	>30

[a]IC$_{50}$ values are presented as the mean (n = 2 independent replicates).

Direct and time-dependent inhibition of CYP2D6 in human liver microsomes

Table S24. Microsome details used in direct and time-dependent CYP2D6 inhibition study.

Item	Supplier
Pooled Human Liver Microsomes, Mixed Gender	BD Gentest (Cat. No. 452117, Lot. No. 38297)

Table S25. CYP2D6 control compound concentration details.

CYP Isoform	Positive Control	Working Concentration (µM)	Final Concentration (µM)
CYP2D6	Paroxetine	0.006, 0.02, 0.06, 0.2, 0.6, 2	0.03, 0.1, 0.3, 1, 3, 10

Table S26. Final CYP2D6 substrate concentrations used in assay.

CYP Isoform	Substrate	Working Concentration (µM)	Final Concentration (µM)	Incubation Time
CYP2D6	Bufuralol	40	2	20 min

Table S27. Results for CYP2D6 time-dependent inhibition assay in human liver microsomes.

Compound	Pre-incubation	CYP2D6	
		IC_{50} (µM)[a]	Inhibition percentage(%) at Top conc.
Paroxetine	0 min	0.26	92.30
	30 min without NADPH	0.38	91.21
	30 min with NADPH	0.098	95.47
**9**	0 min	1.05	78.15
	30 min without NADPH	1.16	78.78
	30 min with NADPH	1.82	74.73

[a]IC_{50} values are presented as the mean (*n* = 2 independent replicates).

Direct and time-dependent inhibition of CYP2D6 in primary human hepatocytes

Table S28. Hepatocyte details used in direct and time-dependent CYP2D6 inhibition study.

Item	Supplier
Human Hepatocytes, Mixed Gender	BiolVT (Cat. No. X008001, Lot No. AUY)

Table S29. CYP2D6 control compound concentration details.

CYP Isoform	Positive Control	Final Concentration (µM)
CYP2D6	Paroxetine	0.01, 0.03, 0.1, 0.3, 1, 3, 10

Table S30. CYP2D6 isoform substrate concentration details.

CYP Isoform	Substrate	Working Concentration (mM)	Final Concentration (µM)	Incubation Time
CYP2D6	Dextromethorphan	20	40	5 min

Table S31. Results for CYP2D6 time-dependent inhibition assay in primary human hepatocytes.

Compound	Pre-incubation	CYP2D6	
		IC_{50} (µM)[a]	Inhibition percentage(%) at Top conc.
Paroxetine	0 min	0.39	94.56
	30 min	0.04	98.16
**9**	0 min	4.57	63.41
	30min	4.75	66.54

[a]IC_{50} values are presented as the mean (*n* = 2 independent replicates).

AMES fluctuation test

Table S32. *Salmonella* strains used in the AMES fluctuation assay.

Salmonella Strain	DNA Target	His Mutation	Reversion Event
TA98	CGCGCGCG	hisD3052	Frameshifts
TA100	GGG	hisG46	Base-pair substitution
TA1535	GGG	hisG46	Base-pair substitution
TA1537	Near CCC run	hisC3076	Frameshifts

Table S33. Results for AMES fluctuation assay of **9**.

Compound and Test	Test Concentration	Count (# of wells)	Positive Significance (- to +++)[a]	Fisher Exact Test (p-value)
**AMES Test (TA98-S9)**				
2-Aminoanthracene	5.0E-06 M	1	-	1.0000
9-Aminoacridine	1.0E-05 M	0	-	0.5000
Quercetin	1.0E-05 M	12	+++	0.0009
Streptozotocin	2.5E-06 M	1	-	1.0000
**9**	5.0E-06 M	0	-	0.5000
**9**	1.0E-05 M	0	-	0.5000
**9**	5.0E-05 M	2	-	0.5000
**9**	1.0E-04 M	0	-	0.5000
**AMES Test (TA98+S9)**				
2-Aminoanthracene	2.0E-06 M	47	+++	0.0000
9-Aminoacridine	1.0E-05 M	1	-	0.5000
Quercetin	1.0E-05 M	30	+++	0.0000
Streptozotocin	2.5E-06 M	2	-	0.2474
**9**	5.0E-06 M	0	-	1.0000
**9**	1.0E-05 M	2	-	0.2474
**9**	5.0E-05 M	1	-	0.5000
**9**	1.0E-04 M	0	-	1.0000
**AMES Test (TA100-S9)**				
2-Aminoanthracene	5.0E-06 M	4	-	0.1808

9-Aminoacridine	1.0E-05 M	2	-	0.5000
Quercetin	1.0E-05 M	1	+++	1.0000
Streptozotocin	2.5E-06 M	42	+++	0.0000
**9**	5.0E-06 M	1	-	1.0000
**9**	1.0E-05 M	4	-	0.1808
**9**	5.0E-05 M	0	-	0.5000
**9**	1.0E-04 M	0	-	0.5000
**AMES Test (TA100+S9)**				
2-Aminoanthracene	1.0E-06 M	21	+++	0.0000
9-Aminoacridine	1.0E-05 M	3	-	1.0000
Quercetin	1.0E-05 M	5	-	0.3572
Streptozotocin	2.5E-06 M	39	+++	0.0000
**9**	5.0E-06 M	3	-	1.0000
**9**	1.0E-05 M	3	-	1.0000
**9**	5.0E-05 M	0	-	0.1211
**9**	1.0E-04 M	0	-	0.1211
**AMES Test (TA1535-S9)**				
2-Aminoanthracene	2.0E-06 M	47	+++	0.0000
9-Aminoacridine	1.0E-05 M	1	-	0.5000
Quercetin	1.0E-05 M	30	+++	0.0000
Streptozotocin	2.5E-06 M	2	-	0.2474
**9**	5.0E-06 M	0	-	1.0000
**9**	1.0E-05 M	0	-	1.0000
**9**	5.0E-05 M	0	-	1.0000
**9**	1.0E-04 M	0	-	1.0000
**AMES Test (TA1535+S9)**				
2-Aminoanthracene	5.0E-06 M	15	+++	0.0001
9-Aminoacridine	1.0E-05 M	0	-	0.5000
Quercetin	1.0E-05 M	4	-	0.1808
Streptozotocin	2.5E-06 M	48	+++	0.0000
**9**	5.0E-06 M	1	-	1.0000
**9**	1.0E-05 M	3	-	0.3085
**9**	5.0E-05 M	0	-	0.5000
**9**	1.0E-04 M	0	-	0.5000

**AMES Test (TA1537-S9)**				
2-Aminoanthracene	5.0E-06 M	1	-	1.0000
9-Aminoacridine	1.0E-05 M	11	++	0.0018
Quercetin	1.0E-05 M	1	-	1.0000
Streptozotocin	2.5E-06 M	2	-	0.5000
**9**	5.0E-06 M	1	-	1.0000
**9**	1.0E-05 M	0	-	0.5000
**9**	5.0E-05 M	0	-	0.5000
**9**	1.0E-04 M	0	-	0.5000
**AMES Test (TA1537+S9)**				
2-Aminoanthracene	1.0E-05 M	14	++	0.0031
9-Aminoacridine	1.0E-05 M	17	+++	0.0004
Quercetin	3.0E-05 M	6	-	0.2430
Streptozotocin	2.5E-06 M	7	-	0.1584
**9**	5.0E-06 M	3	-	1.0000
**9**	1.0E-05 M	5	-	0.3572
**9**	5.0E-05 M	4	-	0.5000
**9**	1.0E-04 M	1	-	0.3085

[a]Significance of the positive counts between treatment and control were calculated using a one-tailed Fisher's exact test. Significance levels are reported as follows: weak positive, if $P < 0.05$ (denoted as "+"); strong positive, if $P < 0.01$ (denoted as "++"); very strong positive, if $P < 0.001$ (denoted as "++"). Hyphens (-) indicate negative results.

Table S34. Results for background bacterial cytotoxicity assay of **9**.

Compound and Test	Test Concentration	Mean % Control (n = 3)	Cytotoxicity (% of control)[a]	Flag[b]
**Bacterial Cytotoxicity (TA98-S9)**				
**9**	6.0E-07 M	89.0	89	-
**9**	1.2E-06 M	92.3	92	-
**9**	2.5E-06 M	91.7	92	-
**9**	5.0E-06 M	95.7	96	-
**9**	1.0E-05 M	91.1	91	-
**9**	2.5E-05 M	94.8	95	-
**9**	5.0E-05 M	87.8	88	-
**9**	1.0E-04 M	84.2	84	-
**Bacterial Cytotoxicity (TA100-S9)**				
**9**	6.0E-07 M	91.6	92	-
**9**	1.2E-06 M	91.2	91	-
**9**	2.5E-06 M	89.5	90	-
**9**	5.0E-06 M	93.2	93	-
**9**	1.0E-05 M	90.3	90	-
**9**	2.5E-05 M	80.9	81	-
**9**	5.0E-05 M	67.3	67	-
**9**	1.0E-04 M	34.0	34	Cytox.
**Bacterial Cytotoxicity (TA1535-S9)**				
**9**	6.0E-07 M	98.9	99	-
**9**	1.2E-06 M	120.4	120	-
**9**	2.5E-06 M	101.2	101	-
**9**	5.0E-06 M	108.1	108	-
**9**	1.0E-05 M	99.4	99	-
**9**	2.5E-05 M	97.6	98	-
**9**	5.0E-05 M	85.7	86	-
**9**	1.0E-04 M	78.4	78	-
**Bacterial Cytotoxicity (TA1537-S9)**				
**9**	6.0E-07 M	101.7	102	-
**9**	1.2E-06 M	98.4	98	-
**9**	2.5E-06 M	90.5	91	-
**9**	5.0E-06 M	98.8	99	-
**9**	1.0E-05 M	88.7	88	-

**9**	2.5E-05 M	83.8	84	-
**9**	5.0E-05 M	71.1	71	-
**9**	1.0E-04 M	60.3	60	-

[a]Cytotoxicity is presented as % of control growth. [b]A cytotoxicity value of less than 60% is flagged and the compound is considered as toxic (denoted as Cytox.) at the respective concentration. A hyphen (-) denotes no cytotoxicity at the respective concentration.

Micronucleus test

Table S35. Results for *in vitro* micronucleus test of **9**.

Compound and Test	Test Concentration	Scored Cells	%Cytotoxicity CBPI Index	%Cytotoxicity Cell Numbers	%Micronucleated Cells	*P*-value[a]	Result	Flag
**Micronucleus (CHO+S9, HCA)**								
Control + S9	0 M	4193	3.3	37.6	0.43	N/A	N/A	-
Cyclophosphamide	7.2 E-05 M	1700	10.7	62.5	1.65	0.0023	+	-
**9**	8.0E-06 M	2166	-10.3	4.6	0.46	0.4482	-	-
**9**	1.6E-05 M	2086	-3.5	30.5	0.48	0.4104	-	-
**9**	3.1E-05 M	1623	13.0	71.3	0.12	0.1072	-	-
**9**	6.2E-05 M	N/A	N/A	N/A	N/A	N/A	N/A	Cytox.
**9**	1.3E-04 M	N/A	N/A	N/A	N/A	N/A	N/A	Cytox.
**9**	2.5E-04 M	N/A	N/A	N/A	N/A	N/A	N/A	Cytox.
**9**	5.0E-04 M	N/A	N/A	N/A	N/A	N/A	N/A	Cytox.
**9**	1.0E-03 M	N/A	N/A	N/A	N/A	N/A	N/A	Cytox.
**Micronucleus (CHO-S9, HCA)**								
Control - S9	0 M	4051	0.1	0.0	0.42	N/A	N/A	-
Mitomycin C	3.0E-07 M	2178	8.2	50.5	1.46	0.0009	+	-
**9**	8.0E-06 M	2078	12.6	6.6	0.58	0.1685	-	-
**9**	1.6E-05 M	N/A	N/A	N/A	N/A	N/A	N/A	Cytox.
**9**	3.1E-05 M	N/A	N/A	N/A	N/A	N/A	N/A	Cytox.
**9**	6.2E-05 M	N/A	N/A	N/A	N/A	N/A	N/A	Cytox.
**9**	1.3E-04 M	N/A	N/A	N/A	N/A	N/A	N/A	Cytox.
**9**	2.5E-04 M	N/A	N/A	N/A	N/A	N/A	N/A	Cytox.
**9**	5.0E-04 M	N/A	N/A	N/A	N/A	N/A	N/A	Cytox.
**9**	1.0E-03 M	N/A	N/A	N/A	N/A	N/A	N/A	Cytox.

[a]Significance of the positive counts between treatment and control were calculated using a one-tailed t-test with two sample equal variance. Significance levels are reported as follows: "+" if $P < 0.05$ by t-test and % of micronucleated cells at least 3-fold higher than background levels. "+/-" if $P < 0.05$ by t-test and % of micronucleated cells at least 2-fold higher than background levels. "-" if $P > 0.05$ by t-test and % of micronucleated cells less than 2-fold higher than background levels. Hyphens (-) indicate negative results. Cytotoxicity (denoted as Cytox.) refers to high cytotoxicity resulting in an insufficient number of scorable cells (> 80% cytotoxicity).

In vitro CTG assay

Table S36. Materials used in cell viability assays.

Item	Supplier
RPMI 1640	Invitrogen (Cat No. C22400500BT)
DMEM	Life Technologies (Cat No. C11995500BT)
L-15	Gibco (Cat No. 11415064)
BEGM BulletKit	Lonza (Cat No. CC-3171 & CC-4175)
FBS	ExCell Bio (Cat No. FND500)
McCoy's 5a	Invitrogen (Cat No. 16600082)
96-Well Polystyrene Microplates	Corning (Cat No. 3610)
CellTiter-Glo® Luminescent Cell Viability Assay	Promega (Cat No. G7572)
Backseal Black Adhesive Bottom Seal	Perkin Elmer (Cat No. 6005189)
Cisplatin	Qilu Pharma (Batch No. 6J015A89)

Table S37. Cell line information for cell viability assays.

Cell Line Name	Tissue Origin	Culture Property	Cell Culture Medium[a]	Compound Treatment Time	Seeding Density
HCC827	Lung	Adherent	RPMI1640+10%FBS	72 h	2000-4000
NCI-H1975	Lung	Adherent	RPMI1640+10%FBS	72 h	1500
NCI-H3255	Lung	Adherent	BEGM+10%FBS	72 h	3000
A 431	Skin	Adherent	DMEM+10%FBS	72 h	3000-3500
Ba/F3 del E746_A750/C797S	B lymphocyte	Suspension	RPMI1640+10%FBS	72 h	5000
Ba/F3 L858R/C797S	B lymphocyte	Suspension	RPMI1640+10%FBS	72 h	5000
SK-BR-3	Breast	Adherent	McCoy's 5a+10% FBS	72 h	4000
ZR-75-30	Breast	Adherent	RPMI1640+20% FBS	72h	7000
AU565	Breast	Adherent	RPMI1640+10%FBS	72h	6000

[a]Cells were cultured in a 37 °C incubator with 5% CO_2.

KINOMEScan

Table S38. Full KINOMEScan results for **9** at a single concentration of 1 μM.

DiscoveRx Gene Symbol	Entrez Gene Symbol	Percent Control
AAK1	AAK1	92
ABL1(E255K)-phosphorylated	ABL1	6.4
ABL1(F317I)-nonphosphorylated	ABL1	54
ABL1(F317I)-phosphorylated	ABL1	48
ABL1(F317L)-nonphosphorylated	ABL1	16
ABL1(F317L)-phosphorylated	ABL1	4
ABL1(H396P)-nonphosphorylated	ABL1	1
ABL1(H396P)-phosphorylated	ABL1	6.6
ABL1(M351T)-phosphorylated	ABL1	12
ABL1(Q252H)-nonphosphorylated	ABL1	6.7
ABL1(Q252H)-phosphorylated	ABL1	8.8
ABL1(T315I)-nonphosphorylated	ABL1	68
ABL1(T315I)-phosphorylated	ABL1	30
ABL1(Y253F)-phosphorylated	ABL1	6.6
ABL1-nonphosphorylated	ABL1	8.9
ABL1-phosphorylated	ABL1	7.7
ABL2	ABL2	64
ACVR1	ACVR1	100
ACVR1B	ACVR1B	86

ACVR2A	ACVR2A	100
ACVR2B	ACVR2B	100
ACVRL1	ACVRL1	100
ADCK3	CABC1	90
ADCK4	ADCK4	79
AKT1	AKT1	100
AKT2	AKT2	91
AKT3	AKT3	68
ALK	ALK	89
ALK(C1156Y)	ALK	100
ALK(L1196M)	ALK	100
AMPK-alpha1	PRKAA1	100
AMPK-alpha2	PRKAA2	97
ANKK1	ANKK1	97
ARK5	NUAK1	100
ASK1	MAP3K5	100
ASK2	MAP3K6	85
AURKA	AURKA	75
AURKB	AURKB	84
AURKC	AURKC	82
AXL	AXL	100
BIKE	BMP2K	99
BLK	BLK	16
BMPR1A	BMPR1A	90
BMPR1B	BMPR1B	79
BMPR2	BMPR2	99
BMX	BMX	93
BRAF	BRAF	89
BRAF(V600E)	BRAF	82
BRK	PTK6	94
BRSK1	BRSK1	100
BRSK2	BRSK2	74

BTK	BTK	100
BUB1	BUB1	81
CAMK1	CAMK1	100
CAMK1B	PNCK	82
CAMK1D	CAMK1D	100
CAMK1G	CAMK1G	89
CAMK2A	CAMK2A	88
CAMK2B	CAMK2B	87
CAMK2D	CAMK2D	91
CAMK2G	CAMK2G	97
CAMK4	CAMK4	100
CAMKK1	CAMKK1	94
CAMKK2	CAMKK2	85
CASK	CASK	72
CDC2L1	CDK11B	100
CDC2L2	CDC2L2	96
CDC2L5	CDK13	100
CDK11	CDK19	67
CDK2	CDK2	100
CDK3	CDK3	100
CDK4	CDK4	85
CDK4-cyclinD1	CDK4	100
CDK4-cyclinD3	CDK4	87
CDK5	CDK5	100
CDK7	CDK7	70
CDK8	CDK8	96
CDK9	CDK9	100
CDKL1	CDKL1	64
CDKL2	CDKL2	98
CDKL3	CDKL3	46
CDKL5	CDKL5	100
CHEK1	CHEK1	100

CHEK2	CHEK2	98
CIT	CIT	76
CLK1	CLK1	88
CLK2	CLK2	90
CLK3	CLK3	100
CLK4	CLK4	85
CSF1R	CSF1R	86
CSF1R-autoinhibited	CSF1R	82
CSK	CSK	100
CSNK1A1	CSNK1A1	73
CSNK1A1L	CSNK1A1L	100
CSNK1D	CSNK1D	95
CSNK1E	CSNK1E	68
CSNK1G1	CSNK1G1	92
CSNK1G2	CSNK1G2	93
CSNK1G3	CSNK1G3	100
CSNK2A1	CSNK2A1	76
CSNK2A2	CSNK2A2	46
CTK	MATK	85
DAPK1	DAPK1	97
DAPK2	DAPK2	90
DAPK3	DAPK3	90
DCAMKL1	DCLK1	92
DCAMKL2	DCLK2	91
DCAMKL3	DCLK3	100
DDR1	DDR1	37
DDR2	DDR2	77
DLK	MAP3K12	71
DMPK	DMPK	100
DMPK2	CDC42BPG	100
DRAK1	STK17A	8.3
DRAK2	STK17B	96

DYRK1A	DYRK1A	92
DYRK1B	DYRK1B	66
DYRK2	DYRK2	96
EGFR	EGFR	0.3
EGFR(E746-A750del)	EGFR	12
EGFR(G719C)	EGFR	0.05
EGFR(G719S)	EGFR	0.15
EGFR(L747-E749del, A750P)	EGFR	7.8
EGFR(L747-S752del, P753S)	EGFR	14
EGFR(L747-T751del,Sins)	EGFR	0.25
EGFR(L858R)	EGFR	0.95
EGFR(L858R,T790M)	EGFR	10
EGFR(L861Q)	EGFR	0.1
EGFR(S752-I759del)	EGFR	0.1
EGFR(T790M)	EGFR	44
EIF2AK1	EIF2AK1	100
EPHA1	EPHA1	47
EPHA2	EPHA2	91
EPHA3	EPHA3	99
EPHA4	EPHA4	71
EPHA5	EPHA5	93
EPHA6	EPHA6	14
EPHA7	EPHA7	100
EPHA8	EPHA8	95
EPHB1	EPHB1	100
EPHB2	EPHB2	72
EPHB3	EPHB3	100
EPHB4	EPHB4	67
EPHB6	EPHB6	95
ERBB2	ERBB2	0.45

ERBB3	ERBB3	55
ERBB4	ERBB4	24
ERK1	MAPK3	96
ERK2	MAPK1	100
ERK3	MAPK6	88
ERK4	MAPK4	91
ERK5	MAPK7	100
ERK8	MAPK15	96
ERN 1.00	ERN 1.00	96
FAK	PTK2	100
FER	FER	97
FES	FES	98
FGFR1	FGFR1	71
FGFR2	FGFR2	98
FGFR3	FGFR3	94
FGFR3(G697C)	FGFR3	80
FGFR4	FGFR4	100
FGR	FGR	63
FLT1	FLT1	100
FLT3	FLT3	100
FLT3(D835H)	FLT3	54
FLT3(D835V)	FLT3	20
FLT3(D835Y)	FLT3	14
FLT3(ITD)	FLT3	84
FLT3(ITD,D835V)	FLT3	21
FLT3(ITD,F691L)	FLT3	13
FLT3(K663Q)	FLT3	100
FLT3(N841I)	FLT3	70
FLT3(R834Q)	FLT3	89
FLT3-autoinhibited	FLT3	99
FLT4	FLT4	97
FRK	FRK	54

FYN	FYN	78
GAK	GAK	34
GCN2(Kin.Dom.2,S808G)	EIF2AK4	47
GRK1	GRK1	59
GRK2	ADRBK1	97
GRK3	ADRBK2	72
GRK4	GRK4	100
GRK7	GRK7	96
GSK3A	GSK3A	94
GSK3B	GSK3B	91
HASPIN	GSG2	100
HCK	HCK	42
HIPK1	HIPK1	90
HIPK2	HIPK2	100
HIPK3	HIPK3	93
HIPK4	HIPK4	100
HPK1	MAP4K1	81
HUNK	HUNK	100
ICK	ICK	94
IGF1R	IGF1R	95
IKK-alpha	CHUK	88
IKK-beta	IKBKB	97
IKK-epsilon	IKBKE	86
INSR	INSR	64
INSRR	INSRR	94
IRAK1	IRAK1	64
IRAK3	IRAK3	100
IRAK4	IRAK4	75
ITK	ITK	100
JAK1(JH1domain-catalytic)	JAK1	100
JAK1(JH2domain-pseudokinase)	JAK1	93

JAK2(JH1domain-catalytic)	JAK2	100
JAK3(JH1domain-catalytic)	JAK3	79
JNK1	MAPK8	81
JNK2	MAPK9	81
JNK3	MAPK10	86
KIT	KIT	100
KIT(A829P)	KIT	100
KIT(D816H)	KIT	52
KIT(D816V)	KIT	48
KIT(L576P)	KIT	95
KIT(V559D)	KIT	100
KIT(V559D,T670I)	KIT	84
KIT(V559D,V654A)	KIT	98
KIT-autoinhibited	KIT	98
LATS1	LATS1	100
LATS2	LATS2	85
LCK	LCK	12
LIMK1	LIMK1	100
LIMK2	LIMK2	97
LKB1	STK11	67
LOK	STK10	61
LRRK2	LRRK2	100
LRRK2(G2019S)	LRRK2	95
LTK	LTK	100
LYN	LYN	7.4
LZK	MAP3K13	100
MAK	MAK	100
MAP3K1	MAP3K1	90
MAP3K15	MAP3K15	80
MAP3K2	MAP3K2	74
MAP3K3	MAP3K3	91

MAP3K4	MAP3K4	65
MAP4K2	MAP4K2	100
MAP4K3	MAP4K3	100
MAP4K4	MAP4K4	100
MAP4K5	MAP4K5	100
MAPKAPK2	MAPKAPK2	100
MAPKAPK5	MAPKAPK5	100
MARK1	MARK1	76
MARK2	MARK2	91
MARK3	MARK3	74
MARK4	MARK4	83
MAST1	MAST1	100
MEK1	MAP2K1	92
MEK2	MAP2K2	75
MEK3	MAP2K3	63
MEK4	MAP2K4	89
MEK5	MAP2K5	45
MEK6	MAP2K6	100
MELK	MELK	100
MERTK	MERTK	97
MET	MET	90
MET(M1250T)	MET	91
MET(Y1235D)	MET	100
MINK	MINK1	62
MKK7	MAP2K7	79
MKNK1	MKNK1	100
MKNK2	MKNK2	82
MLCK	MYLK3	100
MLK1	MAP3K9	100
MLK2	MAP3K10	78
MLK3	MAP3K11	99
MRCKA	CDC42BPA	86

MRCKB	CDC42BPB	100
MST1	STK4	100
MST1R	MST1R	100
MST2	STK3	92
MST3	STK24	78
MST4	MST4	78
MTOR	MTOR	96
MUSK	MUSK	100
MYLK	MYLK	85
MYLK2	MYLK2	84
MYLK4	MYLK4	90
MYO3A	MYO3A	100
MYO3B	MYO3B	100
NDR1	STK38	83
NDR2	STK38L	74
NEK1	NEK1	100
NEK10	NEK10	100
NEK11	NEK11	84
NEK2	NEK2	92
NEK3	NEK3	56
NEK4	NEK4	76
NEK5	NEK5	98
NEK6	NEK6	98
NEK7	NEK7	96
NEK9	NEK9	100
NIK	MAP3K14	89
NIM1	MGC42105	82
NLK	NLK	100
OSR1	OXSR1	91
p38-alpha	MAPK14	92
p38-beta	MAPK11	86
p38-delta	MAPK13	100

p38-gamma	MAPK12	93
PAK1	PAK1	100
PAK2	PAK2	100
PAK3	PAK3	54
PAK4	PAK4	100
PAK6	PAK6	100
PAK7	PAK7	96
PCTK1	CDK16	97
PCTK2	CDK17	100
PCTK3	CDK18	100
PDGFRA	PDGFRA	80
PDGFRB	PDGFRB	74
PDPK1	PDPK1	96
PFCDPK1(P.falciparum)	CDPK1	90
PFPK5(P.falciparum)	MAL13P1.279	95
PFTAIRE2	CDK15	94
PFTK1	CDK14	96
PHKG1	PHKG1	96
PHKG2	PHKG2	79
PIK3C2B	PIK3C2B	54
PIK3C2G	PIK3C2G	70
PIK3CA	PIK3CA	100
PIK3CA(C420R)	PIK3CA	85
PIK3CA(E542K)	PIK3CA	100
PIK3CA(E545A)	PIK3CA	88
PIK3CA(E545K)	PIK3CA	51
PIK3CA(H1047L)	PIK3CA	100
PIK3CA(H1047Y)	PIK3CA	79
PIK3CA(I800L)	PIK3CA	68
PIK3CA(M1043I)	PIK3CA	88
PIK3CA(Q546K)	PIK3CA	83
PIK3CB	PIK3CB	100

PIK3CD	PIK3CD	70
PIK3CG	PIK3CG	99
PIK4CB	PI4KB	91
PIKFYVE	PIKFYVE	1.6
PIM1	PIM1	100
PIM2	PIM2	54
PIM3	PIM3	99
PIP5K1A	PIP5K1A	94
PIP5K1C	PIP5K1C	79
PIP5K2B	PIP4K2B	100
PIP5K2C	PIP4K2C	68
PKAC-alpha	PRKACA	100
PKAC-beta	PRKACB	100
PKMYT1	PKMYT1	75
PKN1	PKN1	95
PKN2	PKN2	66
PKNB(M.tuberculosis)	pknB	94
PLK1	PLK1	71
PLK2	PLK2	100
PLK3	PLK3	100
PLK4	PLK4	100
PRKCD	PRKCD	85
PRKCE	PRKCE	96
PRKCH	PRKCH	83
PRKCI	PRKCI	89
PRKCQ	PRKCQ	100
PRKD1	PRKD1	100
PRKD2	PRKD2	90
PRKD3	PRKD3	100
PRKG1	PRKG1	95
PRKG2	PRKG2	100
PRKR	EIF2AK2	99

PRKX	PRKX	92
PRP4	PRPF4B	95
PYK2	PTK2B	99
QSK	KIAA0999	70
RAF1	RAF1	95
RET	RET	59
RET(M918T)	RET	72
RET(V804L)	RET	99
RET(V804M)	RET	73
RIOK1	RIOK1	73
RIOK2	RIOK2	90
RIOK3	RIOK3	67
RIPK1	RIPK1	100
RIPK2	RIPK2	72
RIPK4	RIPK4	68
RIPK5	DSTYK	100
ROCK1	ROCK1	72
ROCK2	ROCK2	79
ROS1	ROS1	95
RPS6KA4(Kin.Dom.1-N-terminal)	RPS6KA4	100
RPS6KA4(Kin.Dom.2-C-terminal)	RPS6KA4	98
RPS6KA5(Kin.Dom.1-N-terminal)	RPS6KA5	90
RPS6KA5(Kin.Dom.2-C-terminal)	RPS6KA5	100
RSK1(Kin.Dom.1-N-terminal)	RPS6KA1	84
RSK1(Kin.Dom.2-C-terminal)	RPS6KA1	100
RSK2(Kin.Dom.1-N-terminal)	RPS6KA3	73
RSK2(Kin.Dom.2-C-terminal)	RPS6KA3	99

RSK3(Kin.Dom.1-N-terminal)	RPS6KA2	100
RSK3(Kin.Dom.2-C-terminal)	RPS6KA2	70
RSK4(Kin.Dom.1-N-terminal)	RPS6KA6	67
RSK4(Kin.Dom.2-C-terminal)	RPS6KA6	70
S6K1	RPS6KB1	82
SBK1	SBK1	86
SGK	SGK1	78
SgK110	SgK110	86
SGK2	SGK2	100
SGK3	SGK3	76
SIK	SIK1	100
SIK2	SIK2	93
SLK	SLK	41
SNARK	NUAK2	78
SNRK	SNRK	96
SRC	SRC	33
SRMS	SRMS	87
SRPK1	SRPK1	99
SRPK2	SRPK2	94
SRPK3	SRPK3	100
STK16	STK16	100
STK33	STK33	95
STK35	STK35	100
STK36	STK36	60
STK39	STK39	95
SYK	SYK	68
TAK1	MAP3K7	97
TAOK1	TAOK1	100
TAOK2	TAOK2	85

TAOK3	TAOK3	100
TBK1	TBK1	90
TEC	TEC	100
TESK1	TESK1	88
TGFBR1	TGFBR1	76
TGFBR2	TGFBR2	100
TIE1	TIE1	82
TIE2	TEK	83
TLK1	TLK1	94
TLK2	TLK2	100
TNIK	TNIK	89
TNK1	TNK1	77
TNK2	TNK2	100
TNNI3K	TNNI3K	100
TRKA	NTRK1	100
TRKB	NTRK2	100
TRKC	NTRK3	100
TRPM6	TRPM6	84
TSSK1B	TSSK1B	100
TSSK3	TSSK3	100
TTK	TTK	100
TXK	TXK	85
TYK2(JH1domain-catalytic)	TYK2	100
TYK2(JH2domain-pseudokinase)	TYK2	86
TYRO3	TYRO3	86
ULK1	ULK1	65
ULK2	ULK2	100
ULK3	ULK3	62
VEGFR2	KDR	82
VPS34	PIK3C3	100
VRK2	VRK2	100

WEE1	WEE1	100
WEE2	WEE2	100
WNK1	WNK1	100
WNK2	WNK2	94
WNK3	WNK3	94
WNK4	WNK4	95
YANK1	STK32A	94
YANK2	STK32B	93
YANK3	STK32C	86
YES	YES1	59
YSK1	STK25	94
YSK4	MAP3K19	46
ZAK	ZAK	89
ZAP70	ZAP70	98

Selectivity score (S-scores) is a quantitative measure of compound selectivity and was calculated as previously described[1] by dividing the number of kinases that compounds bind to by the total number of distinct kinases tested, excluding mutant variants. S = Number of hits/ Number of assays. This value can be calculated using percent control as a potency threshold and provides a quantitative method of describing compound selectivity.[1] KINOMEScan individual data points are listed above and were used to calculate the following selectivity scores.

Table S39. Selectivity scores for **9** at a screening concentration of 1000 nM.

**Selectivity Score Type**	**Number of Hits**	**Number of Non-Mutant Kinases**	**Screening Concentration (nM)**	**Selectivity Score**
S(35)[a]	12	403	1000	0.03
S(10)[b]	6	403	1000	0.015
S(1)[c]	2	403	1000	0.005

[a]S(35) refers to: (the number of non-mutant kinases with percent control < 35) / (number of non-mutant kinases tested). [b]S(10) refers to: (the number of non-mutant kinases with percent control < 10) / (number of non-mutant kinases tested). [c]S(1) refers to: (the number of non-mutant kinases with percent control < 1) / (number of non-mutant kinases tested).

3. Supporting Chemistry Experimental Details

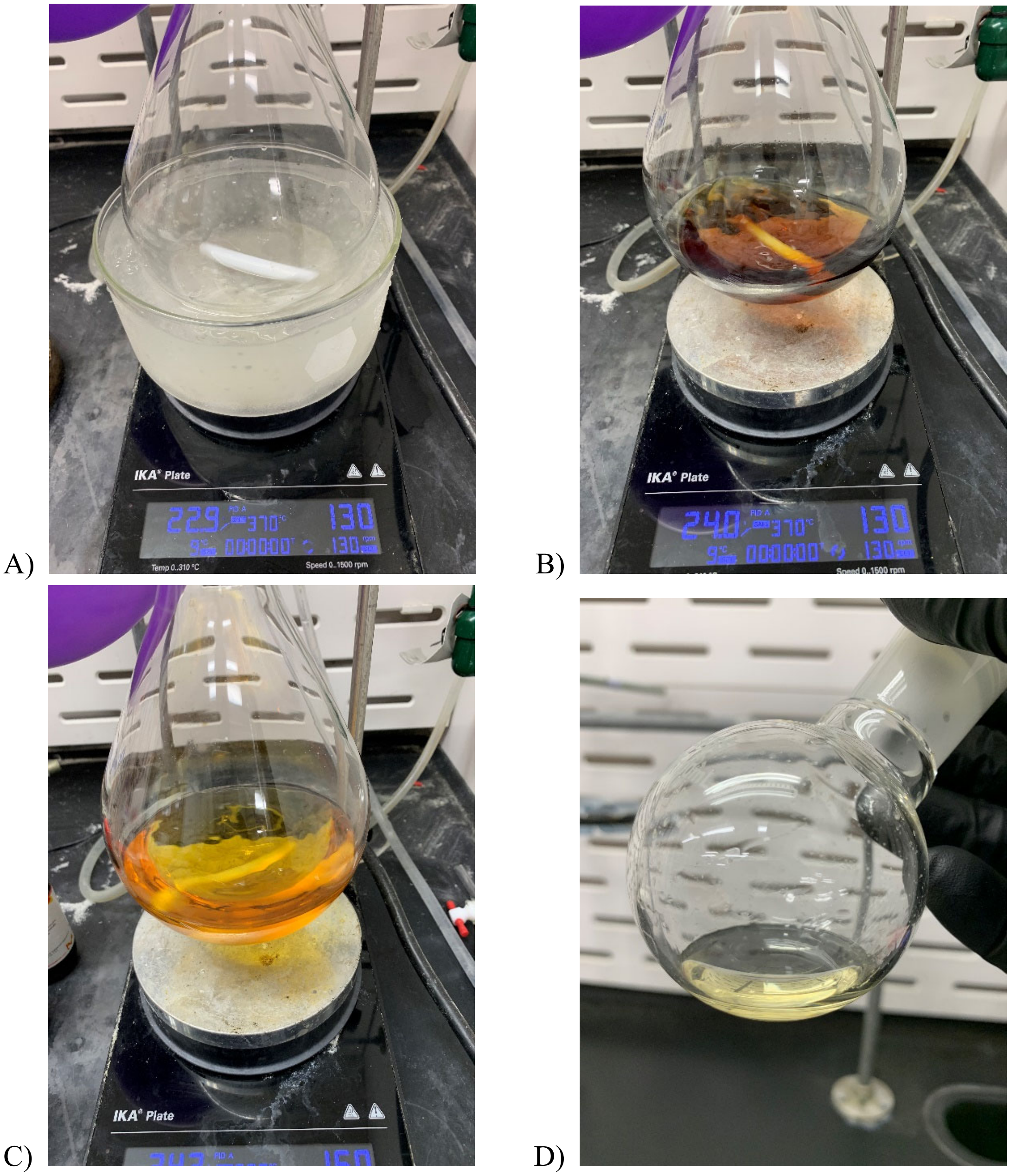

Figure S6. Gram-scale N-O bond formation reaction set-up. A) Morpholine (**12**) at 0 °C prior to addition of ^{i}PrMgCl·LiCl. B) Morpholine-derived magnesium amide, after addition of ^{i}PrMgCl·LiCl and stirring for 45 min. C) Reaction mixture after addition of **11** in anhydrous THF followed by stirring for 3 h. D) Isolated product **16** (4.95 g) after workup and purification by flash column chromatography on silica.

Solubility study between morpholine (12) derived magnesium amides

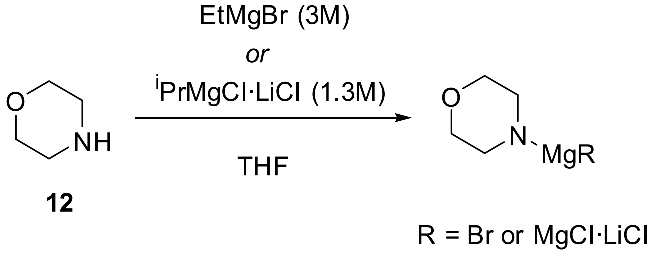

To a stirred solution of morpholine (**12**) (8.5 mL, 97.18 mmol, 1.2 eq.) in anhydrous THF (81 mL) at 0 °C was added EtMgBr (3M in THF) (27 mL, 80.98 mmol, 1.0 eq.) or ^{i}PrMgCl·LiCl (1.3M in THF) (67.5 mL, 80.98 mmol, 1.0 eq.) and the solution brought to r.t. and stirred for 45 min. Visual representation of the magnesium amides are depicted below at t = 0 and 45 min (Figure S7).

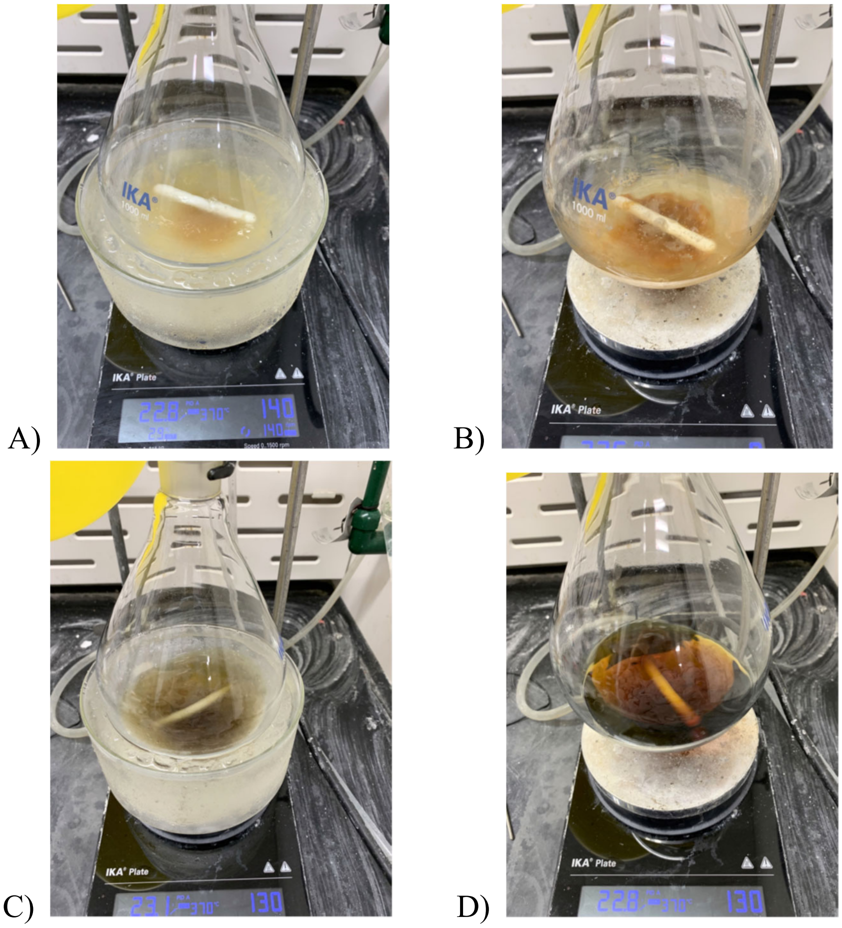

Figure S7. **Visual representation of solubility differences between multi-gram scale morpholine-derived magnesium amides**. A) Morpholine-derived magnesium amide at 0 °C, immediately after full addition of EtMgBr. B) Morpholine-derived magnesium amide, after addition of EtMgBr and stirring for 45 min. C) Morpholine-derived magnesium amide at 0 °C, immediately after full addition of ^{i}PrMgCl·LiCl. D) Morpholine-derived magnesium amide, after addition of ^{i}PrMgCl·LiCl and stirring for 45 min.

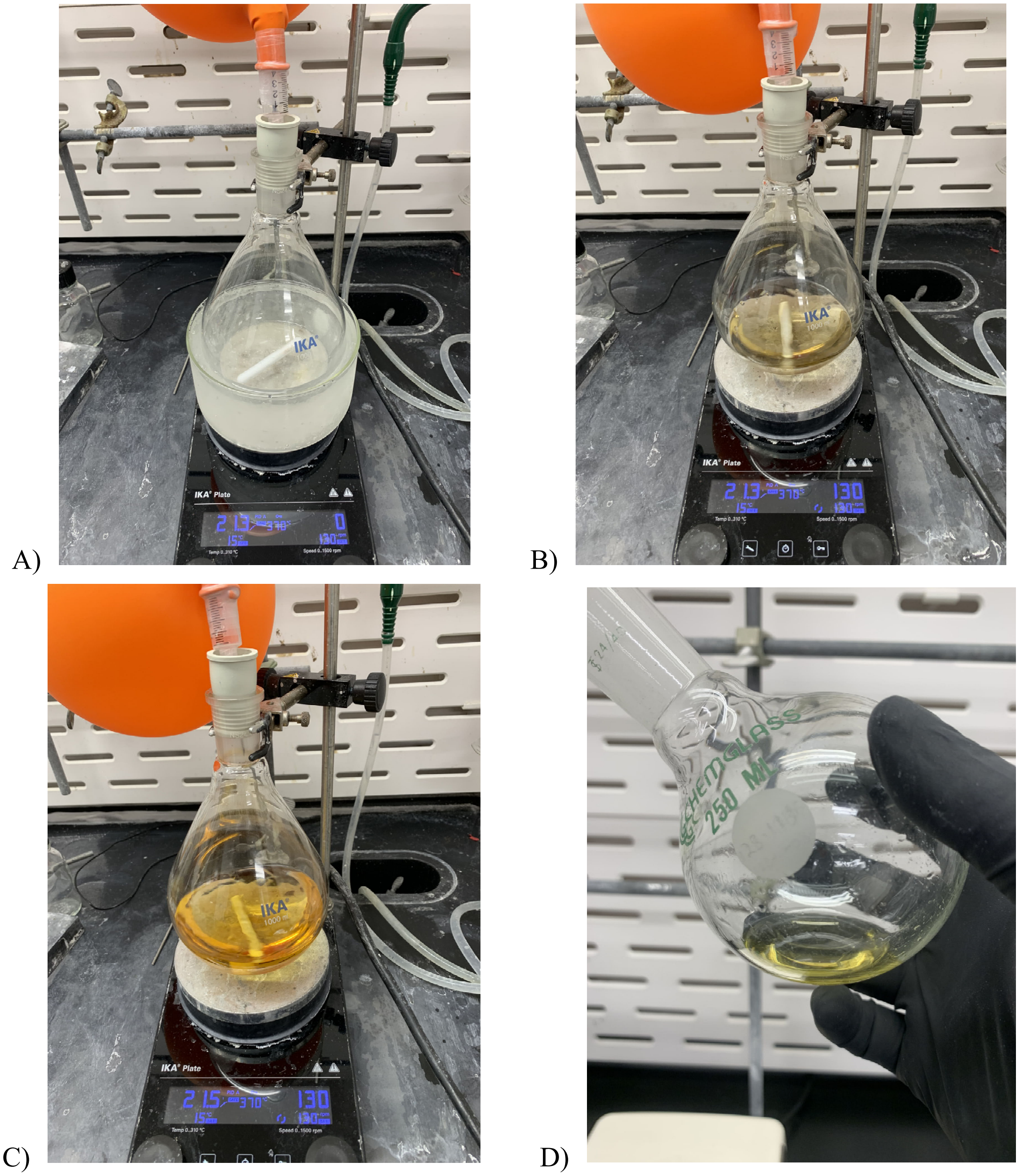

Figure S8. Decagram-scale N-O bond formation reaction set-up. A) *N*-Methylpiperazine at 0 °C prior to addition of ^{i}PrMgCl·LiCl. B) *N*-Methylpiperazine-derived magnesium amide, after addition of ^{i}PrMgCl·LiCl and stirring for 45 min. C) Reaction mixture after addition of **11** in anhydrous THF followed by stirring for 3 h. D) Isolated product **17** (6.99 g) after workup and purification by flash column chromatography on silica.

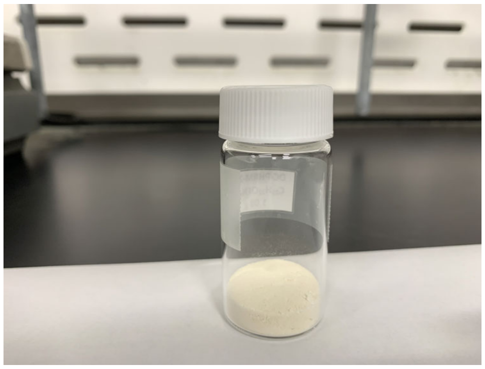

Figure S9. 1.08 g of inhibitor (**6**).

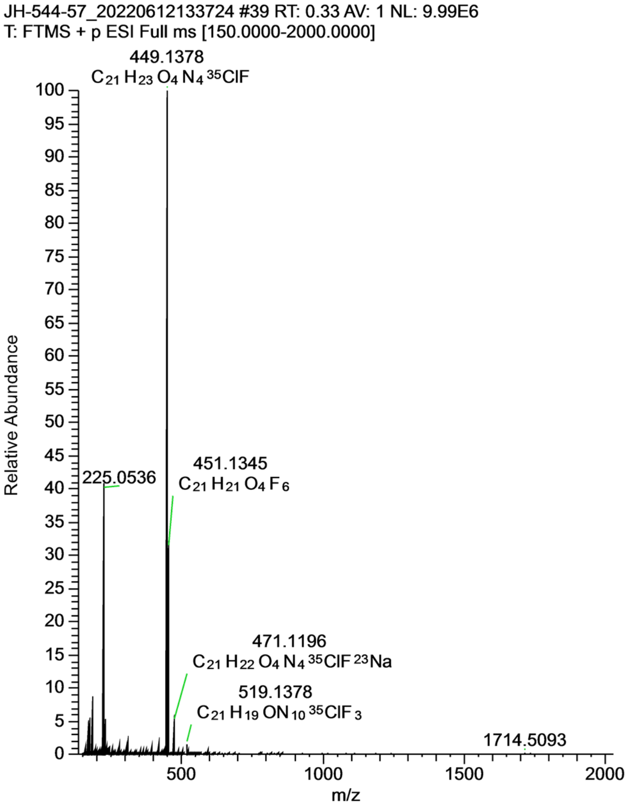

Figure S10. HRMS of inhibitor (**6**).

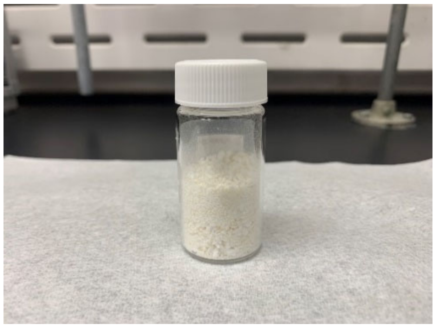

Figure S11. 1.67 g of inhibitor (**7**).

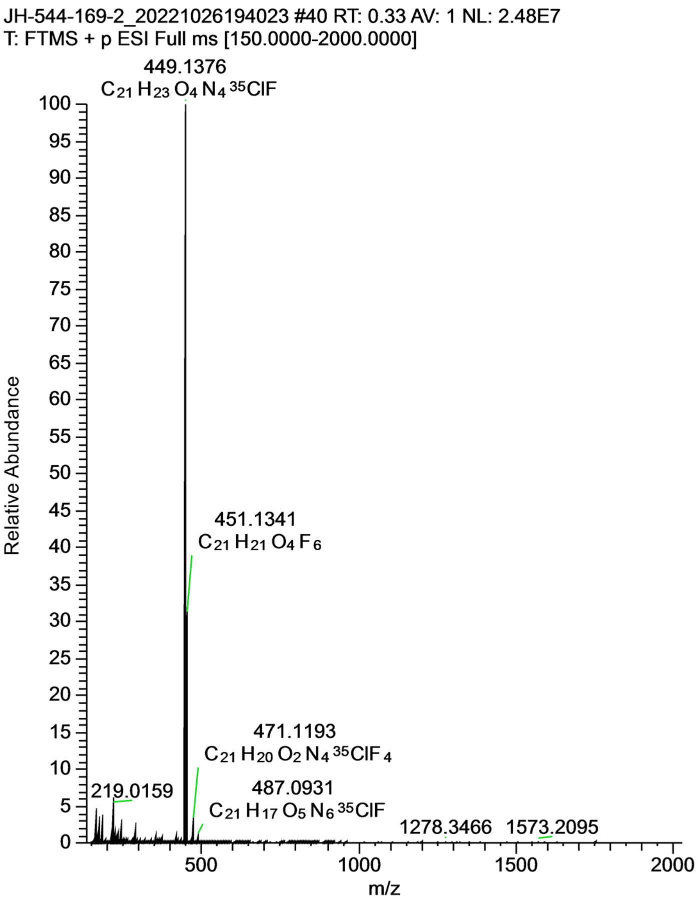

Figure S12. HRMS of inhibitor (**7**).

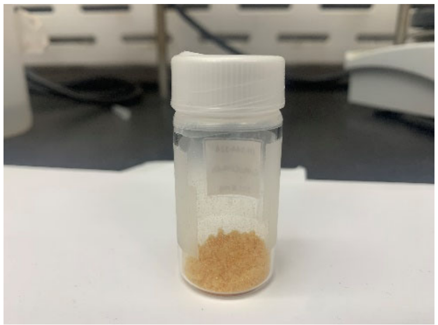

Figure S13. 286 mg of inhibitor (**8**).

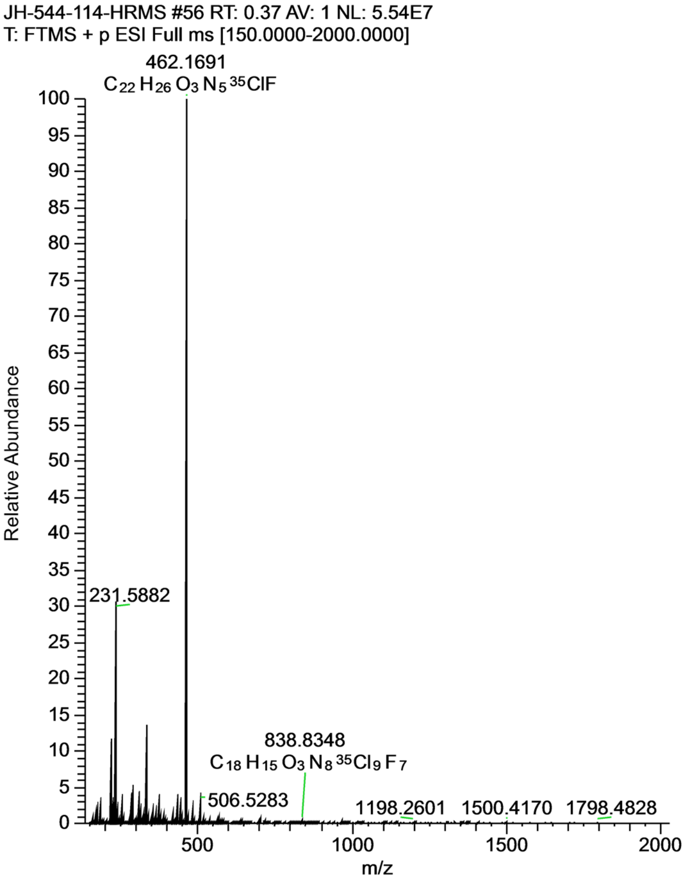

Figure S14. HRMS of inhibitor (**8**).

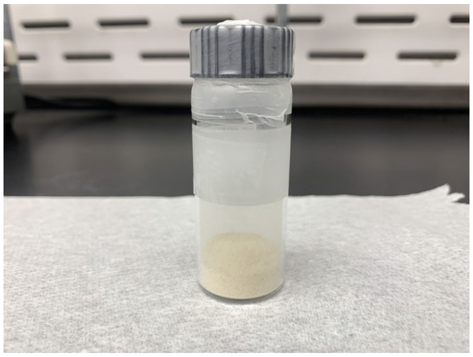

Figure S15. 1.19 g of inhibitor (**9**).

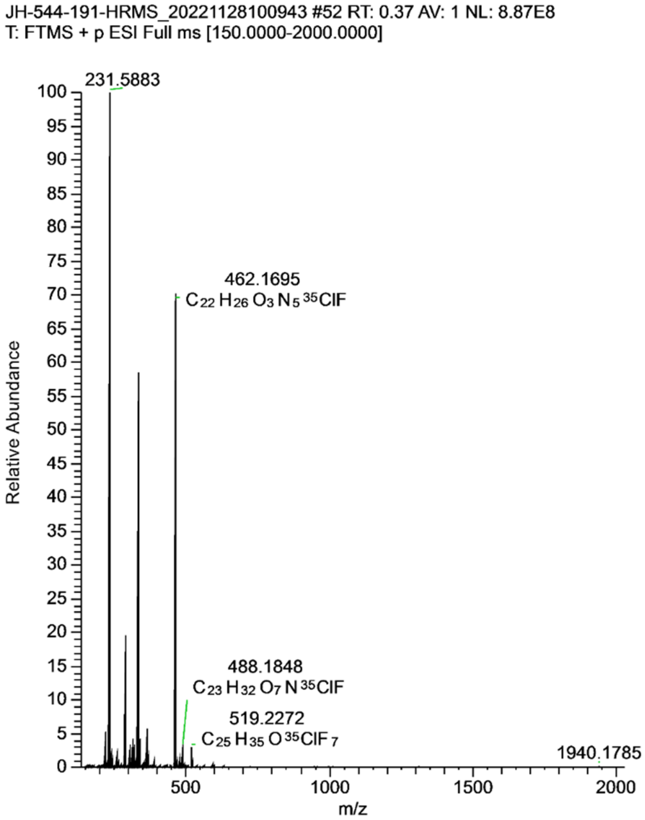

Figure S16. HRMS of inhibitor (**9**).

4. Catalog of Spectra

^{1}H NMR (500 MHz, $CDCl_3$) spectrum of *tert*-butyldimethyl(2-((2-methyltetrahydro-2*H*-pyran-2-yl)peroxy)ethoxy)silane (**11**)

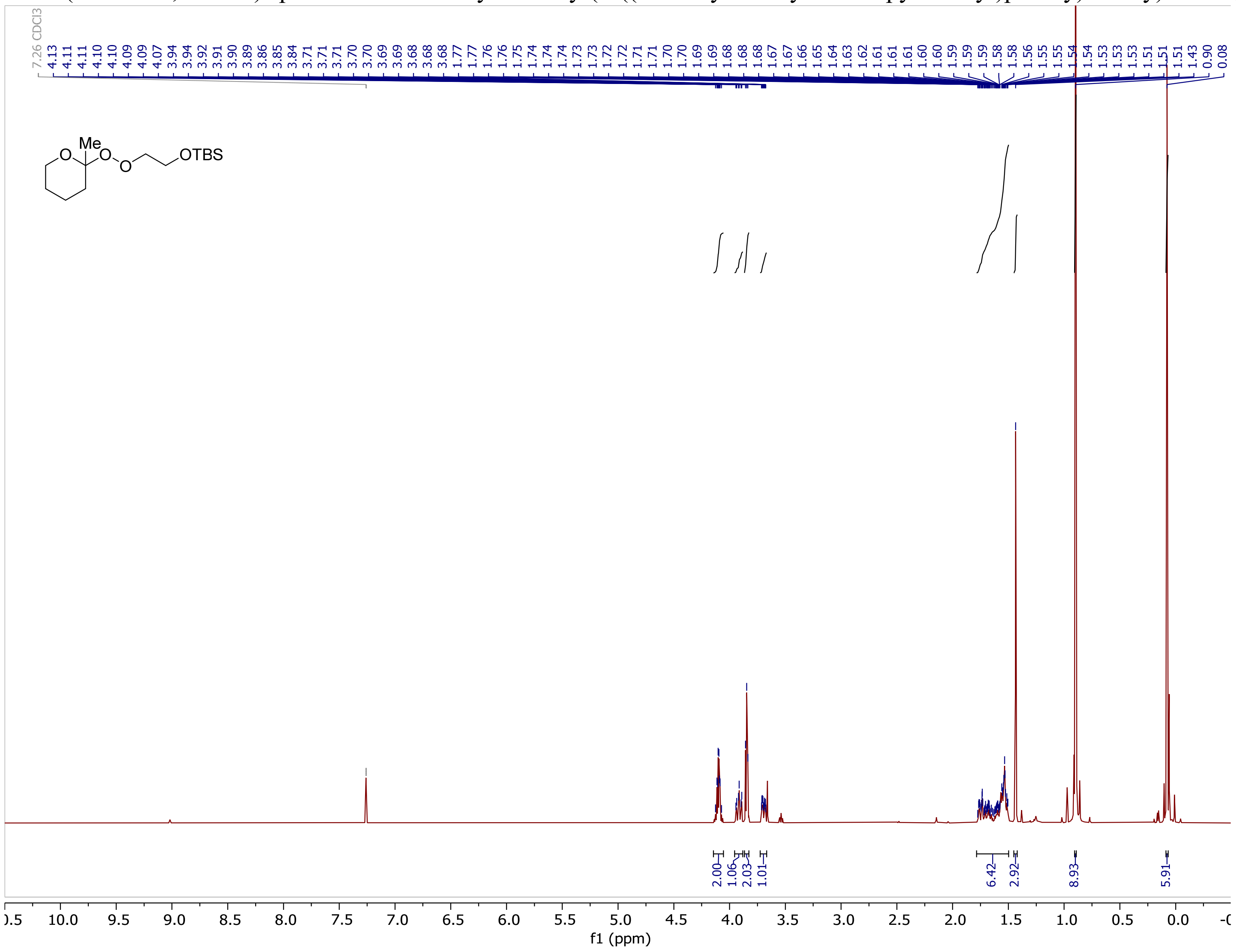

^{13}C **NMR** (126 MHz, $CDCl_3$) spectrum of *tert*-butyldimethyl(2-((2-methyltetrahydro-2*H*-pyran-2-yl)peroxy)ethoxy)silane (**11**)

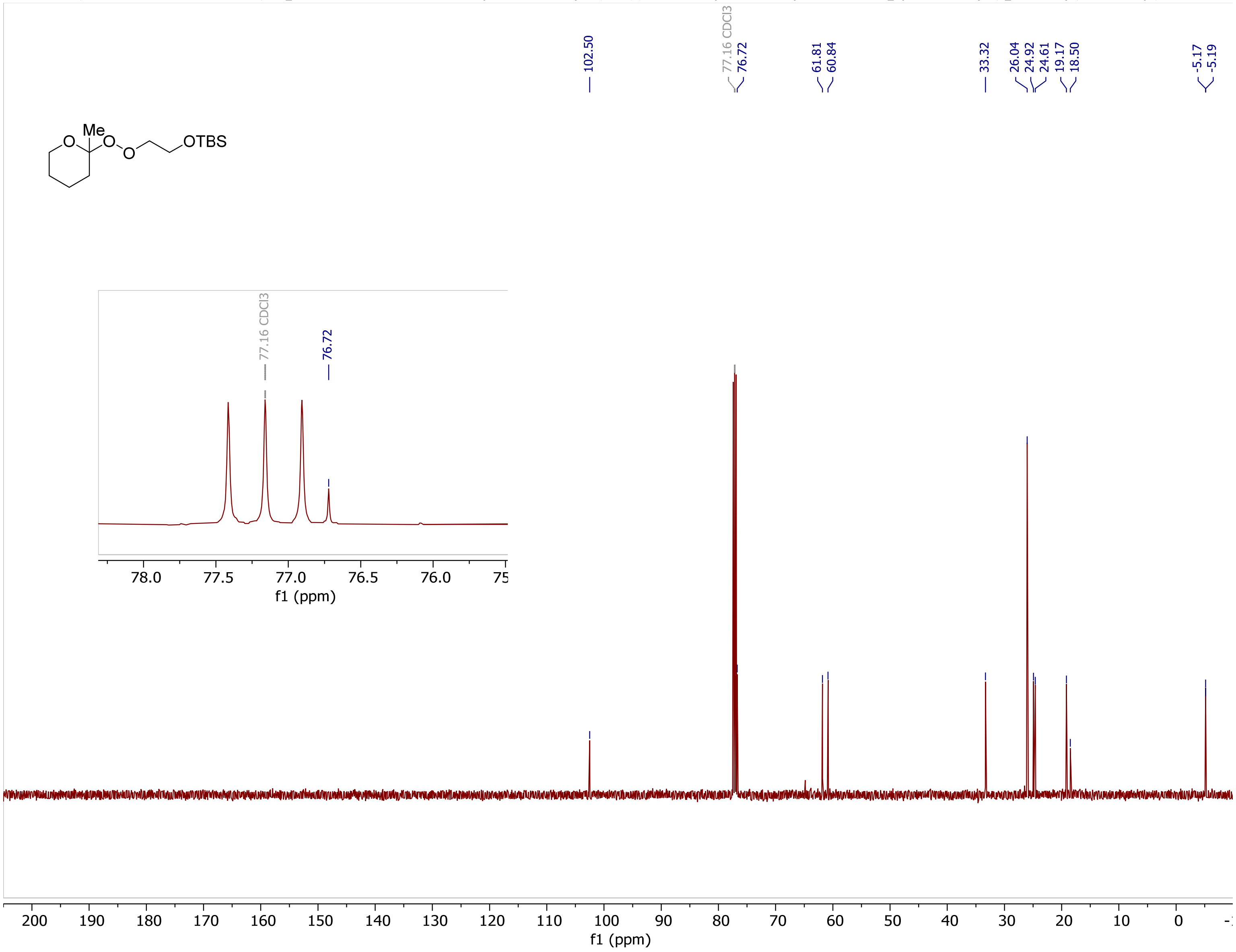

HSQC NMR (500 MHz, $CDCl_3$) spectrum of *tert*-butyldimethyl(2-((2-methyltetrahydro-2*H*-pyran-2-yl)peroxy)ethoxy)silane (**11**)

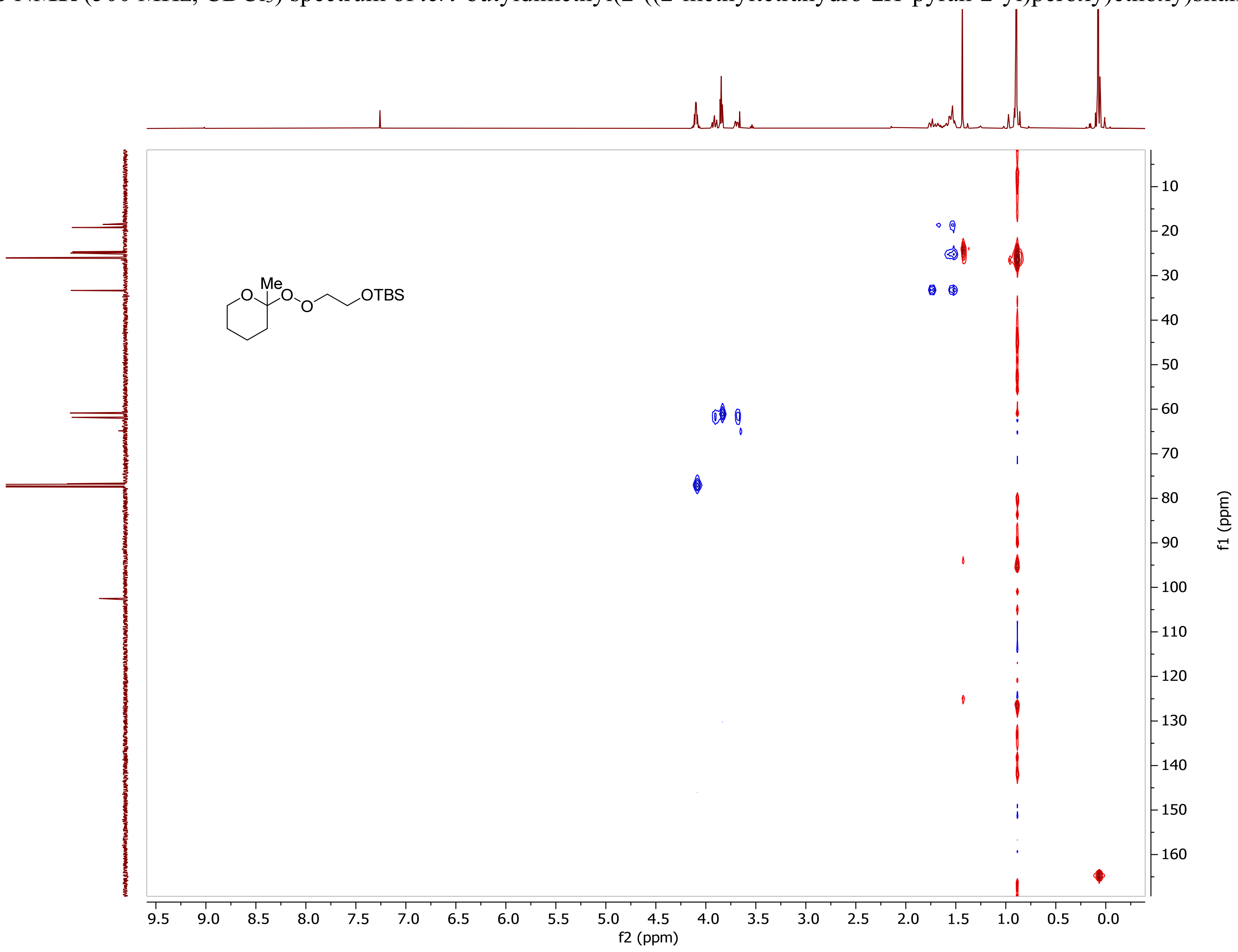

^{1}H NMR (500 MHz, CDCl3) spectrum of 4-(2-((*tert*-butyldimethylsilyl)oxy)ethoxy)morpholine (**14**)

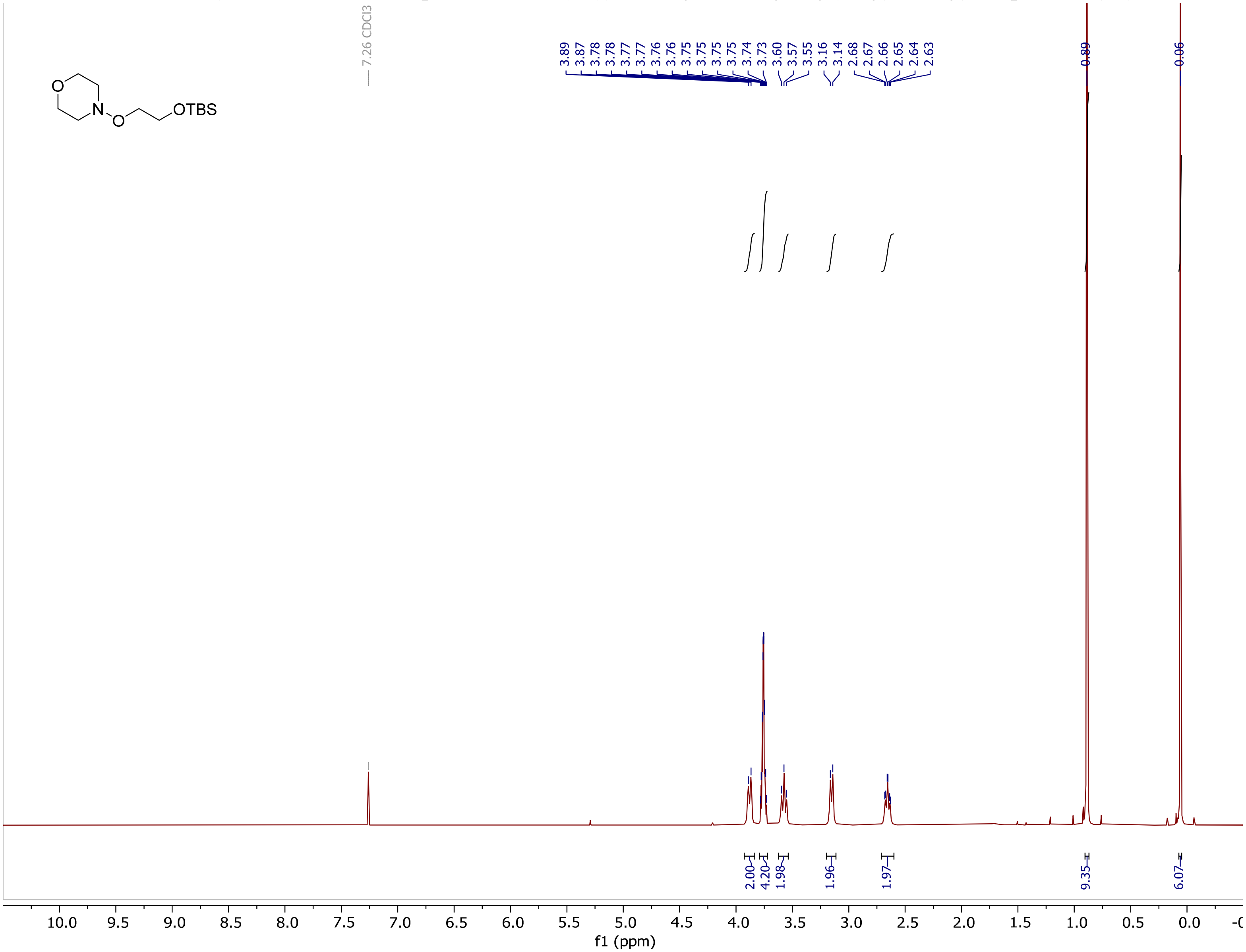

^{13}**C NMR** (126 MHz, CDCl$_3$) spectrum of 4-(2-((*tert*-butyldimethylsilyl)oxy)ethoxy)morpholine (**14**)

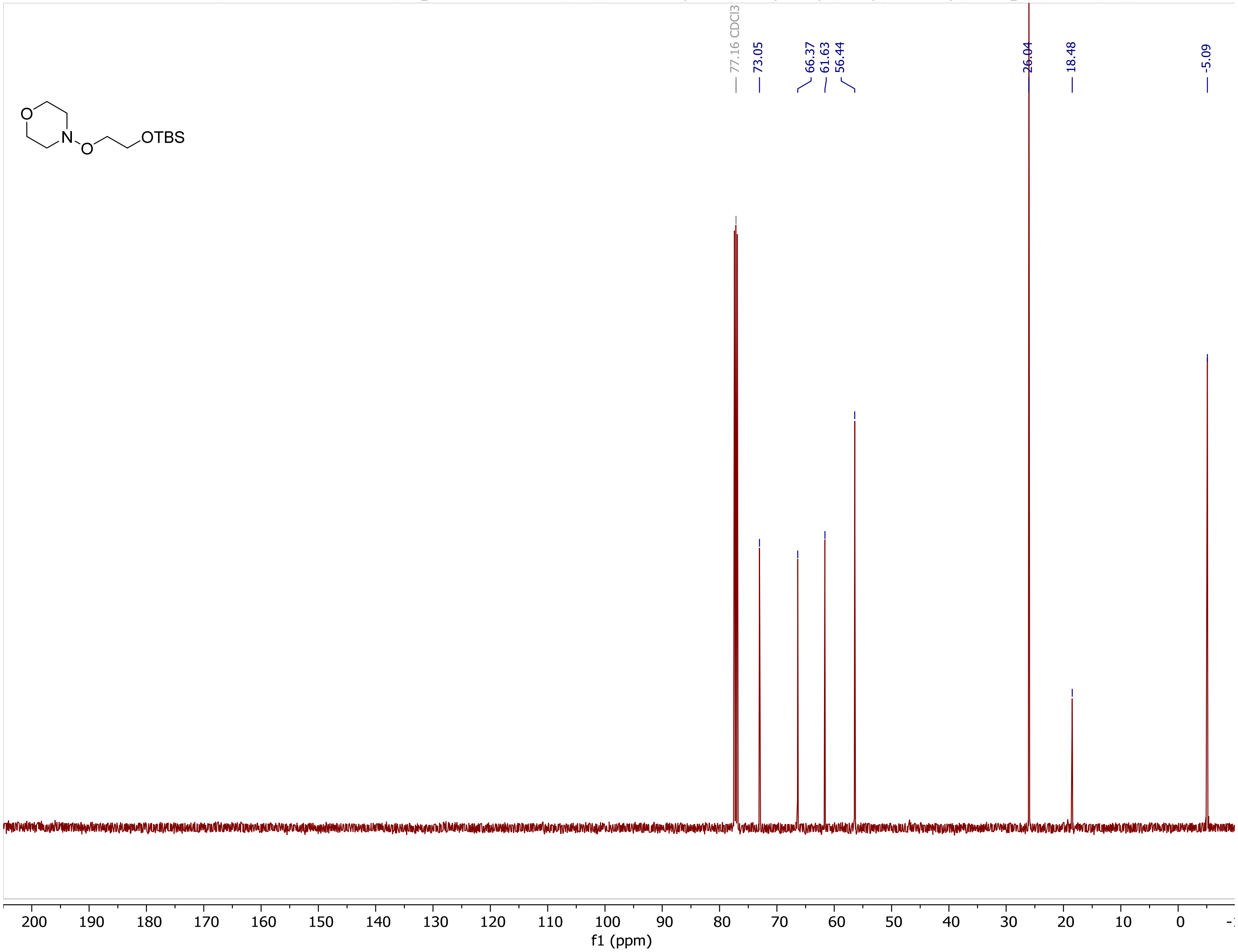

HSQC NMR (500 MHz, $CDCl_3$) spectrum of 4-(2-((*tert*-butyldimethylsilyl)oxy)ethoxy)morpholine (**14**)

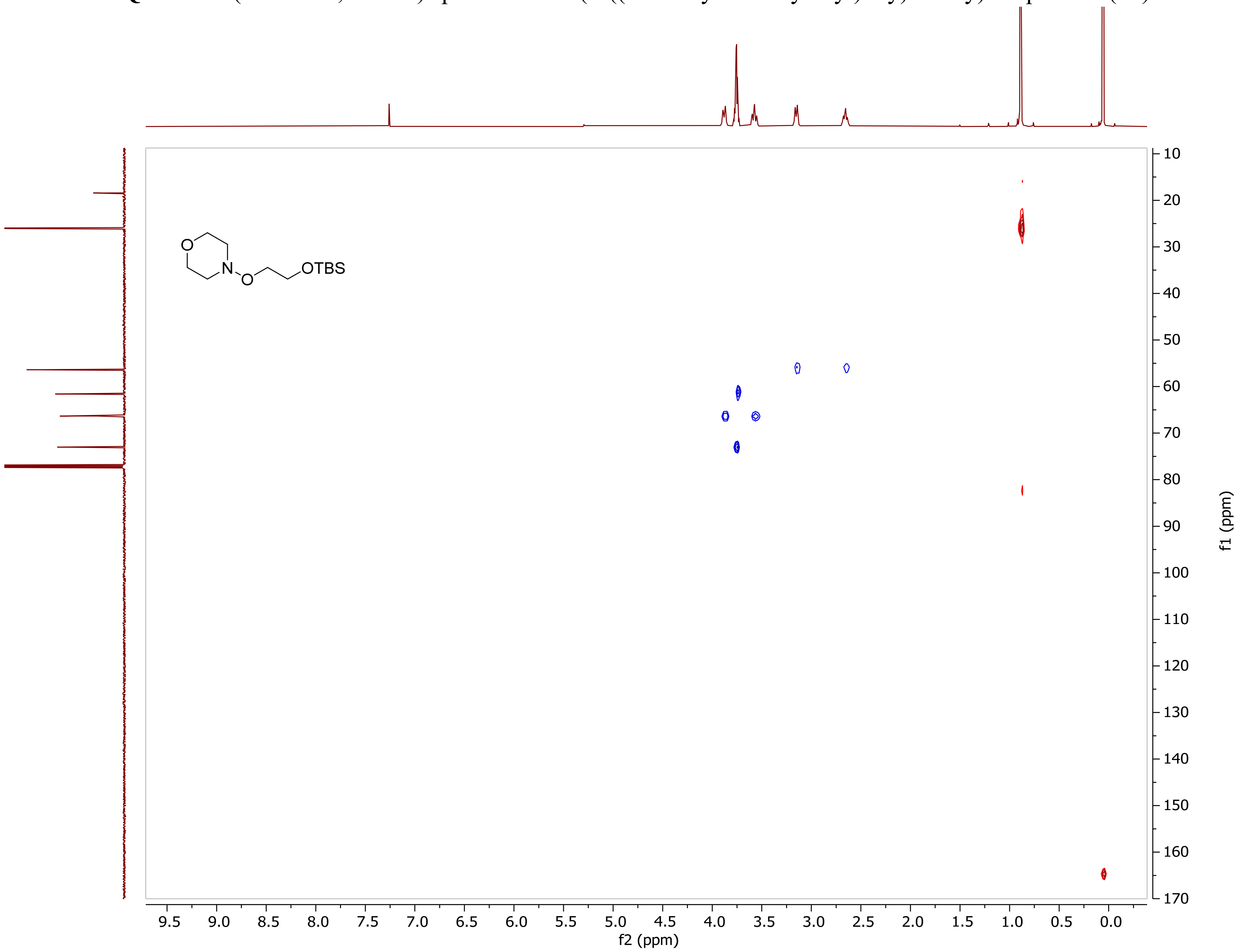

^{1}H NMR (500 MHz, C_6D_6) spectrum of 1-(2-((*tert*-butyldimethylsilyl)oxy)ethoxy)-4-methylpiperazine (**15**)

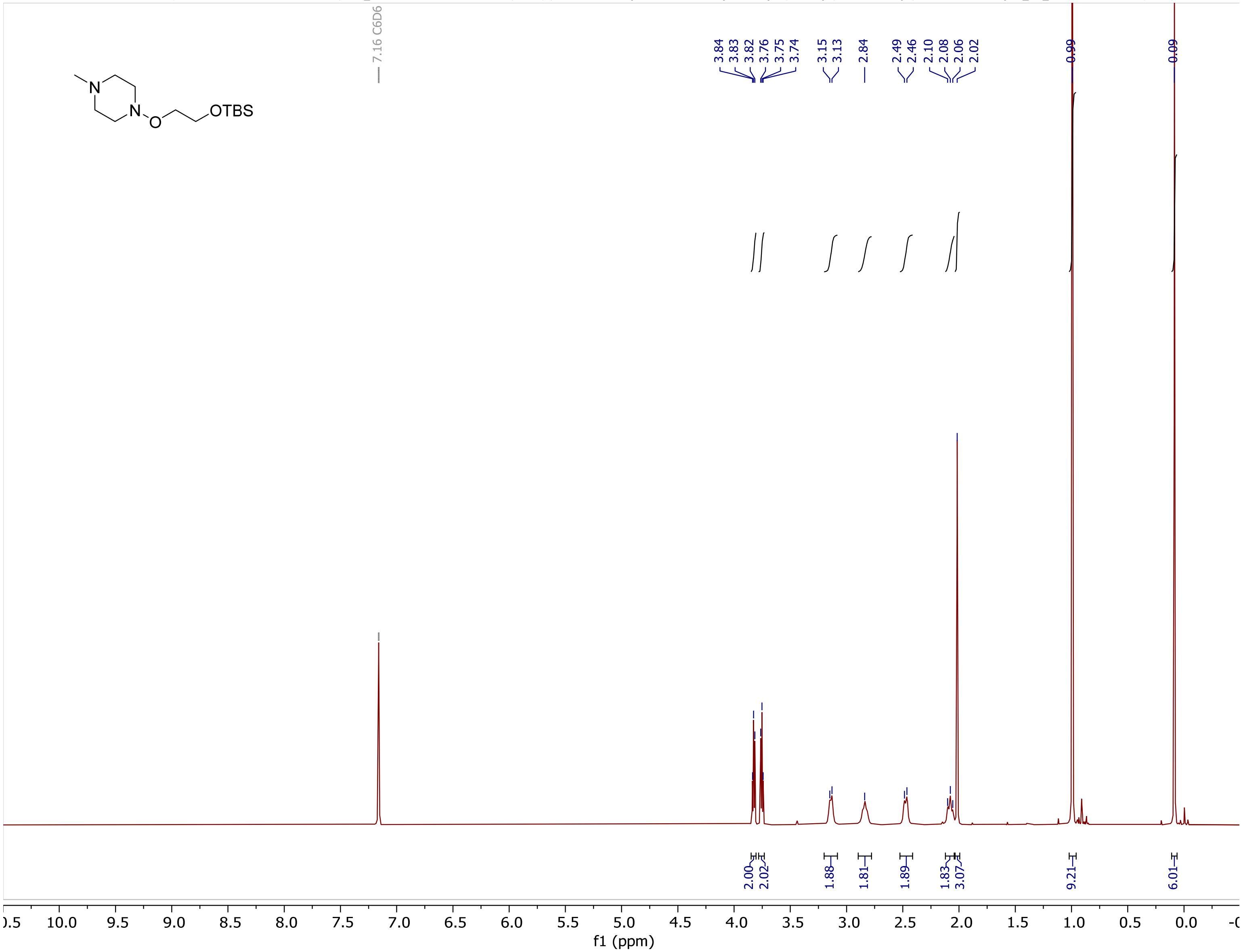

Expanded region of stacked variable temperature **^{1}H NMR** (500 MHz, C_6D_6) spectrum of 1-(2-((*tert*-butyldimethylsilyl)oxy)ethoxy)-4-methylpiperazine (**15**) at (a) 343 K b) 338 K (T_c) c) 328 K d) 318 K e) 308 K and f) 298 K. T_c refers to the coalescence temperature.

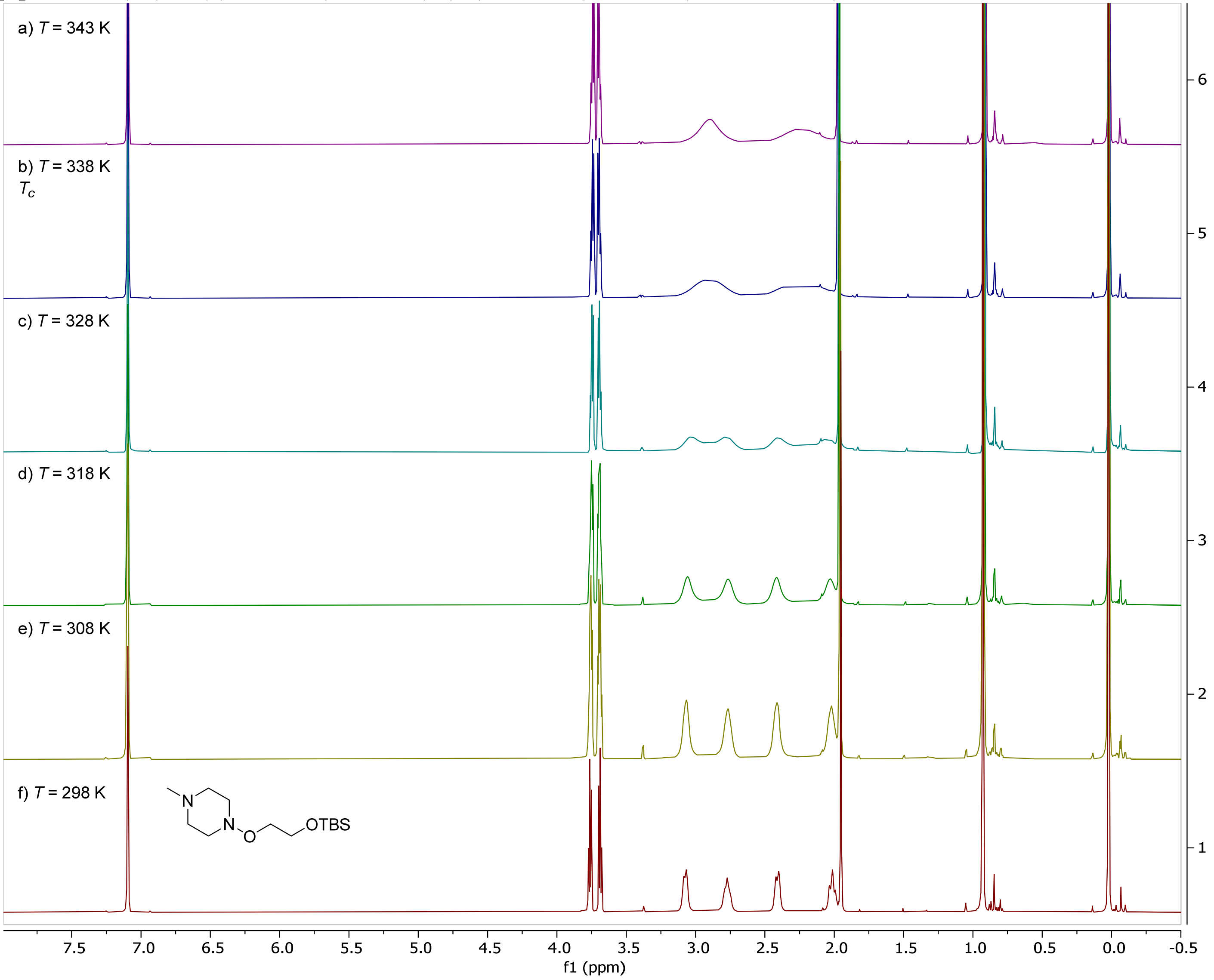

^{13}C NMR (126 MHz, C_6D_6) spectrum of 1-(2-((*tert*-butyldimethylsilyl)oxy)ethoxy)-4-methylpiperazine (**15**)

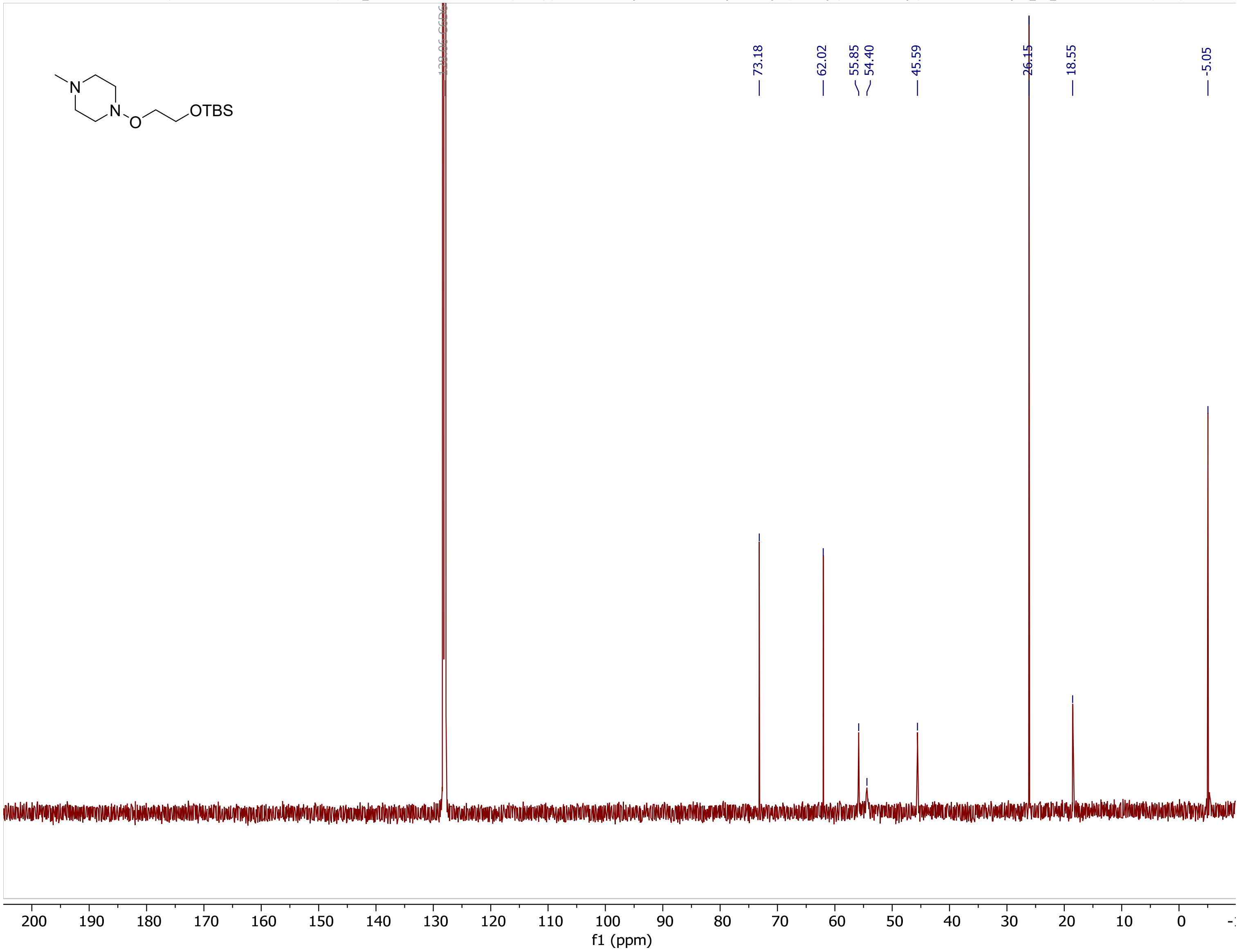

HSQC NMR (500 MHz, C_6D_6) spectrum of1-(2-((*tert*-butyldimethylsilyl)oxy)ethoxy)-4-methylpiperazine (**15**)

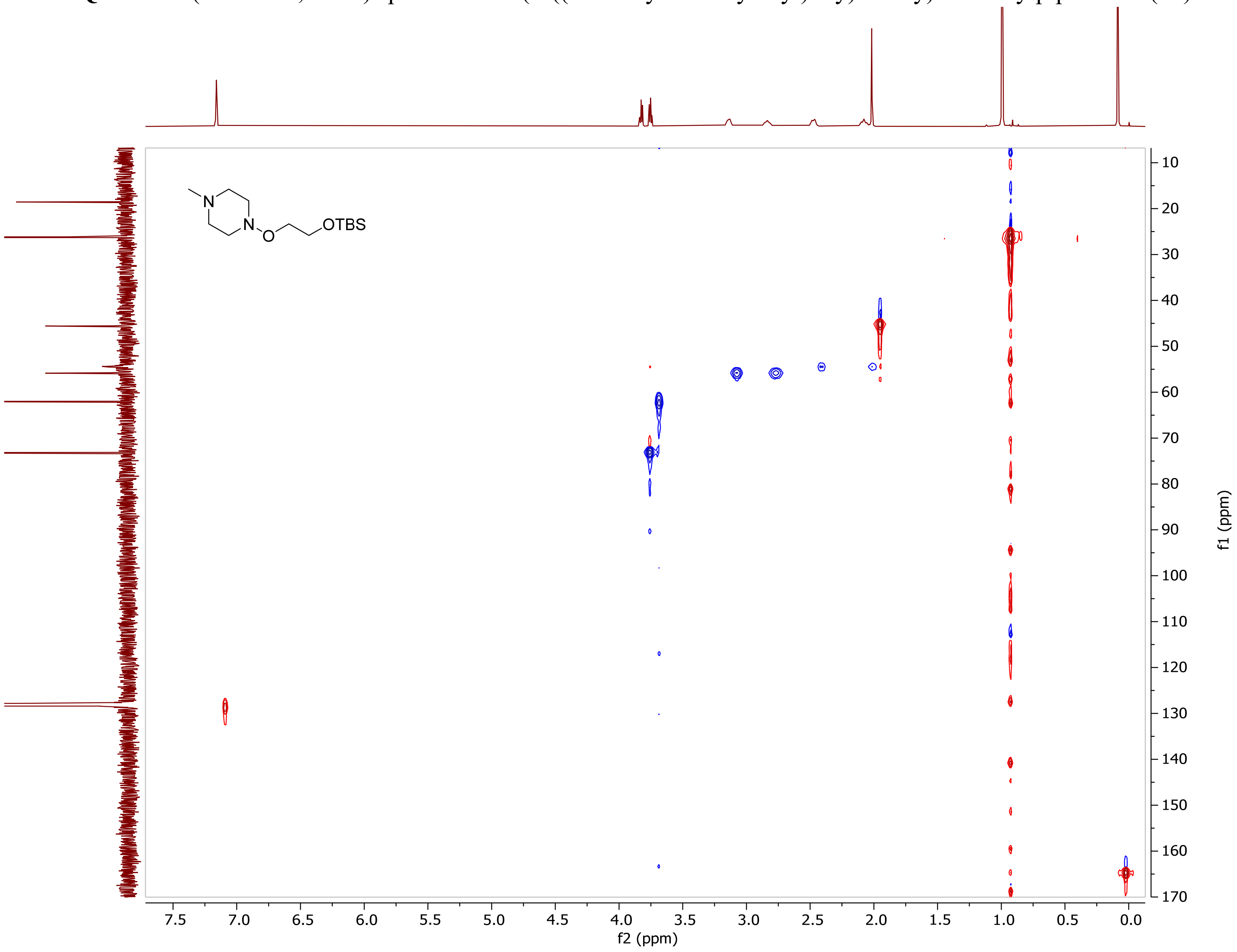

Expanded region of stacked variable temperature **^{13}C NMR** (126 MHz, C_6D_6) spectrum of 1-(2-((*tert*-butyldimethylsilyl)oxy)ethoxy)-4-methylpiperazine (**15**) at a) 338 K and b) 298 K

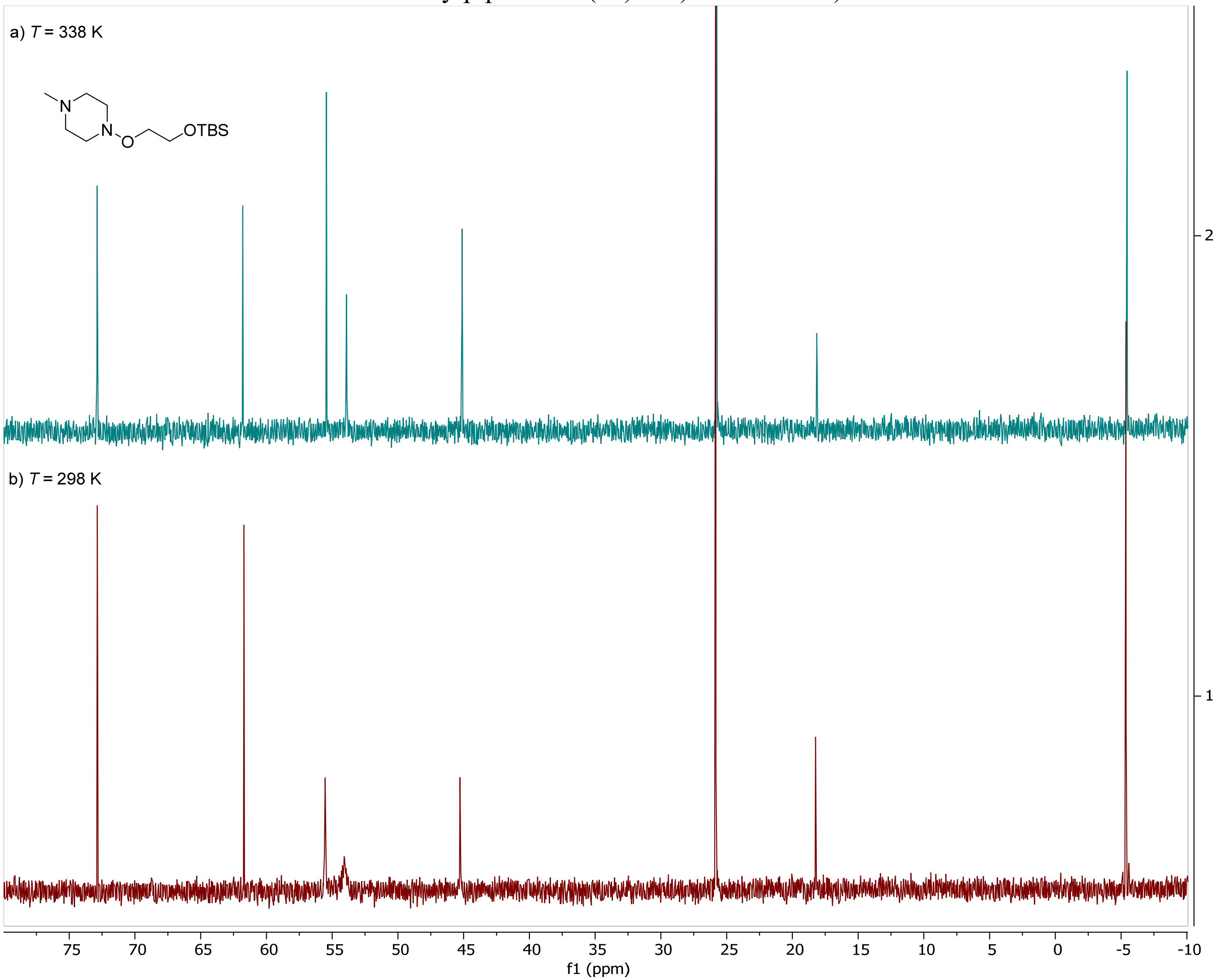

^{1}H NMR (500 MHz, $CDCl_3$) spectrum of 2-(morpholinooxy)ethan-1-ol (**16**)

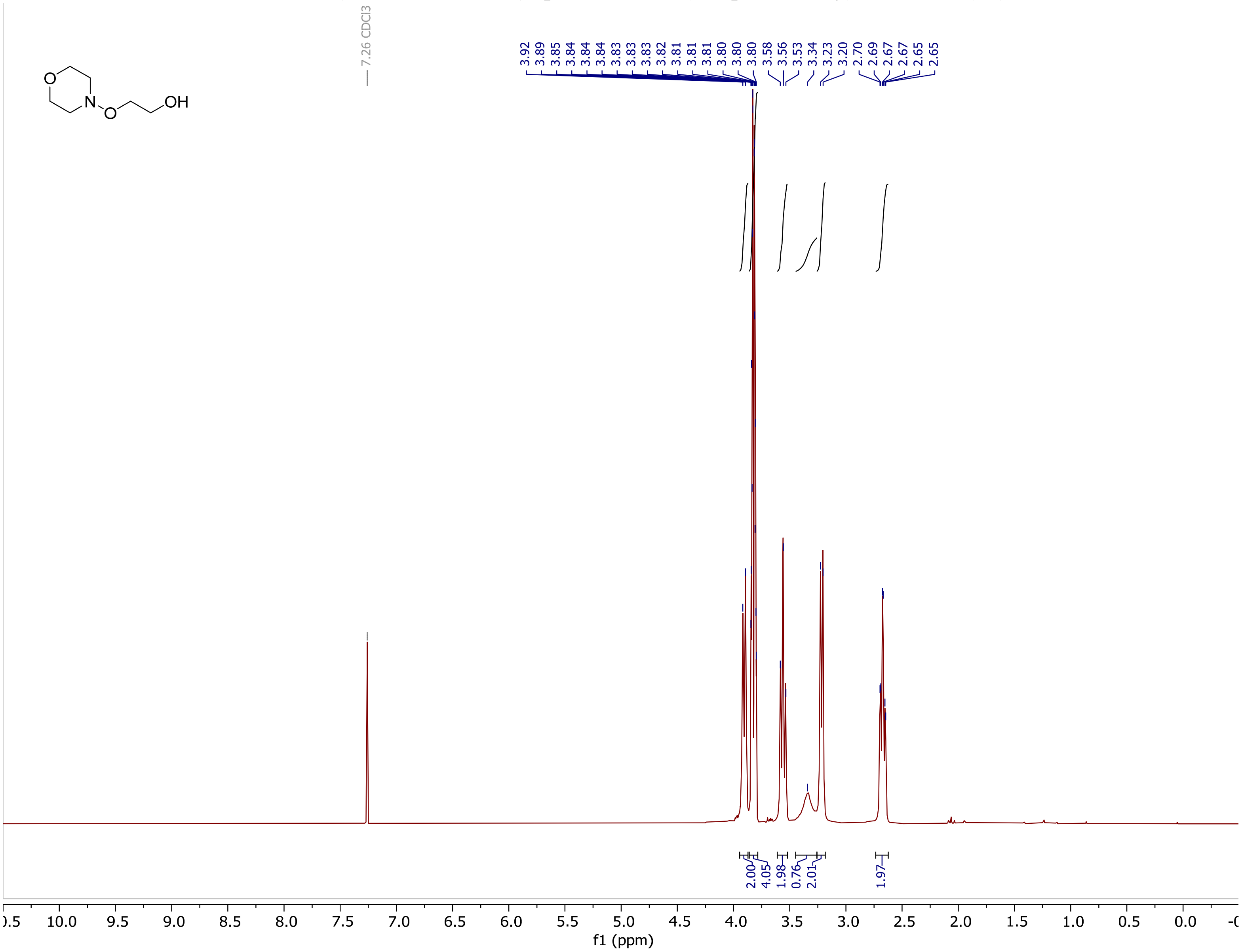

^{13}C NMR (126 MHz, $CDCl_3$) spectrum of 2-(morpholinooxy)ethan-1-ol (**16**)

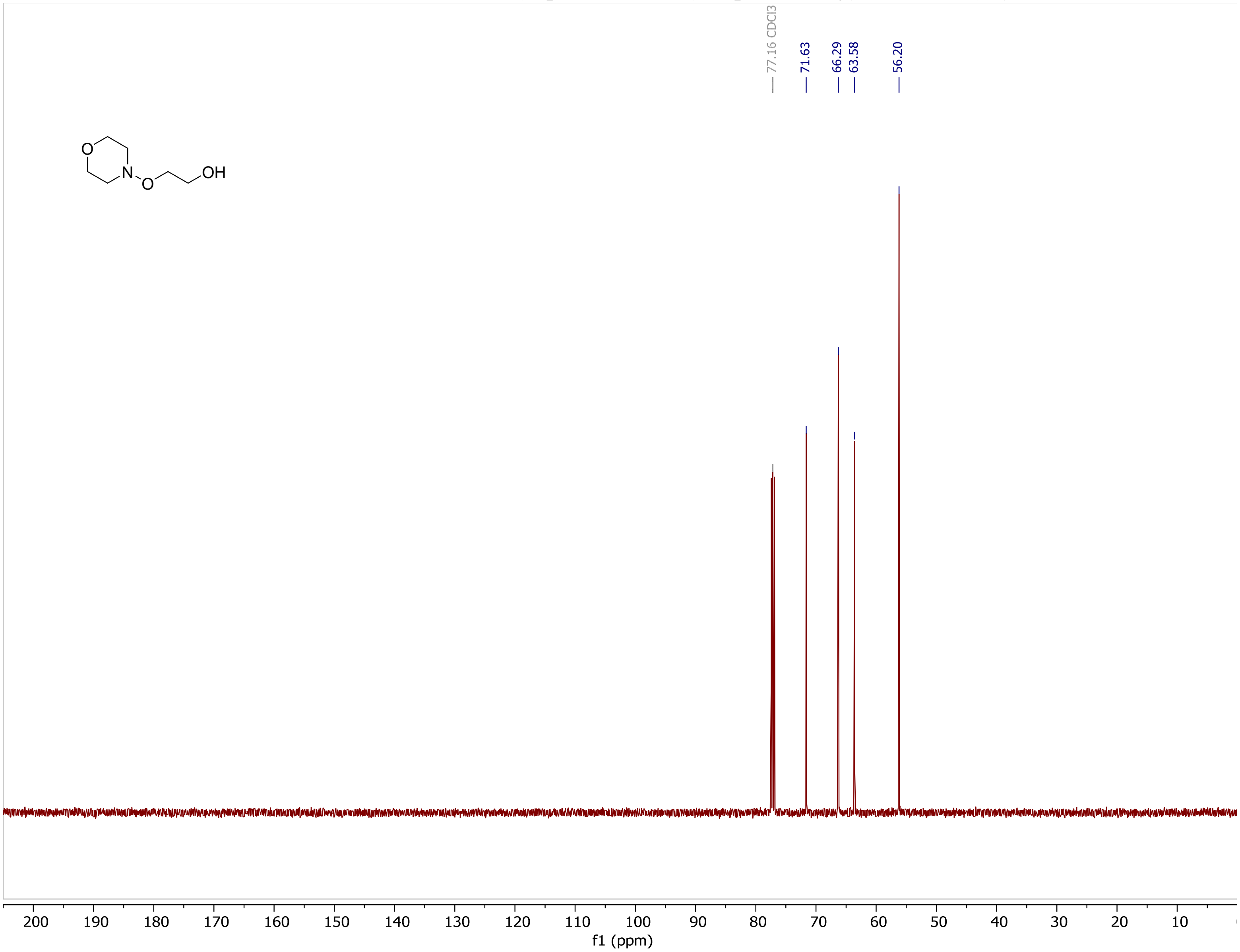

HSQC NMR (500 MHz, $CDCl_3$) spectrum of 2-(morpholinooxy)ethan-1-ol (**16**)

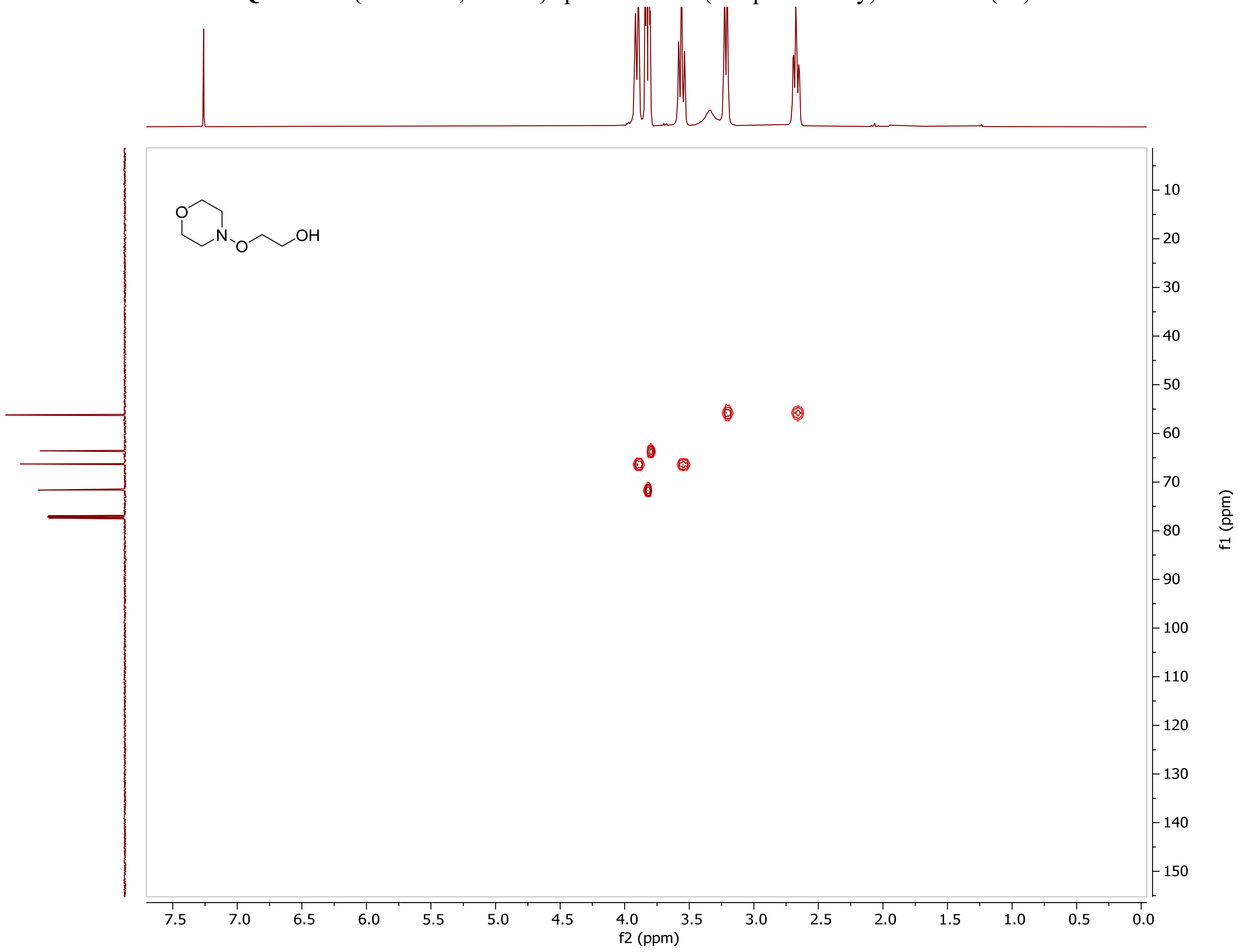

^{1}H NMR (500 MHz, Toluene-D8) spectrum of 2-((4-methylpiperazin-1-yl)oxy)ethan-1-ol (**17**)

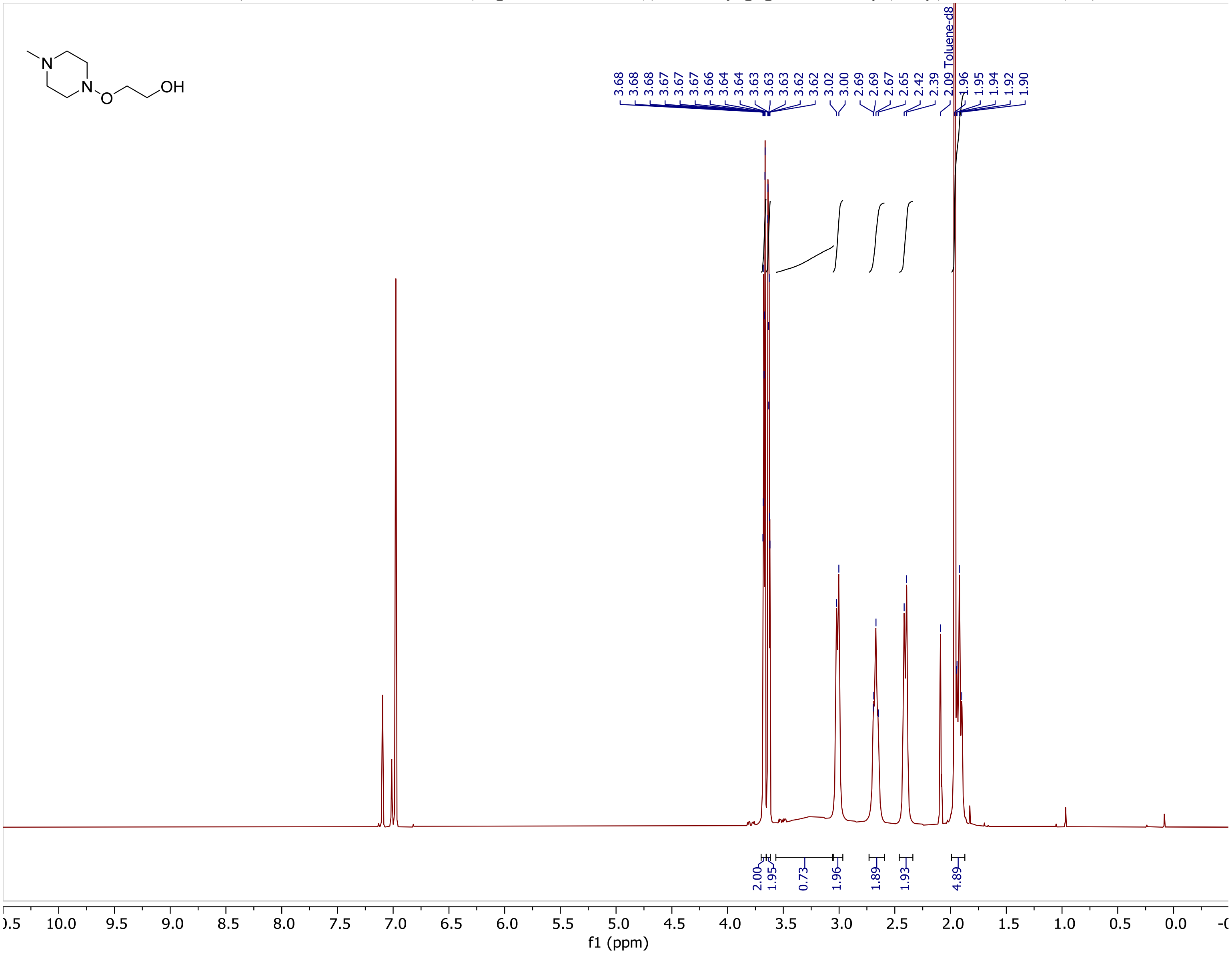

Expanded region of stacked variable temperature **^{1}H NMR** (500 MHz, Toluene-D$_8$) spectrum of 2-((4-methylpiperazin-1-yl)oxy)ethan-1-ol (**17**)at (a) 358 K b) 348 K (T_c) c) 338 K d) 328 K e) 318 K and f) 308 K g) 298 K. T_c refers to the coalescence temperature.

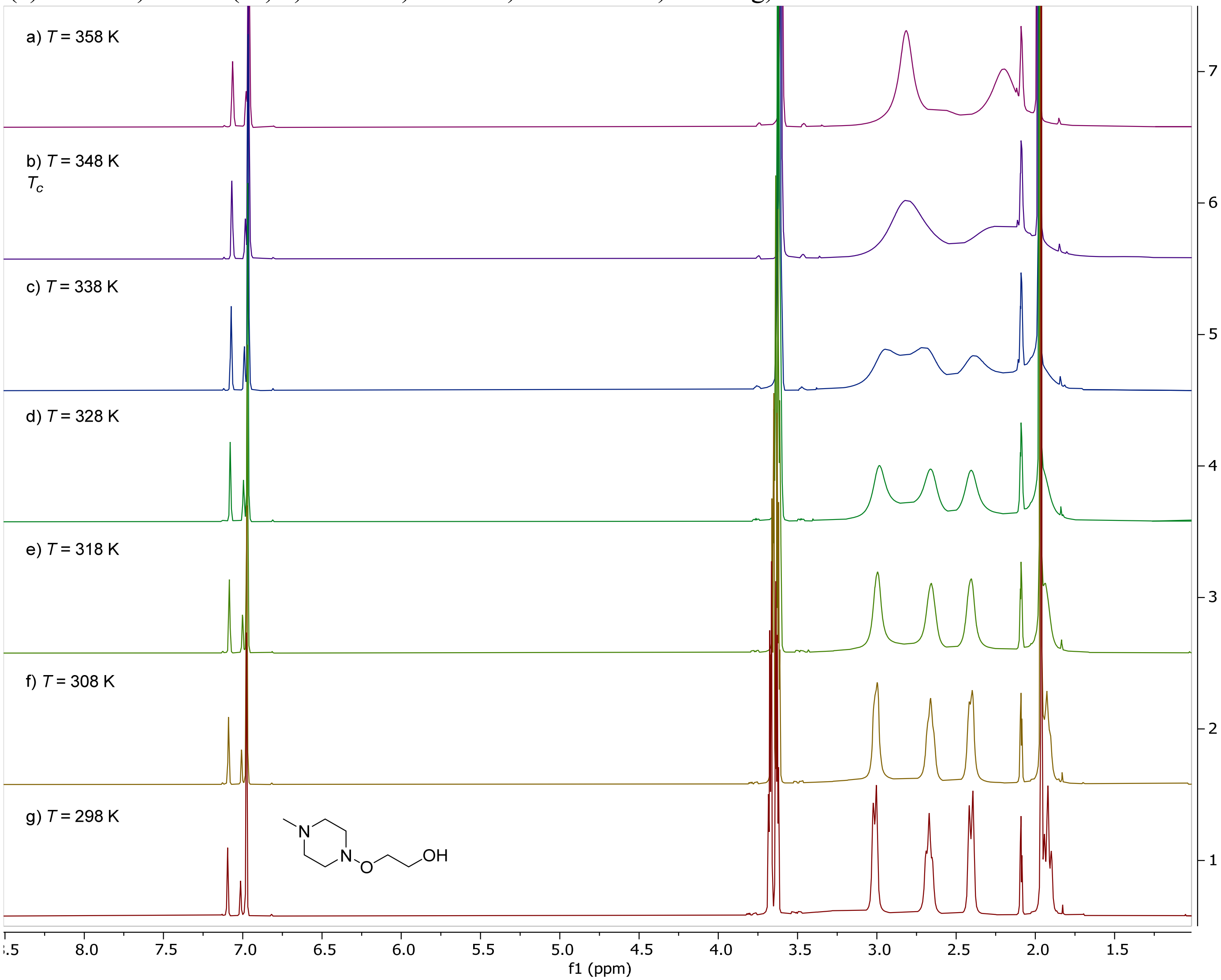

^{13}C **NMR** (126 MHz, Toluene-D$_8$) spectrum of 2-((4-methylpiperazin-1-yl)oxy)ethan-1-ol (**17**)

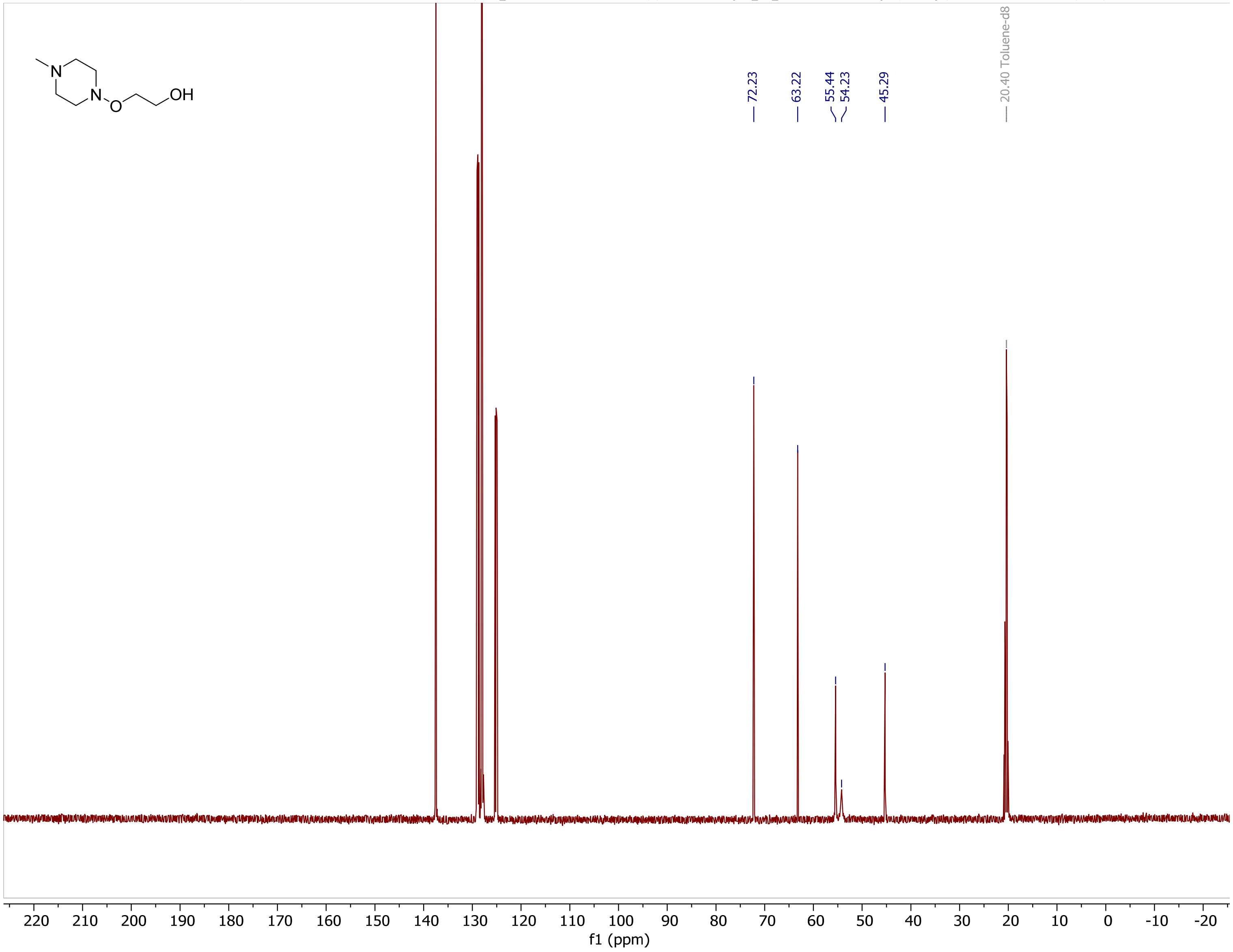

HSQC NMR (500 MHz, Toluene-D_8) spectrum of 2-((4-methylpiperazin-1-yl)oxy)ethan-1-ol (**17**)

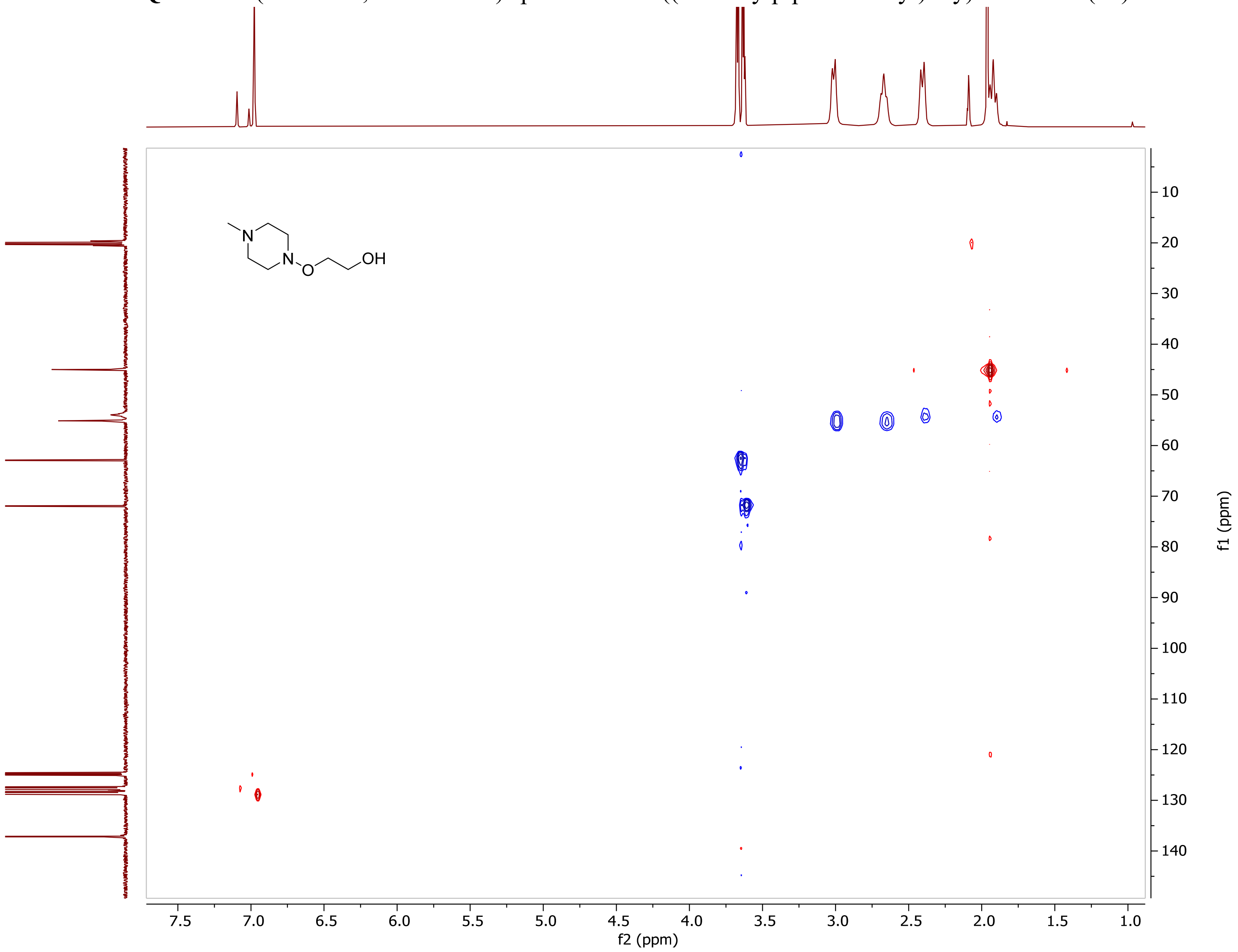

Expanded region of stacked variable temperature **^{13}C NMR** (126 MHz, Toluene-D_8) spectrum of 2-((4-methylpiperazin-1-yl)oxy)ethan-1-ol (**17**)at a) 348 K and b) 298 K

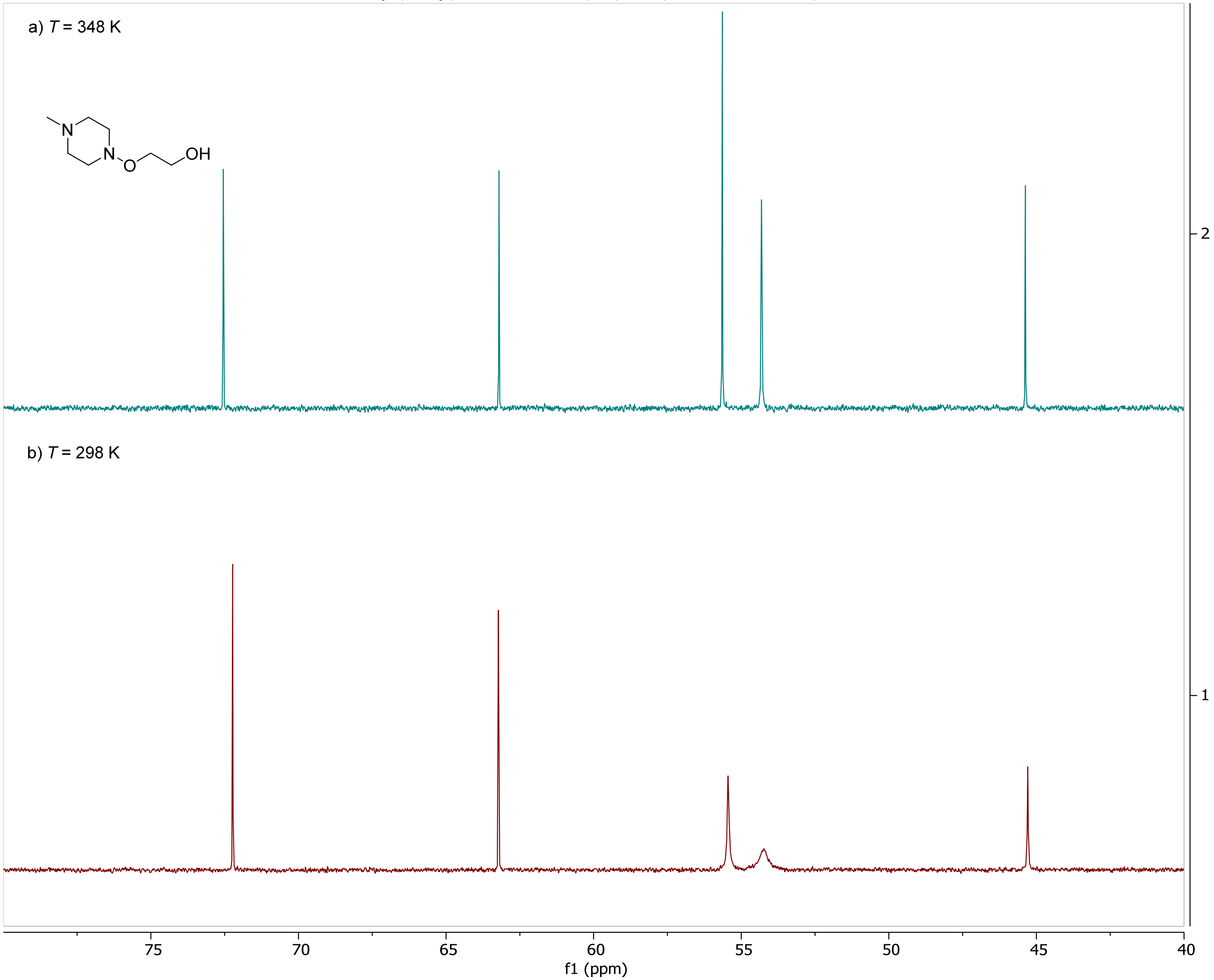

^{1}H NMR (500 MHz, DMSO-D$_6$) spectrum of *N*-(3-chloro-4-fluorophenyl)-7-methoxy-6-(2-(morpholinooxy)ethoxy)quinazolin-4-amine (**6**)

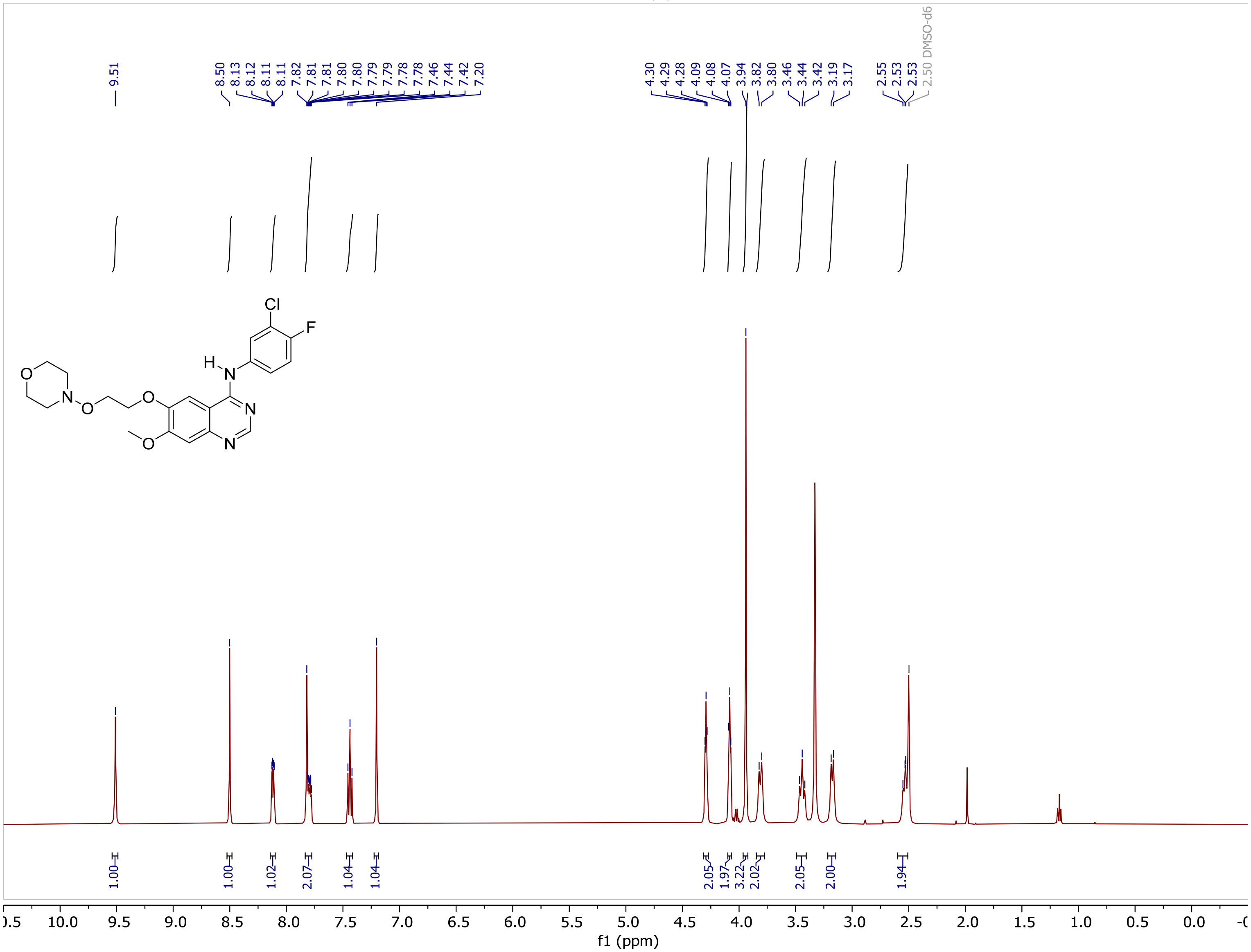

Stacked ^{1}**H NMR** (500 MHz, DMSO-D$_6$) spectrum of a) *N*-(3-chloro-4-fluorophenyl)-7-methoxy-6-(2-(morpholinooxy)ethoxy)quinazolin-4-amine (**6**) and b) Gefitinib (**1**)

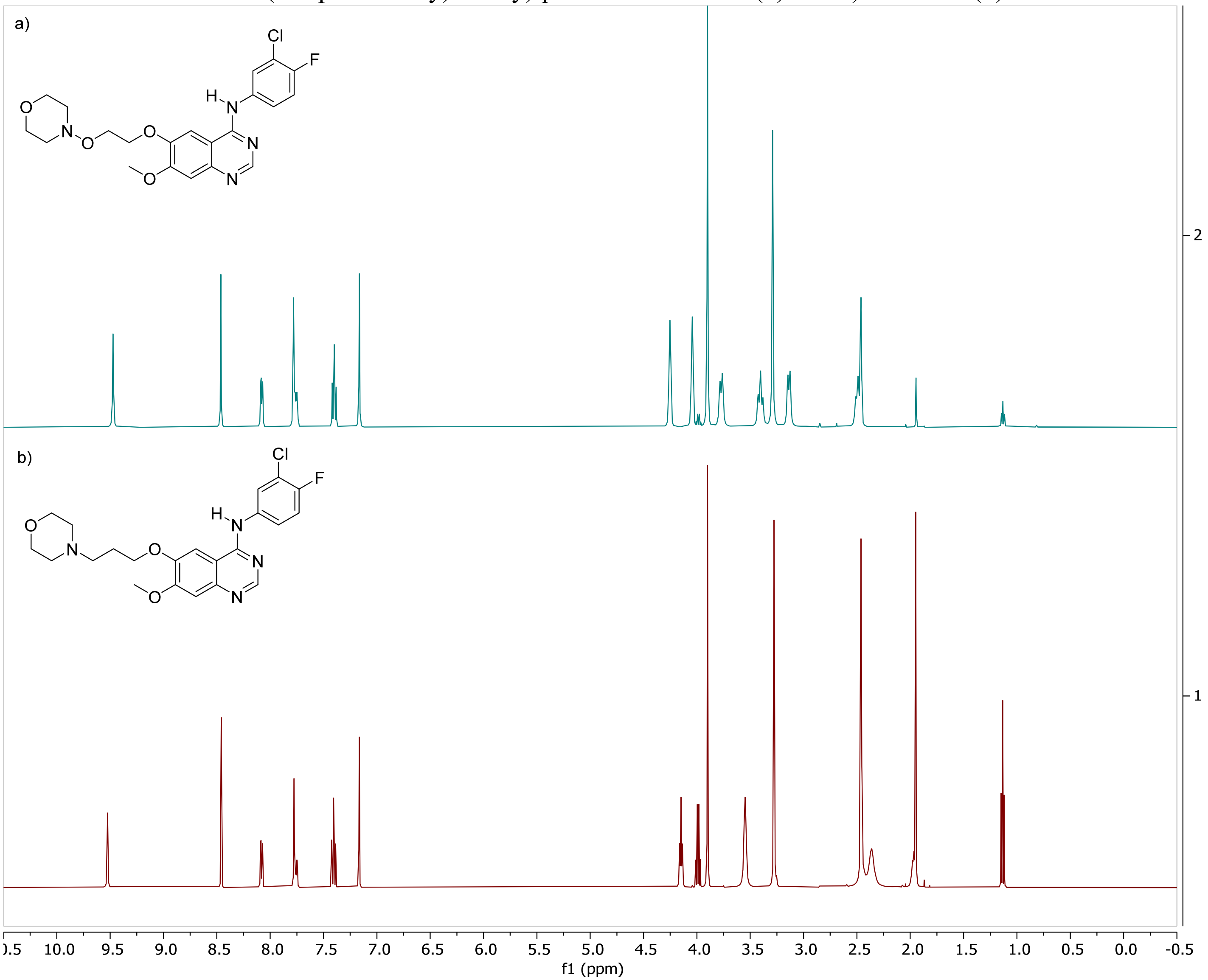

^{13}C NMR (126 MHz, DMSO-D₆) spectrum of *N*-(3-chloro-4-fluorophenyl)-7-methoxy-6-(2-(morpholinooxy)ethoxy)quinazolin-4-amine (**6**)

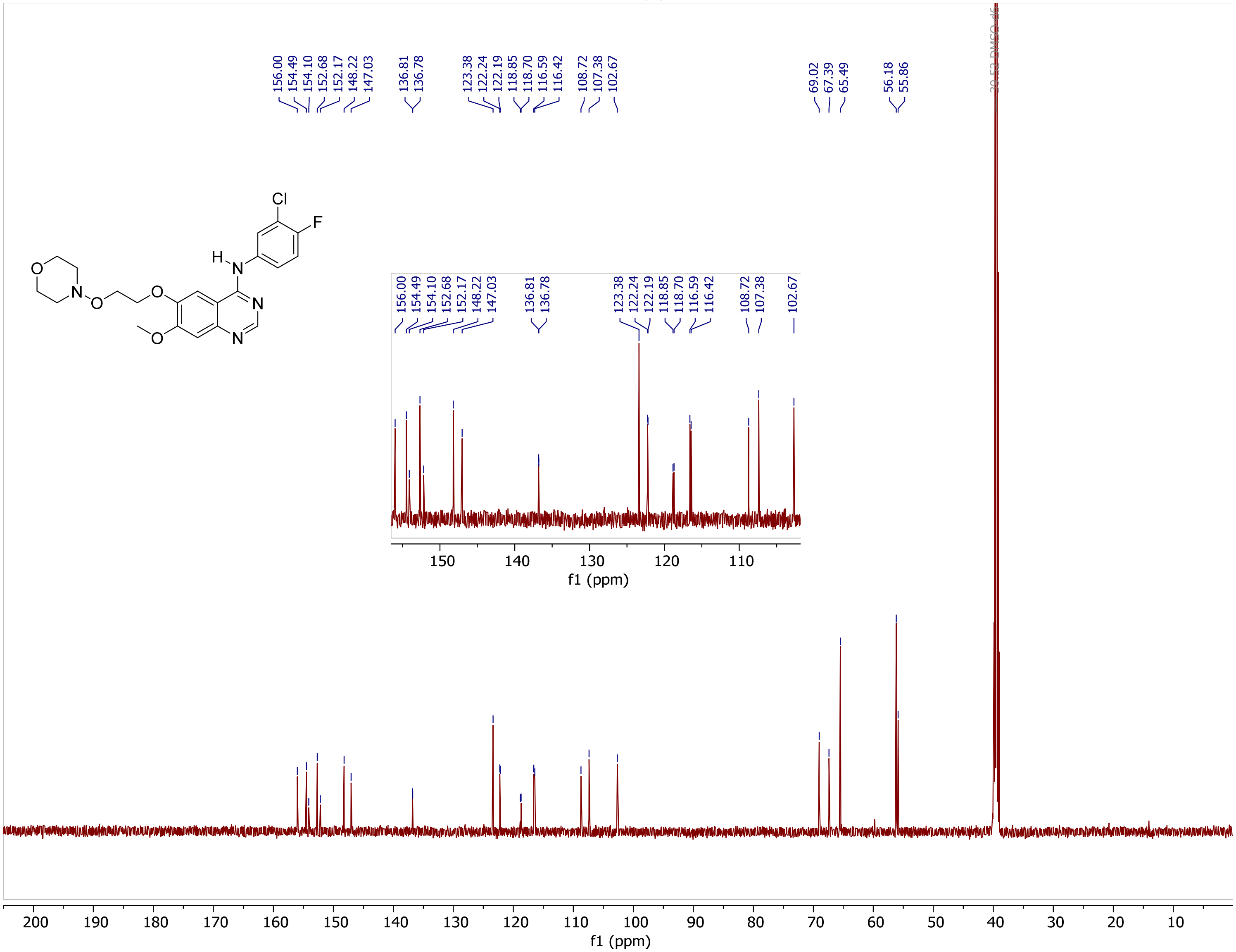

^{13}C NMR {^{19}F} (126 MHz, DMSO-D$_6$) spectrum of *N*-(3-chloro-4-fluorophenyl)-7-methoxy-6-(2-(morpholinooxy)ethoxy)quinazolin-4-amine (**6**)

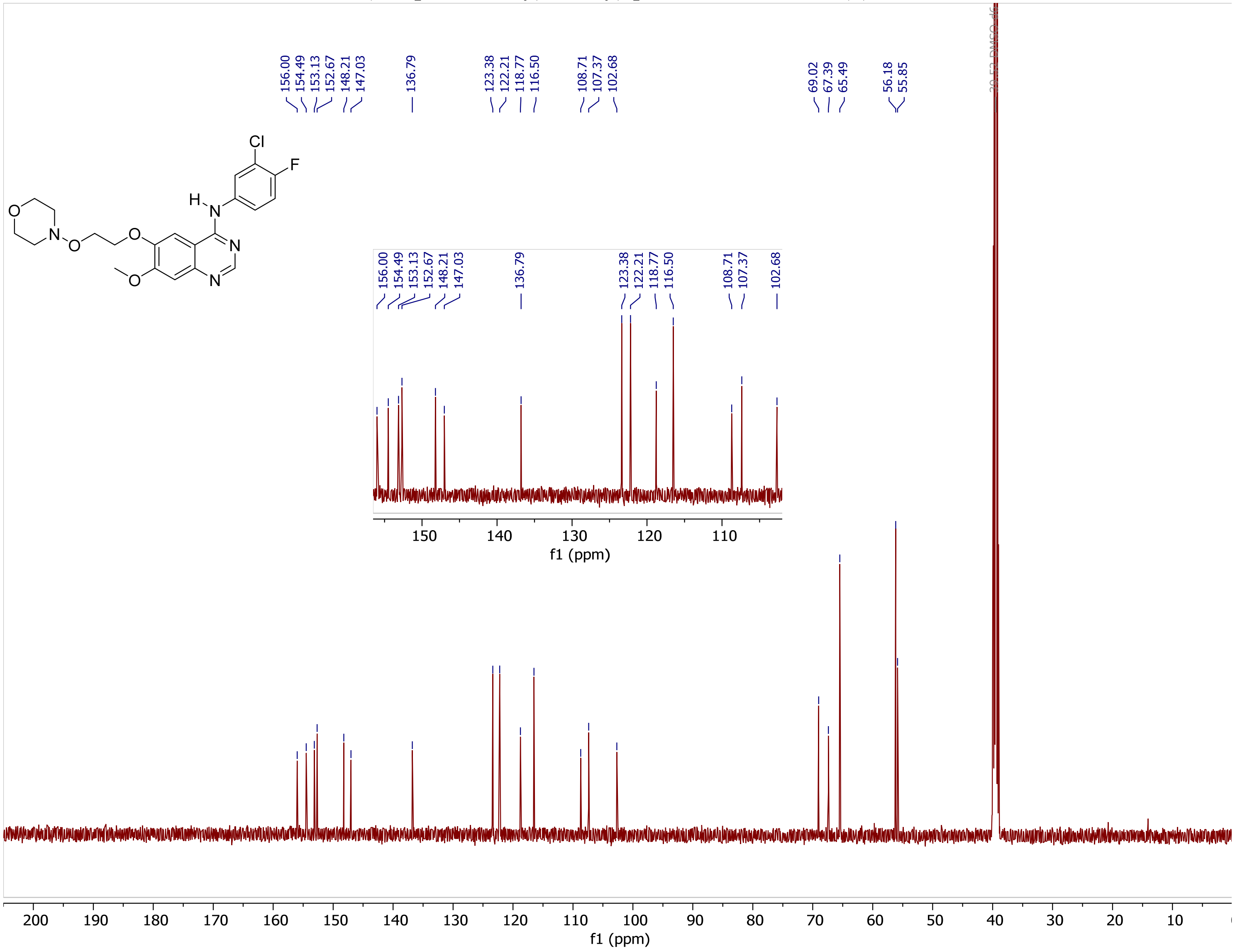

Expanded region of stacked a) **^{13}C NMR** {^{19}F} (126 MHz, DMSO-D6) and b) **^{13}C NMR** (126 MHz, DMSO-D6) spectrum of *N*-(3-chloro-4-fluorophenyl)-7-methoxy-6-(2-(morpholinooxy)ethoxy)quinazolin-4-amine (**6**)

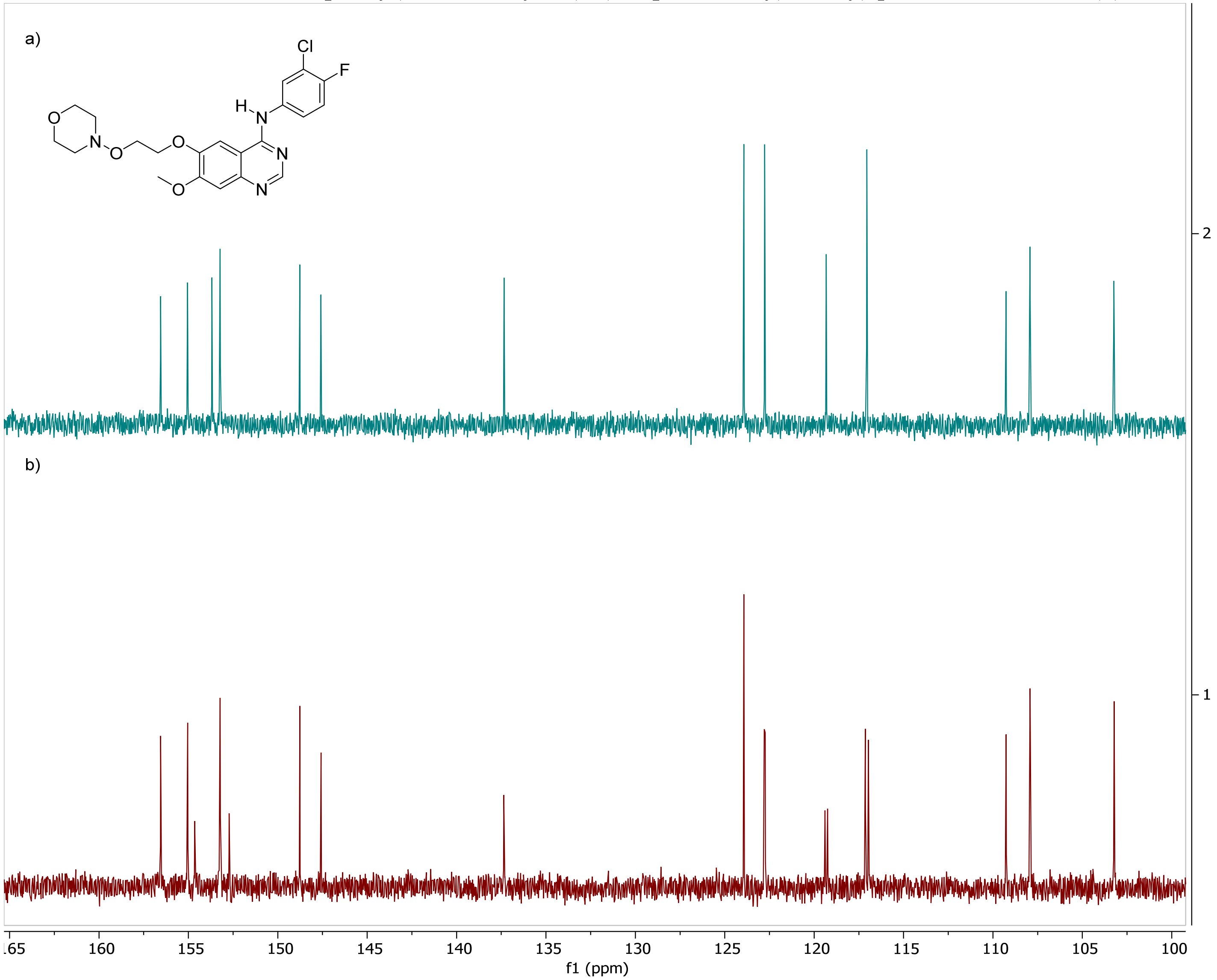

^{19}F NMR {^{1}H} (470 MHz, DMSO-D$_6$) spectrum of *N*-(3-chloro-4-fluorophenyl)-7-methoxy-6-(2-(morpholinooxy)ethoxy)quinazolin-4-amine (**6**)

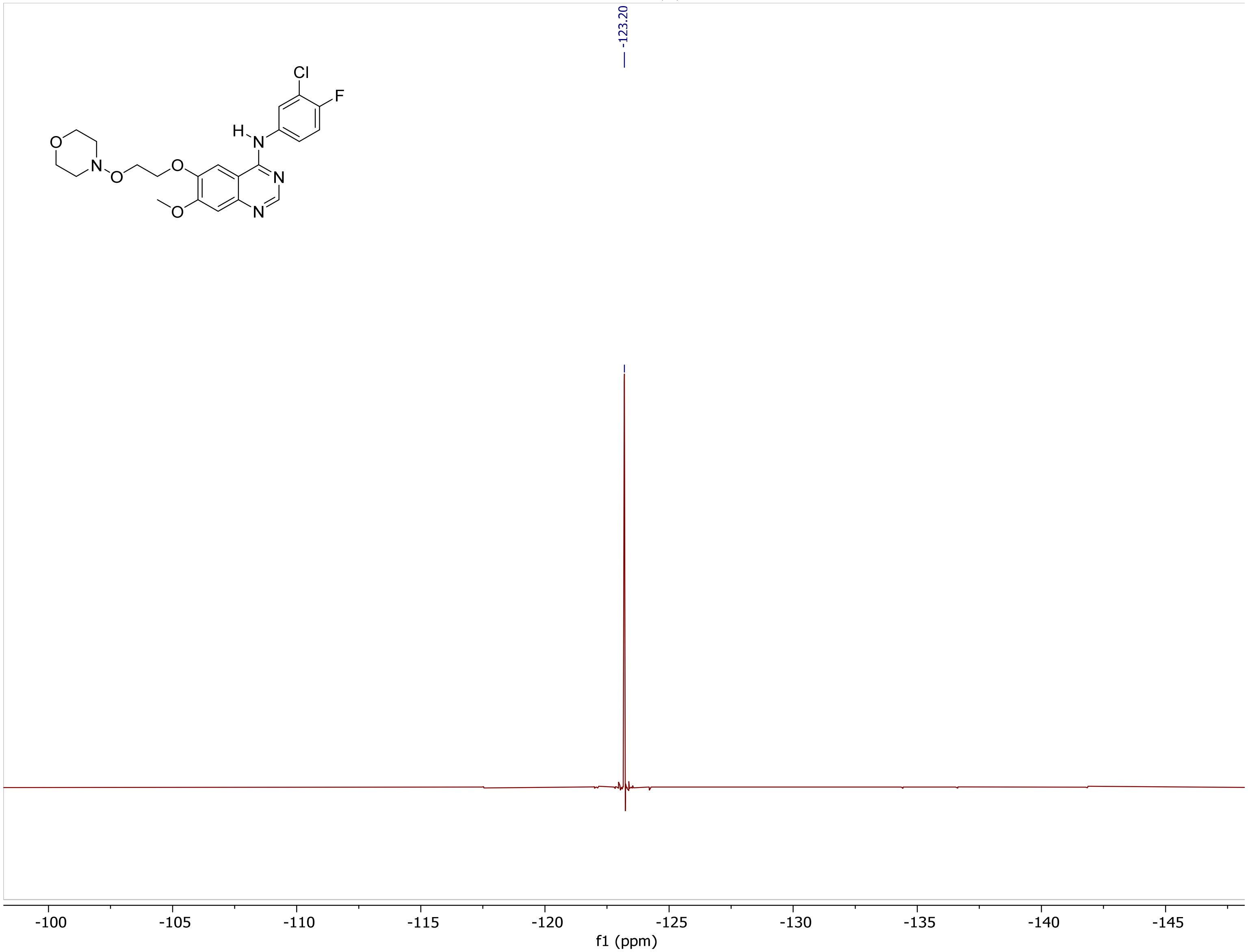

HSQC NMR (500 MHz, DMSO-D$_6$) spectrum of *N*-(3-chloro-4-fluorophenyl)-7-methoxy-6-(2-(morpholinooxy)ethoxy)quinazolin-4-amine (**6**)

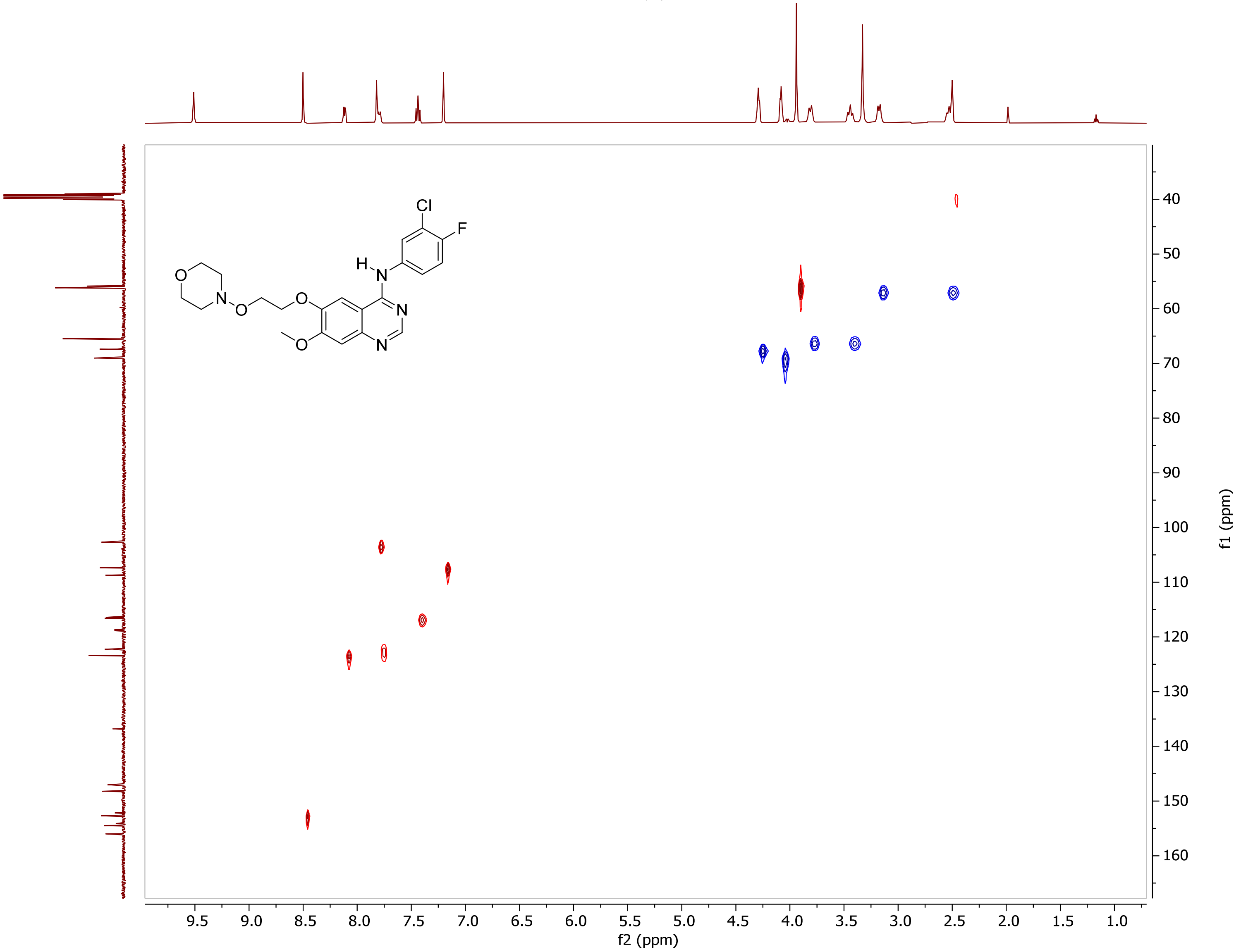

DQF COSY NMR (500 MHz, DMSO-D6) spectrum of *N*-(3-chloro-4-fluorophenyl)-7-methoxy-6-(2-(morpholinooxy)ethoxy)quinazolin-4-amine (**6**)

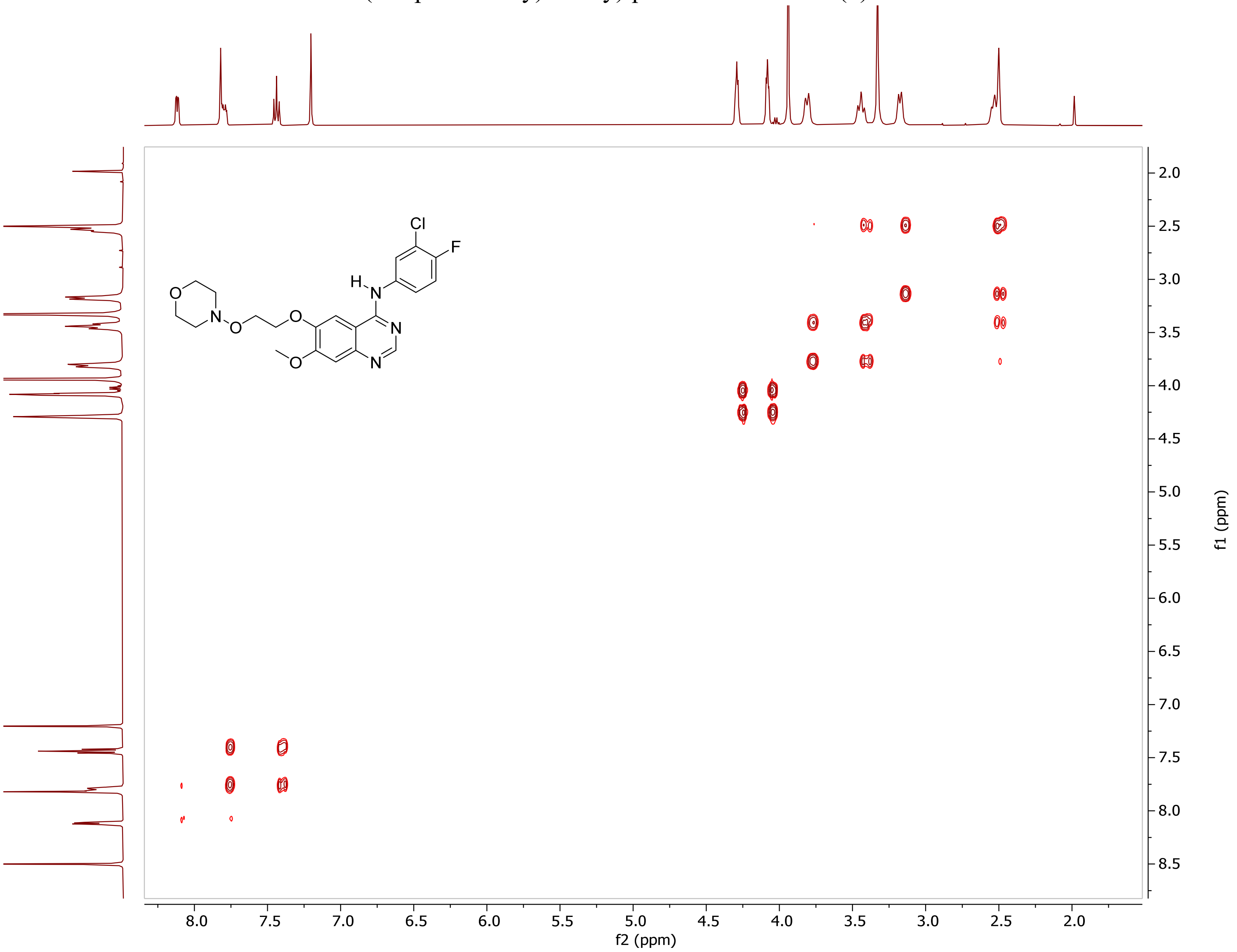

^{1}H NMR (500 MHz, DMSO-D6) spectrum of *N*-(3-chloro-2-fluorophenyl)-7-methoxy-6-(2-(morpholinooxy)ethoxy)quinazolin-4-amine (**7**)

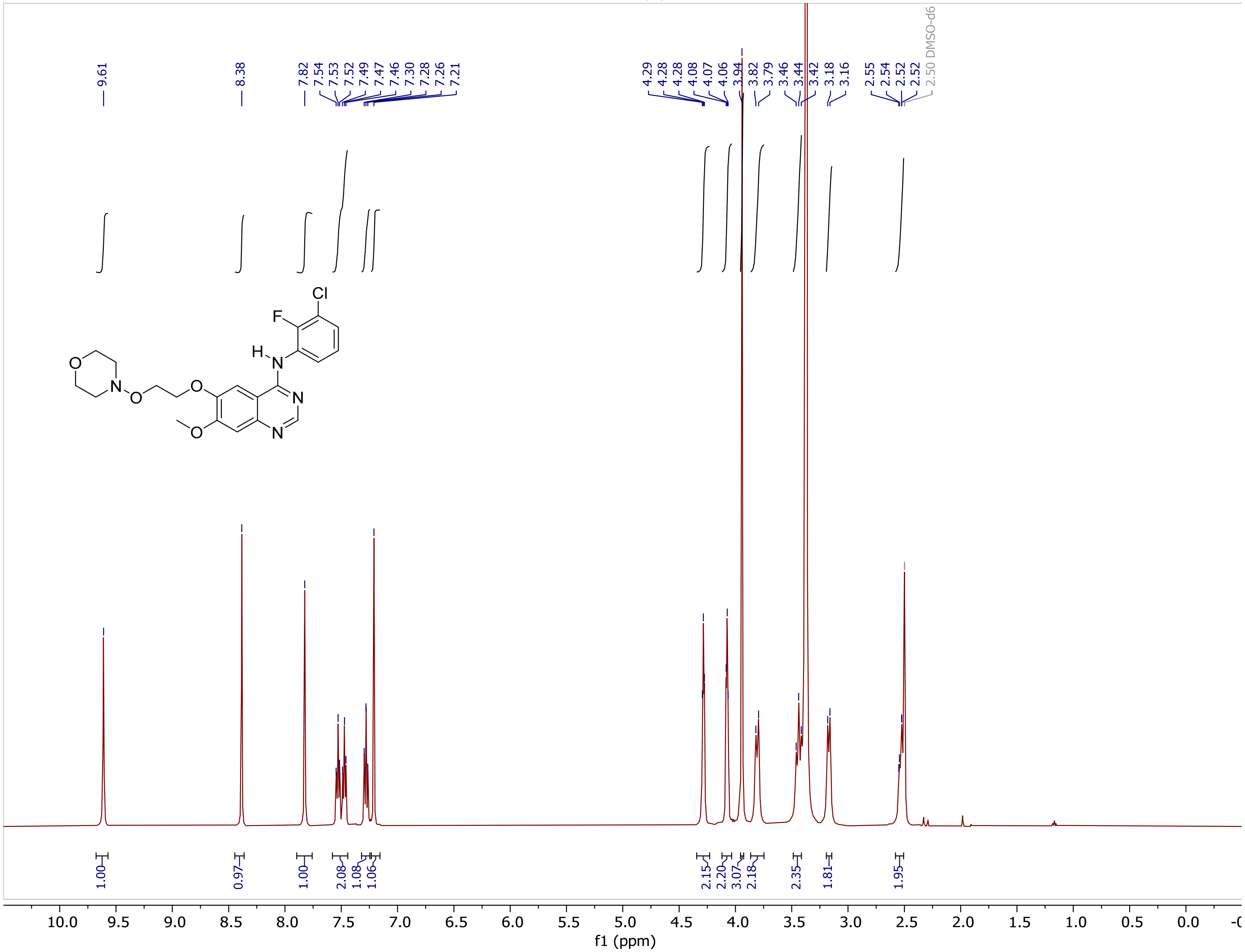

^{13}C NMR (126 MHz, DMSO-D$_6$) spectrum of *N*-(3-chloro-2-fluorophenyl)-7-methoxy-6-(2-(morpholinooxy)ethoxy)quinazolin-4-amine (**7**)

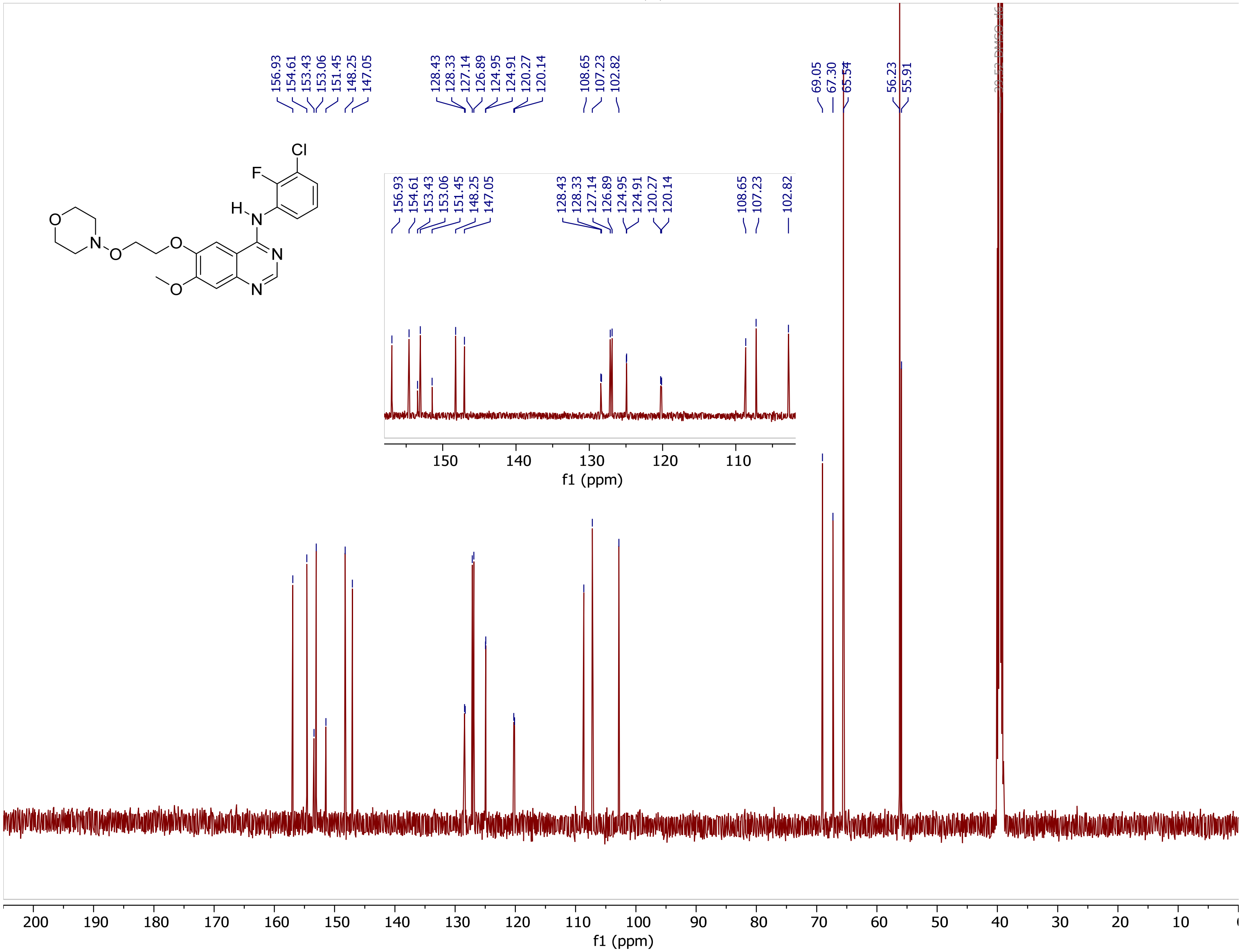

^{13}C NMR {^{19}F} (126 MHz, DMSO-D$_6$) spectrum of *N*-(3-chloro-2-fluorophenyl)-7-methoxy-6-(2-(morpholinooxy)ethoxy)quinazolin-4-amine (**7**)

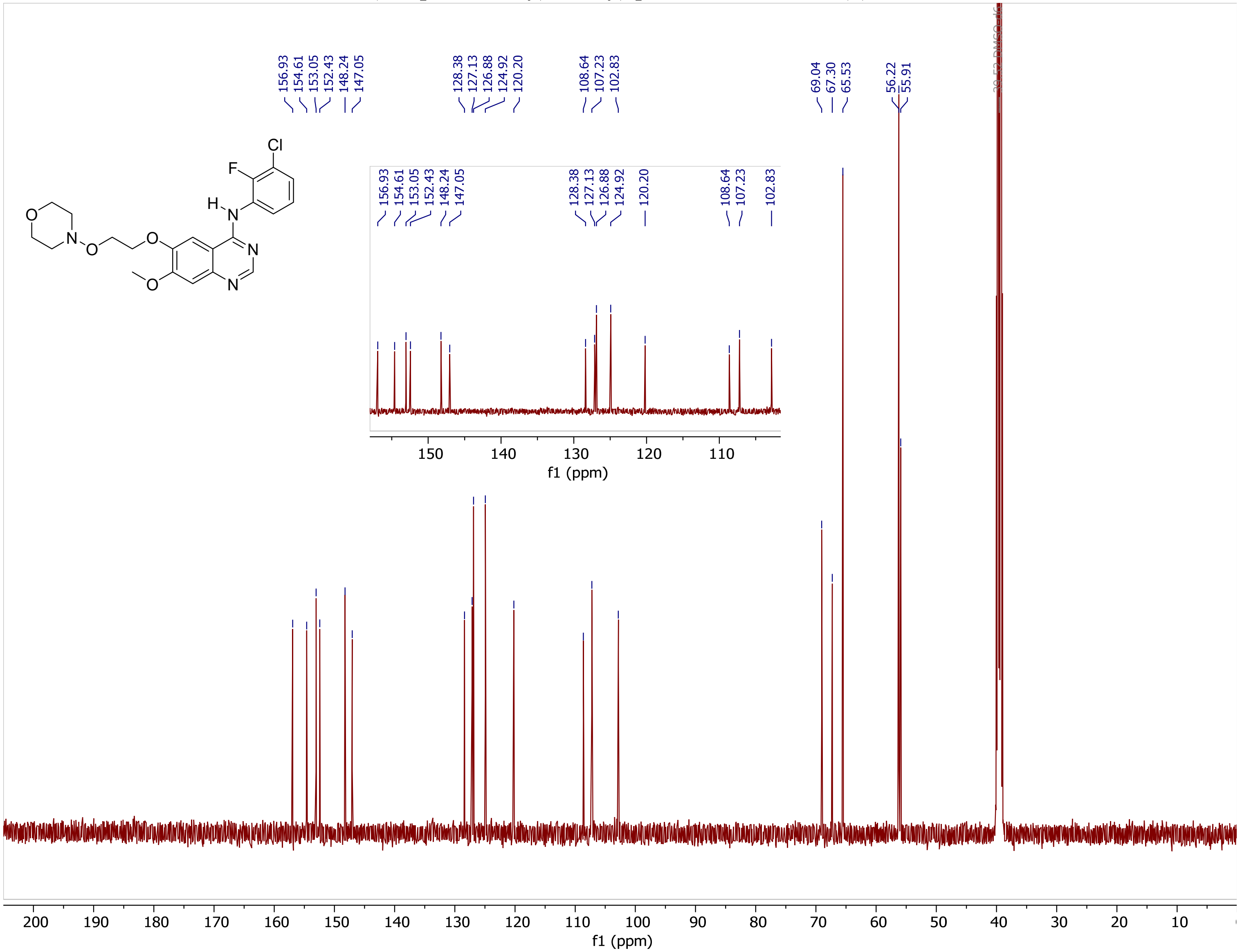

Expanded region of stacked a) **^{13}C NMR** {^{19}F} (126 MHz, DMSO-D$_6$) and b) **^{13}C NMR** (126 MHz, DMSO-D$_6$) spectrum of *N*-(3-chloro-2-fluorophenyl)-7-methoxy-6-(2-(morpholinooxy)ethoxy)quinazolin-4-amine (**7**)

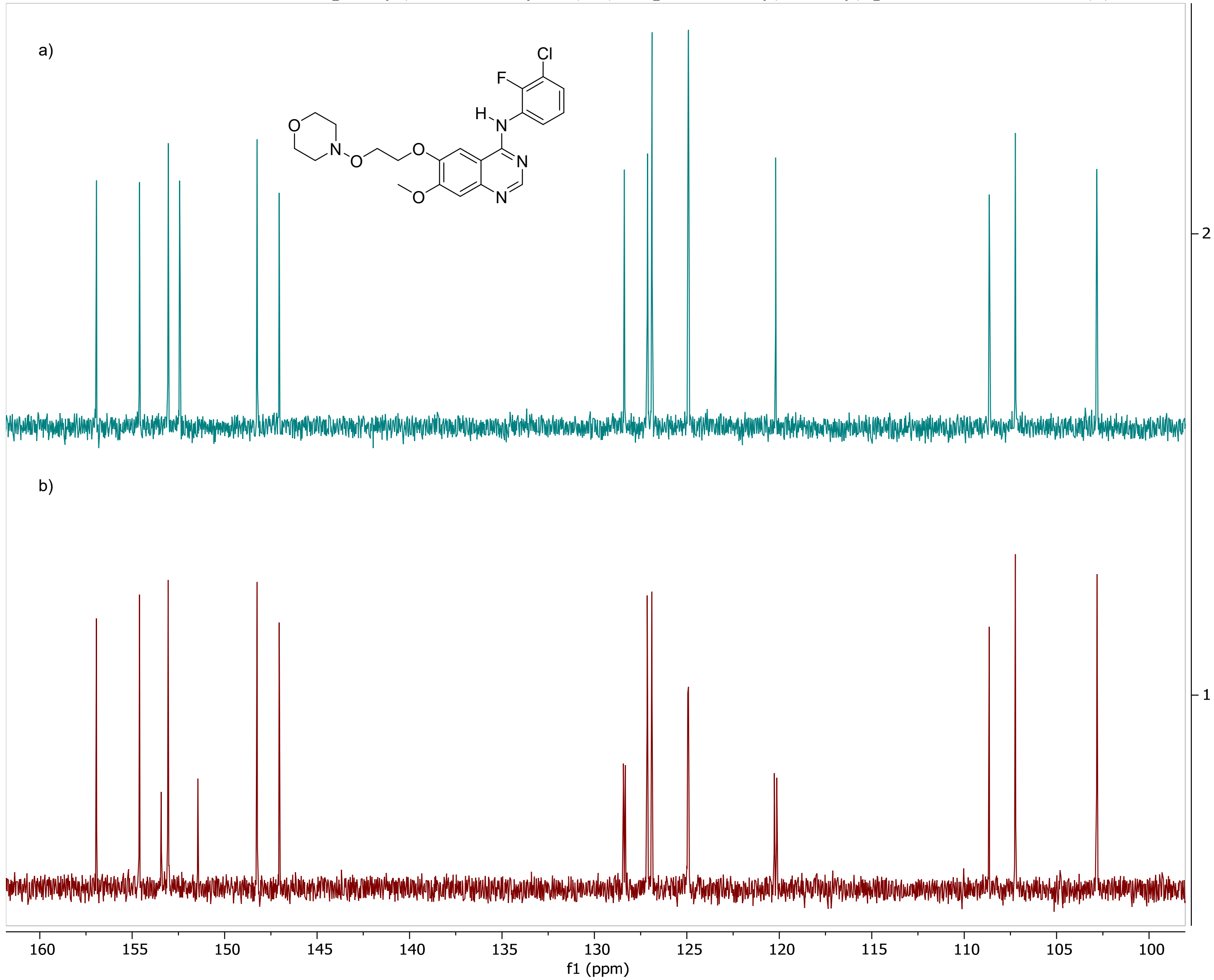

^{19}F NMR {^{1}H} (470 MHz, DMSO-D6) spectrum of *N*-(3-chloro-2-fluorophenyl)-7-methoxy-6-(2-(morpholinooxy)ethoxy)quinazolin-4-amine (**7**)

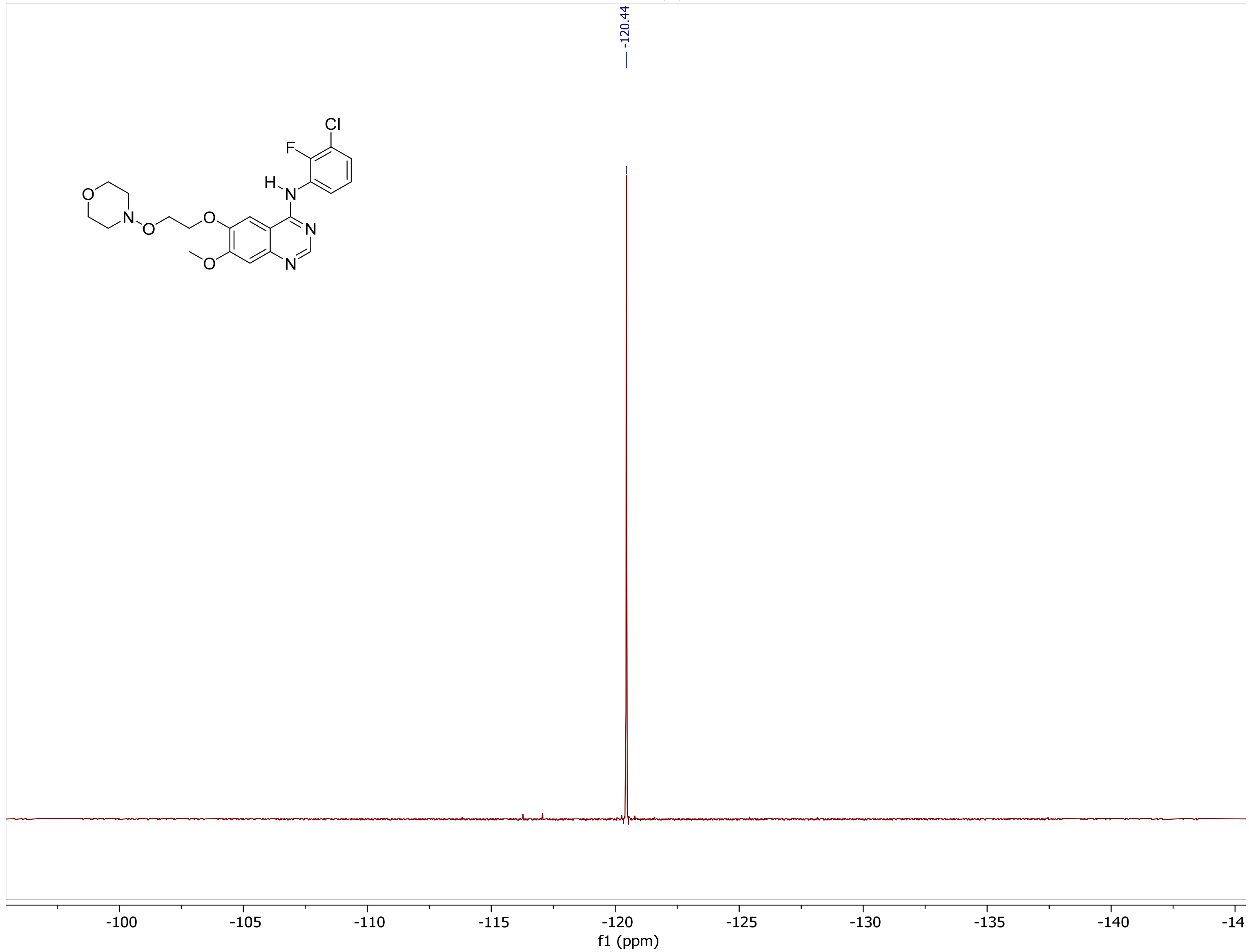

HSQC NMR (500 MHz, DMSO-D$_6$) spectrum of *N*-(3-chloro-2-fluorophenyl)-7-methoxy-6-(2-(morpholinooxy)ethoxy)quinazolin-4-amine (**7**)

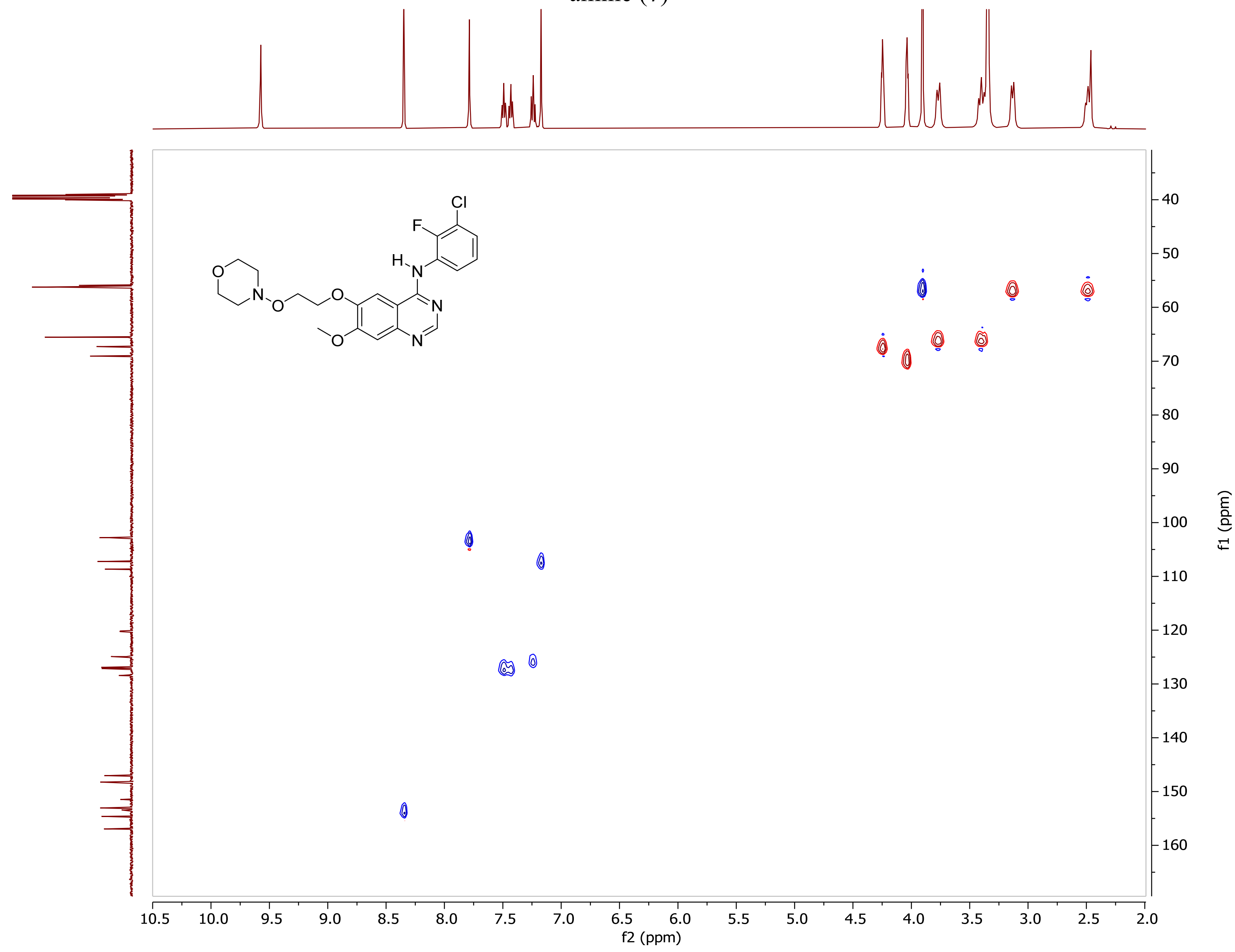

DQF COSY NMR (500 MHz, DMSO-D$_6$) spectrum of *N*-(3-chloro-2-fluorophenyl)-7-methoxy-6-(2-(morpholinooxy)ethoxy)quinazolin-4-amine (**7**)

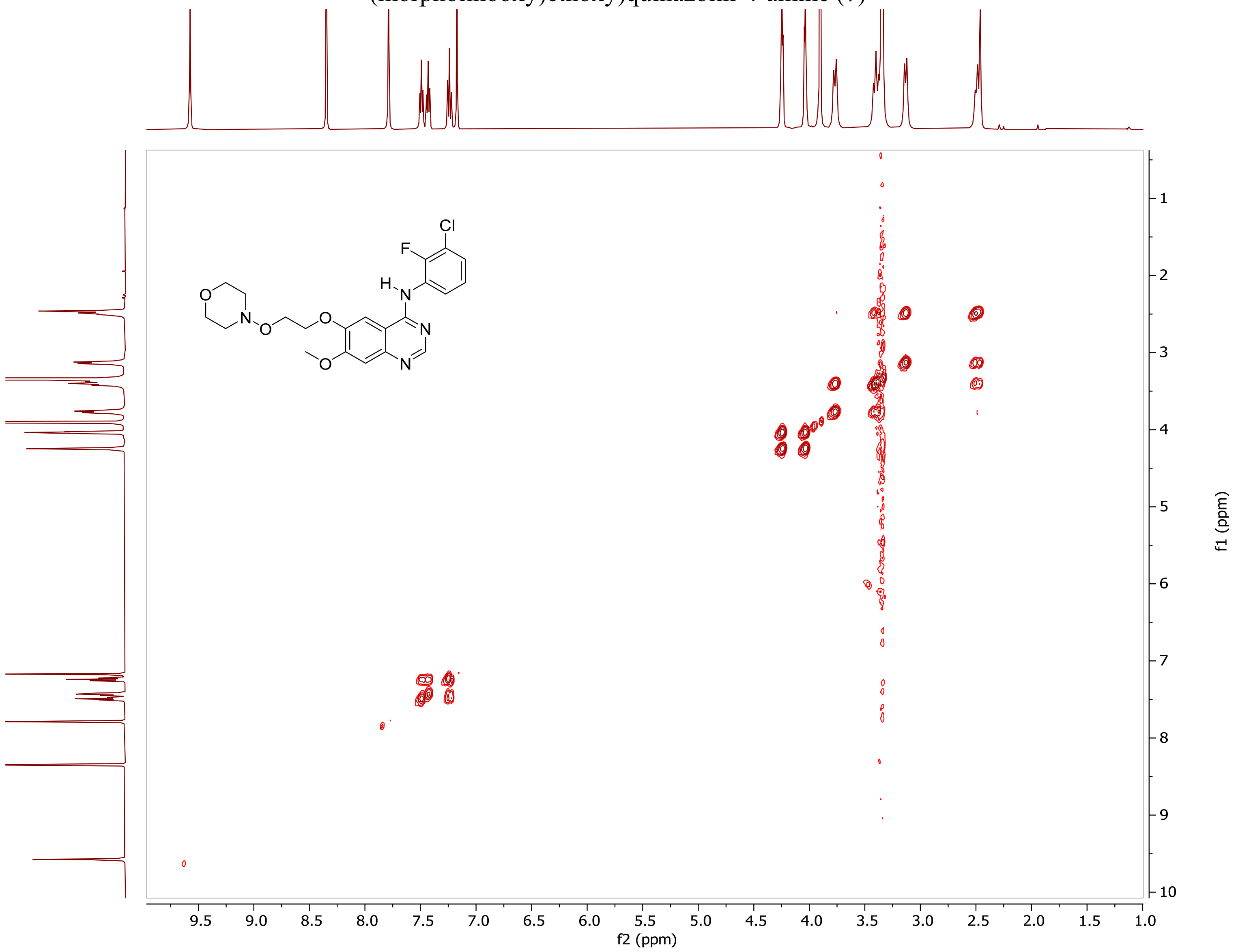

¹H NMR (500 MHz, DMSO-D$_6$) spectrum of *N*-(3-chloro-4-fluorophenyl)-7-methoxy-6-(2-((4-methylpiperazin-1-yl)oxy)ethoxy)quinazolin-4-amine (**8**)

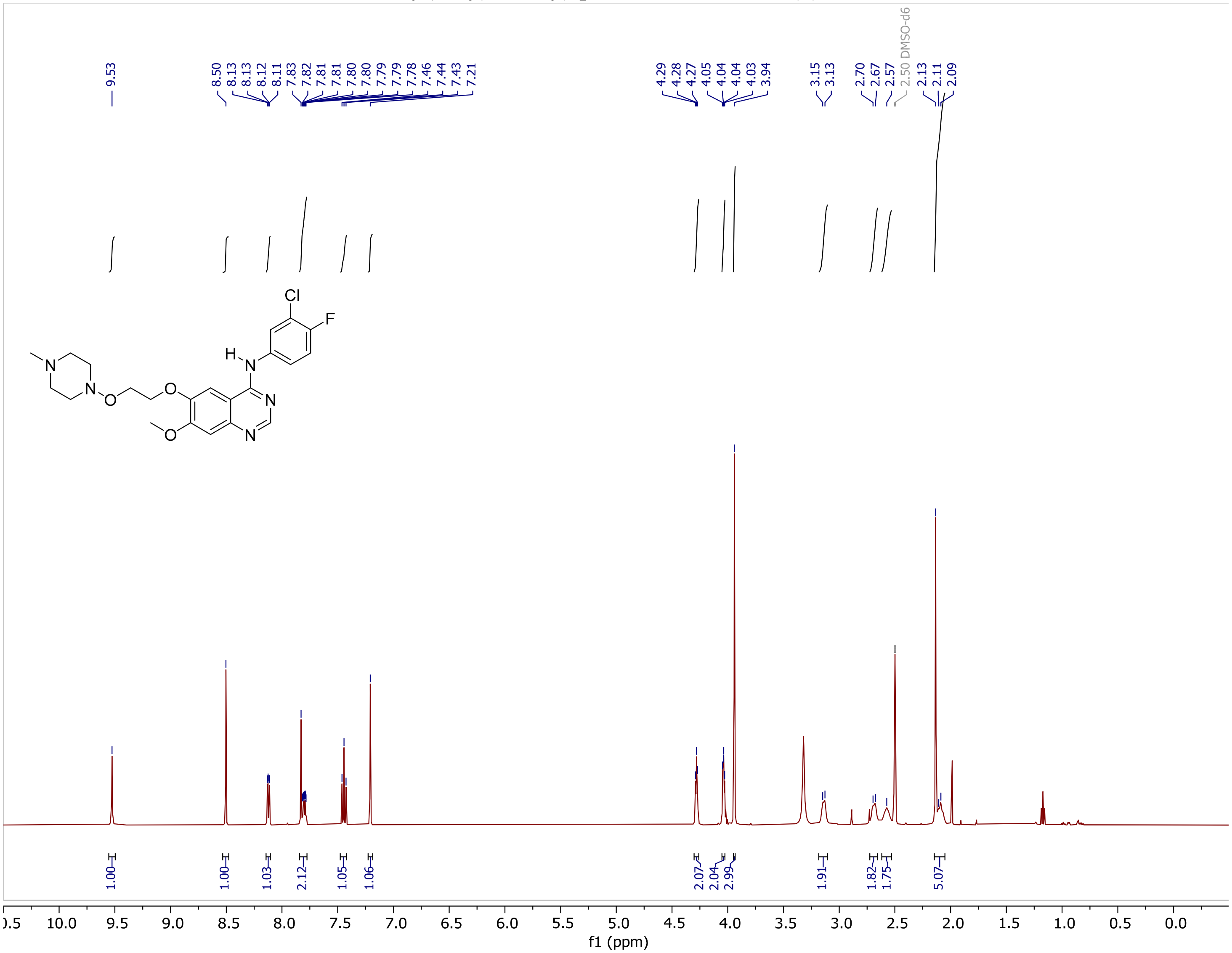

^{13}C NMR (126 MHz, DMSO-D$_6$) spectrum of *N*-(3-chloro-4-fluorophenyl)-7-methoxy-6-(2-((4-methylpiperazin-1-yl)oxy)ethoxy)quinazolin-4-amine (**8**)

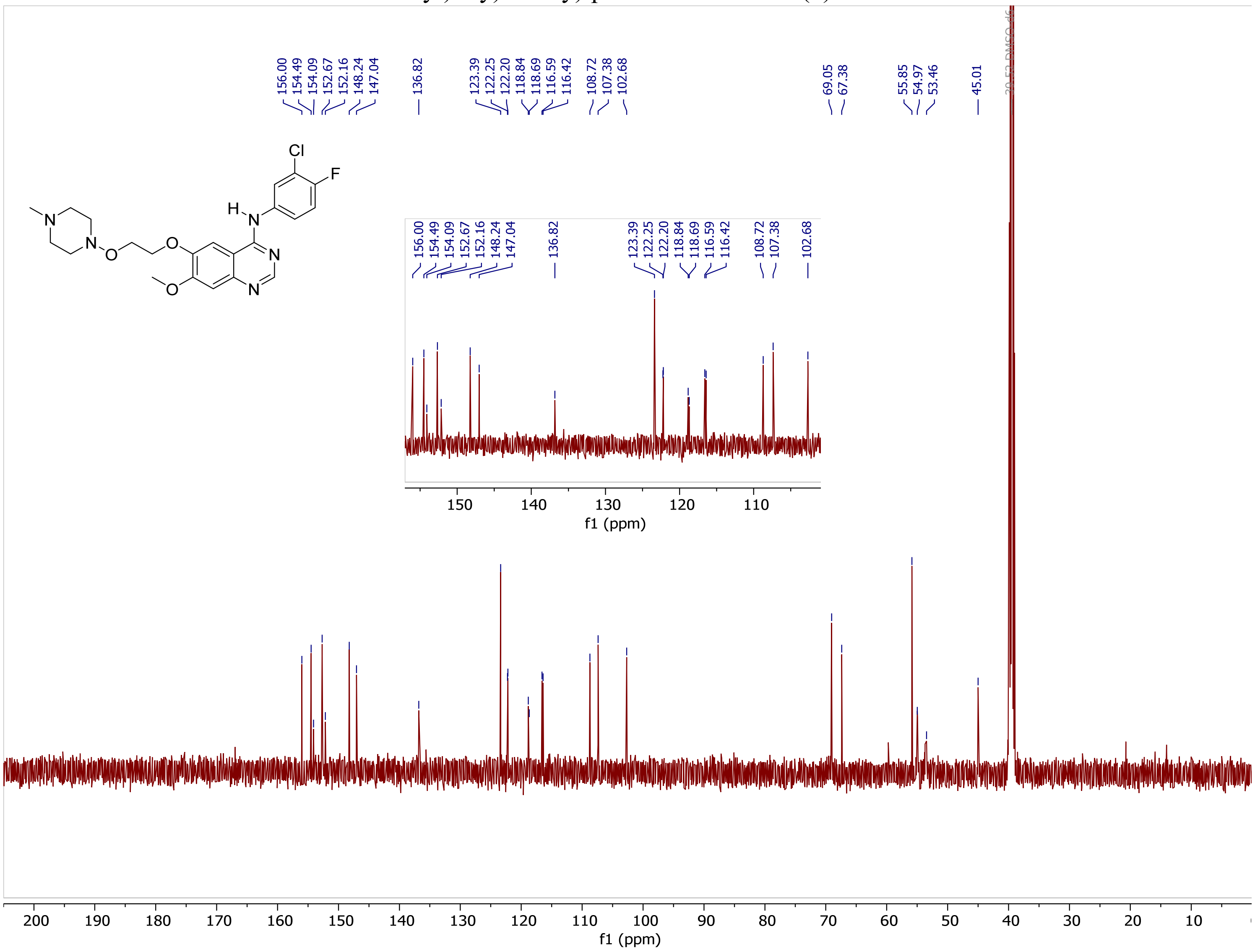

^{13}C NMR {^{19}F} (126 MHz, DMSO-D$_6$) spectrum of *N*-(3-chloro-4-fluorophenyl)-7-methoxy-6-(2-((4-methylpiperazin-1-yl)oxy)ethoxy)quinazolin-4-amine (**8**)

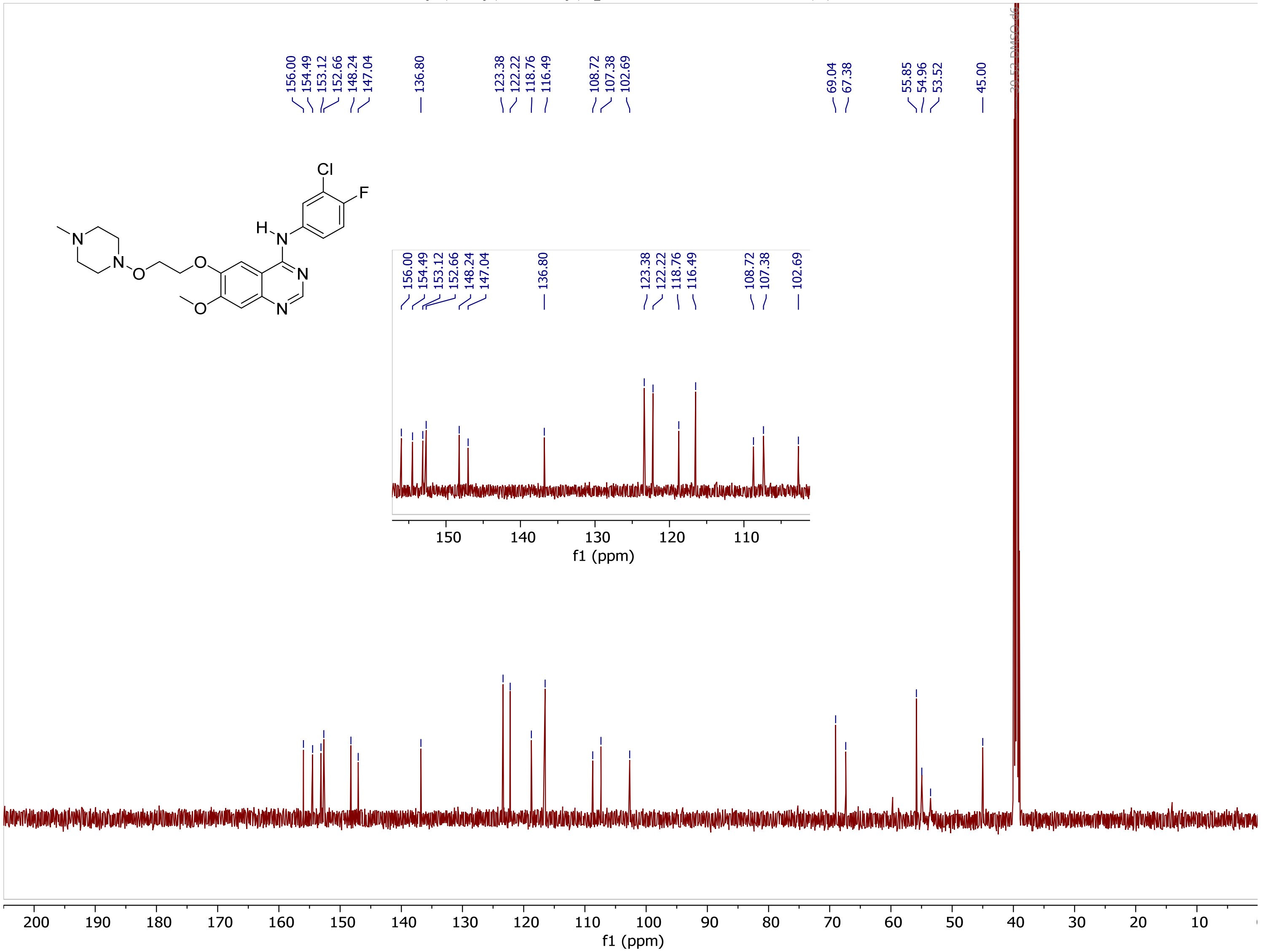

Expanded region of stacked a) **^{13}C NMR** {^{19}F} (126 MHz, DMSO-D$_6$) and b) **^{13}C NMR** (126 MHz, DMSO-D$_6$) spectrum of *N*-(3-chloro-4-fluorophenyl)-7-methoxy-6-(2-((4-methylpiperazin-1-yl)oxy)ethoxy)quinazolin-4-amine (**8**)

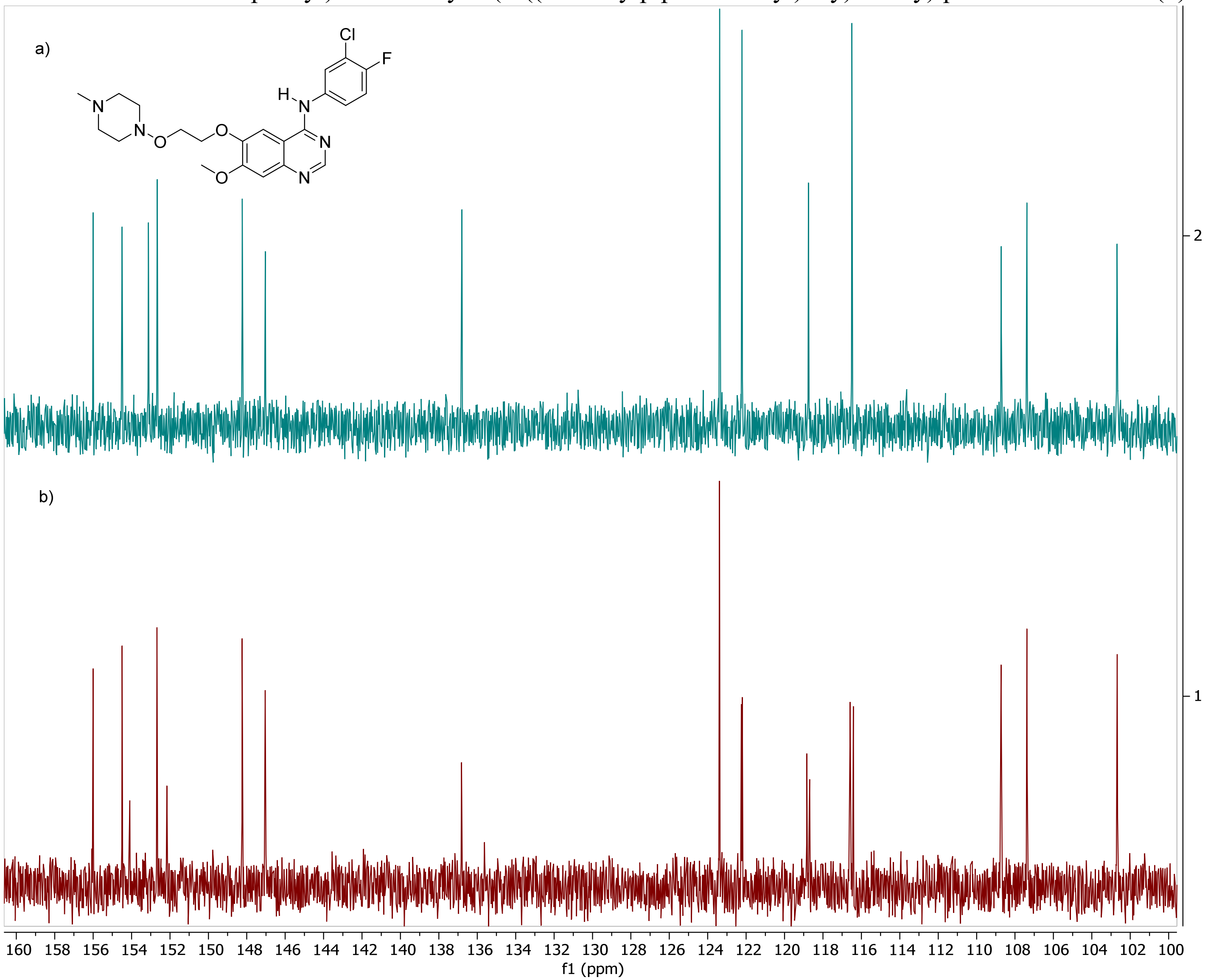

^{19}F NMR {^{1}H} (470 MHz, DMSO-D$_6$) spectrum of *N*-(3-chloro-4-fluorophenyl)-7-methoxy-6-(2-((4-methylpiperazin-1-yl)oxy)ethoxy)quinazolin-4-amine (**8**)

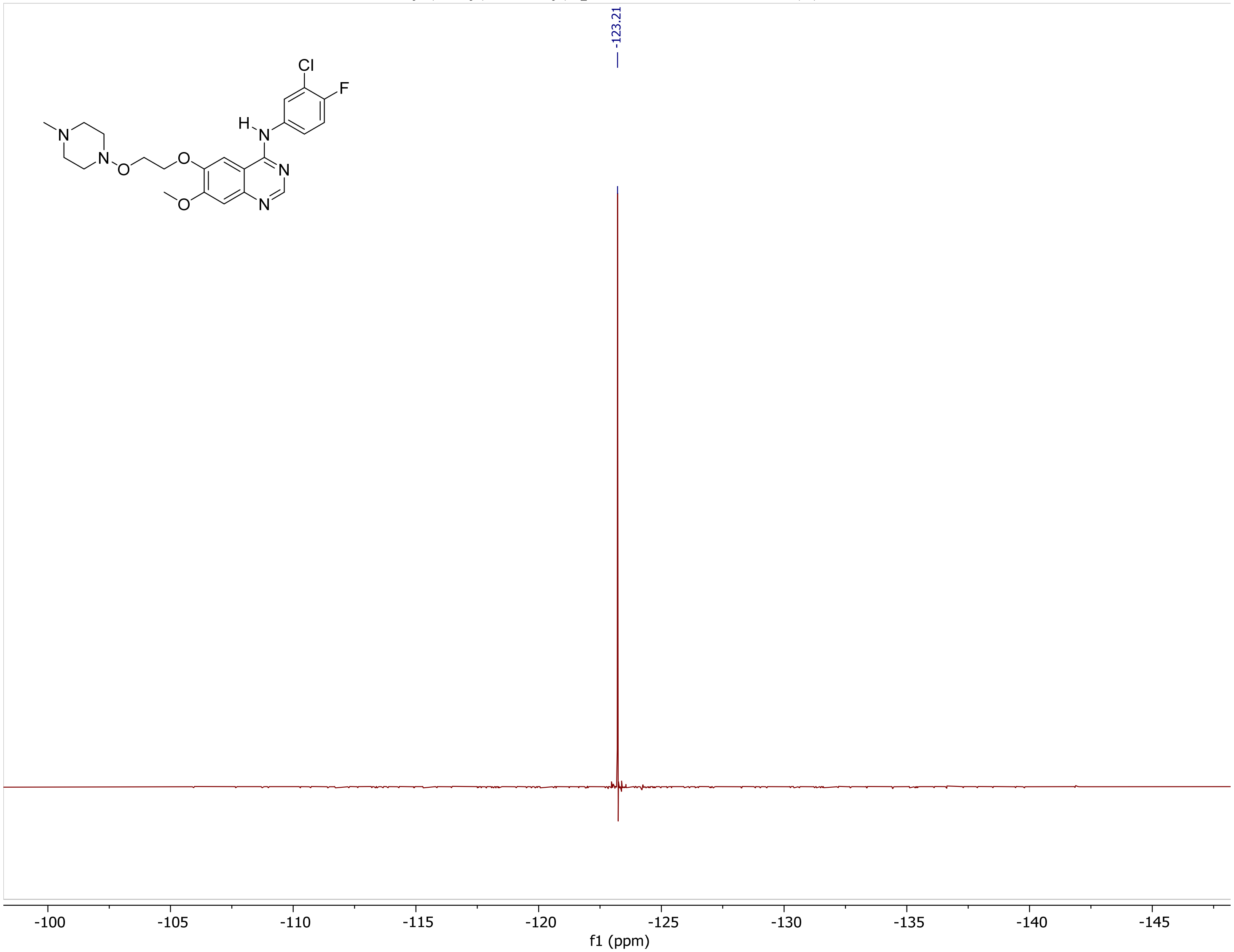

HSQC NMR (500 MHz, DMSO-D$_6$) spectrum of *N*-(3-chloro-4-fluorophenyl)-7-methoxy-6-(2-((4-methylpiperazin-1-yl)oxy)ethoxy)quinazolin-4-amine (**8**)

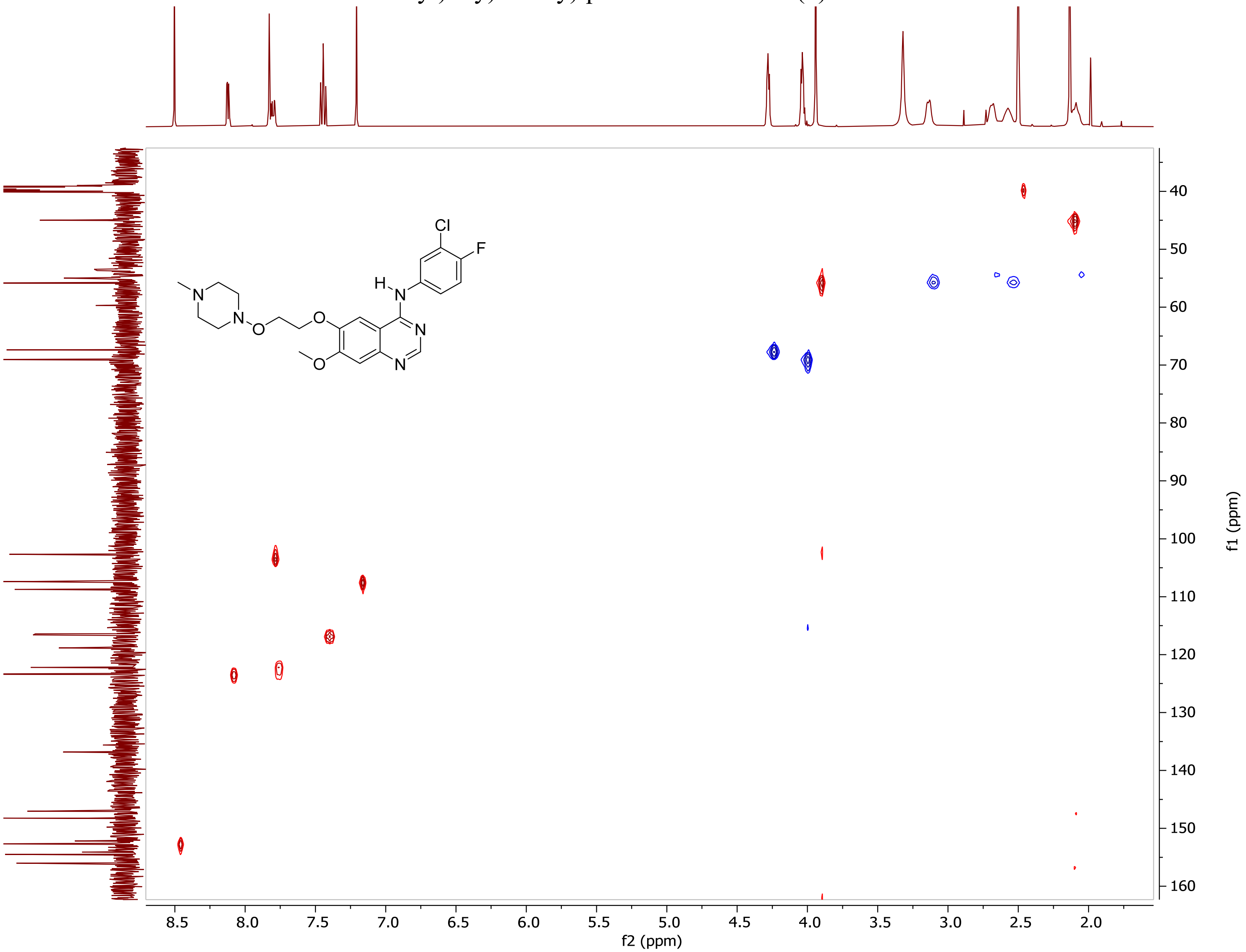

DQF COSY NMR (500 MHz, DMSO-D$_6$) spectrum of *N*-(3-chloro-4-fluorophenyl)-7-methoxy-6-(2-((4-methylpiperazin-1-yl)oxy)ethoxy)quinazolin-4-amine (**8**)

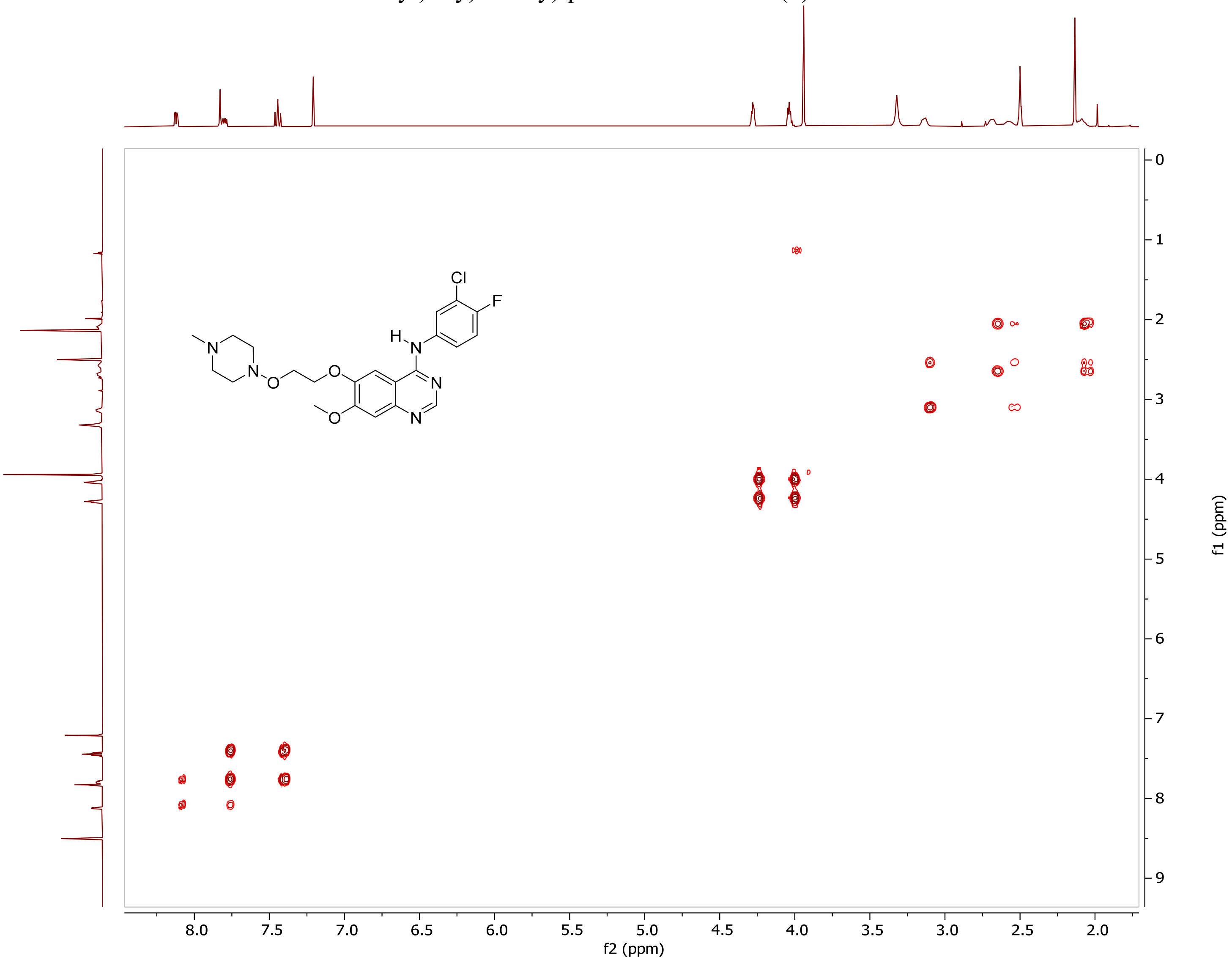

Expanded region of stacked **^{13}C NMR** (126 MHz, DMSO-D$_6$) of *N*-(3-chloro-4-fluorophenyl)-7-methoxy-6-(2-((4-methylpiperazin-1-yl)oxy)ethoxy)quinazolin-4-amine (**8**) at a) 358 K and b) 298 K

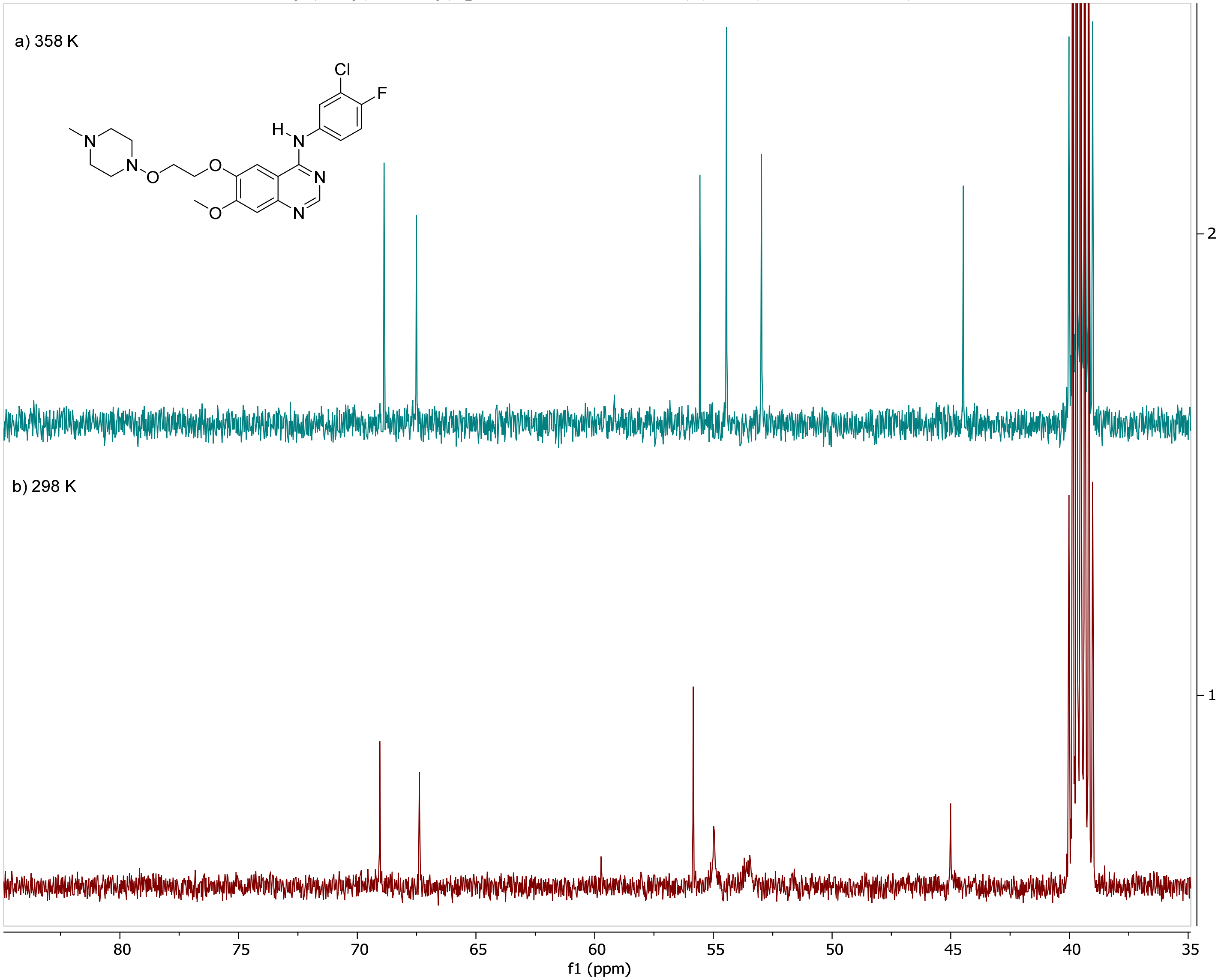

^{1}H NMR (500 MHz, DMSO-D$_6$) spectrum of *N*-(3-chloro-2-fluorophenyl)-7-methoxy-6-(2-((4-methylpiperazin-1-yl)oxy)ethoxy)quinazolin-4-amine (**9**)

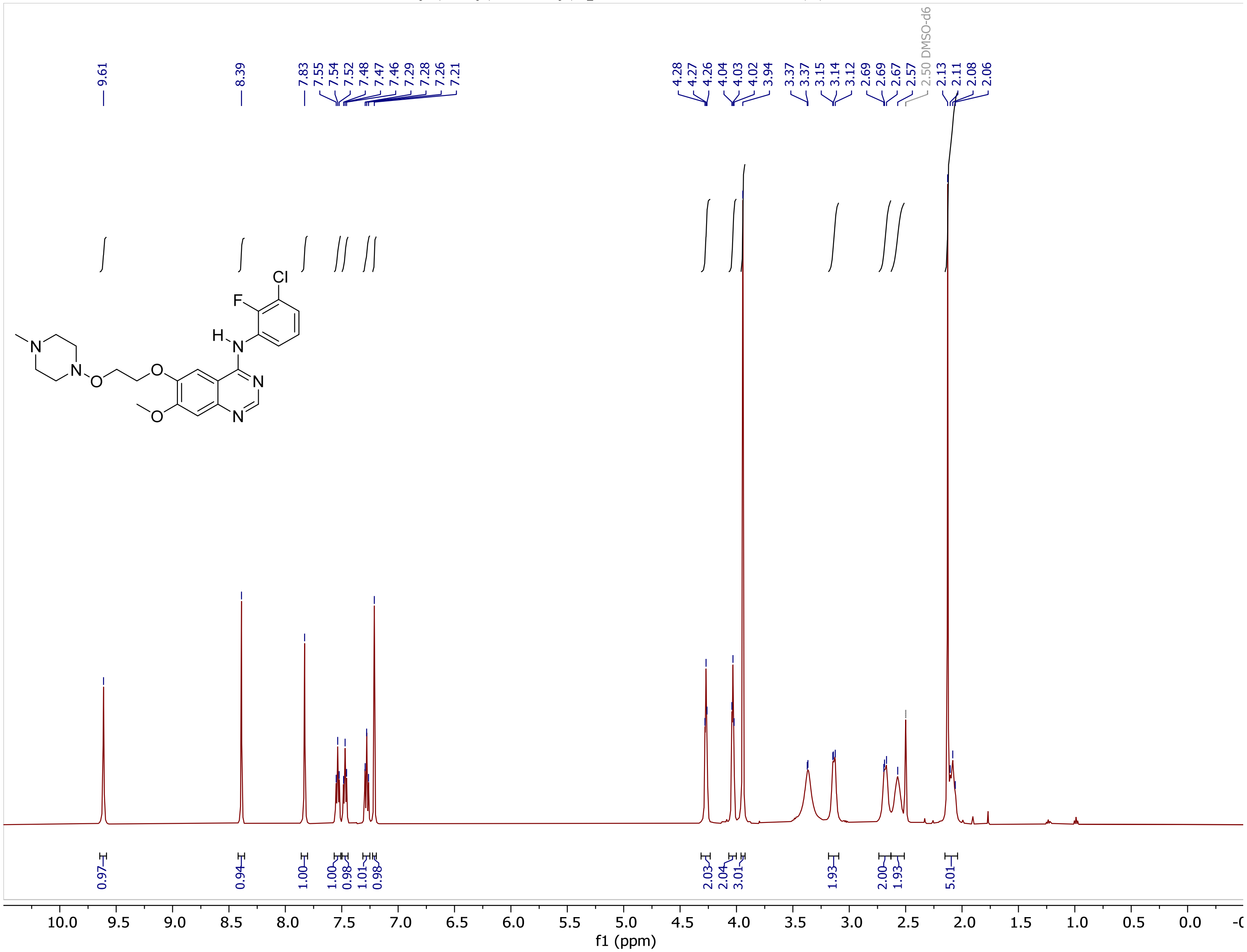

^{13}C NMR (126 MHz, DMSO-D6) spectrum of *N*-(3-chloro-2-fluorophenyl)-7-methoxy-6-(2-((4-methylpiperazin-1-yl)oxy)ethoxy)quinazolin-4-amine (**9**)

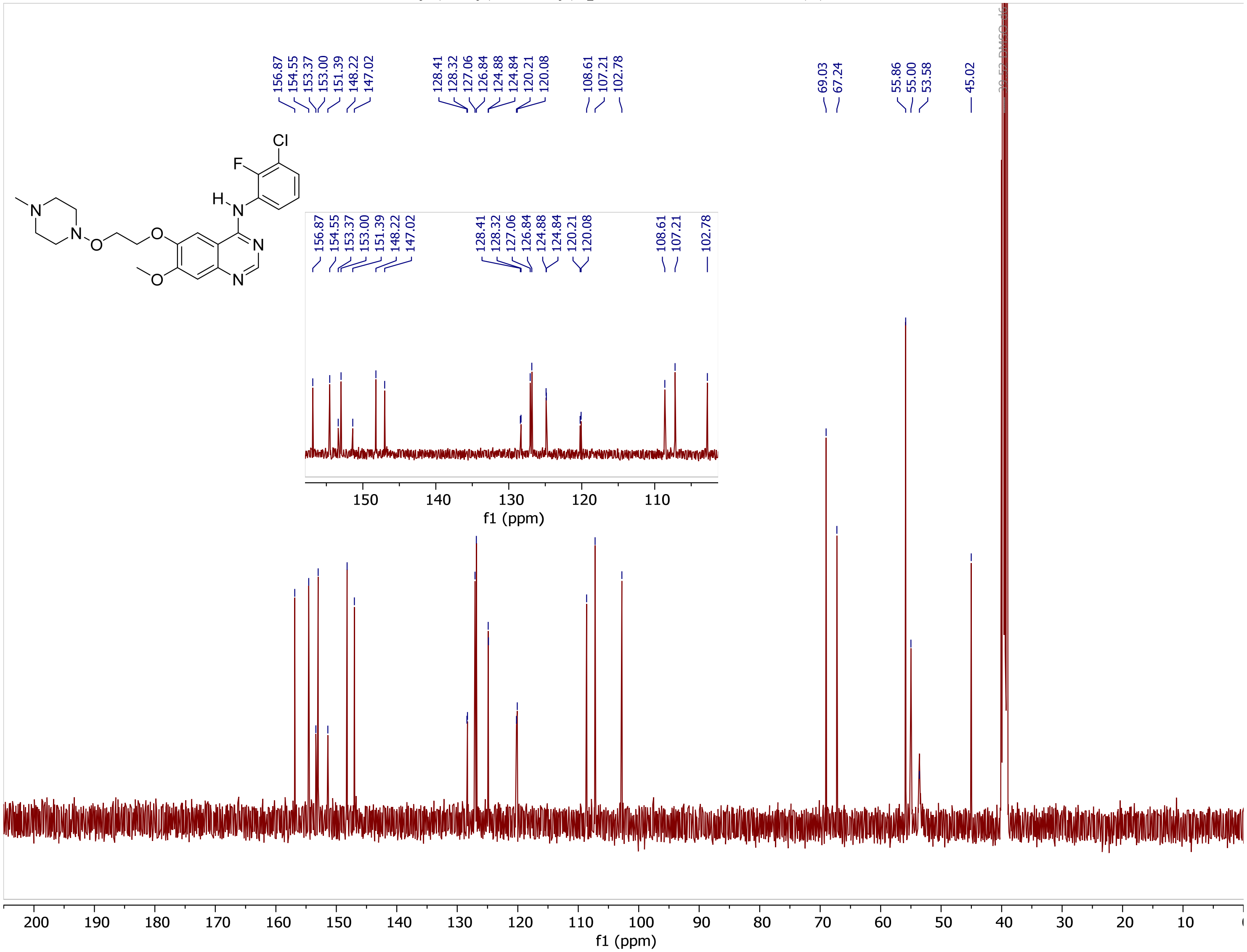

^{13}C NMR {^{19}F} (126 MHz, DMSO-D$_6$) spectrum of *N*-(3-chloro-2-fluorophenyl)-7-methoxy-6-(2-((4-methylpiperazin-1-yl)oxy)ethoxy)quinazolin-4-amine (**9**)

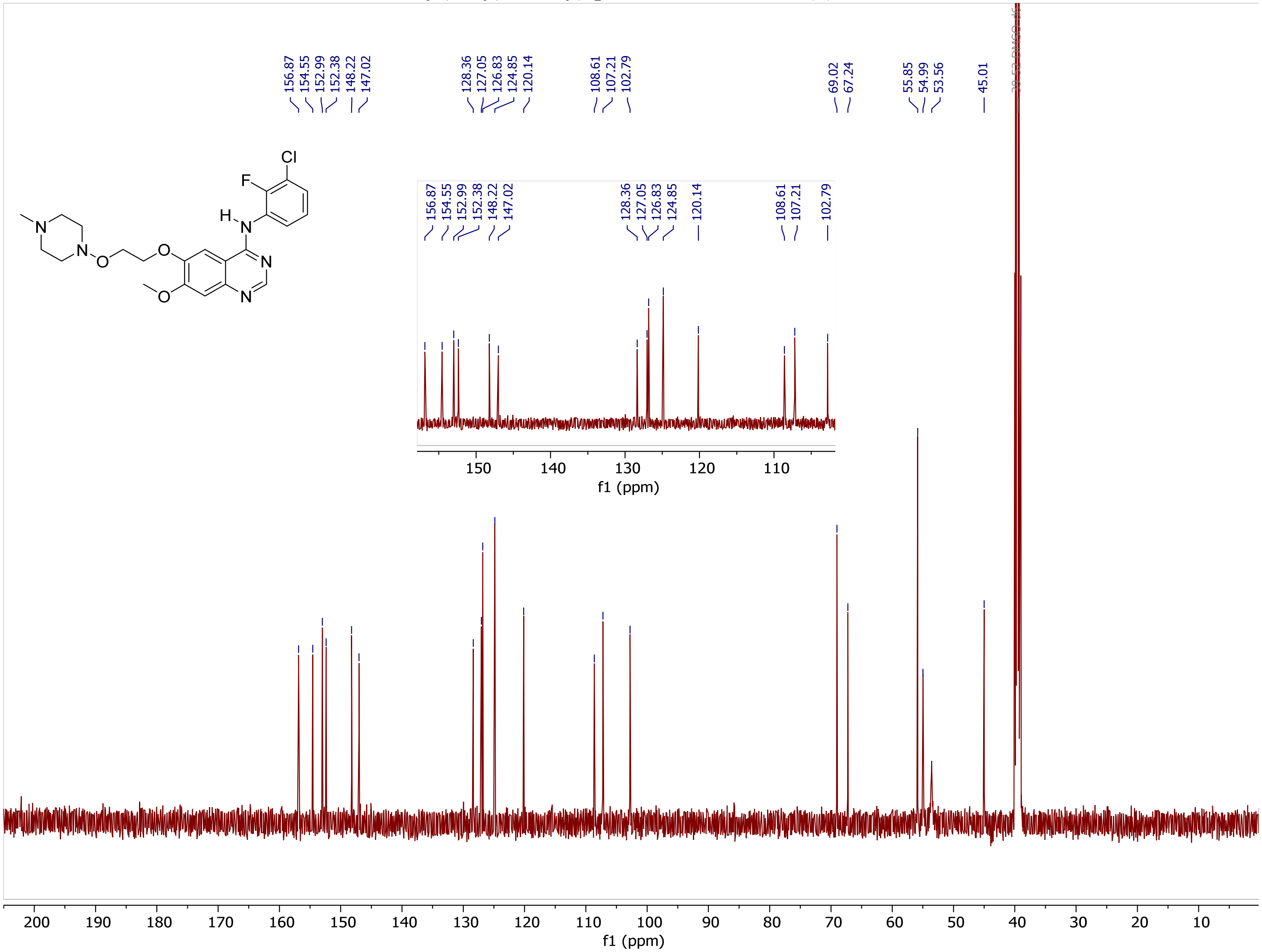

Expanded region of stacked a) **^{13}C NMR** {^{19}F} (126 MHz, DMSO-D$_6$) and b) **^{13}C NMR** (126 MHz, DMSO-D$_6$) spectrum of *N*-(3-chloro-2-fluorophenyl)-7-methoxy-6-(2-((4-methylpiperazin-1-yl)oxy)ethoxy)quinazolin-4-amine (**9**)

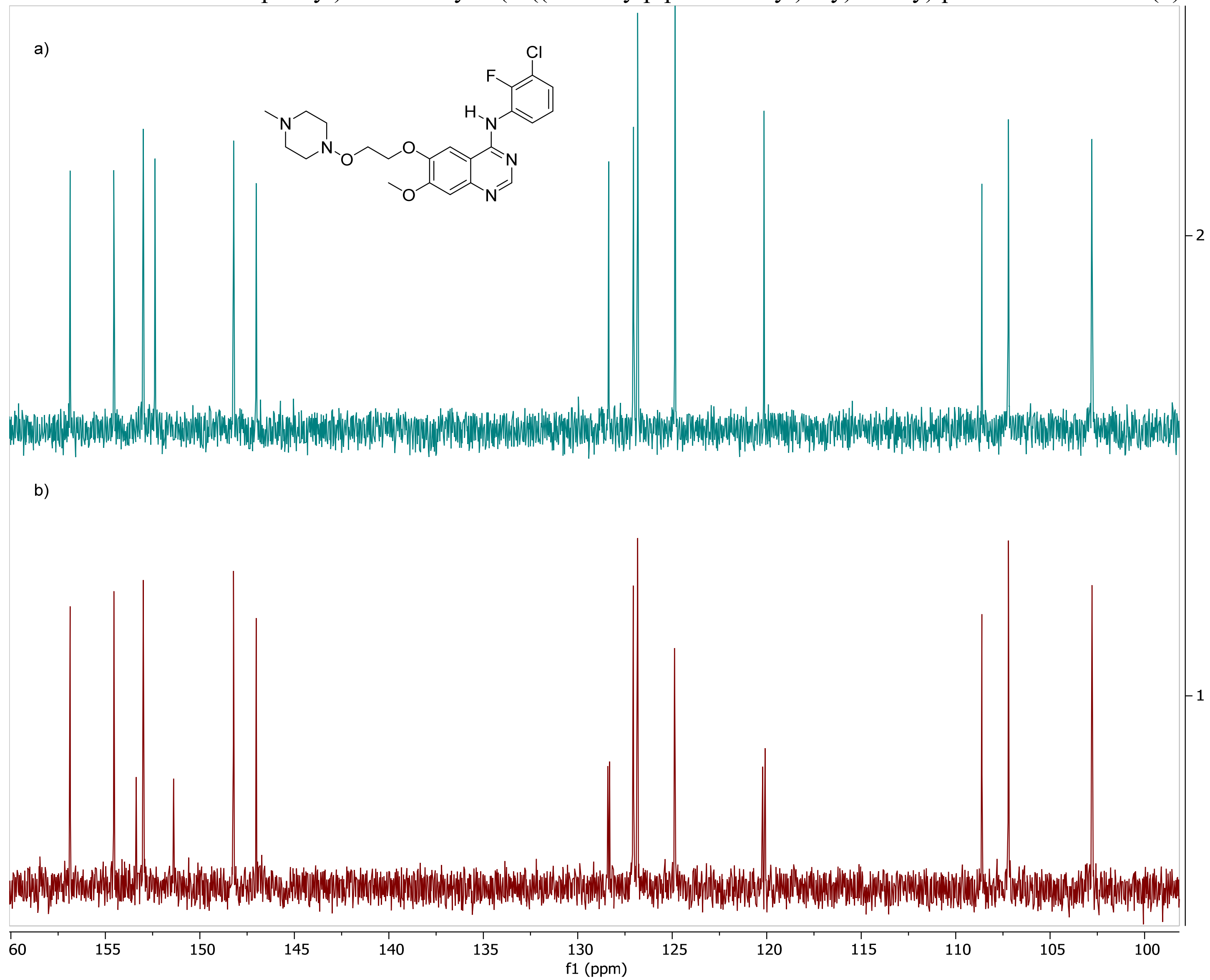

^{19}F NMR {^{1}H} (470 MHz, DMSO-D6) spectrum of *N*-(3-chloro-2-fluorophenyl)-7-methoxy-6-(2-((4-methylpiperazin-1-yl)oxy)ethoxy)quinazolin-4-amine (**9**)

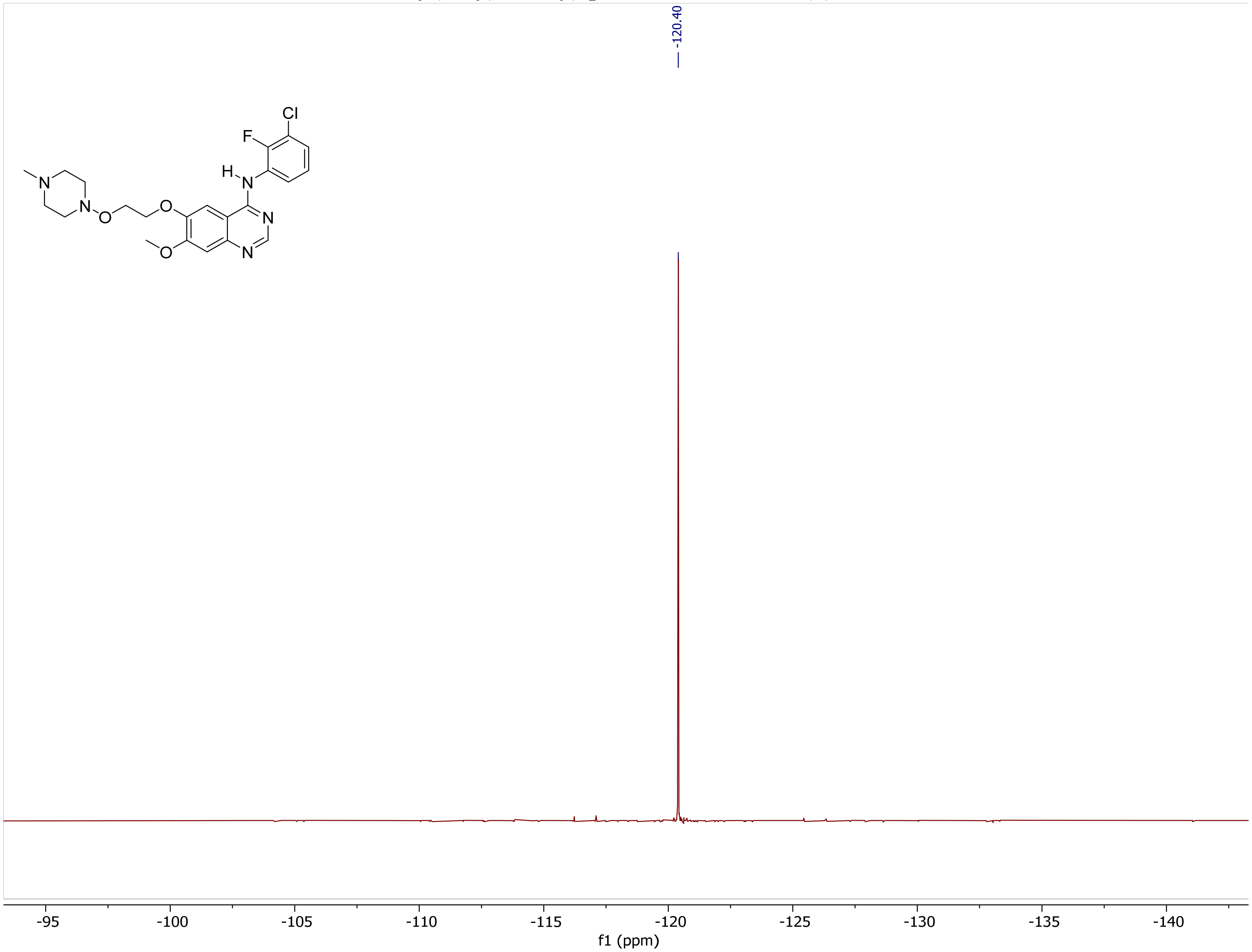

HSQC NMR (500 MHz, DMSO-D6) spectrum of *N*-(3-chloro-2-fluorophenyl)-7-methoxy-6-(2-((4-methylpiperazin-1-yl)oxy)ethoxy)quinazolin-4-amine (**9**)

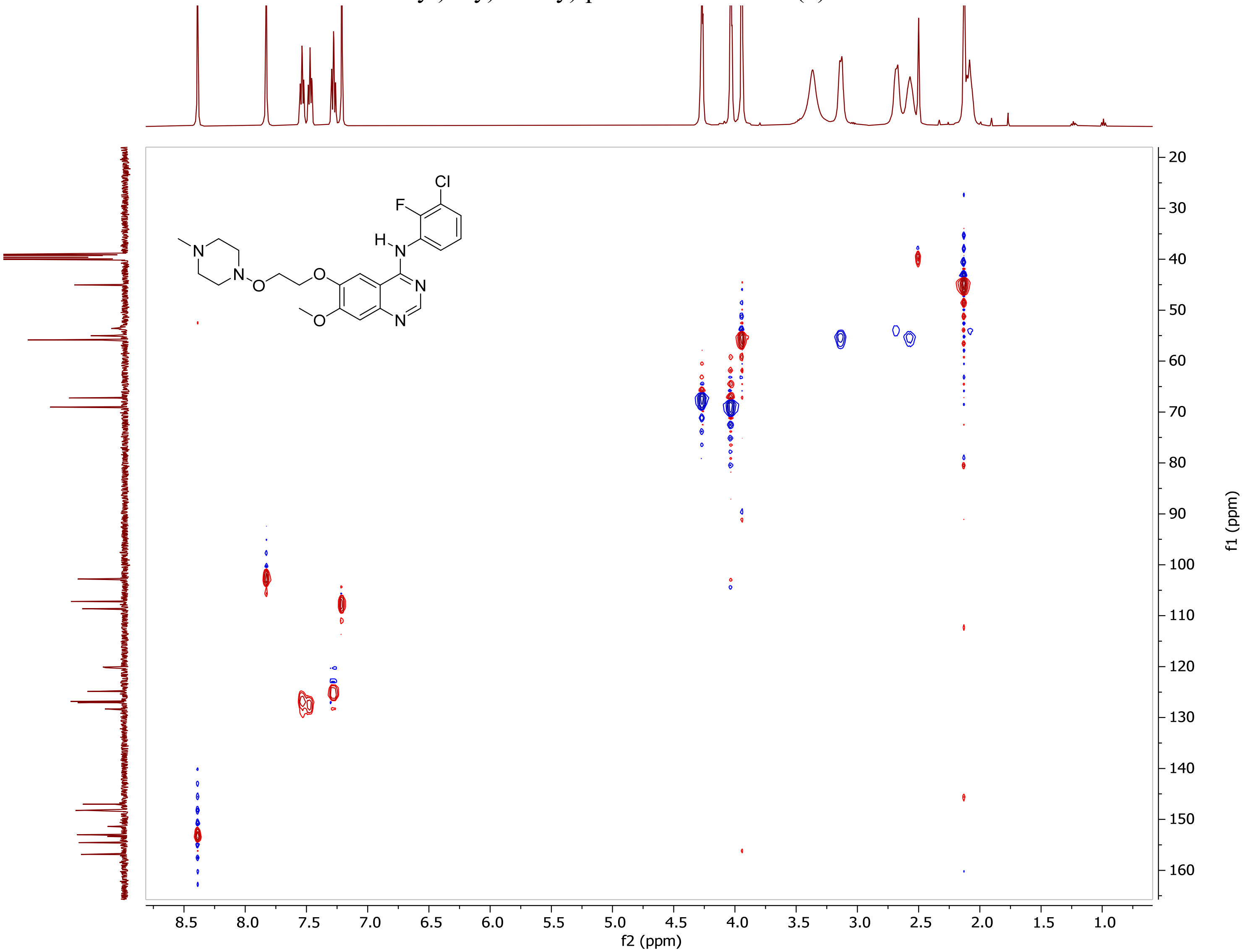

DQF COSY NMR (500 MHz, DMSO-D$_6$) spectrum of *N*-(3-chloro-2-fluorophenyl)-7-methoxy-6-(2-((4-methylpiperazin-1-yl)oxy)ethoxy)quinazolin-4-amine (**9**)

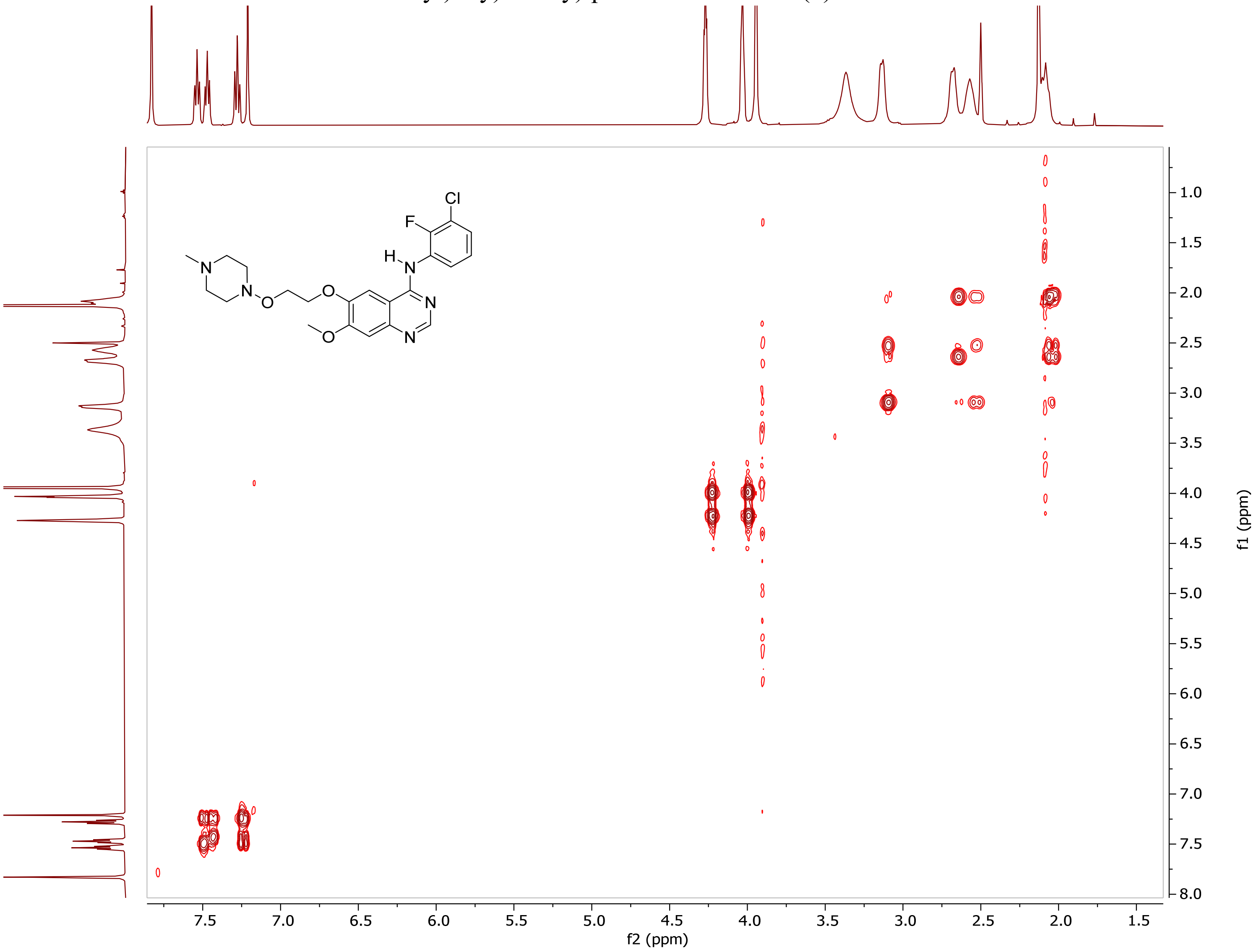

Expanded region of stacked ^{13}**C NMR** (126 MHz, DMSO-D$_6$) of *N*-(3-chloro-2-fluorophenyl)-7-methoxy-6-(2-((4-methylpiperazin-1-yl)oxy)ethoxy)quinazolin-4-amine (**9**) at a) 358 K and b) 298 K

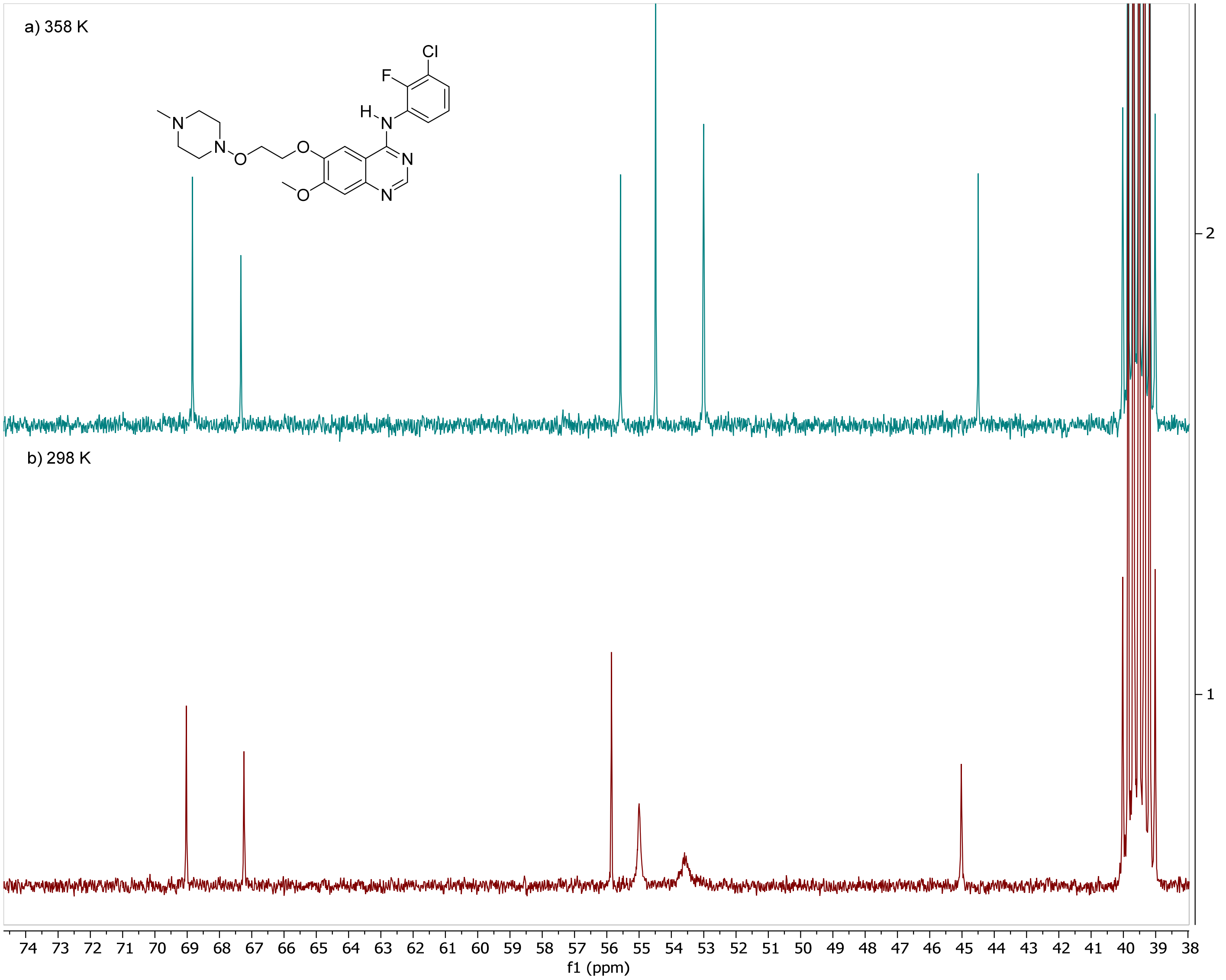

^{1}H NMR (500 MHz, $CDCl_3$) spectrum of benzyl 4-allylpiperazine-1-carboxylate (**21**).

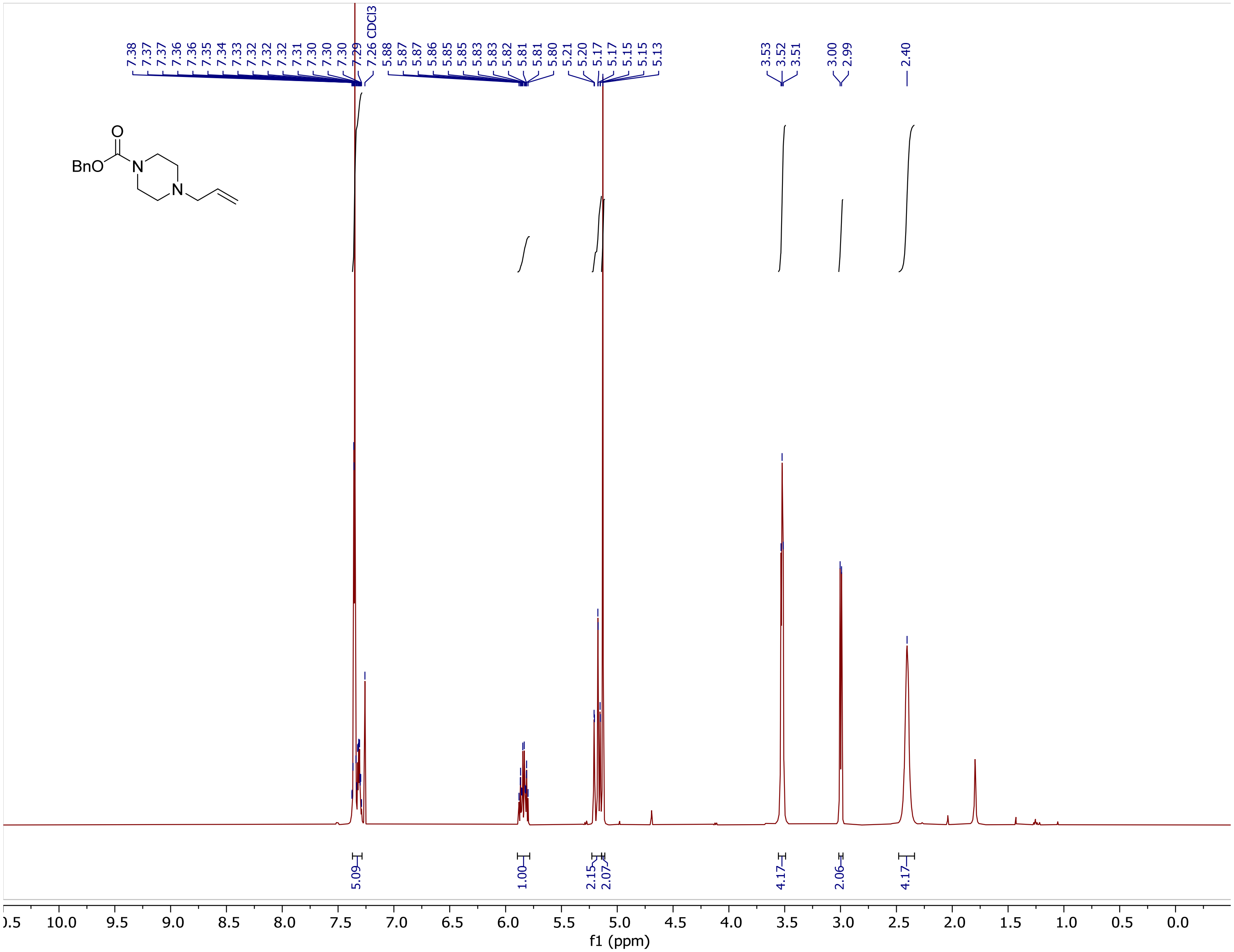

^{13}C NMR (126 MHz, $CDCl_3$) spectrum of benzyl 4-allylpiperazine-1-carboxylate (**21**).

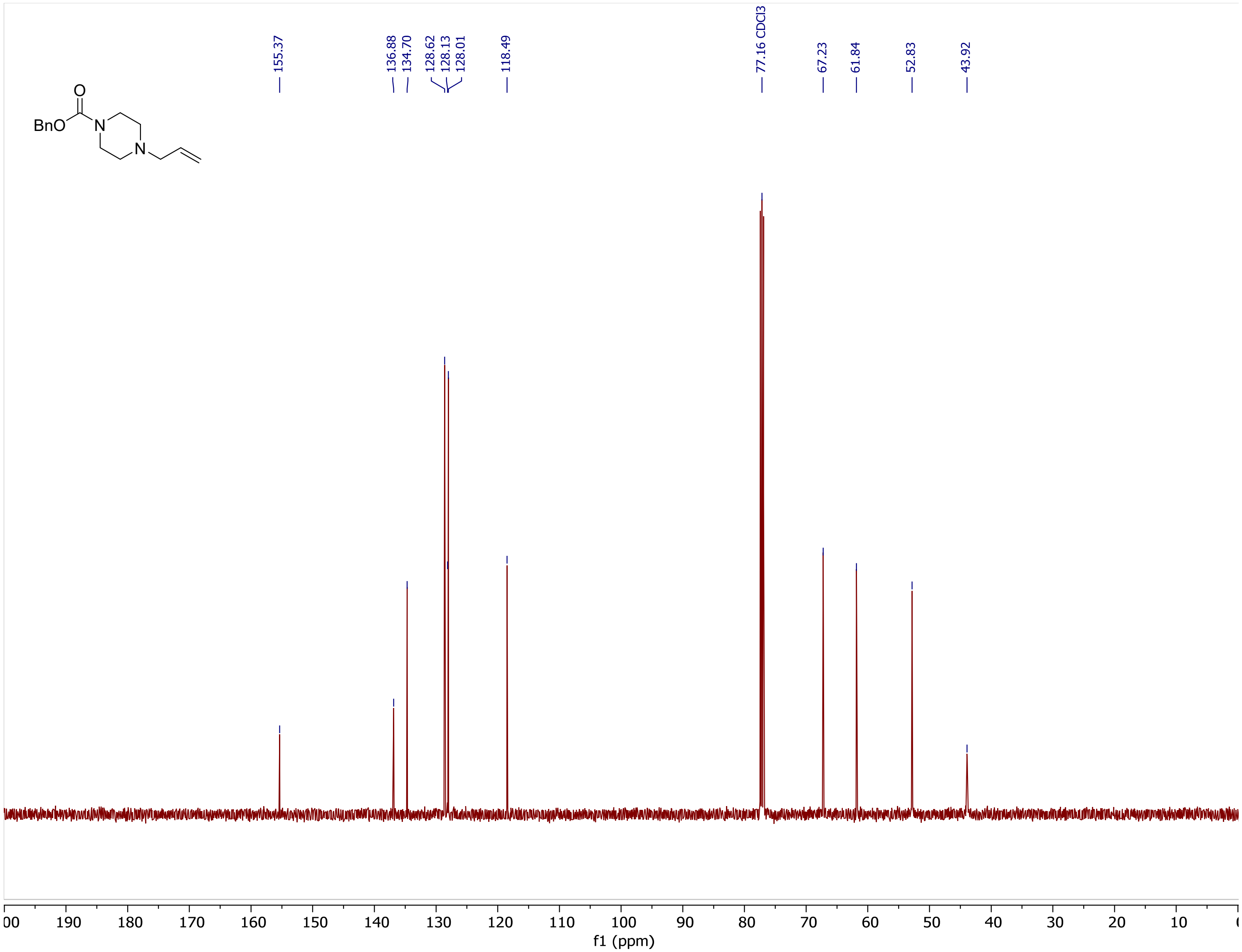

HSQC NMR (500 MHz, $CDCl_3$) spectrum of benzyl 4-allylpiperazine-1-carboxylate (**21**).

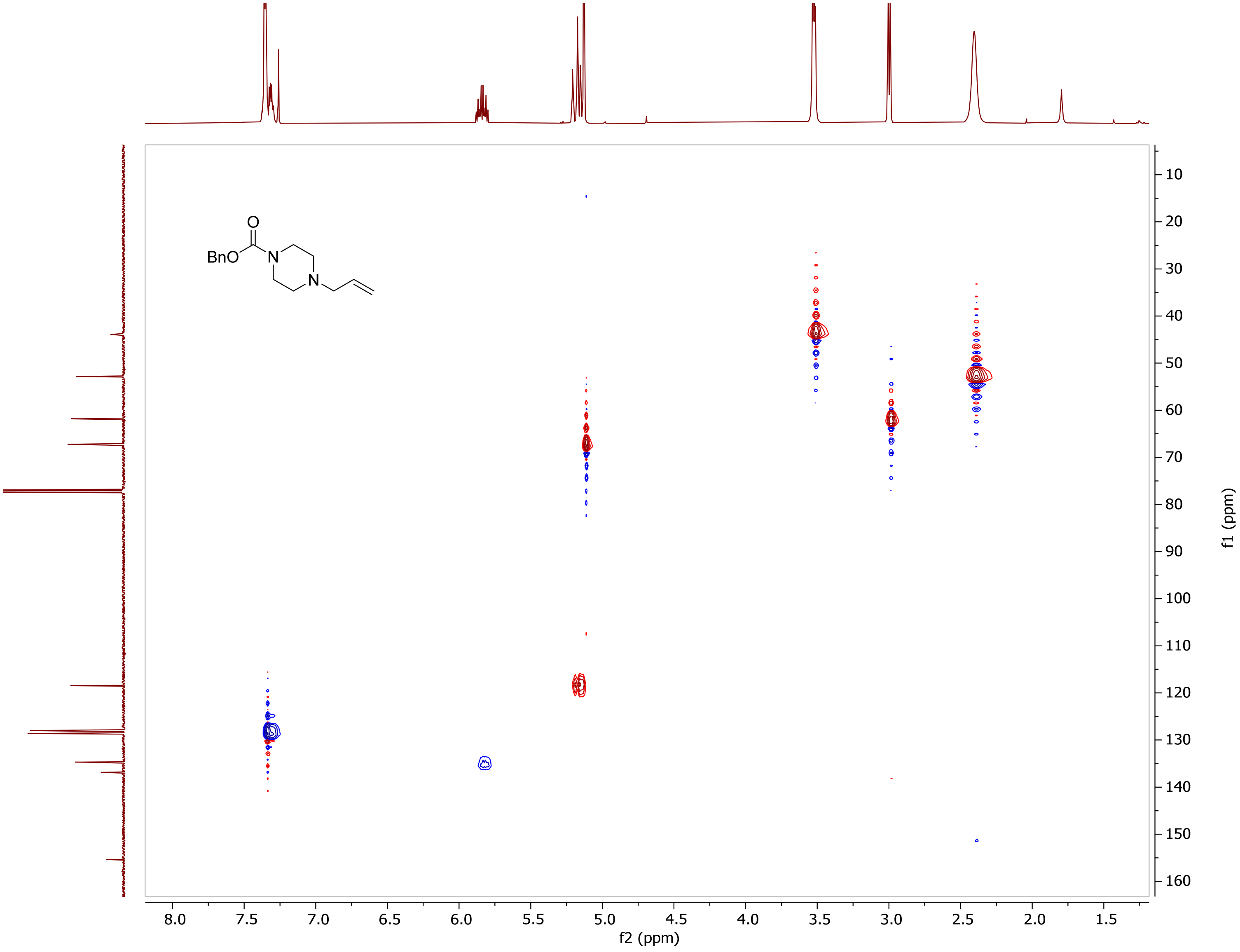

^{1}H NMR (500 MHz, $CDCl_3$) spectrum of benzyl 4-(allyloxy)piperazine-1-carboxylate (**22**).

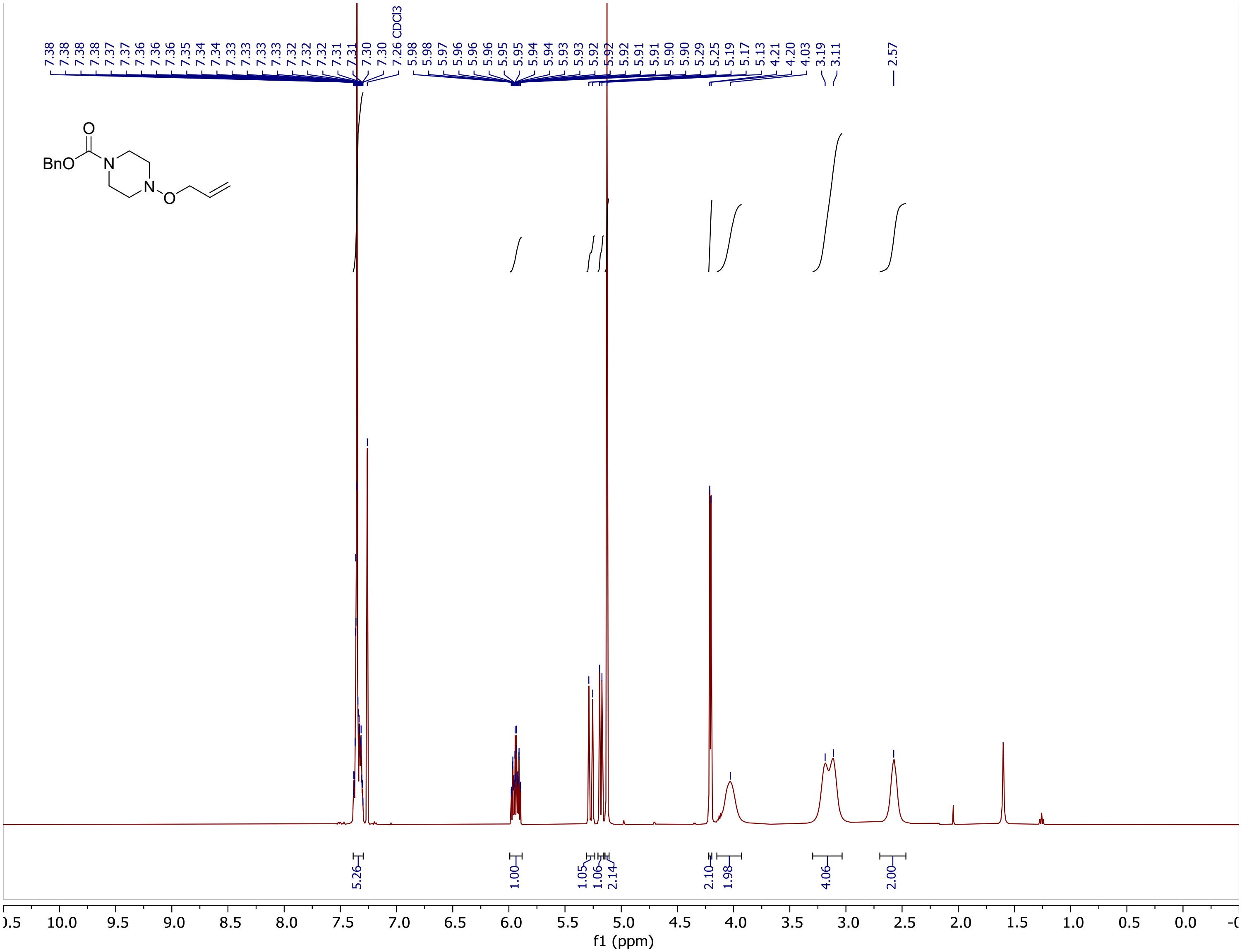

^{13}C NMR (126 MHz, $CDCl_3$) spectrum of benzyl 4-(allyloxy)piperazine-1-carboxylate (**22**).

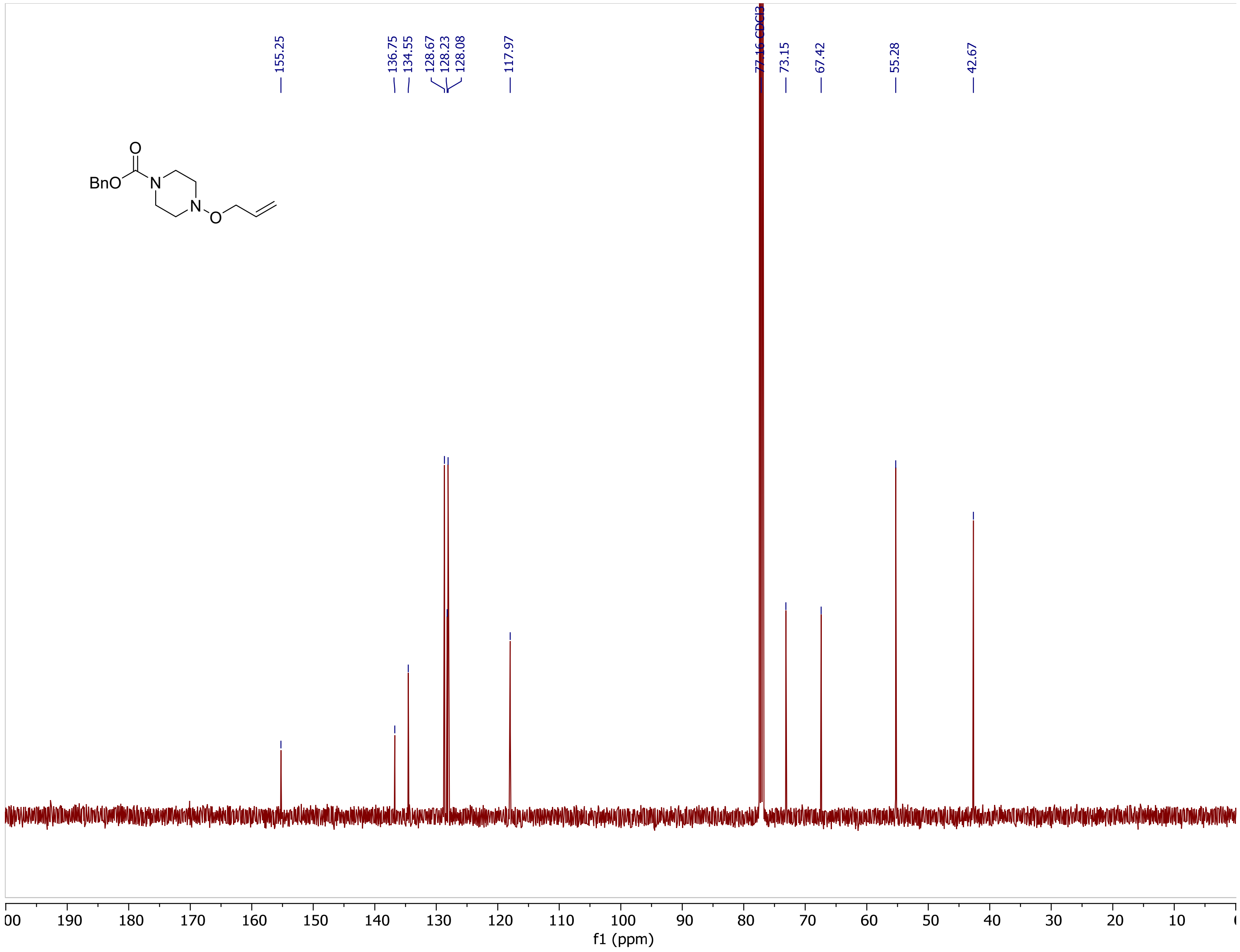

HSQC NMR (126 MHz, $CDCl_3$) spectrum of benzyl 4-(allyloxy)piperazine-1-carboxylate (**22**).

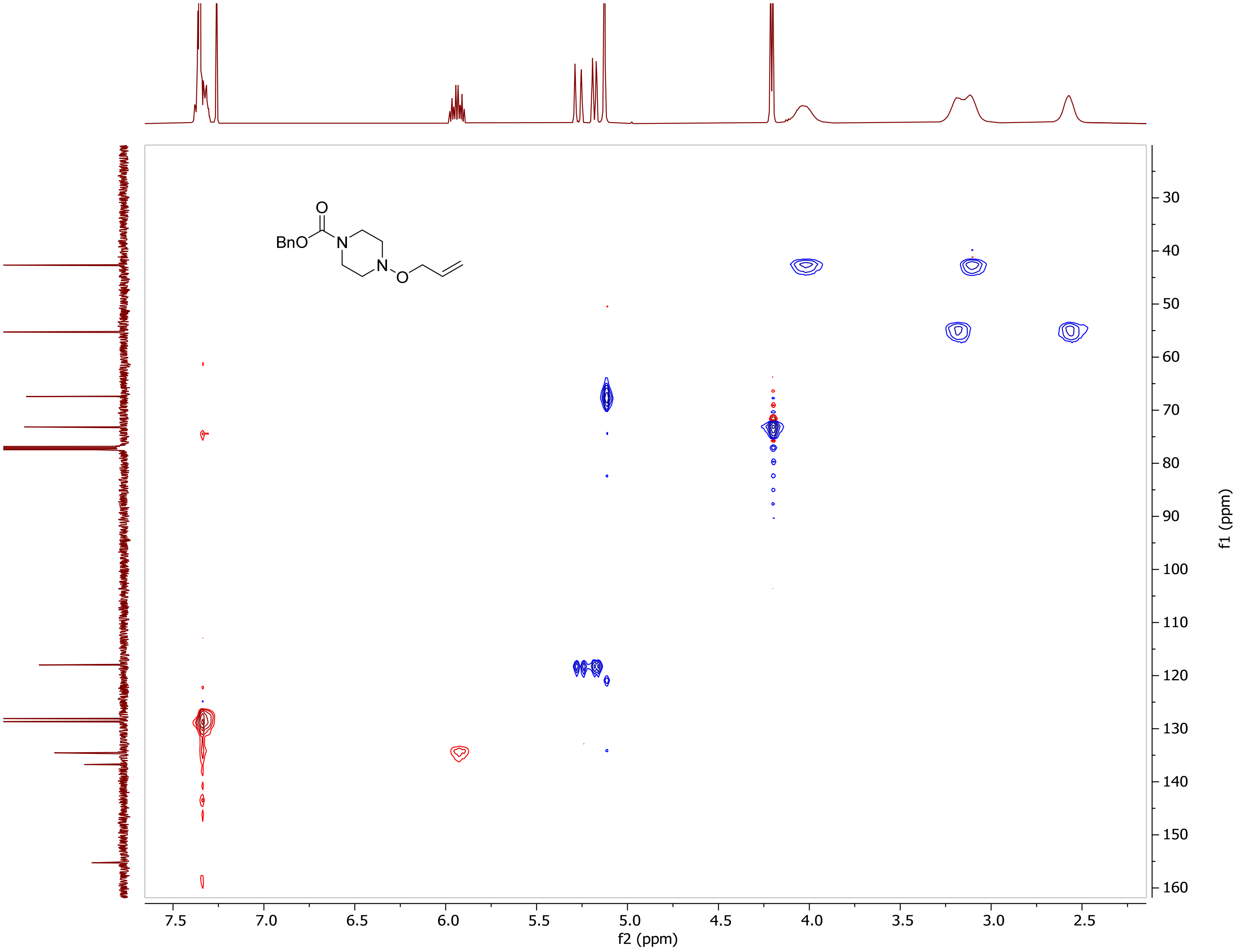

Crude [1]**H NMR** (500 MHz, CDCl$_3$) spectrum of benzyl 4-(2-hydroxyethoxy)piperazine-1-carboxylate.

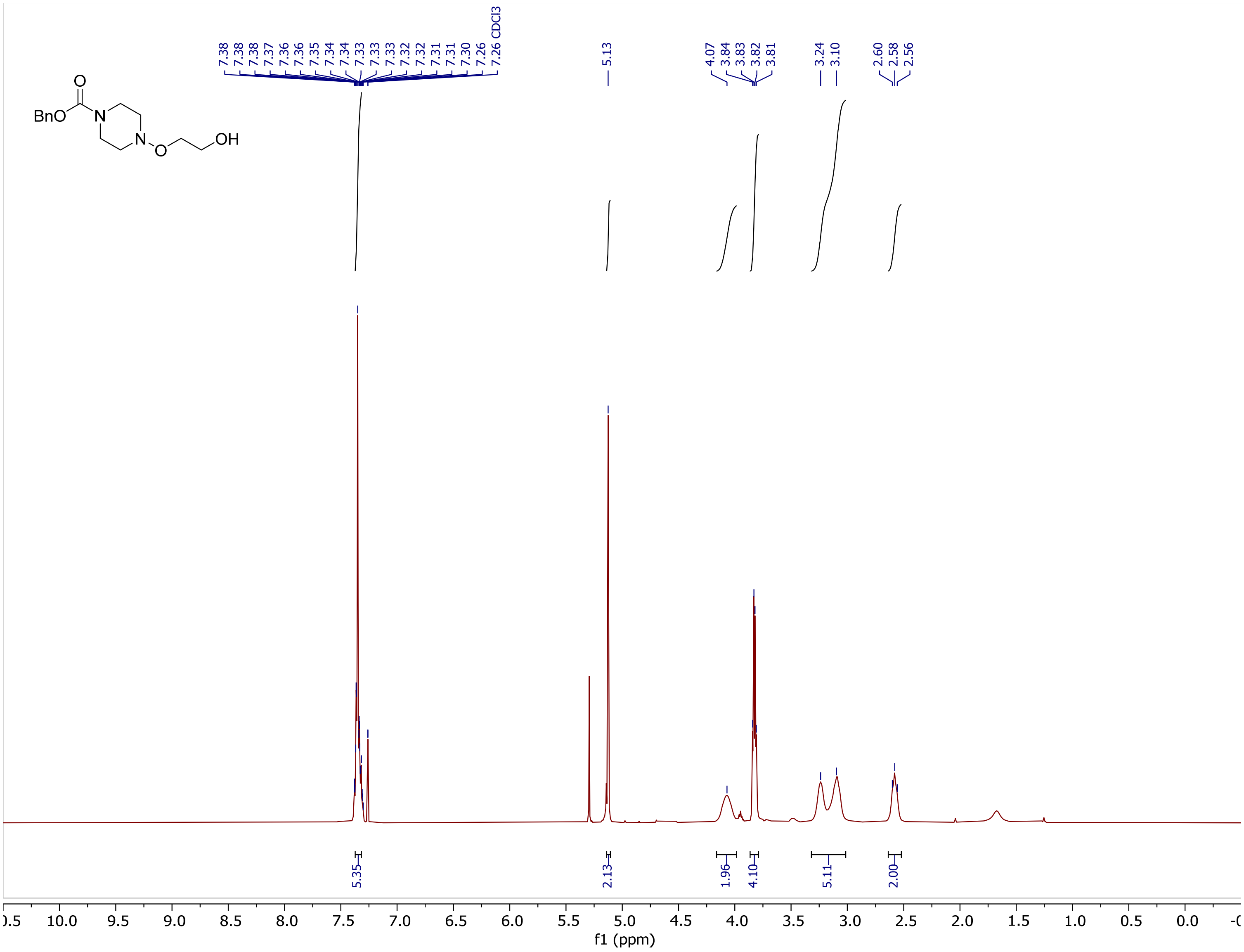

Crude **^{13}C NMR** (126 MHz, $CDCl_3$) spectrum of benzyl 4-(2-hydroxyethoxy)piperazine-1-carboxylate.

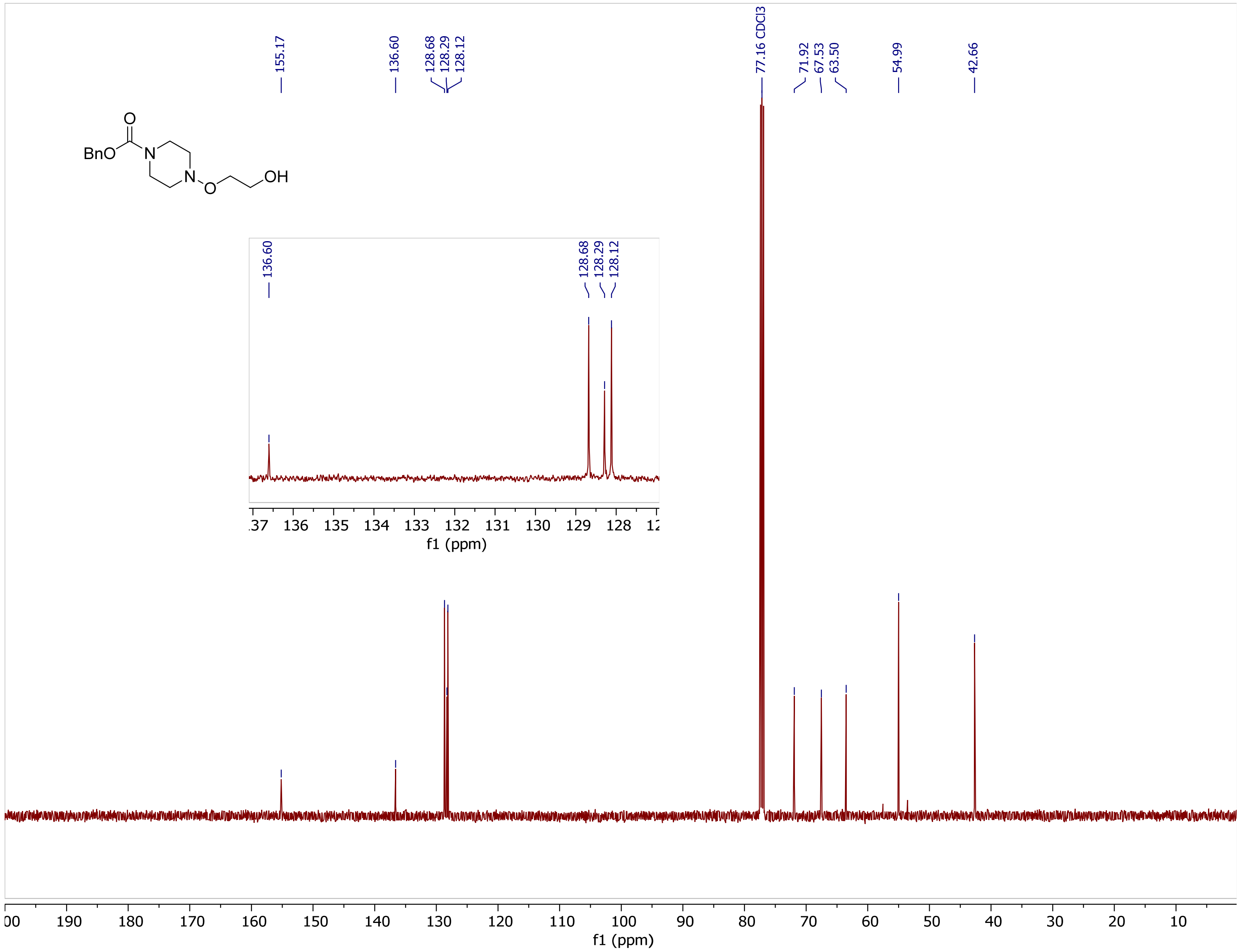

Crude **HSQC NMR** (500 MHz, $CDCl_3$) spectrum of benzyl 4-(2-hydroxyethoxy)piperazine-1-carboxylate.

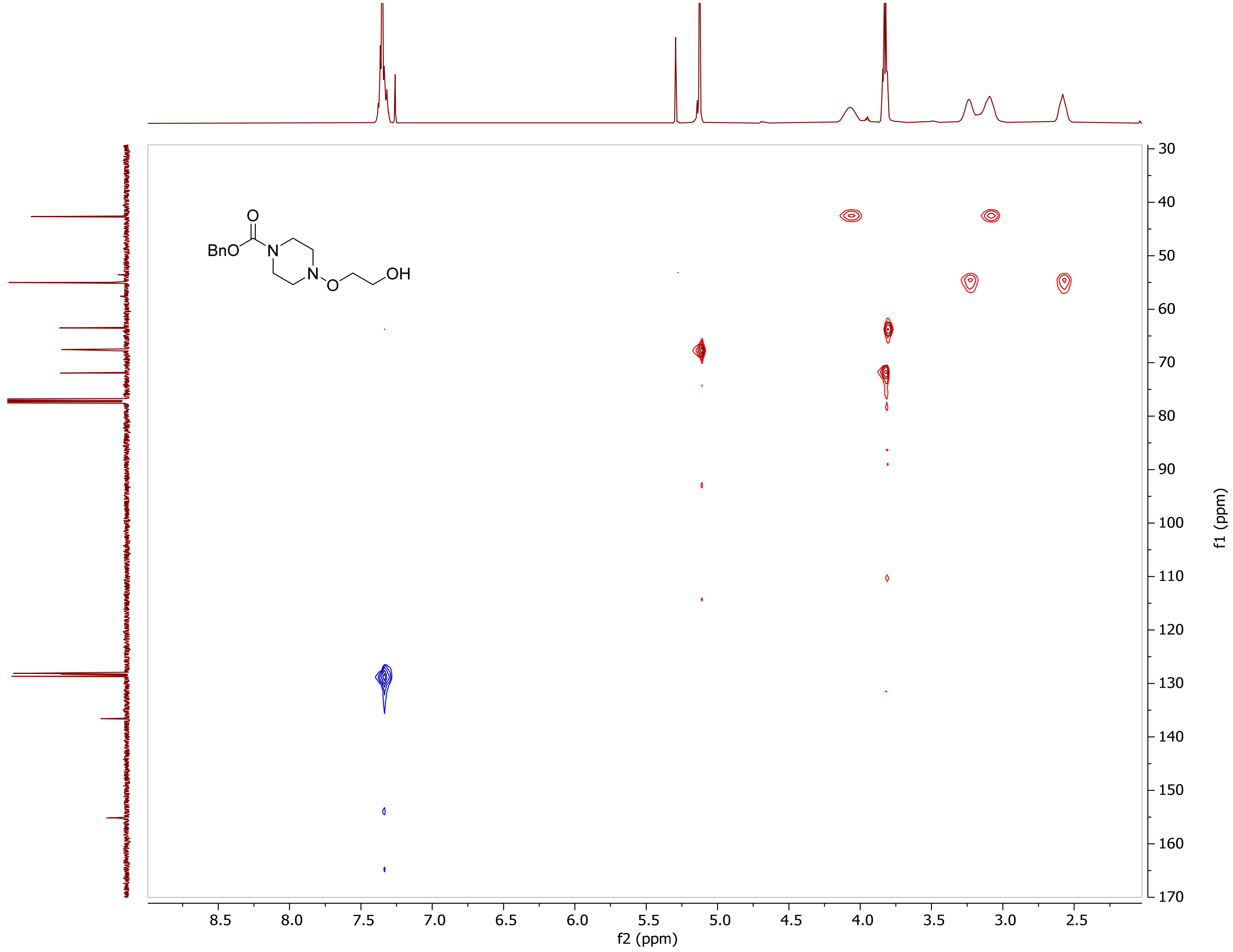

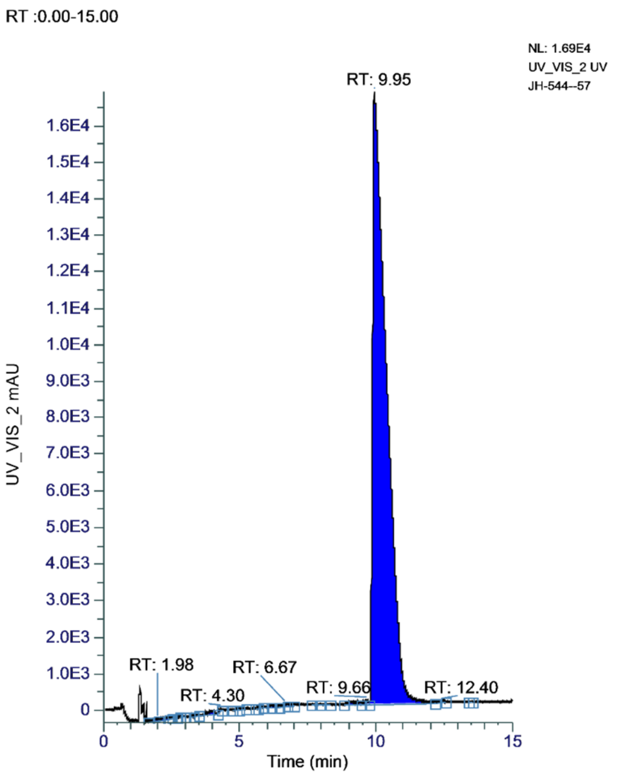

Figure S17. HPLC trace of **6**. HPLC/UV area percent purity of compound **6** at UV = 254 nm was determined to be 98.1% using automated Avalon peak area algorithm (peak list below).

Table S40. Peak List from Avalon peak area algorithm of compound **6** at UV 254 nm.

Retention time (rt) (min)	Start rt (min)	End rt (min)	Peak Area	Area (%)
1.98	1.63	2.02	8.158E+2	0.14
2.27	2.02	2.43	1.25E+3	0.21
2.49	2.43	2.54	4.192E+1	0.03
2.68	2.54	2.74	2.254E+2	0.04
2.98	2.84	3.03	1.006E+2	0.02
3.30	3.19	3.32	7.85E+1	0.01
3.46	3.33	3.51	1.036E+2	0.02
3.89	3.54	4.18	1.375E+3	0.23
4.30	4.20	4.37	3.901E+2	0.06
4.90	4.76	4.98	1.720E+2	0.03
5.17	4.98	5.25	3.004E+2	0.05
5.65	5.56	5.70	7.315E+1	0.01
5.85	5.70	5.88	1.793E+2	0.03
6.14	5.88	6.18	4.673E+2	0.08
6.41	6.18	6.45	4.058E+1	0.09
6.83	6.77	7.03	4.585E+2	0.08
7.75	7.64	7.96	1.453E+2	0.02
8.04	7.96	8.32	1.255E+2	0.02
9.10	8.81	9.46	1.758E+3	0.29
9.66	9.46	9.76	9.846E+1	0.20

9.95	9.76	12.18	5.9E+5	98.12
12.40	12.18	12.58	5.614E+2	0.09
13.46	13.41	13.53	7.734E+1	0.01

Table S41. HPLC method for **6**. Mobile phase A: CH_3CN. Mobile phase B: 0.1% (v/v) formic acid in ultrapure H_2O. Flow rate: 1.000 [$mL\cdot min^{-1}$], injection volume: 0.50 μL.

Time (min)	Mobile Phase A (%)	Mobile Phase B (%)
0	60	40
5	40	60
15	40	60

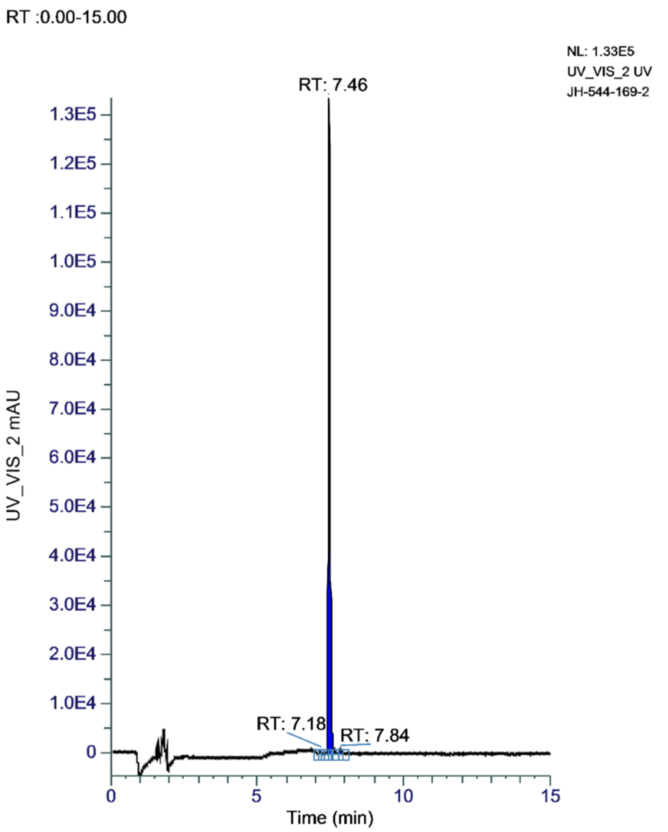

Figure S18. HPLC trace of **7**. HPLC/UV area percent purity of compound **7** at UV = 254 nm was determined to be 99.0% using automated Avalon peak area algorithm (peak list below).

Table S42. Peak List from Avalon peak area algorithm of compound **7** at UV 254 nm.

Retention time (rt) (min)	Start rt (min)	End rt (min)	Peak Area	Area (%)
7.18	7.13	7.26	1.602E+3	0.26
7.46	7.36	7.75	6.15E+3	99.04
7.84	7.75	7.93	4.362E+3	0.70

Table S43. HPLC method for **7**. Mobile phase A: CH_3CN. Mobile phase B: 0.1 % (v/v) formic acid in ultrapure H_2O. Flow rate: 1.000 [$mL \cdot min^{-1}$], injection volume: 0.75 μL.

Time (min)	Mobile Phase A (%)	Mobile Phase B (%)
0	20	80
5	40	60
15	40	60

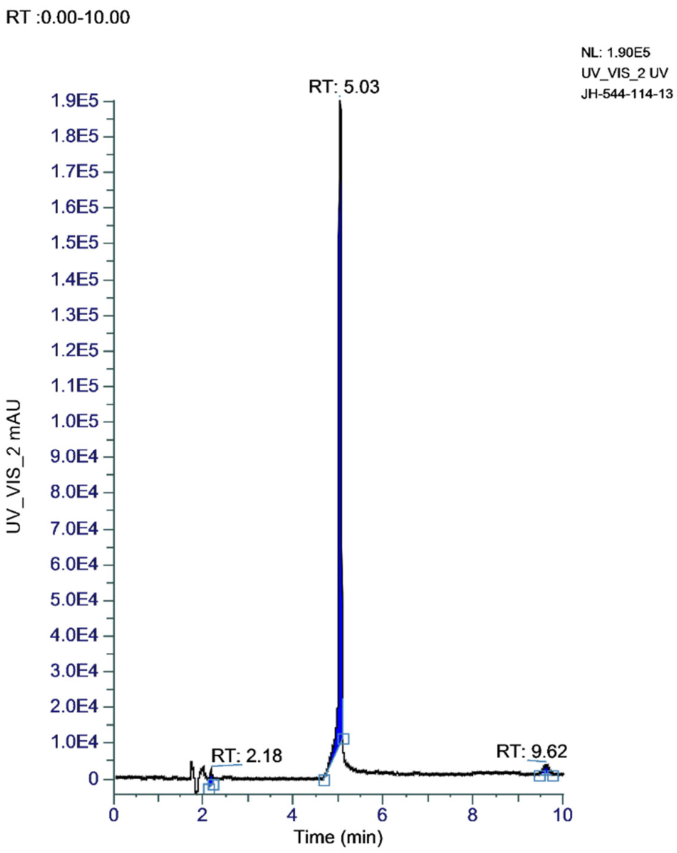

Figure S19. HPLC trace of **8**. HPLC/UV area percent purity of compound **8** at UV = 254 nm was determined to be 95.6% using automated Avalon peak area algorithm (peak list below).

Table S44. Peak List from Avalon peak area algorithm of compound **8** at UV 254 nm.

Retention time (rt) (min)	Start rt (min)	End rt (min)	Peak Area	Area (%)
2.18	2.12	2.22	1.704E+4	2.03
5.03	4.68	5.09	8.041E+5	95.57
9.62	9.47	9.76	2.022E+4	2.40

Table S45. HPLC method for **8**. Mobile phase A: CH_3CN. Mobile phase B: 0.1% (v/v) formic acid in ultrapure H_2O. Flow rate: 1.000 [$mL\cdot min^{-1}$], injection volume 1.0 μL. Sample spiked with 0.1 mL formic acid.

Time (min)	Mobile Phase A (%)	Mobile Phase B (%)
0	10	90
3	40	60
10	40	60

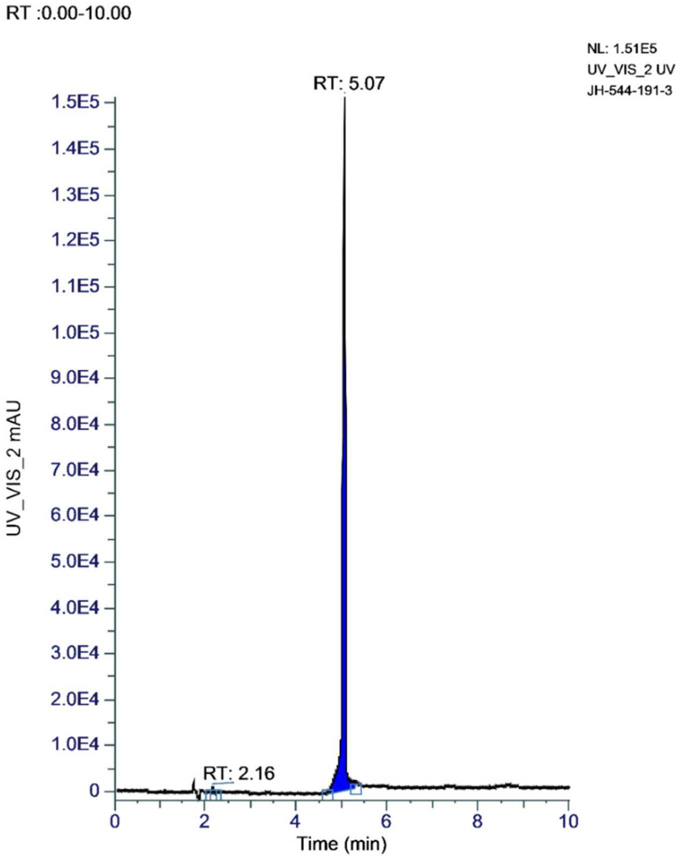

Figure S20. HPLC trace of **9**. HPLC/UV area percent purity of compound **9** at UV = 254 nm was determined to be 99.5% using automated Avalon peak area algorithm (peak list below).

Table S46. Peak List from Avalon peak area algorithm of compound **9** at UV 254 nm.

Retention time (rt) (min)	Start rt (min)	End rt (min)	Peak Area	Area (%)
2.16	2.12	2.21	3.3103E+3	0.47
5.07	4.65	5.29	6.572E+5	99.53

Table S47. HPLC method for **9**. Mobile phase A: CH_3CN. Mobile phase B: 0.1% (v/v) formic acid in ultrapure H_2O. Flow rate: 1.000 [$mL\cdot min^{-1}$], injection volume 0.5 μL. Sample spiked with 0.1 mL formic acid.

Time (min)	Mobile Phase A (%)	Mobile Phase B (%)
0	10	90
3	40	60
10	40	60

5. X-Ray Crystal Structure of **6**

Crystallographic data has been deposited with the CCDC as entry CCDC 2270277.

Table S48. Crystal data and structure refinement for compound **6**.

CCDC Identification code	2270277	
Empirical formula	C43 H48 Cl2 F2 N8 O9	
Formula weight	929.79	
Temperature	297 (2) K	
Wavelength	0.71073	
Crystal system	Triclinic	
Space group	P -1	
Unit Cell Dimensions	a = 13.0590 (17) Å	α = 75.572 (4) °
	b = 13.5834 (18) Å	β = 77.830 (4) °
	c = 13.8183 (18) Å	γ = 67.177 (4) °
Volume	2170.0 (5) $Å^3$	
Z	2	
Density (calculated)	1.423 Mg/m^3	
Absorption coefficient	0.224 mm^{-1}	
F(000)	972	
Crystal Size	0.400 x 0.320 x 0.260 mm	
Theta range for data collection	1.910 to 26.371 °	
Index ranges	-16<=h<=16, -16<=k<=16, -17<=1<=17	
Reflections collected	51999	
Independent reflections	8855 [R(int) = 0.0791]	
Completeness to theta = 25.242°	99.8%	
Absorption correction	Semi-empirical from equivalents	
Max. and min. transmission	0.7454 and 0.5618	
Refinement method	Full-matrix least-squares on F^2	
Data / restraints / parameters	8855 / 10/ 599	
Goodness-of-fit on F2	1.089	
Final R indices [I>2sigma(I)]	R1 = 0.0821, wR2 = 0.2121	
R indices (all data)	R1 = 0.1295, wR2 = 0.2489	
Largest diff. peak and hole	1.032 and -0.441 e.$Å^{-3}$	

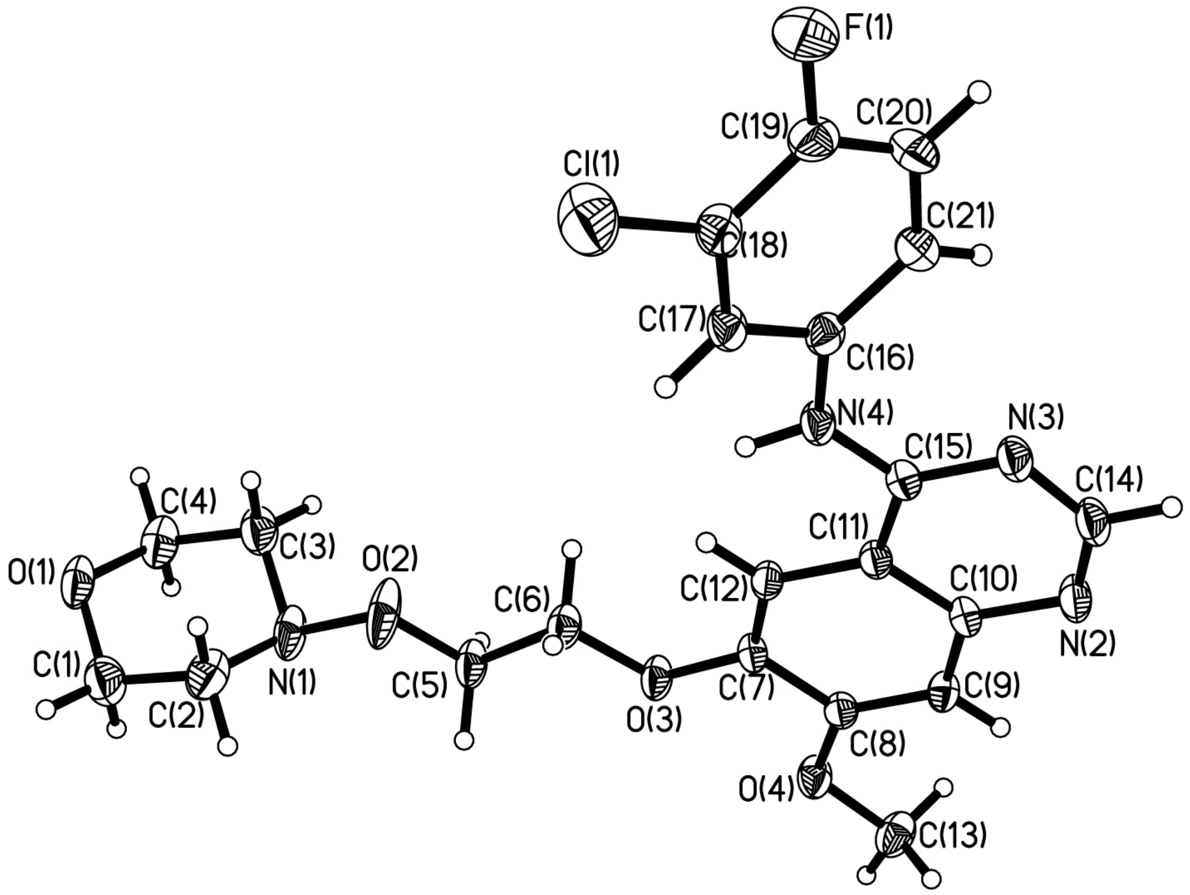

Figure S21. ORTEP drawing of compound **6** with 30% probability for thermal ellipsoids.

6. References

1) Davis, M. I.; Hunt, J. P.; Herrgard, S.; Ciceri, P.; Wodicka, L. M.; Pallares, G.; Hocker, M.; Treiber, D. K.; Zarrinkar, P. P. Comprehensive analysis of kinase inhibitor selectivity. *Nat. Biotechnol.* **2011**, *29* (11), 1046-1051.
